# Sequential Immunization with gp140 Boosts Immune Responses Primed by Modified Vaccinia Ankara or DNA in HIV-Uninfected South African Participants

**DOI:** 10.1371/journal.pone.0161753

**Published:** 2016-09-01

**Authors:** Gavin Churchyard, Koleka Mlisana, Shelly Karuna, Anna-Lise Williamson, Carolyn Williamson, Lynn Morris, Georgia D. Tomaras, Stephen C. De Rosa, Peter B. Gilbert, Niya Gu, Chenchen Yu, Nonhlanhla N. Mkhize, Tandile Hermanus, Mary Allen, Michael Pensiero, Susan W. Barnett, Glenda Gray, Linda-Gail Bekker, David C. Montefiori, James Kublin, Lawrence Corey

**Affiliations:** 1 Aurum Institute for Health Research, Klerksdorp, South Africa; 2 School of Public Health, University of Witwatersrand, Johannesburg, South Africa; 3 Advancing Care and Treatment for TB and HIV, Medical Research Council Collaborating Centre, Klerksdorp, South Africa; 4 University of Kwa-Zulu Natal, Durban, South Africa; 5 Vaccine and Infectious Disease Division, Fred Hutchinson Cancer Research Center, Seattle, WA, United States of America; 6 Institute of Infectious Disease and Molecular Medicine, Division of Medical Virology, University of Cape Town, Cape Town, South Africa; National Health Laboratory Services, Observatory, Cape Town, South Africa; 7 National Institute for Communicable Diseases, National Health Laboratory Services, Sandringham, Johannesburg, South Africa; 8 Duke Human Vaccine Institute, Duke University Medical Center, Durham, NC, United States of America; 9 Department of Laboratory Medicine, University of Washington, Seattle, WA, United States of America; 10 Vaccine Research Program, Division of AIDS, National Institute of Allergy and Infectious Diseases, National Institutes of Health, Bethesda, MD, United States of America; 11 Novartis Vaccines and Diagnostics, Cambridge, MA, United States of America; 12 South African Medical Research Council, Cape Town, South Africa; 13 Perinatal HIV Research Unit, Faculty of Health Sciences, University of the Witwatersrand, Braamfontein, Johannesburg, South Africa; 14 Desmond Tutu HIV Centre, Institute of Infectious Disease and Molecular Medicine, University of Cape Town, Cape Town, South Africa; 15 Laboratory for AIDS Vaccine Research and Development, Duke University Medical Center, Durham, NC, United States of America; Rush University, UNITED STATES

## Abstract

**Background:**

The safety and immunogenicity of SAAVI DNA-C2 (4 mg IM), SAAVI MVA-C (2.9 x 10^9^ pfu IM) and Novartis V2-deleted subtype C gp140 (100 mcg) with MF59 adjuvant in various vaccination regimens was evaluated in HIV-uninfected adults in South Africa.

**Methods:**

Participants at three South African sites were randomized (1:1:1:1) to one of four vaccine regimens: MVA prime, sequential gp140 protein boost (M/M/P/P); concurrent MVA/gp140 (MP/MP); DNA prime, sequential MVA boost (D/D/M/M); DNA prime, concurrent MVA/gp140 boost (D/D/MP/MP) or placebo. Peak HIV specific humoral and cellular responses were measured.

**Results:**

184 participants were enrolled: 52% were female, all were Black/African, median age was 23 years (range, 18–42 years) and 79% completed all vaccinations. 159 participants reported at least one adverse event, 92.5% were mild or moderate. Five, unrelated, serious adverse events were reported. The M/M/P/P and D/D/MP/MP regimens induced the strongest peak neutralizing and binding antibody responses and the greatest CD4+ T-cell responses to Env. All peak neutralizing and binding antibody responses decayed with time. The MVA, but not DNA, prime contributed to the humoral and cellular immune responses. The D/D/M/M regimen was poorly immunogenic overall but did induce modest CD4+ T-cell responses to Gag and Pol. CD8+ T-cell responses to any antigen were low for all regimens.

**Conclusions:**

The SAAVI DNA-C2, SAAVI MVA-C and Novartis gp140 with MF59 adjuvant in various combinations were safe and induced neutralizing and binding antibodies and cellular immune responses. Sequential immunization with gp140 boosted immune responses primed by MVA or DNA. The best overall immune responses were seen with the M/M/P/P regimen.

**Trial Registration:**

ClinicalTrials.gov NCT01418235

## Introduction

In 2012, there were an estimated 2.3 million new HIV infections and 35.3 million people living with HIV globally, of which 71% reside in sub-Saharan Africa.[1] In South Africa, a country with a generalized epidemic with heterosexual intercourse being the main mode of transmission, the prevalence of HIV based on household surveys has increased from 10.6% in 2008 to 12.2% in 2012. The estimated annual HIV incidence among 15–49 year olds was 2.2% in 2002–2005 and declined to 1.72% in 2012 (males 1.21% and females 2.28%).[2] The prevalence of HIV remains high even though the number of new infections are decreasing, largely due to increasing coverage of antiretroviral therapy, longer life expectancy and ongoing transmission.[2–4] The need for an HIV-1 vaccine, particularly in South Africa and other high HIV prevalent countries in sub-Saharan Africa, remains an urgent priority.

In response to the devastating HIV-1 subtype C epidemic in southern Africa, a prime-boost vaccine regimen was developed by the South African AIDS Vaccine Initiative (SAAVI), in collaboration with the University of Cape Town and the United States National Institutes of Health.[5] This regimen includes a DNA prime with HIV-1 subtype C Gag, RT, Tat, Nef and Env inserts (SAAVI DNA-C2) and a boost of modified vaccinia Ankara (MVA), an orthopoxvirus vector containing the same inserts, (SAAVI MVA-C) boost.[6–9] This regimen induced a balanced CD4+/CD8+ response in non-human primates and a strong, predominantly CD4+ T-cell immune response in humans.[5;10;11]

The role of humoral immunity in HIV vaccine prevention has received renewed emphasis, primarily because of the results of the Thai RV144 trial [12;13] and lack of efficacy of recombinant adenovirus 5 vector based vaccines tested in three efficacy trials.[14–16] The phase 3 RV144 HIV vaccine trial evaluated a recombinant canarypox vector vaccine prime (ALVAC B/E) with a B/E gp120 subunit vaccine boost (AIDSVAX) and demonstrated modest protective efficacy and highlighted the potential role of eliciting T-helper and antibody responses in preventing HIV infection.[12;13;17]

The aim of our trial (HVTN 086/SAAVI 103) was to evaluate the safety and immunogenicity of SAAVI DNA-C2, SAAVI MVA-C and Novartis subtype C gp140 with MF59 adjuvant in various combinations and vaccination schedules in HIV-uninfected healthy vaccinia-naïve adult participants in South Africa. The trial builds on the results of HVTN 073/SAAVI 102 (DNA-C2 prime/MVA-C boost), a phase I trial, by including a subunit protein boosting with the aim of inducing stronger antibody responses.[11] We hypothesized that Env protein boosting would allow a comparison of the MVA and DNA vectors to prime antibody responses without dampening the vector-elicited cellular responses.

## Methods

### Study population

This phase 1 multicentre randomised placebo controlled study enrolled HIV-uninfected healthy vaccinia-naïve adults aged 18 to 45 years in 3 sites within South Africa (Soweto, Klerksdorp and Cape Town). Participants were partially blinded to treatment assignment (described below) the study and assigned to 4 groups with a vaccine and placebo arm in each group (Table 1). Treatment group 1 received an MVA prime (months 0 and 1) with sequential gp140 boost (months 3 and 6) (M/M/P/P); Group 2 received concurrent MVA/gp140 vaccinations (months 0 and 3) (MP/MP); Group 3 received a DNA prime (months 0 and 1) with sequential MVA boost (months 3 and 6) (D/D/M/M); Group 4 received a DNA prime (months 0 and 1) with concurrent MVA/gp140 boost (months 3 and 6) (D/D/MP/MP).

**Table 1:** Vaccination schedule.

Treatment Group	Number participants	Month 0	Month 1	Month 3	Month 6
**Group 1 (T1: M/M/P/P)**	38	Placebo+ MVA-C	MVA-C	Placebo + gp140/MF59	Placebo + gp140/MF59
	8	Placebo + Placebo	Placebo	Placebo + Placebo	Placebo + Placebo
**Group 2 (T2: MP/MP)**	38	MVA-C + gp140/MF59	Placebo	MVA-C + gp140/MF59	Placebo + Placebo
	8	Placebo + Placebo	Placebo	Placebo + Placebo	Placebo + Placebo
**Group 3 (T3:D/D/M/M)**	38	Placebo + DNA-C2	DNA-C2	Placebo + MVA-C	Placebo + MVA-C
	8	Placebo + Placebo	Placebo	Placebo + Placebo	Placebo + Placebo
**Group 4 (T4:D/D/MP/MP)**	38	Placebo + DNA-C2	DNA-C2	MVA-C + gp140/MF59	MVA-C + gp140/MF59
	8	Placebo + Placebo	Placebo	Placebo + Placebo	Placebo + Placebo

Placebo for DNA-C2, MVA-C and gp140 was sodium chloride for injection, 0.9%.

### Eligibility criteria

Healthy adults of relatively ‘low risk’ of acquiring HIV, based on self-reported risk behaviour in the 12 months prior to enrolment (sexual abstinence, monogamous relations and regular condom use), were recruited from the general community and were eligible for enrolment. The exclusion criteria included a history or evidence of vaccinia virus (smallpox) vaccination, previous participation in HIV vaccine trials, live attenuated vaccines in the past 30 days, any sexually transmitted infections in the previous 12 months and any medical, psychiatric or occupational condition of concern to the investigators. Pregnant or breastfeeding women were ineligible and all women had to agree to consistent use of effective contraception (2 methods—barrier and other effective method like hormonal contraception). The protocol was amended to discontinue MVA vaccinations after observing floculations in MVA vials.

### Regulatory approvals and trial registration

Written informed consent in either English or local language was obtained from all participants. The study was approved by the South African Medicines Control Council and the Biomedical Research Ethics Committees of the University of the Witwatersrand, University of Cape Town and the University of KwaZulu Natal. Trial registration number NCT01418235 (ClinicalTrials.gov), SANCTR DOH-27-111-3540 (South Africa National Clinical Trial Register) and SA NHREC #2540 (South African National Health Research Ethics Council).

### Randomisation and blinding

Participants were randomised to one of the four groups in a 1:1:1:1 ratio allocation, whilst randomisation to vaccine versus placebo within each group used a 38:8 allocation. The randomisation sequence was obtained by computer-generated random numbers and distributed to each site via a web-based randomisation system. The randomization was stratified by site. Participants and site staff (except for site pharmacists) were blinded to assignment to receipt of vaccine versus placebo within each treatment group, but were unblinded in terms of assignment to treatment groups with a MVA prime (M/M/P/P or MP/MP) or a DNA prime (D/D/M/M or D/D/MP/MP). Laboratory staff was blinded to all assignments.

### Vaccine regimens

DNA or placebo DNA, MVA or placebo MVA vaccinations were administered in the right deltoid, whereas gp140 with MF59 or placebo gp140 was administered in the left deltoid. Placebo for DNA-C2, MVA-C and gp140 was sodium chloride for injection, 0.9%. At some visits participants received bilateral deltoid injections.

### Safety assessment

Vaccination with vaccinia virus is associated with increased risk of myopericarditis.[18–21] Although myopericarditis has not been reported with vaccination with MVA, this protocol included safety measures to reduce the possible risk of such an adverse event. Eligibility criteria excluded individuals with cardiac disease, or 2 or more cardiac risk factors, such as elevated blood cholesterol, current cigarette smoking, body mass index of 35 or greater, or family history of early coronary artery disease. Also excluded were individuals with Troponin T above institutional upper limit of normal, or whose screening electrocardiogram had clinically significant findings or features that would interfere with the assessment of myopericarditis (e.g., ST segment or T wave abnormality).

Throughout the study, participants underwent safety evaluations including physical examinations, and standard serum chemistry and hematological tests. Local injection site reactogenicity (pain, tenderness, erythema and induration) and systemic events (malaise/fatigue, headache, fever, chills, myalgia, arthralgia, nausea, and vomiting) were assessed for 3 days following each vaccination until resolution. Adverse events were recorded for each participant for 12 months following the first vaccination. In addition, due to the evaluation of the investigational protein vaccine adjuvant formulation, specific monitoring was conducted for Adverse Events of Special Interest (AESIs) such as autoimmune diseases. Severity of adverse events and reactogenicity were graded according to standard criteria (http://rcc.tech-res.com/safetyandpharmacovigilance/).

Cardiac troponin T was measured at 2 weeks after each potential MVA vaccination time-point, and participants with any cardiopulmonary symptoms underwent physical examination and 12 lead electrocardiogram (ECG). As a consequence of maintaining the study blind, some cardiac evaluations may have been performed post-vaccination with DNA, protein or placebo, rather than solely post-vaccination with MVA/MVA-placebo.

ECGs were obtained locally and transmitted electronically to the Saint Louis University Core ECG Laboratory for interpretation for consistency of interpretation across multiple network and non-network studies of vaccinia virus -vectored vaccines. Sites also ensured availability of a local cardiologist for consultation for cases requiring additional evaluation. US Centres for Disease Control case definition of myopericarditis was used.[20]

### Study products

#### SAAVI DNA-C2 and MVA-C

SAAVI DNA-C2 (4mg/ml) was manufactured by Althea Technologies, Inc. (San Diego, USA) and contained an equimolar mixture (w/w) of two plasmids: pVRCgrttnC that expressed an HIV-1C polyprotein comprising Gag-Reverse Transcriptase-Tat-Nef (Grttn); and pVRCgp150CT that expressed an HIV-1 clade C Env (strain Du151) with 372 bases deleted from the 3' end (cytoplasmic tail deletion). The SAAVI MVA-C (2.9 x 10^9^ pfu) is a recombinant MVA expressing the same immunogens as the DNA vaccine with Grttn inserted into Del III region under the 40K promoter, and gp150CT into the 49/50 region under the I3 promoter of the same MVA and was manufactured by Therion Biologics (Cambridge, MA, USA). Vaccine inserts were cloned from viral isolates obtained from individuals within 3 months of HIV infection and selection was based on genetic relatedness to a South African HIV-1 clade C consensus sequence.[6] The *RT*, *tat* and *nef* genes were inactivated for safety, and all genes were human codon-optimized and modified for expression levels, stability and immunogenicity.[7;8]

#### Subtype C gp140 / MF59 vaccine

The gp140 protein subunit vaccine used for these studies was a recombinant oligomeric V2-deleted envelope glycoprotein derived from the South African subtype C strain TV1. The construct used for production expresses an oligomeric gp140 antigen with a partial sequence deletion in the second variable loop (V2) and was designed to better expose epitopes in the receptor and co-receptor binding regions of the glycoprotein.[22] The gp140 protein was produced in Chinese hamster ovary (CHO) cells and was purified to homogeneity and characterized extensively as described previously.[23] A 100 mcg dose was combined with the MF59® adjuvant and administered as a single 0.5 mL IM injection into the deltoid. MF59 is an oil-in-water emulsion with a squalene internal oil phase and an external aqueous phase.[24] The placebo was Sodium Chloride for Injection, 0.9%.

### Laboratory assays

#### Intracellular Cytokine Staining (ICS)

Cryopreserved peripheral blood mononuclear cells (PBMC) were thawed and incubated overnight before stimulation. PBMC were stimulated with 9 potential T cell epitope (PTE) peptide pools, three Env, two Gag, three Pol and one Nef. The peptide diluent (0.5% DMSO) was used as a negative control. PBMC stimulated with PHA and a CMV peptide pools were used as positive controls. ICS was performed using a validated 12-color protocol as previously described.[25] Subjects with high background responses in the negative control (>0.1% of T-cells expressing IFN-γ and/or IL-2) were excluded from analysis.

#### HIV-1 specific binding antibody assay

Plasma IgG HIV-1 specific antibodies to HIV-1 gp120/gp140 proteins and V1/V2 scaffolds were measured by a binding antibody multiplex assay as previously described.[26–28] Positive controls included a HIVIG and CH58 mAb IgG titration.[29] Negative controls were blank, MulVgp70_His6 (empty gp70 scaffold) coupled beads, and HIV-1 negative sera. Antibody measurements were acquired on a Bio-Plex instrument (Bio-Rad, Hercules, CA) with a Mean Fluorescent Intensity (MFI) readout. The following antigens were used; Group M consensus: ConSgp140CFI [30;31], Con6gp120/B; Subtype C Envelopes: 1086Cgp140C_avi; V1-V2 Antigens: gp70_B.CaseA2 V1/V2 and C.1086C_V1_V2 Tags (provided by Drs. Liao/Haynes, Duke University); and o-gpTV1deltaV2 gp140 (provided by Novartis).

Serum HIV-1-specific IgA responses (1/50 final dilution) from IgG-depleted samples against the HIV-1 gp140 (Con S gp140 CFI, TV1c8.2_21 gp140C_avi), gp41, HIV-1 gp120 (Con 6 gp120/B, TV1c8_D11gp120.avi/293F), p24 Gag antigens were also measured as described above.[26]

#### Neutralizing antibody assay

Neutralizing antibodies against HIV-1 were measured at various time points (see immunogenicity section) as a function of reductions in Tat-regulated luciferase (Luc) reporter gene expression after a single round of infection with Env-pseudotyped viruses in TZM-bl cells as described.[32] Neutralization was assessed against three Tier 1A laboratory-adapted viruses that exhibit a highly sensitive neutralization phenotype (Clade B: MN.3, SF162.LS; Clade C: MW965.26), and against the two Env vaccine strains (Clade C: Du151 and TV1.21), which have a Tier 2 neutralization phenotype that is typical of most circulating strains.[33]

#### HIV infections and vaccine-induced seropositivity

HIV infection was assessed at multiple time points (visit/day: 4/14, 9/98, 12/182, 13/273, 14/364) during the study using the HVTN In-Study diagnostic algorithm, which utilizes a single EIA test, the BioRad GenScreen Ultra HIV Ag-Ab HIV 1/2 EIA. On samples with a reactive EIA, Western Blot (BioRad Genetic Systems HIV-1 Western Blot) and RNA PCR (Roche Taqman v2 PCR) are run to distinguish vaccine-induced responses from actual infection.

Vaccine-induced seropositivity was assessed using ELISA (Abbott Axsym HIV Ag/Ab Combo, BioRad GenScreen Ultra HIV Ag-Ab HIV 1/2, BioRad Multispot HIV-1/HIV-2 Rapid Test, bioMerieux Vironostika HIV Ag/Ab HIV ½) and Western Blot (BioRad Genetic Systems HIV-1) assays.

### Statistical methods

#### Sample size calculations

With 38 participants per vaccine arm, there was a 90% chance of observing at least 1 adverse event if the true rate of such an adverse event was 5.9% or more; and there was a 90% chance of observing no adverse events if the true rate was 2.7% or less. The sample size calculations for immunogenicity were based on the neutralization area under the magnitude-breadth curve (AUC-MB) endpoint. With 38 participants per vaccine group and 32 participants in the pooled placebo group, there was at least a 90% probability that any vaccine regimen with a mean neutralisation AUC-MB of at least 1 standard deviation larger than that of the pooled placebo group would be detected, based on a two-sided p-value below 0.05 from a two-sample t-test with common variance across the study arms, and after multiplicity adjustment via Dunnett’s procedure [34]. These calculations allow for up to 10% of vaccinees with missing immune response data.

The primary analysis also ranks the four vaccine regimens by the estimated mean AUC-MB. Probabilities of correctly selecting the truly best vaccine arm (with highest mean AUC-MB) were estimated using thousands of vaccine trials simulated in the following way. First, one vaccine arm was simulated using a normal distribution with mean AUC-MB of mu1 = 1.04 and standard deviation (SD) of 0.204, matching the results observed in the VaxGen 004 trial. The other vaccine arms were simulated using the same SD and different mean values mu2, mu3, and mu4 ranging from 1.04 to 1.34 (1.34 versus 1.04 represents a 2-fold increase in average neutralization titer). The results show that there is at least 90% power to select a vaccine arm with mean AUC-MB within 1.2-fold of the truly best vaccine arm. For secondary analyses that compare positive immune response rates between pairs of vaccine arms, there is 80% power to detect differences of 40% vs. 82%, 50% vs. 90%, and 60% vs. 96%; these calculations allow for 10% missing data and use an exact test with Type I error rate 0.05/6 to control for the 6 pairwise comparisons.

#### Statistical analysis

All data from enrolled participants who received at least one vaccination were analysed, and participants were analysed according to their assigned treatments (intention to treat). A per protocol analysis in participants receiving all vaccinations within protocol-specified visit windows was also done but the data are not shown. Analyses were performed using Statistical Analysis Software (SAS) version 9.2 and R version 2.15.1 statistical software.

Reactogenicity: The number and percentage of participants experiencing each type of reactogenicity sign or symptom were tabulated by severity and vaccine regimen. For a given systemic or local sign or symptom, each participant's reactogenicity was counted once under the maximum severity for all injection visits. For each reactogenicity event type, the Kruskal-Wallis rank sum test was used to test for overall differences between each vaccine group versus placebo and among the four vaccine groups after converting each outcome to an integer variable ranging from 1 to N, where N is the number of event categories. The placebo groups were pooled to provide more statistical power. P-values not adjusted for multiple testing were used in order to maximize power to detect elevated reactogenicity in vaccine arms.

Adverse Experiences: AEs were classified using MedDRA preferred terms. The number and percentage of participants experiencing each specific AE were tabulated by severity and by relationship to treatment. Each participant’s AE was counted once under the maximum severity or the assessed relationship to study product.

Immunogenicity: For each Env antigen or peptide pool, rates of positive response for neutralization by TZM-bl, binding IgG by binding antibody multiplex assay, and CD4 and CD8 response by ICS, were estimated for each group (the 4 vaccine groups and the pooled placebo group) and time-point (Day 0 (visit 2), 98 (visit 9), 182 (visit 12), 364 (visit 14). The primary time-point was 2 weeks post last vaccination, equal to visit 9 for MP/MP and to visit 12 for M/M/P/P, D/D/M/M, and D/D/MP/MP. The decay kinetic time point for all vaccine groups was day 364 (visit 14). Boxplots were used to describe distributions of responses to each Env antigen or peptide pool for each treatment group, for the peak immunogenicity and decay kinetics (visit 14) time-points. All of the analyses were conducted for the primary time-point and the decay kinetics time-point. Wilcoxon signed-rank tests were used to assess significant waning of response levels from the peak to the decay kinetics time-points (paired data). These tests were applied for each vaccine arm separately, given the objective to assess waning for each vaccine arm.

### Endpoints

#### Neutralizing antibody

Response to a particular Enveloped pseudovirus was considered positive if the neutralization titer was ≥ 10, where a titer was defined as the serum dilution that reduces relative luminescence units (RLUs) by 50% relative to the RLUs in virus control wells (cells + virus only) after subtraction of background RLU (cells only). For the TZM-bl assay, the area-under-the-magnitude-breadth curve (AUC-MB) of neutralization titers to the panel of 3 Tier 1 and 2 Tier 2 pseudoviruses was computed for each participant with evaluable neutralization data [35]. Dunnett’s procedure using two-sample t-statistics with common variance across the study arms was applied with overall 2-sided alpha = 0.05 to determine which of the 4 vaccine groups had a significantly higher mean AUC-MB than the pooled placebo group, using Liu’s (1997) formula.[34] This procedure was applied to construct 95% simultaneous confidence intervals (CIs) about the 4 differences in mean AUC-MBs for each vaccine group versus placebo (vaccine–placebo), which determines which vaccine arms have familywise-error rate adjusted 2-sided p-value < 0.05. In addition, an F-test was used for assessing any differences in mean AUC-MBs among the 4 vaccine groups, which assumes normally distributed outcomes and a common variance across the study arms. Then, the 6 pairwise vaccine group differences in mean AUC-MBs were estimated with pointwise and simultaneous 95% CIs (computed using t-statistics and Tukey’s procedure), and Holm-Bonferroni adjusted p-values from unequal-variance t-tests were used for testing different mean AUC-MBs between vaccine arms accounting for the six pairs of vaccine arm comparisons. Comparisons were first done for each vaccine arm versus placebo to answer whether there are any vaccine-induced responses to HIV, and secondly done among vaccine arms to answer whether responses differ among the vaccine regimens.

The neutralization positive response rate was compared between each vaccine group with unadjusted and Holm-Bonferroni adjusted Fisher’s exact test together with unadjusted and simultaneous 95% Wilson confidence intervals. Then, a Chi-squared test was used to test for any differences in response rate among the 4 vaccine groups, and the Fisher’s and Wilson procedures were applied to compare response rates among the 6 pairs of vaccine groups. To assess magnitudes among positive responders, the above procedures were repeated with t-test based 95% confidence intervals (difference in sample means plus or minus the t-distribution critical value multiplied by s, where s is the square-root of s1^2^/n1 + s2^2^/n2, with s1 and s2 the sample standard deviations for the two groups being compared. In addition, the Kruskal-Wallis procedure was used for testing any differences among the 4 vaccine groups.

#### HIV-specific binding antibody

The same statistical procedures as described for neutralizing antibodies were used to compare IgG antibodies among groups.

#### Intracellular cytokine staining

Positivity for a peptide pool was based on a one-sided Fisher’s exact test comparing the percentage of T cells with positive staining for IFN-γ and/or IL-2 between the experimental and negative control wells, with a discrete Bonferroni multiplicity adjustment for the 9 peptide pools. If the adjusted p-value for a peptide pool is ≤0.00001, the response to the peptide pool for the T-cell subset is considered positive. If any peptide pool for a T-cell subset was positive then the overall response was considered positive. The protein-specific magnitude of response was the maximum for the protein-specific pools, with the overall magnitude being the sum of the protein magnitudes.

The polyfunctionality of CD4+ and CD8+ T-cells was assessed by measuring the proportion of T-cells expressing the following functional markers alone or in various combinations: IFN-γ, IL-2, TNF-α, CD40L, granzyme B. Cells expressing granzyme B alone without another function were excluded, since granzyme B is constitutively expressed, even in T cells that are not induced to express functional markers in the assay. Polyfunctionality analysis was only performed for CD4+ and CD8+ T-cell responses that are positive for IFN-γ and/or IL-2, which results in the numbers of data points in each graph varying.

## Results

### Participant accrual and demographics

**Fig 1:**
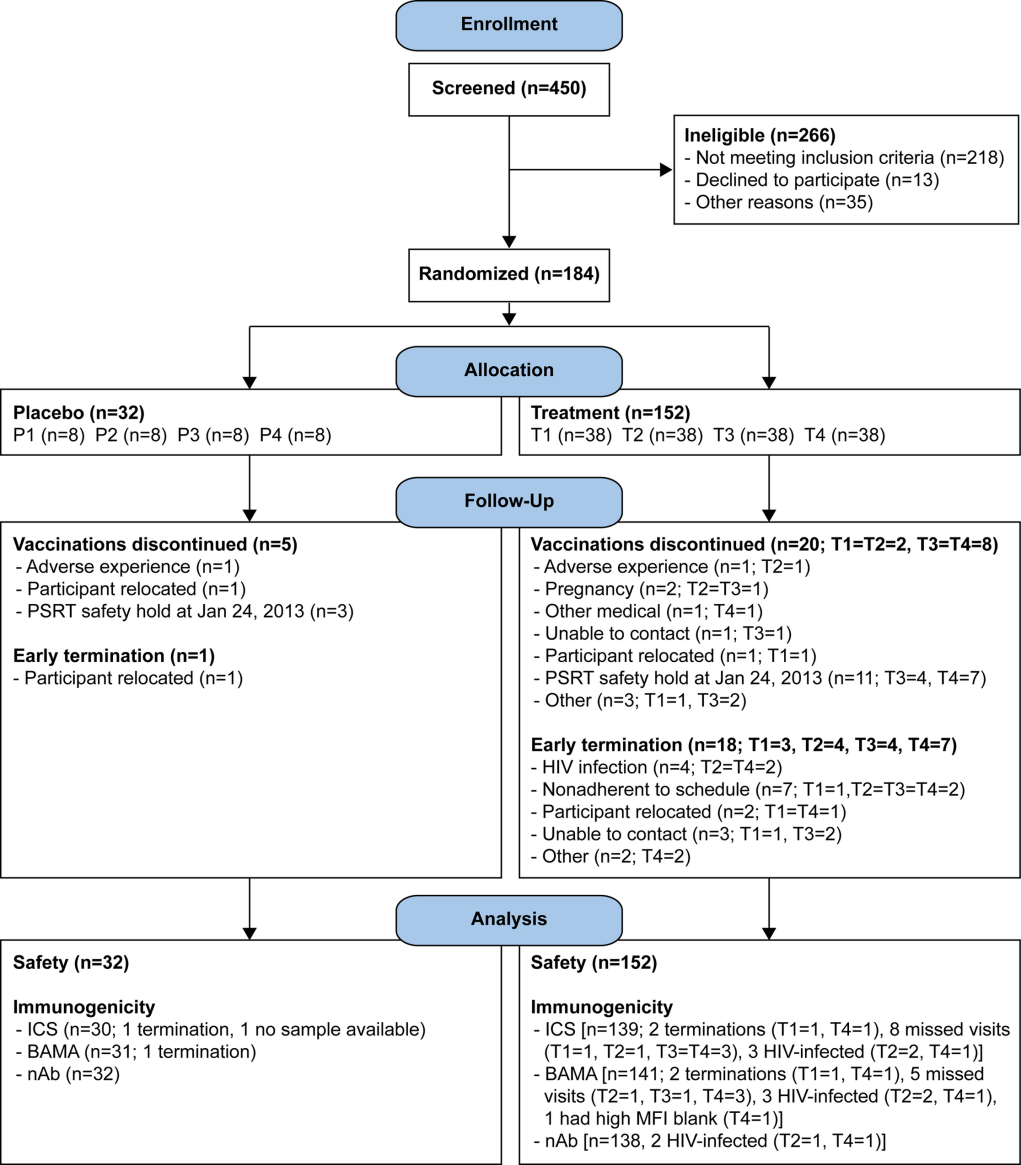
CONSORT flow diagram: number of individuals assessed for eligibility, enrolled and randomized to vaccine or placebo, followed-up and analysed. T1: MVA prime, sequential gp140 boost (M/M/P/P); T2 (MP/MP): concurrent MVA/gp140; T3 (D/D/M/M): DNA prime, sequential MVA boost; T4 (D/D/MP/MP): DNA prime, concurrent MVA/gp140 boost) or placebo. P1-P4 = placebo.

Participants were enrolled between December 2011 and October 2012, the last vaccination was administered in March 2013 and the last visit occurred in October 2013. The study enrolled 184 participants from 3 study sites: Cape Town (n = 61), KOSH (n = 62), Soweto (n = 61). The participant flow is shown in Fig 1.

Overall, 95 (52%) participants were female, and all were Black/ African, with a median age of 23 years (range, 18–42 years) (Table 2). Baseline demographics were similar across the study groups (Table 2).

**Table 2:** Participant characteristics.

	Placebo (P) (n = 32)	M/M/P/P (T1) (n = 38)	MP/MP (T2) (n = 38)	D/D/M/M (T3) (n = 38)	D/D/MP/MP (T4) (n = 38)	Total (N = 184)
**Female (%)**	19 (59%)	21 (55%)	18 (47%)	17 (45%)	20 (53%)	95 (52%)
**Black (%)**	32 (100%)	38 (100%)	38 (100%)	38 (100%)	38 (100%)	184 (100%)
**Age (Median)**	23.0	22.5	22.0	23.0	23.0	23.0
**Age (%)**						
18–20	6 (19%)	11 (29%)	12 (32%)	13 (34%)	11 (29%)	53 (29%)
21–30	23 (72%)	22 (58%)	24 (63%)	23 (61%)	22 (58%)	114 (62%)
31–40	3 (9%)	4 (11%)	2 (5%)	2 (5%)	4 (11%)	15 (8%)
41–50	0 (0%)	1 (3%)	0 (0%)	0 (0%)	1 (3%)	2 (1%)
Number of vaccinations among those that completed follow up*						
Day 0	32 (100%)	38 (100%)	38 (100%)	38 (100%)	38 (100%)	184 (100%)
Day 28	30 (94%)	35 (92%)	37 (97%)	37 (97%)	37 (97%)	176 (96%)
Day 84	30 (94%)	36 (95%)	37 (97%)	34 (89%)	36 (95%)	173 (94%)
Day 168	25 (78%)	36 (95%)	33 (87%)	26 (68%)	26 (68%)	146 (79%)

*19 completed follow up but discontinued vaccinations, 13 completed vaccinations but came off study early, 6 discontinued vaccinations and came off study early. M/M/P/P: MVA prime, sequential gp140 boost; MP/MP: concurrent MVA/gp140; D/D/M/M: DNA prime, sequential MVA boost; D/D/MP/MP: DNA prime, concurrent MVA/gp140 boost).

### Discontinuation of vaccinations

Overall, 146 (79%) participants received all 4 scheduled vaccinations and completed follow up. Vaccinations were discontinued in 25 participants due to: an adverse event (Placebo = 1, MP/MP = 1), pregnancy (MP/MP = 1, D/D/M/M = 1), an unrelated medical condition (D/D/MP/MP = 1), unable to schedule vaccination within study window (D/D/M/M = 1), participant relocated (Placebo = 1, M/M/P/P = 1) and other reasons (Placebo = 3, M/M/P/P = 1, D/D/M/M = 6, D/D/MP/MP = 7).

In 14 of 17 participants that discontinued vaccinations for “other reasons”, the observation of flocculation within MVA vials prompted a decision to terminate MVA administration. Poxvirus vaccine preparations may contain clumps of aggregates on storage and the decision to terminate MVA vaccination was not based on any safety concerns, but reflected an “abundantly cautious” approach.

### Early termination from study

19 participants were terminated from study early due to: HIV infection (MP/MP = 2, D/D/MP/MP = 2), non-adherence to study schedule (M/M/P/P = 1, MP/MP = 2, D/D/M/M = 2, D/D/MP/MP = 2), relocation (placebo = 1, D/D/MP/MP = 1, D/D/M/M = 1), unable to contact (M/M/P/P = 1, D/D/M/M = 2) and “Other reason” (D/D/MP/MP = 2). Of note, one participant was diagnosed with HIV at the final study visit (visit 14) and was therefore not counted as an early termination.

### Safety and tolerability

The vaccines were safe and generally well tolerated.

### Reactogenicity

**Fig 2:**
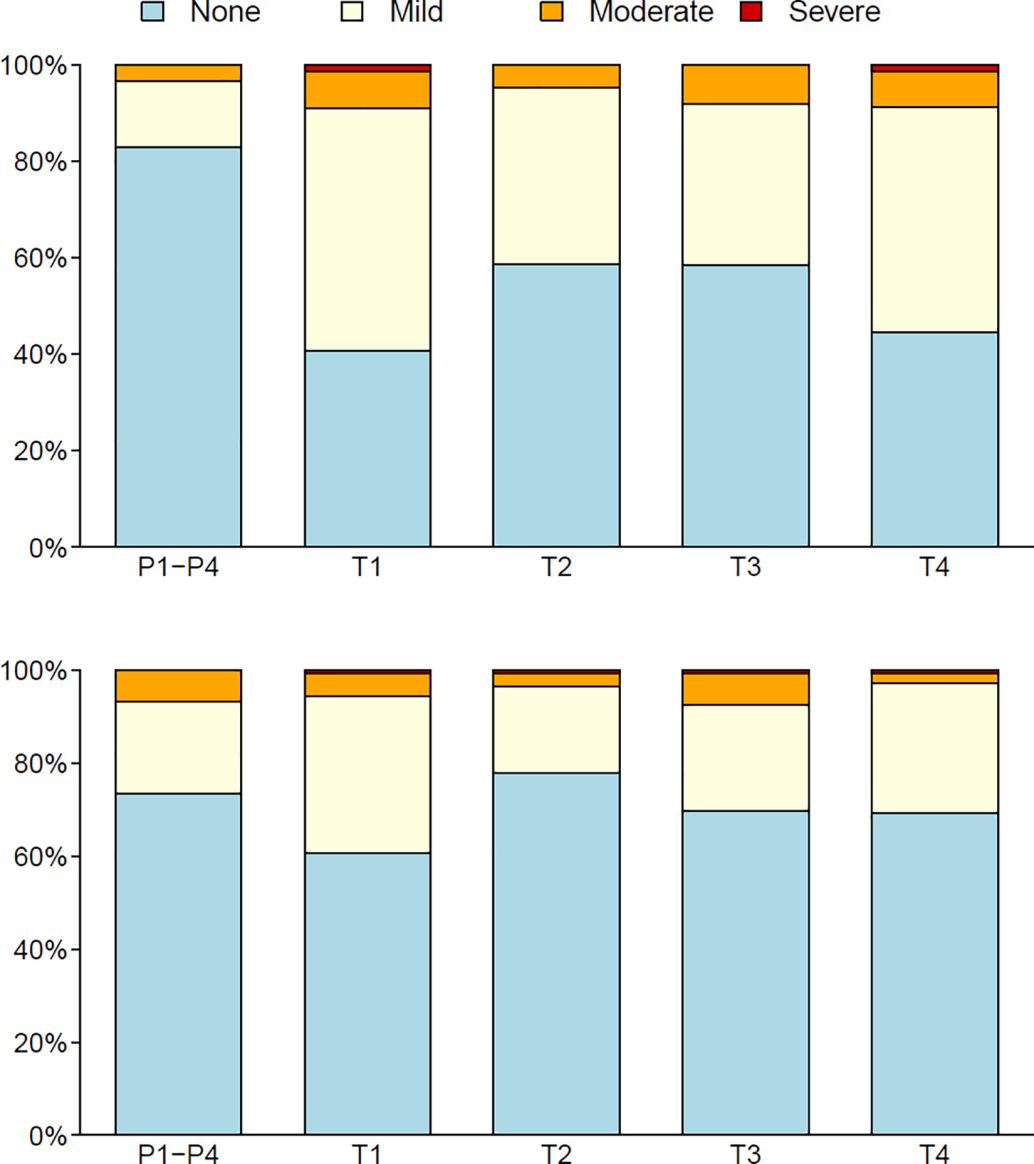
Local and systemic reactinogenicity by treatment group. Local and systemic reactinogenicity for the pooled placebo and each vaccine regimen (T1-T4) are shown in the top and bottom panel respectively. None, mild, moderate and severe reactions are colour coded blue, yellow, orange and red respectively. T1: MVA prime, sequential gp140 boost (M/M/P/P); T2 (MP/MP): concurrent MVA/gp140; T3 (D/D/M/M): DNA prime, sequential MVA boost; T4 (D/D/MP/MP): DNA prime, concurrent MVA/gp140 boost) or placebo. Two-sided unadjusted p-values from Fisher’s exact tests were used to compare proportions of participants that had local or systemic reactions between each pair of vaccine arms.

Most local and systemic reactogenicity was mild to moderate (Fig 2), and, as expected, the proportion of participants that had local or systemic reactions was greater for each vaccine versus the pooled placebo group (p-values < 0.01). Only 4 participants reported severe injection site pain and/or tenderness (2 each in groups M/M/P/P and D/D/MP/MP). Severe systemic symptoms were reported by 4 individuals: one each in groups M/M/P/P (myalgia), MP/MP (headache), D/D/M/M (fever) and D/D/MP/MP (headache). Small, palpable nodules at the injection sites were observed in three participants with onset within 15 days post injection with MVA (MP/MP = 1, D/D/M/M = 1 and D/D/MP/MP = 1). All nodules resolved. The proportion of participants that had local or systemic reactions was similar across vaccine groups (p>0.05) (Fig 2). Similar nodules have been reported in other studies of MVA HIV vaccines.[36]

### Adverse events

A total of 159 participants (placebo 28, M/M/P/P 33, MP/MP 32, D/D/M/M 32, D/D/MP/MP 34) reported at least one adverse event (AE). The maximum severity of these AEs was assessed as mild (85), moderate (62), severe (11) or potentially life-threatening (1). Thirty participants reported one or more AEs assessed as related to study product. Two participants had elevated troponin T levels that were attributed to excess alcohol intake; one of these individuals (in the D/D/MP/MP arm) was diagnosed with alcohol-induced myocarditis. No myocarditis or other diagnosed cardiac adverse events were attributed to MVA vaccination.

Twelve participants had severe and potentially life threatening AEs, which included: unexplained weight loss, neutropenia, right radial neuropathy, acute tonsillitis, headache, hematuria, and pelvic inflammatory disease (none of which were reported as serious adverse events, SAEs) (Table 3).

**Table 3:** Severe or potentially life threatening adverse adverse events.*

	Placebo (P) (n = 32) n (%)	M/M/P/P (T1) (n = 38) n (%)	MP/MP (T2) (n = 38) n (%)	D/D/M/M (T3) (n = 38) n (%)	D/D/MP/MP (T4) (n = 38) n (%)	Total (N = 184) N (%)
**Abnormal weight loss**	1 (3.1%)	0 (0%)	0 (0%)	0 (0%)	2 (5.3%)	**3 (1.6%)**
**Acute tonsilitis**	0 (0%)	0 (0%)	0 (0%)	0 (0%)	1 (2.6%)	**1 (0.5%)**
**Haematuria**	0 (0%)	0 (0%)	1 (2.6%)	0 (0%)	0 (0%)	**1 (0.5%)**
**Decreased haemoglobin**	0 (0%)	0 (0%)	0 (0%)	1 (2.6%)	0 (0%)	**1 (0.5%)**
**Headache**	0 (0%)	0 (0%)	0 (0%)	1 (2.6%)	0 (0%)	**1 (0.5%)**
**Peripheral neuropathy**	0 (0%)	1 (2.6%)	0 (0%)	0 (0%)	0 (0%)	**1 (0.5%)**
**Neutropenia**	0 (0%)	0 (0%)	0 (0%)	0 (0%)	1 (2.6%)	**1 (0.5%)**
**Pelvic inflammatory disease**	0 (0%)	0 (0%)	1 (2.6%)	0 (0%)	0 (0%)	**1 (0.5%)**
**Soft tissue injury**	1 (3.1%)	0 (0%)	0 (0%)	0 (0%)	0 (0%)	**1 (0.5%)**
**Substance-induced psychotic disorder**	0 (0%)	0 (0%)	0 (0%)	0 (0%)	1 (2.6%)	**1 (0.5%)**
**Total**	**2 (6.3%)**	**1 (2.6%)**	**2 (5.3%)**	**2 (5.3%)**	**5 (13.2%)**	**12 (6.5%)**

* All adverse events were graded as severe, apart from 1 potentially life threatening adverse event due to a decreased haemoglobin. There were no fatal events. M/M/P/P: MVA prime, sequential gp140 boost; MP/MP: concurrent MVA/gp140; D/D/M/M: DNA prime, sequential MVA boost; D/D/MP/MP: DNA prime, concurrent MVA/gp140 boost).

Five SAEs were reported during study; all were assessed as not related to study products. Four SAEs were assessed as severe: substance-induced psychosis, pelvic inflammatory disease, soft tissue injuries following a physical assault, and acute follicular tonsillitis. A fifth SAE was a potentially life-threatening decrease in hemoglobin during pregnancy which was successfully treated with transfusions. The psychosis and pelvic inflammatory disease remained unresolved despite treatment.

### HIV infection and vaccine-induced seropositivity

Five participants (4 female, 1 male; M/M/P/P = 1, MP/MP = 2, D/D/MP/MP = 2) acquired HIV infection during study, with time to infection ranging from 3 to 10 months after enrollment. Vaccine-induced seroreactivity for M/M/P/P, MP/MP, D/D/M/M and D/D/MP/MP was 94.6%, 94.4%, 55.3%, 83.3%, respectively

### Pregnancy

Two pregnancies were reported during study (MP/MP = 1, D/D/M/M = 1). For one participant, conception was estimated to have occurred shortly after initial vaccination with MVA. The other participant was pregnant at the time of initial vaccination with DNA vaccine, but had a negative pregnancy test. Each pregnancy resulted in the full term birth of a healthy infant.

### Social impact events

Four social impact events were reported during the study (Placebo = 1, MP/MP = 2, D/D/M/M = 1). Two participants reported an alteration in interpersonal relationships and two participants reported difficulty in receiving appropriate health care (Placebo = 1, MP/MP = 2, D/D/M/M = 1). No events were reported related to housing, employment, insurance or travel.

### Immunogenicity results

#### Neutralizing antibody

**Fig 3:**
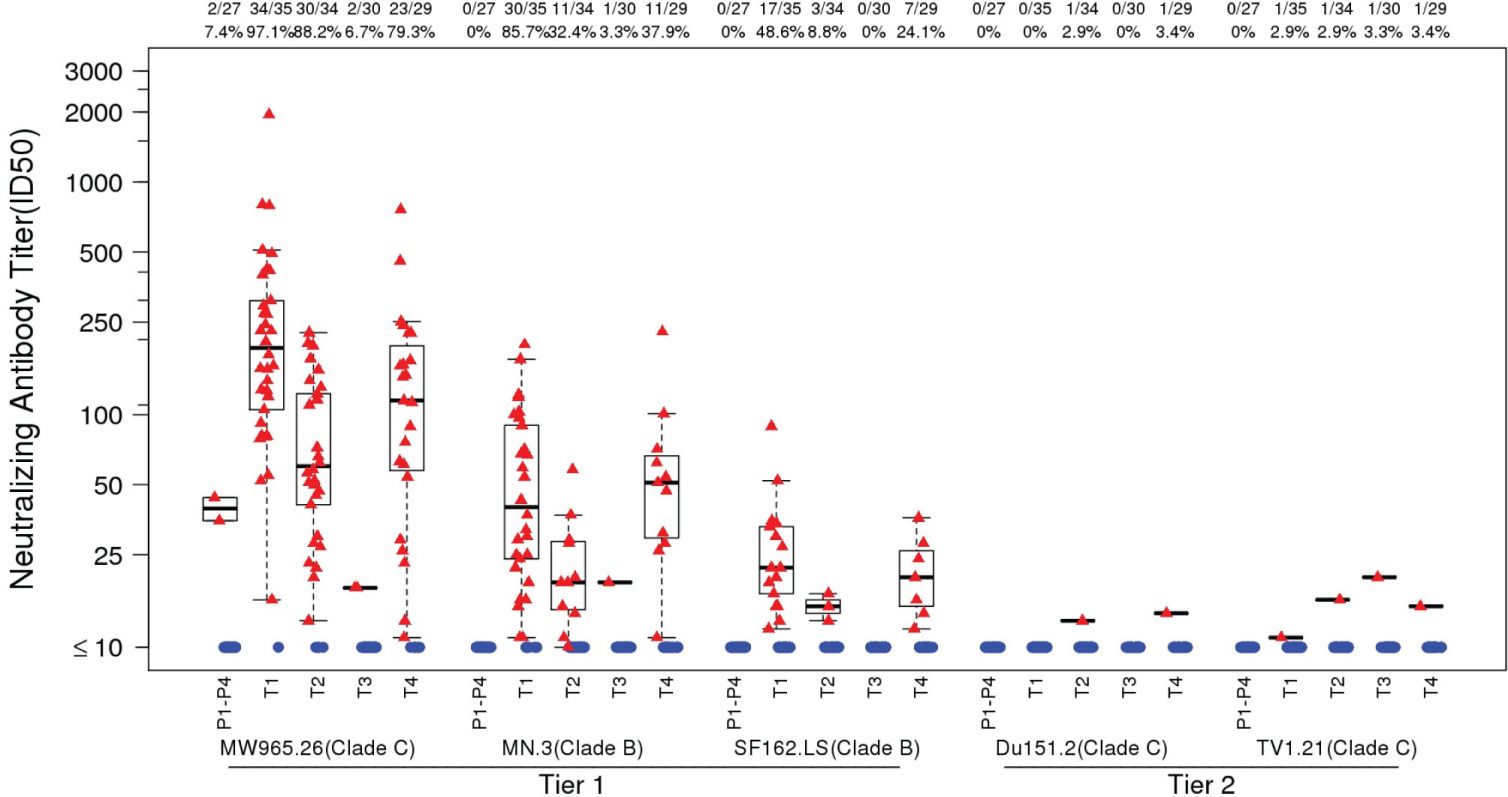
Peak neutralizing antibody titers. TZM-bl neutralizing antibody titers by treatment group at peak immunogenicity (2 weeks post last vaccination) over the panel of 5 virus isolates (Tier 1: Clade B [MN.3, SF162], Clade C [MW925]; Tier 2: Clade C [Du151.2, TV1.21]). Each dot represents an individual, with data from responders in red and non-responders in blue. Box plots based on data from responders only are shown. The mid-line of the box denotes the median and the ends of the box denote the 25th and 75th percentiles. Whiskers extend to the extreme data points that are no more than 1.5 times the interquartile range or if no value meets this criterion, to the data extremes. The number and percent positive responders in each group are shown above the graphs. T1: MVA prime, sequential gp140 boost (M/M/P/P); T2: (MP/MP): concurrent MVA/gp140; T3 (D/D/M/M): DNA prime, sequential MVA boost; T4 (D/D/MP/MP): DNA prime, concurrent MVA/gp140 boost) or placebo.

*Frequency and magnitude of responses (peak time-point)*: The strongest and most frequent neutralizing antibody responses were seen against the Tier 1A viruses MW965.26 (clade C), followed by MN.3 and to a lesser extent, SF162.LS (both clade B) (Fig 3). The highest positive response rates against these viruses were seen in M/M/P/P followed by D/D/MP/MP and MP/MP groups with very few responders in the D/D/M/M group. Differences in these positive response rates were often highly statistically significant (Table 4), apart from MP/MP and D/D/MP/MP which showed no difference in responses to all three Tier 1A viruses. Little or no neutralization was detected against the two clade C vaccine strains (Du151.2 and TV1.21), both of which exhibit a Tier 2 phenotype. In the per protocol analysis, peak neutralizing antibody immune responses were similar to that seen in the intention to treat analysis (data not shown).

**Table 4:** Comparisons of peak neutralizing and binding antibody response rates by treatment group (Fisher's exact test).*

Virus or Antigen	Vaccine regimen P‐value					
	M/M/P/P	M/M/P/P	M/M/P/P	MP/MP	MP/MP	D/D/M/M
	MP/MP	D/D/M/M	D/D/MP/MP	D/D/M/M	D/D/MP/MP	D/D/MP/MP
	(T1 vs T2)	(T1 vs T3)	(T1 vs T4)	(T2 vs T3)	(T2 vs T4)	(T3 vs T4)
**Neutralizing**						
***Tier 1 (Clade C)***						
MW965.26	0.40	<0.001	0.12	<0.001	0.49	<0.001
***Tier 1 (Clade B)***						
MN.3	<0.001	<0.001	0.0004	0.01	0.79	0.003
SF162.LS	0.002	<0.001	0.21	0.33	0.33	0.02
***Tier 2 (Clade C)***						
Du151.2	1.00	NA	1.00	1.00	1.00	1.00
TV1.21	1.00	1.00	1.00	1.00	1.00	1.00
**Binding**						
1086 gp120) (C)	1.00	<0.001	0.05	<0.001	0.16	<0.001
Con 6 gp120/BC	1.00	<0.001	0.05	<0.001	0.16	<0.001
Con S gp140 CFI	1.00	<0.001	0.25	<0.001	0.25	0.01
o‐gpTV1ΔV2 ©	1.00	<0.001	0.60	<0.001	1.00	<0.001

*2-sided P-values are Holm-Bonferroni adjusted (across the 6 pairs of treatment arm comparisons) for each antigen. M/M/P/P: MVA prime, sequential gp140 boost; MP/MP: concurrent MVA/gp140; D/D/M/M: DNA prime, sequential MVA boost; D/D/MP/MP: DNA prime, concurrent MVA/gp140 boost).

**Fig 4:**
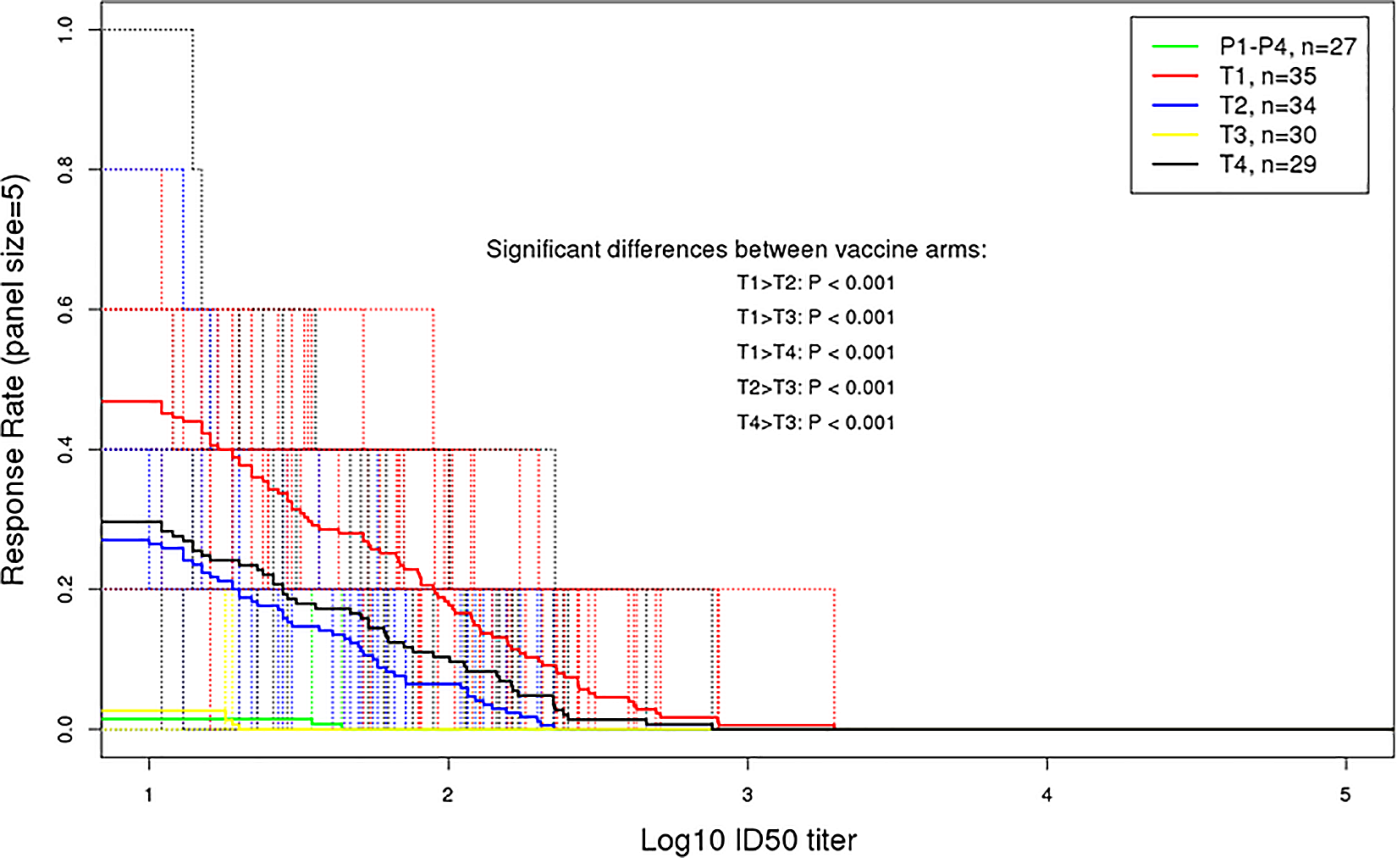
Peak neutralizing antibody–magnitude breadth curves. Average magnitude-breadth (MB) curves in the TZM-bl assay at peak immunogenicity (2 weeks post last vaccination) by treatment group. The area under the curve (AUC)-MB across all subjects in each group was calculated as the average of the log10 neutralizing antibody titers over the panel of 5 virus isolates (Tier 1: Clade B [MN.3, SF162], Clade C [MW965.26]; Tier 2: Clade C [Du151.2, TV1.21]). T1: MVA prime, sequential gp140 boost (M/M/P/P); T2 (MP/MP): concurrent MVA/gp140; T3 (D/D/M/M): DNA prime, sequential MVA boost; T4(D/D/MP/MP): DNA prime, concurrent MVA/gp140 boost) or placebo. Holm-Bonferroni adjusted p-values from unequal-variance t-tests were used for testing different mean AUC-MBs between vaccine arms accounting for the six pairs of vaccine arm comparisons.

*Magnitude breadth curves (peak time-point)*: The peak mean AUC-MBs of neutralization titers to the panel of three Tier 1 (2 clade B, 1 clade C) and two Tier 2 (clade C) vaccine strain pseudoviruses for each vaccine regimen versus the pooled placebo groups (vaccine-placebo) showed that M/M/P/P, MP/MP and D/D/MP/MP had a significantly higher mean AUC-MB than that of the pooled placebo groups (Dunnett p < .0001) (Fig 4). The D/D/M/M vaccine regimen failed to pass this Tier 1 screen (Dunnett p = 0.98). The peak mean AUC-MBs for M/M/P/P was significantly greater than for MP/MP (Holm-Bonferroni adjusted t-test p < .001) and D/D/MP/MP (p = 0.001) (Fig 4). The peak mean AUC-MBs for MP/MP and D/D/MP/MP were similar (p = 0.28). Thus, protein inoculation was required to induce a detectable neutralizing antibody response, where MVA prime followed by sequential protein boost was superior to MVA and protein co-administration. Moreover, DNA priming afforded no significant advantage for MVA and protein co-administration.

**Fig 5:**
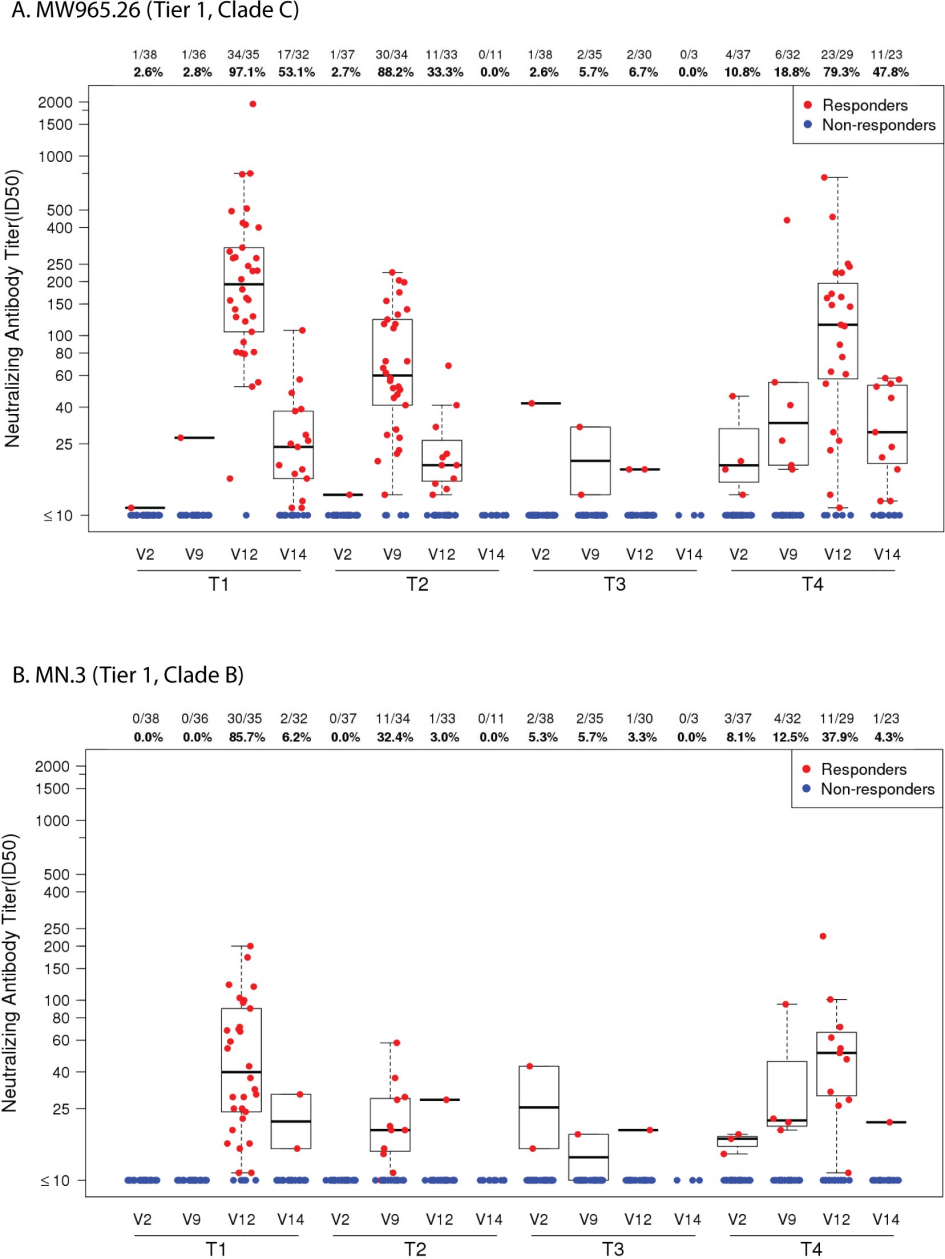
Kinetics of neutralizing antibody responses. TZM-bl neutralizing antibody titers by treatment group at all time-points, including the decay time point (visit 14). Neutralizing antibody titers against the viruses (A). MW965.26 (Clade C; Tier 1) and (B) MN.3 (Clade B; Tier 1), that had the highest responses at the peak immunogenicity time point are shown. Each dot represents an individual, with data from responders in red and non-responders in blue. Box plots based on data from responders only are shown. The number and percent positive responders in each group are shown above the graphs. T1: MVA prime, sequential gp140 boost (M/M/P/P), peak responses at visit 12; T2: concurrent MVA/gp140 (MP/MP), peak responses at visit 9; T3: DNA prime, sequential MVA boost (D/D/M/M), peak responses at visit 12; T4: DNA prime, concurrent MVA/gp140 boost (D/D/MP/MP), peak responses at visit 12. The decay kinetics time point was measured at visit 14, 6 months after last vaccination of M/M/P/P, D/D/P/P and D/D/MP/MP and 9 months after last vaccination of MP/MP.

*Decay kinetics*: Neutralization decay kinetics were assessed against the two most sensitive viruses (MW965.26 (clade C) and MN.3 (clade B) at visit 14, which was approximately 6 months post final boosting for groups M/M/P/P, D/D/M/M, D/D/MP/MP and 9 months post boosting for MP/MP. The response rates against the MW965.26 virus for groups M/M/P/P and D/D/MP/MP declined from 97.1% to 53.1% and 79.3% to 47.8%, respectively and for group MP/MP declined from 88.2% at visit 9, to 33.3% at visit 12 and 0% at visit 14 (Fig 5A). Among positive responders in groups M/M/P/P and D/D/MP/MP, the peak titers declined significantly by visit 14 (Wilcoxon signed-rank test p ≤ 0.001) (Fig 5A). There was no significant differences in the decline rate between M/M/P/P and D/D/MP/MP (p = 0.46). The results were similar for responses against the MN.3 virus (Fig 5B), except the magnitudes of responses were lower and the positive response rates for groups M/M/P/P and D/D/MP/MP declined from visit 12 to 14 from 85.7% to 6.2% and 37.9% to 4.3%, respectively. For the MP/MP group the response rates declined from 32.4% at visit 9 to 3.0% at visit 12 and 0% at visit 14. For the D/D/M/M group response rates to both viruses were minimal at peak and 0% at visit 14.

#### HIV-specific binding antibodies

**Fig 6:**
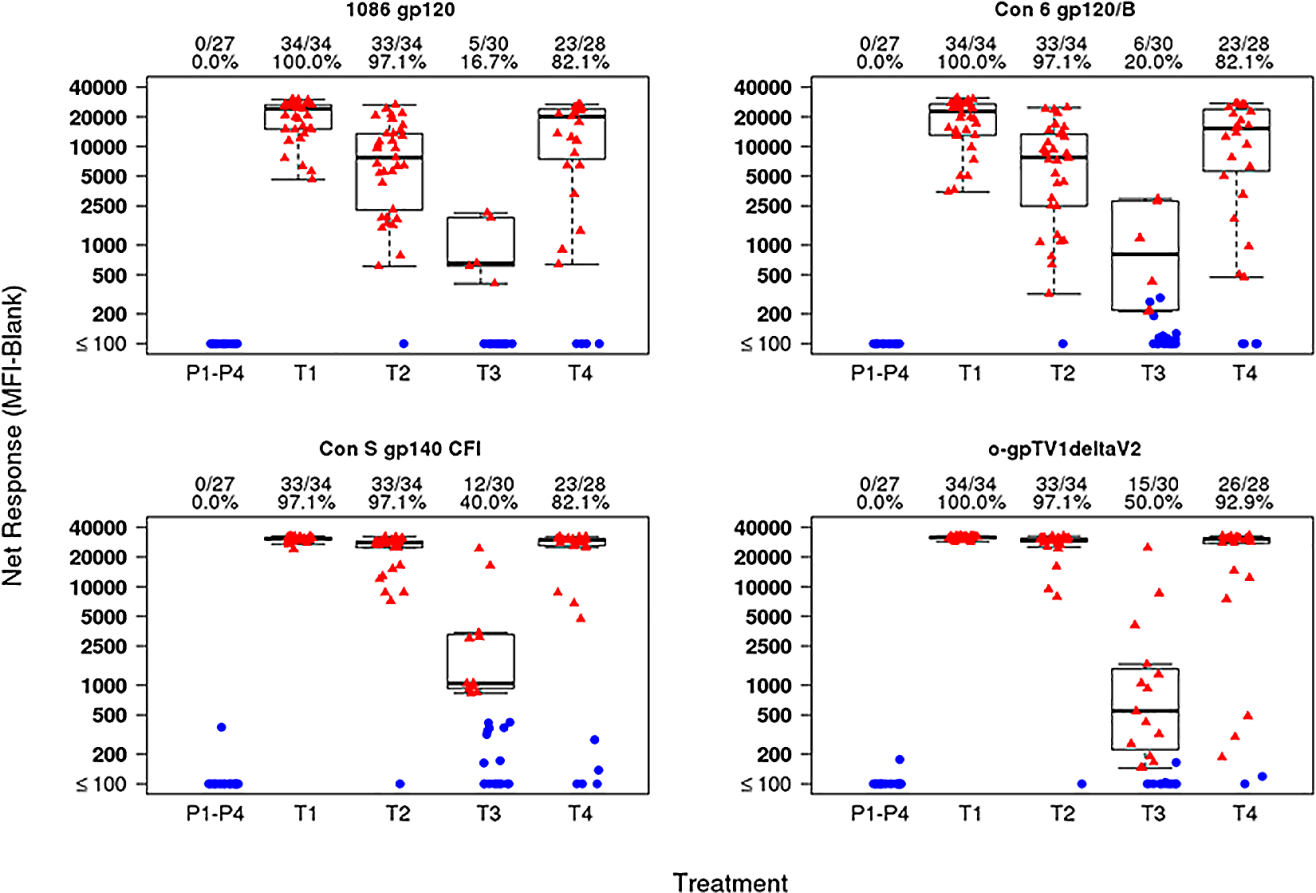
Peak IgG binding antibody response. Binding magnitude of IgG responses to gp120 and gp140 antigens are shown as mean fluorescent intensity (MFI) in top, middle and lower panels, respectively. Positive responders are indicated in red and negative responders in blue. The mid-line of the box denotes the median and the ends of the box denote the 25th and 75th percentiles. The whiskers that extend from the top and bottom of the box extend to the most extreme data points that are no more than 1.5 times the interquartile range (i.e., height of the box) or if no value meets this criterion, to the data extremes. The number and percent positive responders in each group are shown above the graphs. Placebo recipients from all treatment groups are shown together. T1: MVA prime, sequential gp140 boost (M/M/P/P); T2 (MP/MP): concurrent MVA/gp140; T3 (D/D/M/M): DNA prime, sequential MVA boost; T4 (D/D/MP/MP): DNA prime, concurrent MVA/gp140 boost) or placebo.

**Fig 7:**
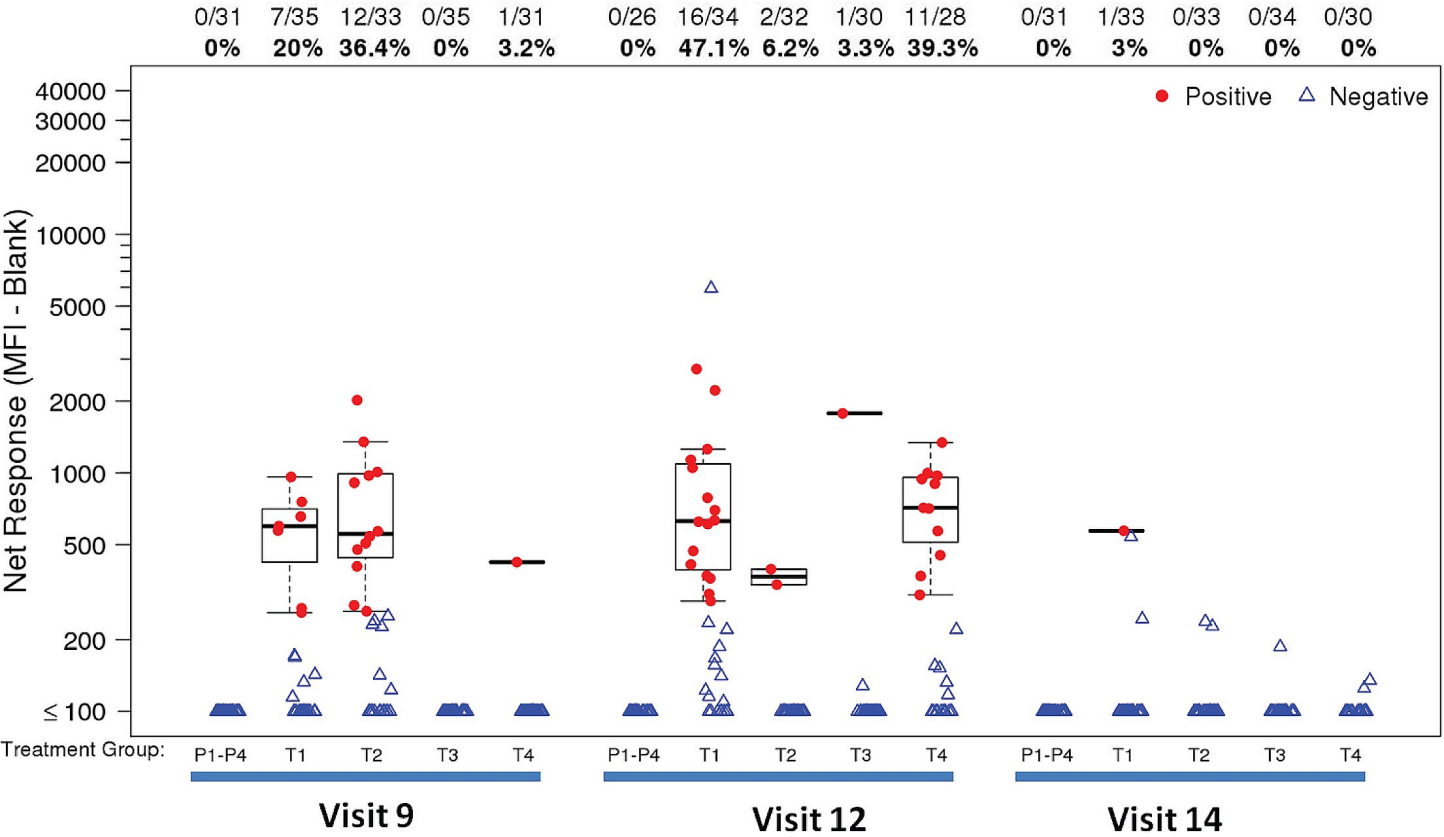
Kinetics of IgA binding antibody responses to Con S gp140 CFI. Binding magnitude of IgA responses to Con S gp140 CFI is shown as mean fluorescent intensity (MFI) in top, middle and lower panels, respectively. Positive responders are indicated in red and negative responders in blue. The mid-line of the box denotes the median and the ends of the box denote the 25th and 75th percentiles. The whiskers that extend from the top and bottom of the box extend to the most extreme data points that are no more than 1.5 times the interquartile range (i.e., height of the box) or if no value meets this criterion, to the data extremes. The number and percent positive responders in each group are shown above the graphs. Placebo recipients from all treatment groups are shown together. T1: MVA prime, sequential gp140 boost (M/M/P/P); T2 (MP/MP): concurrent MVA/gp140; T3 (D/D/M/M): DNA prime, sequential MVA boost; T4 (D/D/MP/MP): DNA prime, concurrent MVA/gp140 boost) or placebo.

*Peak time-point*: At the peak time point, the IgG response rates to HIV-1 gp120 antigens (1086 gp120, Con 6 gp120/B), and gp140 antigens (Con S gp140 CFI, o-gpTV1ΔV2) were high (>80%) in M/M/P/P, MP/MP and D/D/MP/MP and minimal (≤20%) in D/D/M/M (Fig 6). Furthermore, the magnitudes of response to all gp120 and gp140 antigens were high in M/M/P/P, MP/MP and D/D/MP/MP groups and low in the D/D/M/M group (Fig 6). The p-values for the pairwise comparisons of peak binding antibody responses between vaccine groups are shown in Table 4, with several significant differences. For both gp120 antigens, responses were higher in the M/M/P/P group compared to the D/D/MP/MP group. In the per protocol analysis, peak binding antibody immune responses were similar to that seen in the intention to treat analysis (data not shown). The greatest frequency and magnitude of IgA binding antibody responses, at the peak immunogenicity time point (visit 12 for M/M/P/P and D/D/MP/MP; visit 9 for MP/MP), were observed for Con S gp140 CFI (M/M/P/P: 47.1%; D/D/MP/MP: 39.3%; MP/MP: 36.4%) (Fig 7), gp140C_avi (M/M/P/P: 44.1%; D/D/MP/MP: 32.1%; MP/MP: 18.2%) and gp41 ((M/M/P/P: 20.6%; D/D/MP/MP: 32.1%; MP/MP: 27.3%). There were no IgA responses to V1V2 antigens (data not shown).

**Fig 8:**
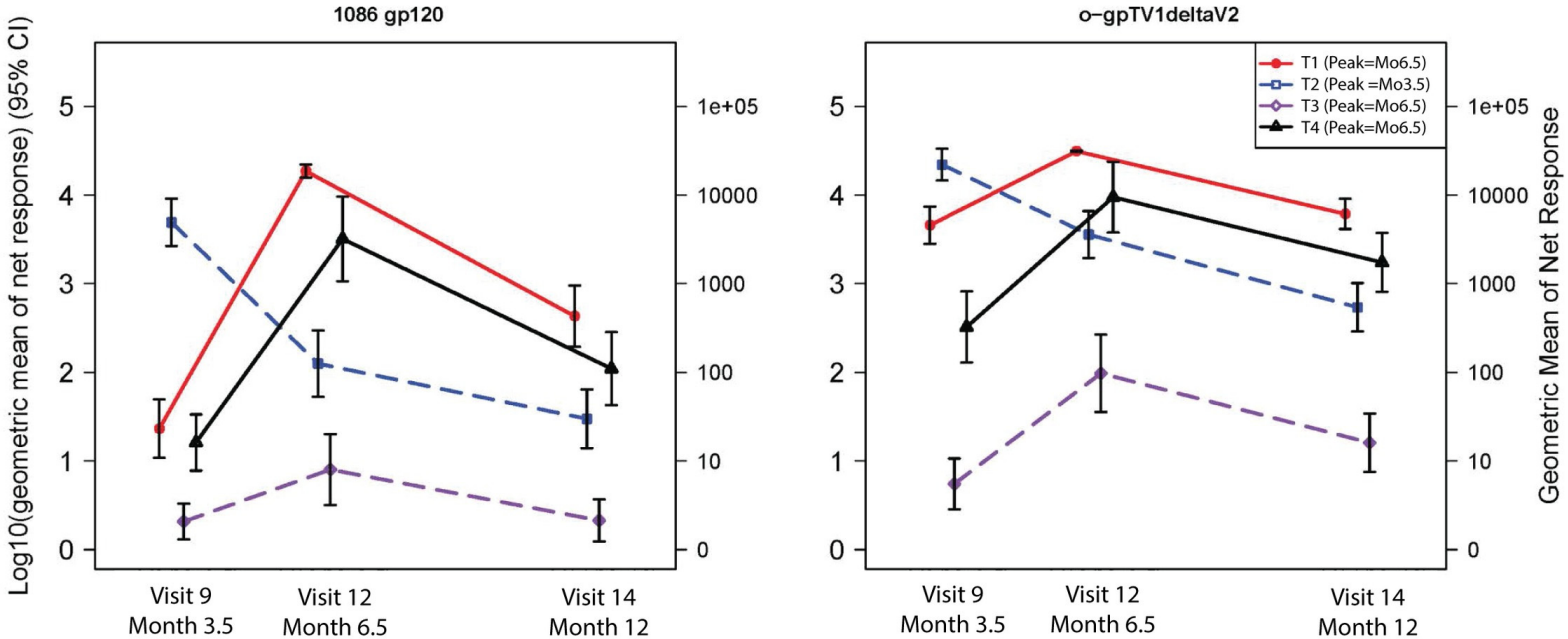
Kinetics of IgG binding antibody responses. The geometric mean (and 95% error bars) of net responders are shown for visit 9, 12 and 14. T1: MVA prime, sequential gp140 boost (M/M/P/P), peak responses at visit 12; T2: concurrent MVA/gp140 (MP/MP), peak responses at visit 9; T3: DNA prime, sequential MVA boost (D/D/M/M), peak responses at visit 12; T4: DNA prime, concurrent MVA/gp140 boost (D/D/MP/MP), peak responses at visit 12. The decay kinetics time point was measured at visit 14, 6 months after last vaccination of M/M/P/P, D/D/P/P and D/D/MP/MP and 9 months after last vaccination of MP/MP.

*Decay kinetics*: Protein boosting in groups M/M/P/P, MP/MP and D/D/MP/MP induced strong IgG binding antibody responses to 1086 gp120 and o−gpTV1deltaV2 at peak, which declined to modest levels by visit 14, with the greatest decline observed in the MP/MP group (Fig 8). The frequency of IgA binding antibody responses to Con S gp140 CFI decayed from the peak responses (see section on binding antibodies at peak timepoints) to being absent or minimal at visit 14 (5.5 months after peak responses for M/M/P/P and D/D/MP/MP; 8.5 months after peak responses for MP/MP) for all vaccination groups (Fig 7).

#### CD4+ and CD8+ T-cell responses (peak time-point)

**Fig 9:**
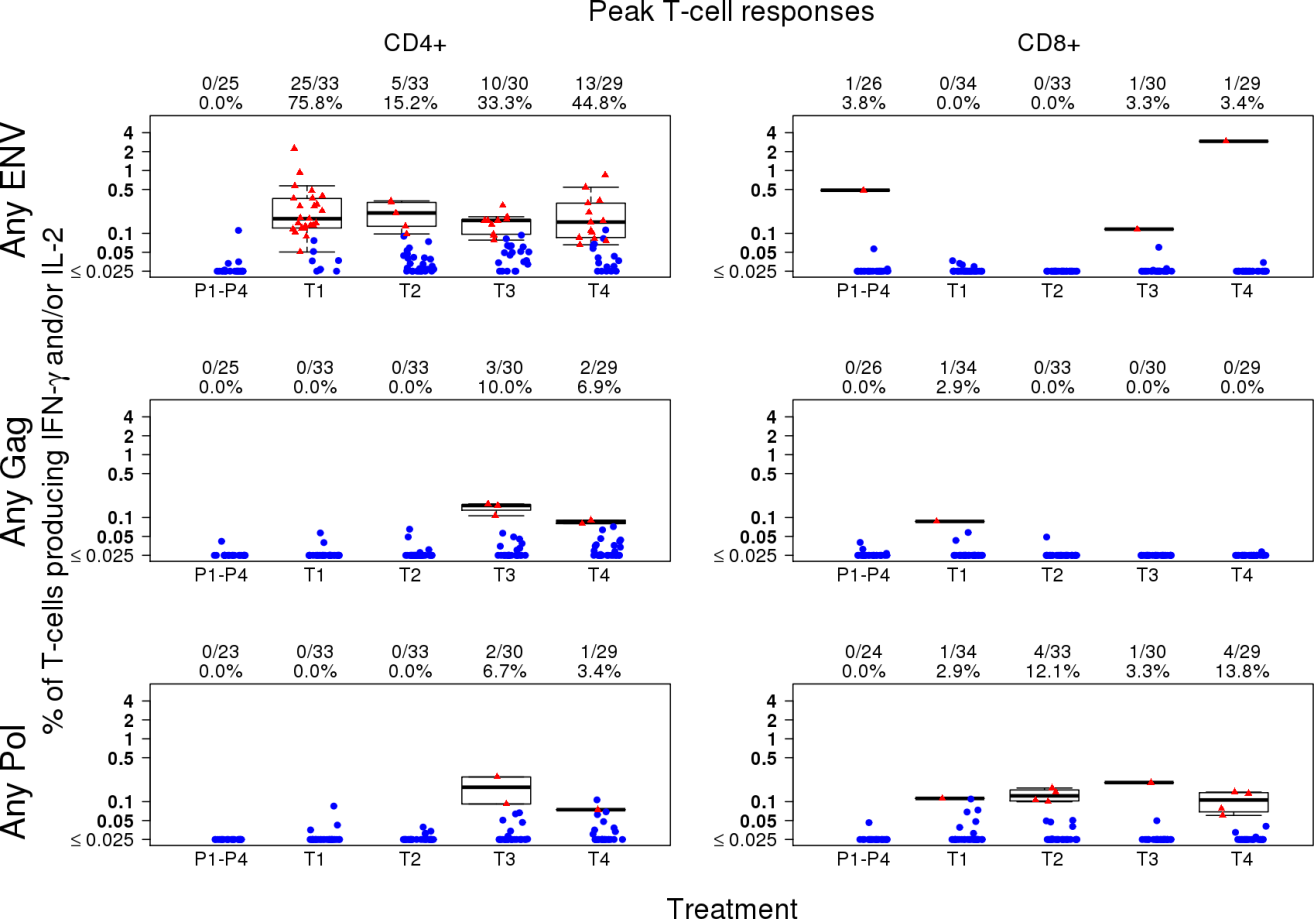
Peak T-cell responses to vaccine antigens. CD4+ and CD8+ background-adjusted T-cell responses as determined by intracellular cytokine staining 2 weeks following the fourth vaccination in groups 1, 3 and 4 (month 6.5) and following the second vaccination in group 2 (month 3.5). Because of overlap of peptides between PTE_g_ peptide pools for the same HIV protein, the magnitude for the protein is calculated as the maximum among the peptide pools for the protein. The overall magnitude is calculated as the sum of the individual HIV protein magnitudes. Plots include data from responders in red and non-responders in blue. Box plots based upon data from responders only are superimposed on the distributions. The mid-line of the box denotes the median and the ends of the box denote the 25th and 75th percentiles. The whiskers that extend from the top and bottom of the box extend to the most extreme data points that are no more than 1.5 times the interquartile range (i.e., height of the box) or if no value meets this criterion, to the data extremes. The number and percent positive responders in each group are shown above the graphs. Placebo recipients from all treatment groups are shown together. T1 (M/M/P/P): MVA prime, sequential gp140 boost; T2 (MP/MP): concurrent MVA/gp140; T3 (D/D/M/M): DNA prime, sequential MVA boost; T4 (D/D/MP/MP): DNA prime, concurrent MVA/gp140 boost) or placebo.

For CD4+ T cells producing IFN-γ and/or IL-2, the highest response rate at the peak time point for any Env PTE_g_ was observed in M/M/P/P (75.8%), followed by D/D/MP/MP (44.8%), D/D/M/M (33.3%), and MP/MP (15.2%) (Fig 9). The magnitude of CD4+ T cell responses for any Env PTE_g_ was similar across the treatment groups (Fig 9). At the peak time point D/D/M/M induced modest and D/D/MP/MP induced minimal CD4+ T cell responses to any Gag and any Pol while no responses were seen in the D/D/P/P and MP/MP groups (Fig 9). None of the vaccine regimens induced responses to any Nef peptide pools (data not shown). The comparisons of peak CD4+ and CD8+ T cell response by treatment group (Fisher's exact test) are shown in Table 5.

**Table 5:** Comparisons of peak CD4+ and CD8+ T cell response by treatment group (Fisher's exact test).*

T cell	Peptide Pool	M/M/P/P	M/M/P/P	M/M/P/P	MP/MP	MP/MP	D/D/M
		MP/MP	D/D/M	D/D/MP/MP	D/D/M	D/D/MP/MP	D/D/MP/MP
**CD4+**	ANY ENV PTEG	0.60	0.97	1.00	0.047	0.13	1.00
	ANY GAG PTEG	1.00	0.04	0.02	0.011	0.004	1.00
	ANY POL PTEG	1.00	0.59	0.12	0.21	0.025	1.00
**CD8+**	ANY ENV PTEG	N/A	1.00	N/A	1.00	N/A	1.00
	ANY NEF PTEG	1.00	1.00	1.00	1.00	1.00	1.00
	ANY POL PTEG	1.00	0.32	1.00	0.99	1.00	1.00

*2-sided P-values are Holm-Bonferroni adjusted (across the 6 pairs of treatment arm comparisons) for each antigen. M/M/P/P: MVA prime, sequential gp140 boost; MP/MP: concurrent MVA/gp140; D/D/M/M: DNA prime, sequential MVA boost; D/D/MP/MP: DNA prime, concurrent MVA/gp140 boost).

For CD8+ T cells producing IFN-γ and/or IL-2, the greatest response rate at the peak time point was to any Pol PTE_g_ (D/D/MP/MP (13.8%), MP/MP (12.1%), D/D/M/M (3.3%) and M/M/P/P (2.9%)) (Fig 9). The CD8+ T cell response rate for any Env or any Gag was minimal or absent and there were no responses to any Nef (data not shown) (Fig 9).

#### Polyfunctionality data (peak time-point)

**Fig 10:**
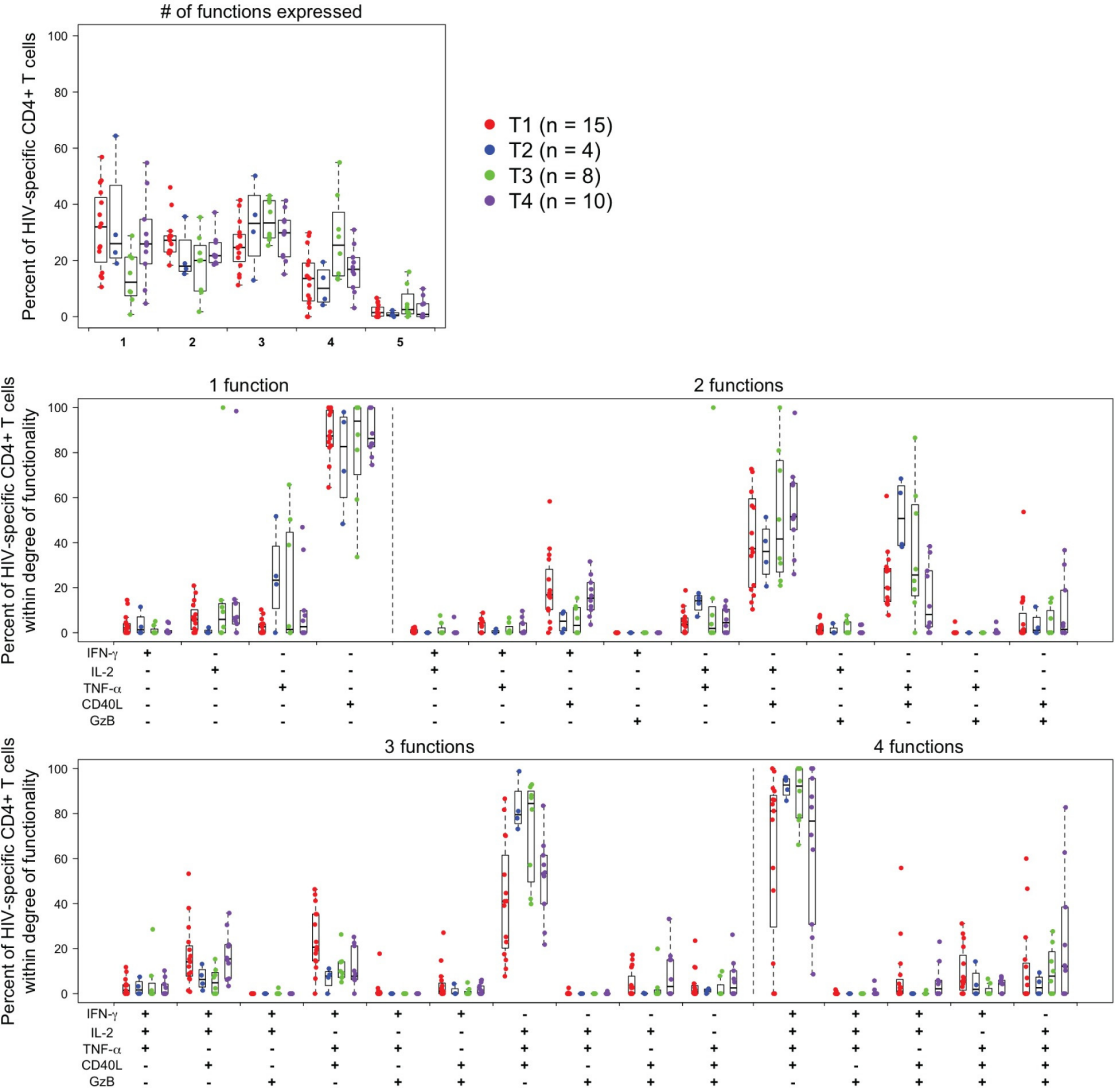
Polyfunctionality analysis of peak CD4+ T-cell Env-specific responses. Only responses positive for IFN-γ and/or IL-2 are shown; data are for the Env pool with the largest response within each individual at the peak timepoint for each treatment group. The upper left graph shows the proportion of Env-specific cells expressing the indicated number of functions. Total Env-specific cells are determined as cells producing any of the 5 functions alone or in combination, except that granzyme B must be co-expressed with another function since it is constitutively expressed. The next graphs show the proportion of cells within each degree of functionality expressing the indication combinations of functions. T1 (M/M/P/P): MVA prime, sequential gp140 boost; T2 (MP/MP): concurrent MVA/gp140; T3 (D/D/M/M): DNA prime, sequential MVA boost; T4 (D/D/MP/MP): DNA prime, concurrent MVA/gp140 boost) or placebo.

Peak CD4+ T-cell responses to any Env PTE_g_ by number of functions and among cells expressing 1, 2, 3, 4 or 5 functions are shown in Fig 10. For CD4 T-cells, responding cells are relatively evenly divided between cells producing 1, 2, or 3 functions, with a smaller proportion producing 4 functions and virtually none producing 5 functions. CD40L is the dominant single function. The IL-2/CD40L and TNF-α/CD40L combinations are the dominant dual functions. The combined IL-2/TNF-α /CD40L is the dominant triple function, with smaller proportions of cells co-producing IFN-γ with CD40L along with either IL-2 or TNF-α. For 4 functions, IFN-γ/IL-2/TNF-α /CD40L is dominant. There are only relatively minor differences between treatment arms, and differences were not consistent. Because of the low response rate for CD8+ T-cells and some variability in the profiles, it was not possible to determine CD8+ T-cell polyfunctionality profiles.

## Discussion

The SAAVI DNA-C2, SAAVI MVA-C and Novartis TV1gp140ΔV2 with MF59 adjuvant in various vaccination schedules in HIV-uninfected healthy vaccinia-naïve adult participants in South Africa were safe and induced humoral and cellular immune responses. The immune responses however differed by vaccine regimen. These results are likely to be generalizable to HIV-uninfected adults living in sub-Saharan Africa.

### Immunogenicity: summary of HVTN 086/SAAVI 103 responses

The best neutralizing antibody responses were produced by the sequential protein boost regimens (M/M/P/P, D/D/MP/MP). Strong binding antibody responses to Env were produced by all vaccine regimens containing a protein boost (M/M/P/P, MP/MP, D/D/MP/MP). The CD4+ T-cell responses were greatest to Env with the sequential protein boost regimens (M/M/P/P, D/D/MP/MP). CD4+ T-cell responses to Gag and Pol were modest for the DNA prime/ MVA boost regimen (D/D/M/M). CD4+ T-cell responses to Nef were absent for all vaccine regimens. CD8+ T-cell responses to any antigen were low for all regimens. These results confirm the importance of sequential protein boosting to achieve maximum antibody titers and demonstrate that the MVA vector was superior to the DNA vector for priming Env-specific binding and neutralizing antibody responses. We note that the neutralizing activity was limited to viruses that exhibit a highly sensitive Tier 1 neutralization phenotype and was not seen with viruses that exhibit a less sensitive Tier 2 phenotype that is more typical of circulating strains. Furthermore, neutralizing antibody responses waned even with high prevalence and magnitude of CD4+ T-cell responses to HIV Env.

### Sequential versus concomitant administration of protein

The RV144 regimen used a strategy of ALVAC prime, and combination ALVAC: protein boost, which induced binding antibody responses to gp120 in >98% of participants at 6.5 months after the last vaccination.[12] In addition, the RV144 follow on study (RV305) showed that a protein boost administered 6–8 years later, with or without ALVAC, induced strong IgG binding antibody responses.[37] In a phase 1 trial (HVTN 073/SAAVI 102) using the same DNA and MVA used in this trial, the DNA prime (3 doses)/MVA boost (2 doses) did not induce any neutralizing antibodies and induced low levels of binding antibodies. In a follow on study (HVTN 073E/SAAVI 102E), the same protein used in our study was administered approximately two years after the last MVA vaccine, which induced large binding and neutralizing antibody responses.[38] In our study, protein given sequentially with an MVA prime induced stronger peak neutralizing and binding antibody responses than if given concomitantly with MVA, and decayed more slowly. Including protein in a DNA or poxvirus vector based vaccine regimen is important for inducing strong neutralizing and binding antibody and CD4 T-cell responses. Administering protein sequentially as a boost to induce greater frequency and magnitude of neutralizing antibody immune responses may be preferable to administering protein concurrently with the MVA prime in order to induce earlier, but lower frequency and magnitude of peak neutralizing antibody responses.

### DNA prime

DNA prime, protein boost regimens have been shown to induce better and more durable humoral and cellular immune responses, in pre-clinical studies and in human trials, than either DNA or protein alone.[39–44]. Furthermore, clinical trials evaluating DNA prime, vaccinia virus based vector (NYVAC or MVA) boost regimens showed that DNA is effective in priming MVA and NYVAC induced responses.[45–49] The safety and immunogenicity of the SAAVI DNA-C2 prime/MVA-C boost regimen was evaluated in a phase 1 trial in the United States of America and South Africa (HVTN 073/SAAVI 102).[6;11] The SAAVI DNA-C2/MVA-C prime/boost regimen induced: strong peak CD4+ T-cell response rates, largely in response to Env (67% overall), which were stronger in US versus South African participants (75% and 64%, respectively), and about a third expressed three cytokines; modest CD8+ T-cell response rates (33%), largely induced by Pol; no neutralizing antibody responses; and strong binding antibody responses to gp140 and gp120.[5;11] In our trial the DNA prime with the MVA boost alone induced low levels of binding and neutralizing immune responses and modest CD4 T cell responses to any Env, Gag or Pol, whereas the DNA prime with a concomitant MVA/protein boost resulted in comparable binding and neutralizing and cellular responses to the MP/MP regimen. The DNA prime in our trial therefore does not appear to substantially contribute to humoral immune responses.

### MVA prime

MVA is the most common vaccinia vector used in HIV vaccine trials.[50] MVAs containing HIV gene inserts are safe and immunogenic when given alone and induce polyfunctional, durable CD4+ and CD8+ T cell responses and binding antibody responses in the vast majority of participants.[51–54] In a phase 1 trial, two doses of MVA/HIV62 induced CD4+ and CD8+ T cell responses in 43% and 17% of participants respectively.[53] In another phase 1 trial three doses of an MVA with HIV clade B antigens induced T-cell ELISpot, binding and neutralizing antibody responses in 75%, 95% and 33% of participants respectively. In a phase 1 trial, immune responses induced by an MVA prime were boosted by a heterologous pox vector (Fowlpox).[54] In our trial the MVA prime induced minimal binding and neutralizing responses (to Tier 1 clade B and C viruses), which were boosted substantially by the protein. The MVA prime/protein boost (M/M/P/P) regimen appears to induce more frequent polyfunctional CD4+ T-cell responses (76.5% of participants). The MVA prime in our trial therefore did contribute to humoral and cellular immune responses.

### Comparison with RV144

The RV144 correlates of HIV-1 risk demonstrated that IgG binding antibodies to V1V2 Env were inversely correlated with infection risk whereas specific plasma IgA binding antibodies to Env directly correlated with HIV infection risk. [13;27;55;56] Thus, a hypothesis is that vaccines that induce higher levels of V1V2 antibodies and lower levels of Env-specific IgA antibodies than that induced by the RV144 regimen may be more efficacious in preventing HIV infection. A limitation of this study was that the hypervariable region (V2 loop) of the TV-1 HIV-1 envelope glycoprotein 120 (TV1gp140ΔV2) was deleted in order to expose conserved regions of the Env involved in viral entry and increase susceptibility to virus neutralization.[22] This rationale was supported by the induction of neutralising antibody responses when used in DNA primed rabbits and macaques.[22] As V2 was deleted from the gp140 protein boost, we did not expect to detect strong V1V2 binding antibody responses. The low level V1V2 binding antibody responses observed (data not shown) were probably induced by the V1V2 contained within the DNA-C2 and MVA-C vaccines. In our trial minimal to moderate IgA responses that were not durable were observed. Further studies using the MVA prime vaccine utilized in this study in combination with a subsequent sequential boost of a recombinant protein containing the V1/V2 region of gp120 would be warranted, especially if V1V2 and IgA responses to gp120 continue to emerge as important correlates of vaccine efficacy in future vaccine trials.

## Conclusion

Sequential protein boosting induced strong peak neutralizing and binding antibody responses that decayed with time. The MVA/protein prime/boost regimen induced the strongest neutralising antibody and T cell responses. The DNA prime did not contribute substantially to the immune responses. A SAAVI MVA-C based regimen with a protein (likely including the V2 loop) boost should be evaluated in further clinical trials leading to potential efficacy studies.

## Supporting Information

S1 Checklist HVTN 086/SAAVI 103 CONSORT Checklist. (DOC)

S1 Protocol HVTN 086/SAAVI 103 Protocol, Version 2. (PDF)

S2 Protocol HVTN 086/SAAVI 103 Protocol, Version 3. (PDF)

## References

[1] Joint United Nations Programme on HIV/AIDS (UNAIDS). GLOBAL REPORT: UNAIDS report on the global AIDS epidemic 2013. 2013. Report No.: UNAIDS / JC2502/1/E.

[2] Shisana OR, Rehele T, Simbayi LC, Zuma K, Jooste S, Zungu, et al South African National HIV Prevalence, Incidence and Behaviour Survey, 2012 Human Sciences Research Council Press, Cape Town; 2014. 10.2989/16085906.2016.1153491 27002359

[3] Bor J, Herbst AJ, Newell ML, Barnighausen T. Increases in adult life expectancy in rural South Africa: valuing the scale-up of HIV treatment. Science 2013;339:961–5. 10.1126/science.1230413 23430655 PMC3860268

[4] Tanser F, Barnighausen T, Grapsa E, Zaidi J, Newell ML. High coverage of ART associated with decline in risk of HIV acquisition in rural KwaZulu-Natal, South Africa. Science 2013;339:966–71. 10.1126/science.1228160 23430656 PMC4255272

[5] Williamson AL, Rybiki E, Shephard E, Gray G, Bekker LG, Downing K, et al South African HIV-1 vaccine candidates—the journey from the bench to clinical trials. S Afr Med J 2012;102:452–5. 22668934 10.7196/samj.5668

[6] Williamson C, Morris L, Maughan MF, Ping LH, Dryga SA, Thomas R, et al Characterization and selection of HIV-1 subtype C isolates for use in vaccine development. AIDS Res Hum Retroviruses 2003;19:133–44. 12639249 10.1089/088922203762688649

[7] Burgers WA, van Harmelen JH, Shephard E, Adams C, Mgwebi T, Bourn W, et al Design and preclinical evaluation of a multigene human immunodeficiency virus type 1 subtype C DNA vaccine for clinical trial. J Gen Virol 2006;87:399–410. 16432028 10.1099/vir.0.81379-0

[8] Burgers WA, Shephard E, Monroe JE, Greenhalgh T, Binder A, Hurter E, et al Construction, characterization, and immunogenicity of a multigene modified vaccinia Ankara (MVA) vaccine based on HIV type 1 subtype C. AIDS Res Hum Retroviruses 2008;24:195–206. 10.1089/aid.2007.0205 18240957

[9] Shephard E, Burgers WA, van Harmelen JH, Monroe JE, Greenhalgh T, Williamson C, et al A multigene HIV type 1 subtype C modified vaccinia Ankara (MVA) vaccine efficiently boosts immune responses to a DNA vaccine in mice. AIDS Res Hum Retroviruses 2008;24:207–17. 10.1089/aid.2007.0206 18240963

[10] Burgers WA, Chege GK, Muller TL, van Harmelen JH, Khoury G, Shephard EG, et al Broad, high-magnitude and multifunctional CD4+ and CD8+ T-cell responses elicited by a DNA and modified vaccinia Ankara vaccine containing human immunodeficiency virus type 1 subtype C genes in baboons. J Gen Virol 2009;90:468–80. 10.1099/vir.0.004614-0 19141458

[11] Gray G, Elizaga M, Bekker LG. Immunogenicity of a subtype C HIV vaccine regimen, the SAAVI DNA-C2 vaccine boosted by SAAVI MVA-C vaccine: Results of a Phase I study conducted in South Africa and USA amongst HIV uninfected adults (HVTN 073/SAAVI 102). Conference on Retroviruses and Opportunistic Infections (CROI). 2011.

[12] Rerks-Ngarm S, Pitisuttithum P, Nitayaphan S, Kaewkungwal J, Chiu J, Paris R, et al Vaccination with ALVAC and AIDSVAX to prevent HIV-1 infection in Thailand. N Engl J Med 2009;361:2209–20. 10.1056/NEJMoa0908492 19843557

[13] Haynes BF, Gilbert PB, McElrath MJ, Zolla-Pazner S, Tomaras GD, Alam SM, et al Immune-correlates analysis of an HIV-1 vaccine efficacy trial. N Engl J Med 2012;366:1275–86. 10.1056/NEJMoa1113425 22475592 PMC3371689

[14] Buchbinder SP, Mehrotra DV, Duerr A, Fitzgerald DW, Mogg R, Li D, et al Efficacy assessment of a cell-mediated immunity HIV-1 vaccine (the Step Study): a double-blind, randomised, placebo-controlled, test-of-concept trial. Lancet 2008;372:1881–93. 10.1016/S0140-6736(08)61591-3 19012954 PMC2721012

[15] Hammer SM, Sobieszczyk ME, Janes H, Karuna ST, Mulligan MJ, Grove D, et al Efficacy trial of a DNA/rAd5 HIV-1 preventive vaccine. N Engl J Med 2013;369:2083–92. 10.1056/NEJMoa1310566 24099601 PMC4030634

[16] Gray GE, Allen M, Moodie Z, Churchyard G, Bekker LG, Nchabeleng M, et al Safety and efficacy of the HVTN 503/Phambili study of a clade-B-based HIV-1 vaccine in South Africa: a double-blind, randomised, placebo-controlled test-of-concept phase 2b study. Lancet Infect Dis 2011;11:507–15. 10.1016/S1473-3099(11)70098-6 21570355 PMC3417349

[17] Lin L, Finak G, Ushey K, Seshadri C, Hawn TR, Frahm N, et al COMPASS identifies T-cell subsets correlated with clinical outcomes. Nat Biotechnol 2015;33:610–6. 10.1038/nbt.3187 26006008 PMC4569006

[18] Cassimatis DC, Atwood JE, Engler RM, Linz PE, Grabenstein JD, Vernalis MN. Smallpox vaccination and myopericarditis: a clinical review. J Am Coll Cardiol 2004;43:1503–10. 15120802 10.1016/j.jacc.2003.11.053

[19] Eckart RE, Love SS, Atwood JE, Arness MK, Cassimatis DC, Campbell CL, et al Incidence and follow-up of inflammatory cardiac complications after smallpox vaccination. J Am Coll Cardiol 2004;44:201–5. 15234435 10.1016/j.jacc.2004.05.004

[20] Halsell JS, Riddle JR, Atwood JE, Gardner P, Shope R, Poland GA, et al Myopericarditis following smallpox vaccination among vaccinia-naive US military personnel. JAMA 2003;289:3283–9. 12824210 10.1001/jama.289.24.3283

[21] Elizaga M, Vasan S, Marovich M, Sato A, Lawrence D, Chaitman B, et al Prospective surveillance for cardiac adverse events in healthy adults receiving modified vaccinia Ankara vaccines: a systematic review. PLoS One 2013;8: e54407 10.1371/journal.pone.0054407 23349878 PMC3547923

[22] Lian Y, Srivastava I, Gomez-Roman VR, Zur MJ, Sun Y, Kan E, et al Evaluation of envelope vaccines derived from the South African subtype C human immunodeficiency virus type 1 TV1 strain. J Virol 2005;79:13338–49. 16227256 10.1128/JVI.79.21.13338-13349.2005 PMC1262580

[23] Srivastava IK, Kan E, Sun Y, Sharma VA, Cisto J, Burke B, et al Comparative evaluation of trimeric envelope glycoproteins derived from subtype C and B HIV-1 R5 isolates. Virology 2008;372:273–90. 18061231 10.1016/j.virol.2007.10.022

[24] O'Hagan DT, Ott GS, Nest GV, Rappuoli R, Giudice GD. The history of MF59((R)) adjuvant: a phoenix that arose from the ashes. Expert Rev Vaccines 2013;12:13–30. 10.1586/erv.12.140 23256736

[25] De Rosa SC, Carter DK, McElrath MJ. OMIP-014: validated multifunctional characterization of antigen-specific human T cells by intracellular cytokine staining. Cytometry A 2012;81:1019–21. 10.1002/cyto.a.22218 23081852 PMC3581864

[26] Tomaras GD, Yates NL, Liu P, Qin L, Fouda GG, Chavez LL, et al Initial B-cell responses to transmitted human immunodeficiency virus type 1: virion-binding immunoglobulin M (IgM) and IgG antibodies followed by plasma anti-gp41 antibodies with ineffective control of initial viremia. J Virol 2008;82:12449–63. 10.1128/JVI.01708-08 18842730 PMC2593361

[27] Zolla-Pazner S, deCamp A, Gilbert PB, Williams C, Yates NL, Williams WT, et al Vaccine-induced IgG antibodies to V1V2 regions of multiple HIV-1 subtypes correlate with decreased risk of HIV-1 infection. PLoS One 2014;9:e87572 10.1371/journal.pone.0087572 24504509 PMC3913641

[28] Yates NL, Liao H, Fong Y, Camp A, Vandergrift NA, Williams WA, et al Vaccine-Induced Env V1-V2 IgG3 Correlate with Lower HIV-1 Infection Risk and Decline Soon After Vaccination. Science Translational Medicine 2014;6:228ra39 10.1126/scitranslmed.3007730 24648342 PMC4116665

[29] Liao HX, Bonsignori M, Alam SM, McLellan JS, Tomaras GD, Moody MA, et al Vaccine induction of antibodies against a structurally heterogeneous site of immune pressure within HIV-1 envelope protein variable regions 1 and 2. Immunity 2013;38:176–86. 10.1016/j.immuni.2012.11.011 23313589 PMC3569735

[30] Gaschen B, Taylor J, Yusim K, Foley B, Gao F, Lang D, et al Diversity considerations in HIV-1 vaccine selection. Science 2002;296:2354–60. 12089434 10.1126/science.1070441

[31] Liao HX, Sutherland LL, Xia SM, Brock ME, Scearce RM, Vanleeuwen S, et al A group M consensus envelope glycoprotein induces antibodies that neutralize subsets of subtype B and C HIV-1 primary viruses. Virology 2006;353:268–82. 17039602 10.1016/j.virol.2006.04.043 PMC1762135

[32] Montefiori DC. Measuring HIV neutralization in a luciferase reporter gene assay In: Prasad V.R, Kalpana G.V., editors. HIV protocols: Methods in Molecular Biology. Second Edition ed. Humana Press; 2009 p. 395–405. 10.1007/978-1-59745-170-3_26 19020839

[33] Seaman MS, Janes H, Hawkins N, Grandpre LE, Devoy C, Giri A, et al Tiered categorization of a diverse panel of HIV-1 Env pseudoviruses for assessment of neutralizing antibodies. J Virol 2010 2;84(3):1439–52. 10.1128/JVI.02108-09 19939925 PMC2812321

[34] Liu W. On sample size determination of Dunnett's procedure for comparing several treatments with a control. Journal of Statistical Planning and Inference 62[2], 255–261. 1997.

[35] Huang Y, Gilbert PB, Montefiori DC, Self SG. Simultaneous Evaluation of the Magnitude and Breadth of a Left and Right Censored Multivariate Response, with Application to HIV Vaccine Development. Stat Biopharm Res 2009 2 1;1(1):81–91. 20072667 10.1198/sbr.2009.0008 PMC2805400

[36] Gorse GJ, Newman MJ, deCamp A, Hay CM, De Rosa SC, Noonan E, et al DNA and modified vaccinia virus Ankara vaccines encoding multiple cytotoxic and helper T-lymphocyte epitopes of human immunodeficiency virus type 1 (HIV-1) are safe but weakly immunogenic in HIV-1-uninfected, vaccinia virus-naive adults. Clin Vaccine Immunol 2012 5;19(5):649–58. 10.1128/CVI.00038-12 22398243 PMC3346329

[37] Karasavvas N, Karnasutra C, Ngauy V, Vasan s, Trichavaroj R, de Souza M, et al. Investigation of Antibody Responses Induced in RV305 a Late Boost Vaccination of HIV-1 Uninfected Volunteers that Participated in RV144, a Thai Trial. AIDS Vaccine 2013 P03.68 LB. 2013.

[38] Morris L, Mkize NN, Hermanus T, Chung E, Sato A, Grant S, et al. Boosting antibody responses with gp140 protein two years after DNA/MVA priming: Results from the HVTN 073E Phase I vaccine trial. AID Vaccine 2013 P04.36 LB. 2013.

[39] Richmond JF, Lu S, Santoro JC, Weng J, Hu SL, Montefiori DC, et al Studies of the neutralizing activity and avidity of anti-human immunodeficiency virus type 1 Env antibody elicited by DNA priming and protein boosting. J Virol 1998 11;72(11):9092–100. 9765454 10.1128/jvi.72.11.9092-9100.1998 PMC110326

[40] Vaine M, Wang S, Hackett A, Arthos J, Lu S. Antibody responses elicited through homologous or heterologous prime-boost DNA and protein vaccinations differ in functional activity and avidity. Vaccine 2010 4 9;28(17):2999–3007. 10.1016/j.vaccine.2010.02.006 20170767 PMC2847033

[41] Letvin NL, Montefiori DC, Yasutomi Y, Perry HC, Davies ME, Lekutis C, et al Potent, protective anti-HIV immune responses generated by bimodal HIV envelope DNA plus protein vaccination. Proc Natl Acad Sci U S A 1997 8 19;94(17):9378–83. 9256490 10.1073/pnas.94.17.9378 PMC23198

[42] Pal R, Kalyanaraman VS, Nair BC, Whitney S, Keen T, Hocker L, et al Immunization of rhesus macaques with a polyvalent DNA prime/protein boost human immunodeficiency virus type 1 vaccine elicits protective antibody response against simian human immunodeficiency virus of R5 phenotype. Virology 2006 5 10;348(2):341–53. 16460776 10.1016/j.virol.2005.12.029

[43] Wang S, Kennedy JS, West K, Montefiori DC, Coley S, Lawrence J, et al Cross-subtype antibody and cellular immune responses induced by a polyvalent DNA prime-protein boost HIV-1 vaccine in healthy human volunteers. Vaccine 2008 7 23;26(31):3947–57. 18724414 10.1016/j.vaccine.2007.12.060 PMC3743087

[44] Frahm N, Friedrich D, Walsh P, DeRosa S, Spearman P, Barnett S, et al. A DNA Prime/Protein Boost Vaccine Leads to Higher B-cell Responses than a Vector Prime/Protein Boost or DNA Prime/Vector Boost Regimens. AIDS Vaccine Bangkok, Thailand, (OA10.06). 2011.

[45] Harari A, Bart PA, Stohr W, Tapia G, Garcia M, Medjitna-Rais E, et al An HIV-1 clade C DNA prime, NYVAC boost vaccine regimen induces reliable, polyfunctional, and long-lasting T cell responses. J Exp Med 2008 1 21;205(1):63–77. 10.1084/jem.20071331 18195071 PMC2234371

[46] Sandstrom E, Nilsson C, Hejdeman B, Brave A, Bratt G, Robb M, et al Broad immunogenicity of a multigene, multiclade HIV-1 DNA vaccine boosted with heterologous HIV-1 recombinant modified vaccinia virus Ankara. J Infect Dis 2008 11 15;198(10):1482–90. 10.1086/592507 18808335 PMC4793972

[47] Bakari M, Aboud S, Nilsson C, Francis J, Buma D, Moshiro C, et al Broad and potent immune responses to a low dose intradermal HIV-1 DNA boosted with HIV-1 recombinant MVA among healthy adults in Tanzania. Vaccine 2011 10 26;29(46):8417–28. 10.1016/j.vaccine.2011.08.001 21864626 PMC4795940

[48] Goepfert PA, Elizaga ML, Seaton K, Tomaras GD, Montefiori DC, Sato A, et al Specificity and 6-month durability of immune responses induced by DNA and recombinant modified vaccinia Ankara vaccines expressing HIV-1 virus-like particles. J Infect Dis 2014 7 1;210(1):99–110. 10.1093/infdis/jiu003 24403557 PMC4072895

[49] McCormack S, Stohr W, Barber T, Bart PA, Harari A, Moog C, et al EV02: a Phase I trial to compare the safety and immunogenicity of HIV DNA-C prime-NYVAC-C boost to NYVAC-C alone. Vaccine 2008 6 13;26(25):3162–74. 10.1016/j.vaccine.2008.02.072 18502003

[50] Gomez CE, Perdiguero B, Garcia-Arriaza J, Esteban M. Poxvirus vectors as HIV/AIDS vaccines in humans. Hum Vaccin Immunother 2012 9;8(9):1192–207. 10.4161/hv.20778 22906946 PMC3579898

[51] Garcia F, Bernaldo de Quiros JC, Gomez CE, Perdiguero B, Najera JL, Jimenez V, et al Safety and immunogenicity of a modified pox vector-based HIV/AIDS vaccine candidate expressing Env, Gag, Pol and Nef proteins of HIV-1 subtype B (MVA-B) in healthy HIV-1-uninfected volunteers: A phase I clinical trial (RISVAC02). Vaccine 2011 10 26;29(46):8309–16. 10.1016/j.vaccine.2011.08.098 21907749

[52] Gomez CE, Najera JL, Perdiguero B, Garcia-Arriaza J, Sorzano CO, Jimenez V, et al The HIV/AIDS vaccine candidate MVA-B administered as a single immunogen in humans triggers robust, polyfunctional, and selective effector memory T cell responses to HIV-1 antigens. J Virol 2011 11;85(21):11468–78. 10.1128/JVI.05165-11 21865377 PMC3194965

[53] Goepfert PA, Elizaga ML, Sato A, Qin L, Cardinali M, Hay CM, et al Phase 1 safety and immunogenicity testing of DNA and recombinant modified vaccinia Ankara vaccines expressing HIV-1 virus-like particles. J Infect Dis 2011 3 1;203(5):610–9. 10.1093/infdis/jiq105 21282192 PMC3072720

[54] Keefer MC, Frey SE, Elizaga M, Metch B, De Rosa SC, Barroso PF, et al A phase I trial of preventive HIV vaccination with heterologous poxviral-vectors containing matching HIV-1 inserts in healthy HIV-uninfected subjects. Vaccine 2011 2 24;29(10):1948–58. 10.1016/j.vaccine.2010.12.104 21216311 PMC3043112

[55] Yates NL, Liao HX, Fong Y, deCamp A, Vandergrift NA, Williams WT, et al Vaccine-induced Env V1-V2 IgG3 correlates with lower HIV-1 infection risk and declines soon after vaccination. Sci Transl Med 2014 3 19;6(228):228ra39 10.1126/scitranslmed.3007730 24648342 PMC4116665

[56] Tomaras GD, Ferrari G, Shen X, Alam SM, Liao HX, Pollara J, et al Vaccine-induced plasma IgA specific for the C1 region of the HIV-1 envelope blocks binding and effector function of IgG. Proc Natl Acad Sci U S A 2013 5 28;110(22):9019–24. 10.1073/pnas.1301456110 23661056 PMC3670311

