# Supplementary material for: Sequential Immunization with gp140 Boosts Immune Responses Primed by Modified Vaccinia Ankara or DNA in HIV-Uninfected South African Participants
Source: PLoS One. 2016 Sep 1;11(9):e0161753. doi: 10.1371/journal.pone.0161753 (PMC5008759; doi:10.1371/journal.pone.0161753)
Supplement: S1 Checklist — (DOC) [file pone.0161753.s001.doc]

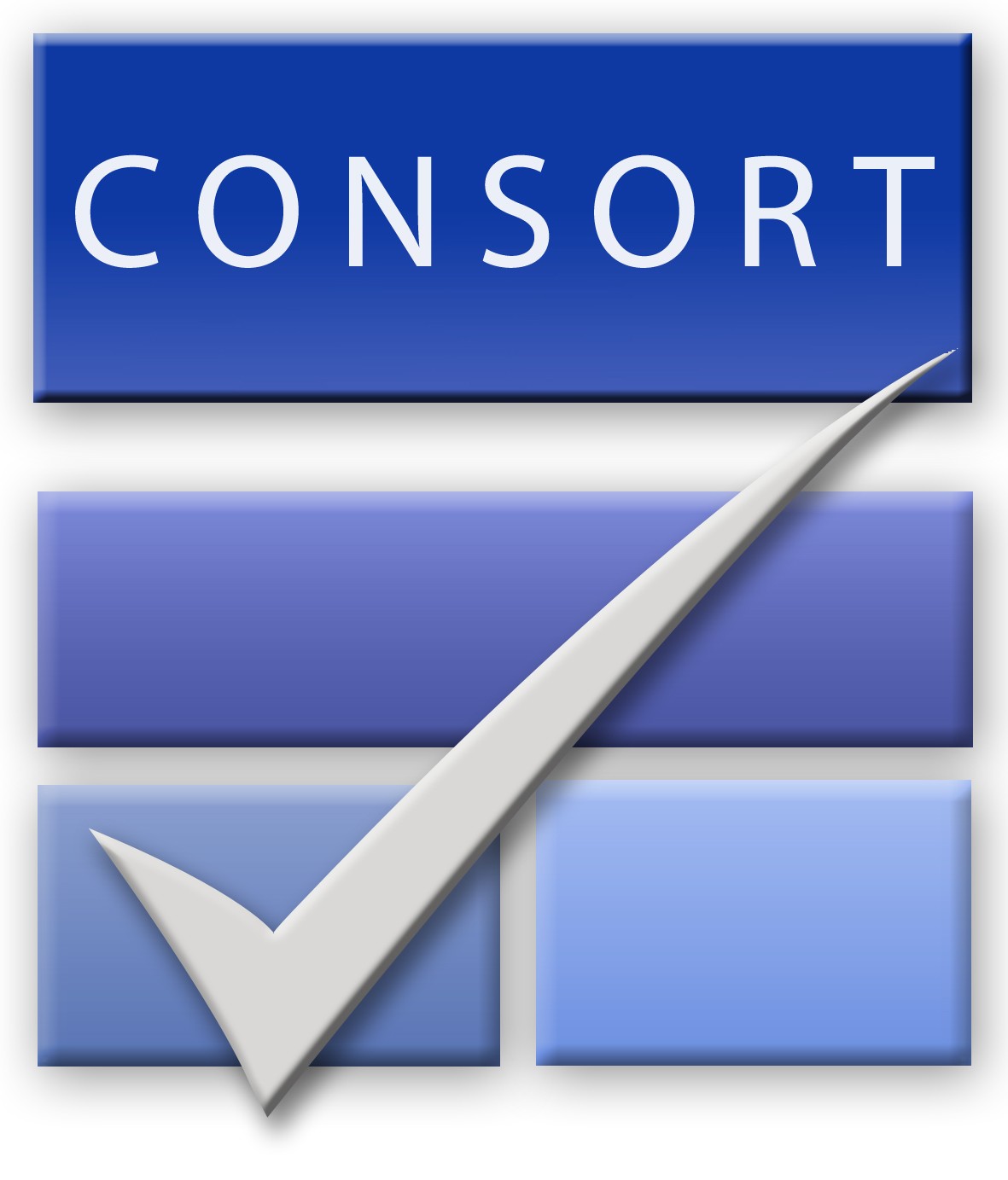
CONSORT 2010 checklist of information to include when reporting a randomised trial*

| Section/Topic | Item No | Checklist item | Reported on page No |
| --- | --- | --- | --- |
| Title and abstract | | | |
|  | 1a | Identification as a randomised trial in the title | Page 1  (Title page) |
|  | 1b | Structured summary of trial design, methods, results, and conclusions (for specific guidance see CONSORT for abstracts) | Page 3 (Abstract) |
| Introduction | | | |
| Background and objectives | 2a | Scientific background and explanation of rationale | Pages 4-5 (Introduction) |
|  | 2b | Specific objectives or hypotheses | Page 5 (Introduction) |
| Methods | | | |
| Trial design | 3a | Description of trial design (such as parallel, factorial) including allocation ratio | Pages 5 (Study population) & 7 (Randomisation and blinding) |
|  | 3b | Important changes to methods after trial commencement (such as eligibility criteria), with reasons | Pages 6-7 (Eligibility criteria) |
| Participants | 4a | Eligibility criteria for participants | Pages 6-7 (Eligibility criteria) |
|  | 4b | Settings and locations where the data were collected | Page 5 (Study population) |
| Interventions | 5 | The interventions for each group with sufficient details to allow replication, including how and when they were actually administered | Page 5, 7-9,10-12 (Study population; Vaccine regimens; Safety assessment; Laboratory assays, Table 1) |
| Outcomes | 6a | Completely defined pre-specified primary and secondary outcome measures, including how and when they were assessed | Pages 12-17 (Statistical methods; Endpoints) |
|  | 6b | Any changes to trial outcomes after the trial commenced, with reasons | NA |
| Sample size | 7a | How sample size was determined | Pages 12-17 (Statistical methods) |
|  | 7b | When applicable, explanation of any interim analyses and stopping guidelines | Not applicable |
| Randomisation: |  |  |  |
| Sequence generation | 8a | Method used to generate the random allocation sequence | Page 7 (Randomization and blinding) |
|  | 8b | Type of randomisation; details of any restriction (such as blocking and block size) | Page 7 (Randomization and blinding) |
| Allocation concealment mechanism | 9 | Mechanism used to implement the random allocation sequence (such as sequentially numbered containers), describing any steps taken to conceal the sequence until interventions were assigned | Page 7 (Randomization and blinding) |
| Implementation | 10 | Who generated the random allocation sequence, who enrolled participants, and who assigned participants to interventions | Page 7 (Randomization and blinding) |
| Blinding | 11a | If done, who was blinded after assignment to interventions (for example, participants, care providers, those assessing outcomes) and how | Page 7 (Randomization and blinding) |
|  | 11b | If relevant, description of the similarity of interventions | Pages 7-8 (Vaccine regimens) |
| Statistical methods | 12a | Statistical methods used to compare groups for primary and secondary outcomes | Pages 12-14 (Statistical methods) |
|  | 12b | Methods for additional analyses, such as subgroup analyses and adjusted analyses | Pages 12-16 (Statistical methods and Endpoints) |
| Results | | | |
| Participant flow (a diagram is strongly recommended) | 13a | For each group, the numbers of participants who were randomly assigned, received intended treatment, and were analysed for the primary outcome | Page 17 (Participant accrual and demographics; Figure 1) |
|  | 13b | For each group, losses and exclusions after randomisation, together with reasons | Pages 16-19 (Participant accrual and demographics; Discontinuation of vaccinations; Early termination from study; Figure 1) |
| Recruitment | 14a | Dates defining the periods of recruitment and follow-up | Page 17 (Participant accrual and demographics) |
|  | 14b | Why the trial ended or was stopped | NA |
| Baseline data | 15 | A table showing baseline demographic and clinical characteristics for each group | Page 17, 18  (Table 2) |
| Numbers analysed | 16 | For each group, number of participants (denominator) included in each analysis and whether the analysis was by original assigned groups | Pages 17-29 (Results) |
| Outcomes and estimation | 17a | For each primary and secondary outcome, results for each group, and the estimated effect size and its precision (such as 95% confidence interval) | Pages 17-29 (Results) |
|  | 17b | For binary outcomes, presentation of both absolute and relative effect sizes is recommended | NA |
| Ancillary analyses | 18 | Results of any other analyses performed, including subgroup analyses and adjusted analyses, distinguishing pre-specified from exploratory | Pages 17-29 (Results) |
| Harms | 19 | All important harms or unintended effects in each group (for specific guidance see CONSORT for harms) | Pages 17-29 (Results) |
| Discussion | | | |
| Limitations | 20 | Trial limitations, addressing sources of potential bias, imprecision, and, if relevant, multiplicity of analyses | Page 32 (Comparison with RV144) |
| Generalisability | 21 | Generalisability (external validity, applicability) of the trial findings | Pages 29 – 33 (Discussion) |
| Interpretation | 22 | Interpretation consistent with results, balancing benefits and harms, and considering other relevant evidence | Pages 29 – 33 (Discussion) |
| Other information | | |  |
| Registration | 23 | Registration number and name of trial registry | Page 7 (Regulatory approvals and trial registration) |
| Protocol | 24 | Where the full trial protocol can be accessed, if available | Pages 34  (Data & protocol availability) |
| Funding | 25 | Sources of funding and other support (such as supply of drugs), role of funders | Provided separately |

*We strongly recommend reading this statement in conjunction with the CONSORT 2010 Explanation and Elaboration for important clarifications on all the items. If relevant, we also recommend reading CONSORT extensions for cluster randomised trials, non-inferiority and equivalence trials, non-pharmacological treatments, herbal interventions, and pragmatic trials. Additional extensions are forthcoming: for those and for up to date references relevant to this checklist, see [www.consort-statement.org](http://www.consort-statement.org/).
