# Supplementary material for: Sequential Immunization with gp140 Boosts Immune Responses Primed by Modified Vaccinia Ankara or DNA in HIV-Uninfected South African Participants
Source: PLoS One. 2016 Sep 1;11(9):e0161753. doi: 10.1371/journal.pone.0161753 (PMC5008759; doi:10.1371/journal.pone.0161753)
Supplement: S1 Protocol — (PDF) [file pone.0161753.s002.pdf]

HIV VACCINE
TRIALS NETWORK

PROTOCOL

HVTN 086 / SAAVI 103

A phase 1 placebo-controlled clinical trial to evaluate the safety and immunogenicity of SAAVI DNA-C2, SAAVI MVA-C and Novartis subtype C gp140 with MF59 adjuvant in various vaccination schedules in HIV-uninfected healthy vaccinia-naïve adult participants in South Africa

DAIDS DOCUMENT ID 11688

CLINICAL TRIAL SPONSORED BY

South African AIDS Vaccine Initiative (SAAVI),
Medical Research Council of South Africa (MRC-SA)
Cape Town, Republic of South Africa

STUDY PRODUCTS PROVIDED BY

Division of AIDS (DAIDS)
National Institute of Allergy and Infectious Diseases (NIAID)
National Institutes of Health (NIH)
Department of Health and Human Services (DHHS)
Bethesda, Maryland, USA

STUDY PRODUCTS DEVELOPED BY

South African AIDS Vaccine Initiative (SAAVI), Medical Research Council of South Africa (MRC–SA) and
University of Cape Town (UCT)
Cape Town, Republic of South Africa

Novartis Vaccines and Diagnostics, Inc
Cambridge, Massachusetts, USA

September 28, 2011

HVTN 086 / SAAVI 103, Version 2-0

Contents

1 Ethical considerations 5

2 IRB/IEC review considerations 7
- 2.1 Minimized risks to participants 7
- 2.2 Reasonable risk/benefit balance 8
- 2.3 Equitable subject selection 8
- 2.4 Appropriate informed consent 8
- 2.5 Adequate safety monitoring 8
- 2.6 Protect privacy/confidentiality 9

3 Overview 10
- 3.1 Protocol Team 14

4 Background 15
- 4.1 Rationale for trial concept 15
- 4.2 SAAVI DNA-C2 18
- 4.3 SAAVI MVA-C 21
- 4.4 Novartis subtype C gp140 22
- 4.5 Novartis MF59 adjuvant 23
- 4.6 Trial design rationale 23
- 4.7 Plans for future product development and testing 27
- 4.8 Preclinical safety studies 28
- 4.9 Preclinical immunogenicity studies 33
- 4.10 Clinical studies 44
- 4.11 Potential risks of study products and administration 50

5 Objectives and endpoints 51
- 5.1 Primary objectives and endpoints 51
- 5.2 Secondary objectives and endpoints 51
- 5.3 Exploratory objectives 52

6 Statistical considerations 54
- 6.1 Accrual and sample size calculations 54
- 6.2 Randomization 58
- 6.3 Blinding 58
- 6.4 Statistical analysis 59

7 Selection and withdrawal of participants 65
- 7.1 Inclusion criteria 65
- 7.2 Exclusion criteria 67
- 7.3 Participant departure from vaccination schedule or withdrawal 71

8 Study product preparation and administration 74
- 8.1 Vaccine regimen 74
- 8.2 Study product formulation 78
- 8.3 Preparation of study products 79
- 8.4 Administration 80
- 8.5 Acquisition of study products 80
- 8.6 Pharmacy records 81
- 8.7 Final disposition of study products 81

9 Clinical procedures 82

9.1 Informed consent ... 82
9.2 Pre-enrollment procedures ... 84
9.3 Enrollment and vaccination visits ... 85
9.4 Follow-up visits ... 87
9.5 Mucosal secretion sampling ... 90
9.6 Extended Safety Surveillance ... 91
9.7 HIV counseling and testing ... 91
9.8 Contraception status ... 92
9.9 Urinalysis ... 93
9.10 Assessments of reactogenicity ... 93
9.11 Visit windows and missed visits ... 94
9.12 Early termination visit ... 95
9.13 Pregnancy ... 95

10 Laboratory ... 96
10.1 HVTN CRS laboratory procedures ... 96
10.2 Total blood volume ... 96
10.3 Primary immunogenicity timepoints ... 96
10.4 Endpoint assays: humoral ... 96
10.5 Endpoint assays: cellular ... 97
10.6 Genotyping ... 98
10.7 Exploratory studies ... 98
10.8 Other use of stored specimens ... 99
10.9 Biohazard containment ... 99

11 Safety monitoring and safety review ... 100
11.1 Safety monitoring and oversight ... 100
11.2 Safety reporting ... 101
11.3 Safety reviews ... 103
11.4 Safety pause and prompt PSRT AE review ... 104
11.5 Review of cumulative safety data ... 105
11.6 Study termination ... 106

12 Protocol conduct ... 107
12.1 Overview of data collection methods ... 107
12.2 HVTN CRS monitoring ... 109
12.3 Social impacts ... 110
12.4 Study participant reimbursement ... 110
12.5 Compliance with NIH guidelines for research involving products containing recombinant DNA ... 110
12.6 Specific regulatory considerations for South Africa ... 111

13 Version history ... 112

14 Document references (other than literature citations) ... 113

15 Acronyms and abbreviations ... 115

16 Literature cited ... 118

Appendix A: Sample informed consent form ... 125

Appendix B: Sample informed consent form for extended safety monitoring ... 143

Appendix C: Approved birth control methods (for sample informed consent form) ... 148

Appendix D: Table of procedures (for sample informed consent form) ... 149

Appendix E: Laboratory procedures 150
Appendix F: Procedures at HVTN CRS 151
Appendix G: Extended Safety Surveillance 152
Appendix H: Case definition of myo/pericarditis for use in adverse events monitoring 153
Appendix I: HVTN 086 / SAAVI 103 Participant Injection Schedule 154
Appendix J: HVTN VISP registry consent 155

1 Ethical considerations

Multiple candidate HIV vaccines will need to be studied simultaneously in different populations around the world before a successful HIV preventive vaccine is found. It is critical that universally accepted ethical guidelines are followed at all sites involved in the conduct of these clinical trials. The HIV Vaccine Trials Network (HVTN) has addressed ethical concerns in the following ways:

- HVTN trials are designed and conducted to enhance the knowledge base necessary to find a preventive vaccine, using methods that are scientifically rigorous and valid, and in accordance with Good Clinical Practice (GCP) guidelines.

- HVTN scientists and operational staff incorporate the philosophies underlying major codes [1-3], declarations, and other guidance documents relevant to human subjects research into the design and conduct of HIV vaccine clinical trials.

- HVTN scientists and operational staff are committed to substantive community input—into the planning, conduct, and follow-up of its research—to help ensure that locally appropriate cultural and linguistic needs of study populations are met. Community Advisory Boards (CABs) are required by DAIDS and supported at all HVTN research sites to ensure community input. The HVTN leadership is aware of the Guidelines for Good Clinical Practice in the Conduct of Clinical Trials with Human Participants in South Africa section 2.4 "Communication and Community Involvement" and the South African Medical Research Council's Guidelines on Ethics for Medical Research: HIV Preventive Vaccine Research (particularly section 5, Community Participation) and works to implement both guidelines generally and those sections specifically.

- HVTN clinical trial staff members counsel each participant at each study visit on how to reduce HIV risk. Participants who become HIV-infected during the trial are provided counseling on notifying their partners and about HIV infection according to local guidelines. Staff members will also counsel them about reducing their risk of transmitting HIV to others.

- The HVTN requires that all international HVTN sites lacking national plans for providing antiretroviral therapy (ART) develop plans for the care and treatment of participants who acquire HIV infection during a trial. Each plan is developed in consultation with representatives of host countries, communities from which potential trial participants will be drawn, sponsors, and the HVTN. Participants will be referred to programs for ART provision when the appropriate criteria for starting ART are met. If a program is not available at a site and ART is needed, a privately established fund will be used to pay for access to treatment to the fullest extent possible.

- The HVTN provides training so that all participating sites similarly ensure fair participant selection, protect the privacy of research participants, and obtain meaningful informed consent. During the study, participants will have their

wellbeing monitored, and to the fullest extent possible, their privacy protected. Participants may withdraw from the study at any time.

- Prior to implementation, HVTN trials are rigorously reviewed by scientists who are not involved in the conduct of the trials under consideration.

- HVTN trials are reviewed by local and national regulatory bodies and are conducted in compliance with all applicable national and local regulations.

- The HVTN designs its research to minimize risk and maximize benefit to both study participants and their local communities. For example, HVTN protocols provide enhancement of participants' knowledge of HIV and HIV prevention, as well as counseling, guidance, and assistance with any social impacts that may result from research participation. HVTN protocols also include careful medical review of each research participant's health conditions and reactions to study products while in the study.

- HVTN research aims to benefit local communities by directly addressing the health and HIV prevention needs of those communities and by strengthening the capacity of the communities through training, support, shared knowledge, and equipment. Researchers involved in HVTN trials are able to conduct other critical research in their local research settings.

- The HVTN recognizes the importance of institutional review and values the role of in-country Institutional Review Boards (IRBs) and Independent Ethics Committees (IECs) as custodians responsible for ensuring the ethical conduct of research in each local setting.

2 IRB/IEC review considerations

United States (US) federal regulations require IRBs or IECs to ensure that certain requirements are satisfied on initial and continuing review of research (Title 45, Code of Federal Regulations (CFR), Part 46.111(a) 1-7). The following section highlights how this protocol addresses each of these research requirements. Each HVTN Investigator welcomes IRB/IEC questions or concerns regarding these research requirements.

This trial is being conducted exclusively in South Africa, with funding from the U. S. NIH and SAAVI. Due to this, the trial is subject to both US and South African regulations and guidelines on the protection of human research subjects and ethical research conduct. These research regulations and guidelines are based on ethical principles of respect for persons, beneficence and nonmaleficence, and justice. Where there is a conflict in regulations or guidelines, the regulation or guideline providing the maximum protection of human research subjects will be followed.

In compliance with the Guidelines For Good Practice In The Conduct Of Clinical Trials In Human Participants In South Africa (“South African GCPs”), each research location in South Africa has a South African-based Principal Investigator (PI) who is qualified to conduct (and supervise the conduct of) the research; and the research addresses an important South African health need: the need for an HIV vaccine that can protect against the specific subtype of HIV circulating in South Africa. In addition, the investigators take responsibility for the conduct of the study and the control of the study products, including obtaining all appropriate South African regulatory and ethical reviews of the research. Each participating site has a standard operating procedure for ensuring that participants have the necessary information to make a decision whether or not to consent to the research. The sections below address each of the review concerns by IRBs/ethics committees regarding how the research will be conducted.

2.1 Minimized risks to participants

45 CFR 46.111 (a) 1: Risks to subjects are minimized.

This protocol minimizes risks to participants by (a) correctly and promptly informing participants about risks so that they can join in partnership with the researcher in recognizing and reporting harms; (b) respecting local/national blood draw limits; (c) performing direct observation of participants postvaccination and collecting information regarding side effects for several days postvaccination; (d) having staff properly trained in administering study procedures that may cause physical harm or psychological distress, such as blood draws, vaccinations, HIV testing and counseling and HIV risk reduction counseling; (e) providing HIV risk reduction counseling and checking on contraception use (for women); and (f) providing safety monitoring.

2.2 Reasonable risk/benefit balance

45 CFR 46.111 (a) 2: Risks to subjects are reasonable in relation to anticipated benefits, if any, to subjects, and the importance of the knowledge that may reasonably be expected to result.

In all public health research, the risk-benefit ratio may be difficult to assess because the benefits to a healthy participant are not as apparent as they would be in treatment protocols, where a study participant may be ill and may have exhausted all conventional treatment options. However, this protocol is designed to minimize the risks to participants while maximizing the potential value of the knowledge it is designed to generate.

2.3 Equitable subject selection

45 CFR 46.111 (a) 3: Subject selection is equitable

This protocol has specific inclusion and exclusion criteria for investigators to follow in admitting participants into the protocol. Participants are selected because of these criteria and not because of positions of vulnerability or privilege. Investigators are required to maintain screening and enrollment logs to document volunteers who screened into and out of the protocol and for what reasons.

2.4 Appropriate informed consent

45 CFR 46.111 (a) 4 & 5: Informed consent is sought from each prospective subject or the subject's legally authorized representative as required by 45 CFR 46.416; informed consent is appropriately documented as required by 45 CFR 46.417

The protocol specifies that informed consent must be obtained before any study procedures are initiated and assessed throughout the trial (see section 9.1). Each site is provided training in informed consent by the HVTN as part of its entering the HVTN. The HVTN requires a signed consent document for documentation, in addition to chart notes or a consent checklist.

2.5 Adequate safety monitoring

45 CFR 46.111 (a) 6: There is adequate provision for monitoring the data collected to ensure the safety of subjects.

This protocol has extensive safety monitoring in place (see section 11). Safety is monitored daily by clinical affairs staff and routinely by the Protocol Safety Review Team (PSRT). Site staff have 24-hour cell phone access to clinical affairs staff. In addition, the HVTN Safety Monitoring Board (SMB) periodically reviews unblinded data.

2.6 Protect privacy/confidentiality

45 CFR 46.111 (a) 7: There are adequate provisions to protect the privacy of subjects and maintain the confidentiality of data.

Privacy refers to an individual's right to be free from unauthorized or unreasonable intrusion into his/her private life and the right to control access to individually identifiable information about him/her. The term "privacy" concerns research participants or potential research participants as individuals whereas the term "confidentiality" is used to refer to the treatment of information about those individuals. This protocol respects the privacy of participants by informing them about who will have access to their personal information and study data (see Appendix A, section 17). The privacy of participants is protected by assigning unique identifiers in place of the participant's name on study data and specimens.

3 Overview

Title

A phase 1 placebo-controlled clinical trial to evaluate the safety and immunogenicity of SAAVI DNA-C2, SAAVI MVA-C and Novartis subtype C gp140 with MF59 adjuvant in various vaccination schedules in HIV-uninfected healthy vaccinia-naïve adult participants in South Africa

Primary objectives

To evaluate the safety and tolerability of IM administration of SAAVI DNA-C2, SAAVI MVA-C and Novartis subtype C gp140/MF59 vaccines, in HIV-uninfected healthy vaccinia-naïve adult participants in South Africa.

To characterize and rank the four vaccine regimens and to select the best performing vaccine regimen based on HIV-specific neutralizing antibody (nAb) responses following vaccination with Novartis subtype C gp140/MF59 vaccine, as a concurrent or sequential boost to SAAVI MVA-C prime; SAAVI MVA-C boost to SAAVI DNA-C2 prime; and SAAVI MVA-C with concurrent Novartis subtype C gp140/MF59 boost to SAAVI DNA-C2 prime in HIV-uninfected healthy vaccinia-naïve adult participants in South Africa.

Study products and routes of administration

- **SAAVI DNA-C2**: Multigene HIV-1 subtype C DNA vaccine comprising 2 DNA plasmids, expressing a polyprotein Gag-RT-Tat-Nef (pVRCgrttnC) and truncated gp160 (pVRCgp150CT). The DNA has been vialed at a concentration of 4 mg/mL. A 4 mg dose is given as a single 1 mL IM injection into the deltoid.

- **SAAVI MVA-C**: Multigene HIV-1 subtype C recombinant modified vaccinia Ankara (MVA) virus vaccine expressing Gag-RT-Tat-Nef (grttn), and gp150CT. Grttn is inserted into 1 insertion site in MVA and gp150CT into a second insertion site in the same MVA. The MVA has been vialed at a titer of 2.9×10^9 pfu/mL. A 1.45×10^9 pfu dose is given as a single 0.5 mL IM injection into the deltoid.

- **Novartis subtype C gp140**: HIV-1 subtype C recombinant oligomeric V2 deleted gp140 (TV1gp140ΔV2) produced in Chinese Hamster Ovary (CHO) cells. A100 mcg dose is combined with MF59 and is given as a single 0.5 mL IM injection into the deltoid.

- **Novartis MF59 adjuvant**: MF59C.1 is an oil-in-water emulsion with a squalene internal oil phase and a citrate buffer external aqueous phase. Two nonionic surfactants, sorbitan trioleate and polysorbate 80, serve to stabilize the emulsion.

- **Placebo for SAAVI DNA-C2**: Sodium Chloride for Injection, 0.9% is given as a single 1mL IM injection into the deltoid.

- **Placebo for SAAVI MVA-C and Novartis subtype C gp140/MF59 adjuvant**: Sodium Chloride for Injection, 0.9% is given as a single 0.5mL IM injection into the deltoid.

Table 3-1 Schema

Study arm	Number participants	SAAVI DNA-C2 (mg)	SAAVI MVA-C (pfu)	Novartis gp140 (mcg)	Month 0	Month 1	Month 3	Month 6
Group 1	38	-	$1.45{\times}10^9$	100	Placebo + MVA-C	MVA-C	Placebo + gp140/MF59	Placebo + gp140/MF59
	8	-	0	0	Placebo + Placebo	Placebo	Placebo + Placebo	Placebo + Placebo
Group 2	38	-	$1.45{\times}10^9$	100	MVA-C + gp140/MF59	Placebo	MVA-C + gp140/MF59	Placebo + Placebo
	8	-	0	0	Placebo + Placebo	Placebo	Placebo + Placebo	Placebo + Placebo
Group 3	38	4mg	$1.45{\times}10^9$	-	Placebo + DNA-C2	DNA-C2	Placebo + MVA-C	Placebo + MVA-C
	8	0	0	-	Placebo + Placebo	Placebo	Placebo + Placebo	Placebo + Placebo
Group 4	38	4mg	$1.45{\times}10^9$	100	Placebo + DNA-C2	DNA-C2	MVA-C + gp140/MF59	MVA-C + gp140/MF59
	8	0	0	0	Placebo + Placebo	Placebo	Placebo + Placebo	Placebo + Placebo
Total	184 (152 vaccine / 32 placebo)							

Note: Enrollment will be restricted to a maximum of 1 participant per group per day until 5 participants have been enrolled in each group. The PSRT will review the safety and reactogenicity data reported for the first 72 hours postvaccination on each of these participants and will determine whether it is safe to proceed with full enrollment.

Participants

184 HIV-uninfected healthy vaccinia-naïve adult participants in South Africa aged 18 to 45 years; 152 vaccinees, 32 placebo recipients

Design

Multicenter, randomized, placebo-controlled, double-blind trial

Duration per participant

3 years per participant (includes 12 months of scheduled clinic visits followed by 2 years of annual contacts for purposes of extended safety surveillance).

Estimated total study duration

4 years (includes enrollment, planned safety hold, and extended safety surveillance)

Medicines Control Council applicant, South Africa

National PI, Gavin J. Churchyard, Center for the AIDS Programme for Research in South Africa (CAPRISA), Aurum CRS, Klerksdorp

Study product provider: DAIDS, NIAID (Bethesda, MD, USA)

Study product developers:

- SAAVI DNA-C2 vaccine: SAAVI, MRC-SA, and UCT (Cape Town, Republic of South Africa)
- SAAVI MVA-C vaccine: SAAVI, MRC-SA, and UCT (Cape Town, Republic of South Africa)
- Novartis subtype C gp140 vaccine: Novartis Vaccine and Diagnostics, Inc. (Cambridge, MA, USA), formerly the Chiron Corporation
- Novartis MF59 adjuvant: Novartis Vaccine and Diagnostics, Inc. (Cambridge, MA, USA)

Core operations

HVTN Vaccine Leadership Group/Core Operations Center, Fred Hutchinson Cancer Research Center (FHCRC) (Seattle, Washington, USA)

Statistical and data management center (SDMC)

Statistical Center for HIV/AIDS Research and Prevention (SCHARP), FHCRC (Seattle, Washington, USA)

HIV diagnostic laboratory

Serology-Virology & Special Molecular Diagnostic Unit, National Institute for Communicable Diseases (Johannesburg, South Africa)

Endpoint assay laboratories

- Duke University Medical Center (Durham, North Carolina, USA)
- South African Immunology Laboratory - National Institute for Communicable Diseases (SAIL-NICD) (Johannesburg, South Africa)

- FHCRC/University of Washington (Seattle, Washington, USA)

Study sites

HVTN Clinical Research Sites (HVTN CRSs) in South Africa, to be specified in the Site Announcement Memo

Safety monitoring

HVTN 086 / SAAVI 103 PSRT; HVTN Safety Monitoring Board (SMB)

3.1 Protocol Team

Protocol leadership

Chair — Gavin Churchyard
CAPRISA Aurum CRS
27-11-638-2228
gchurchyard@auruminstitute.org

Cochair — Koleka Mlisana
CAPRISA Thekwini CRS
27-11-242-7378
mlisanak@ukzn.ac.za

Protocol Team leader — Shelly Karuna
HVTN Core, FHCRC
206-667-4355
skaruna@hvtn.org

Statistician — Peter Gilbert
SCHARP, FHCRC
206-667-7299
pgilbert@scharp.org

Medical officer — Mary Allen
DAIDS, NIAID
301-402-2310
mallen@niaid.nih.gov

Other contributors to the original protocol

Core medical monitor — Shelly Karuna, HVTN Core, FHCRC

Vaccine developer representative — Carolyn Williamson, University of Cape Town

Vaccine developer representative — Anna-Lise Williamson, University of Cape Town

Vaccine developer representative — Susan Barnett, Novartis Vaccines and Diagnostics, Inc

Laboratory Program representative — John Hural, HVTN Laboratory Program, FHCRC

Laboratory Program representative — Jin Bae, HVTN Laboratory Program, FHCRC

Regulatory affairs — Renee Holt, HVTN Core, FHCRC

Clinic coordinator — Tanya Nielson, CAPRISA Aurum CRS

CAB member — Nombeko Mpongo, Desmond Tutu HIV Foundation

CAB member — Papa Vusi Radebe, Perinatal HIV Research Unit

HVTN pharmacist — Jan Johannessen, HVTN Core, FHCRC

DAIDS protocol pharmacist — Scharla Estep, DAIDS, NIAID

Medical officer — Adam Sherwat, DAIDS, NIAID

Statistical research associate — Kruti Pandya, SCHARP, FHCRC

Clinical affairs representative — Hsui-Ying Huang, SCHARP, FHCRC

Clinical affairs representative — Maija Anderson, SCHARP, FHCRC

Clinical trials manager — Niles Eaton, HVTN Core, FHCRC

Laboratory Program scientist — Georgia Tomaras, Duke University

Laboratory Program scientist — David Montefiori, Duke University

Project manager — Shelly Mahilum, SCHARP, FHCRC

Senior project manager — Drienna Holman, SCHARP, FHCRC

Protocol development coordinator — Julie Foster, HVTN Core, FHCRC

Community relations and education unit representative — Genevieve Meyer, HVTN Core, FHCRC

Community educator/recruiter — Tabita Mahlangeni, CAPRISA Aurum CRS

Technical editor — Anya Luke-Killam, HVTN Core, FHCRC

4 Background

4.1 Rationale for trial concept

The number of people living with HIV/AIDS in 2008 was estimated to be between 31million and 36 million people, with sub-Saharan Africa accounting for approximately 71% of new infections. In 2008, there were an estimated 2.7 million people newly infected with HIV. National population-based surveys from 2001-2008 among adults age 15-49 revealed southern Africa to be hardest hit by the epidemic with HIV seroprevalence rates as high as 25% in Botswana and Swaziland, 23% in Lesotho and 17% in South Africa. An estimated 5.7 million South Africans are infected with HIV [4].

In southern Africa, subtype C accounts for over 95% of infections [5-8]. Subtype C is also largely responsible for the epidemics in Ethiopia [9] and India [10], and accounts for over 50% of HIV-1 infections globally [11]. In response to the devastating subtype C epidemic in southern Africa, the South African AIDS Vaccine Initiative (SAAVI), a lead program of the Medical Research Council of South Africa (MRC-SA), in collaboration with the University of Cape Town (UCT) and the US NIH, has developed 2 subtype C HIV vaccines—SAAVI DNA-C2 and SAAVI MVA-C. The vaccines were initially developed to be used in a prime-boost protocol as this induced a better immune response in preclinical studies than either vaccine alone. The immune response seen with this vaccine regimen appears to be primarily a cell-mediated response.

However, the results of the Step trial called into question the value of an HIV vaccine regimen that elicits primarily cell-mediated immune responses. A high frequency of cellular immune responses was seen to a limited number of vaccine specific antigens with the Merck & Co. (Whitehouse Station, NJ, USA) trivalent recombinant Adenovirus serotype 5 (rAd5) vectored vaccine evaluated in the Step trial. However, the trial was stopped prematurely as there was no evidence that the vaccine provided protection against HIV infection. The narrowness of the immune response that was elicited is thought to be an important factor in the vaccine's overall lack of efficacy [12]. Furthermore, the initial results of the Step trial suggested that vaccinated individuals with pre-existing Ad5 nAbs may be at increased risk of acquiring HIV infection [13]. However, after unblinding, the initial difference in the number of HIV infections in vaccinees compared to placebo recipients decreased over time [14].

The hypothetical importance of induction of antibodies for protection against HIV acquisition has been highlighted in the recent results of a trial (RV144) of a recombinant canarypox vector vaccine prime (ALVAC B/E) with a B/E gp120 subunit vaccine boost (AIDSVAX) in Thailand [15]. That trial suggests that eliciting T-helper and antibody (Ab) responses may be important to prevent HIV infection, although immune correlates for the modest protection against HIV acquisition seen in that trial are yet to be determined.

The RV144 trial is the only HIV vaccine trial ever to demonstrate a level of protective efficacy, however modest. HVTN 086 / SAAVI 103 is inspired and informed by this latest building block in the iterative process of HIV vaccine trial development. This trial evaluates various combinations of 3 vaccine candidates: SAAVI DNA-C2, SAAVI

MVA-C, and Novartis oligomeric, subtype C (TV1) glycoprotein 140 with the variable loop 2 deleted (TV1gp140ΔV2).

Two of these 3 vaccine candidates are currently under evaluation in humans in a prime-boost regimen in the HVTN 073/SAAVI 102 trial. That trial aims to evaluate the safety and immunogenicity of SAAVI DNA-C2 followed by boosting with SAAVI MVA-C and initial results are promising, though data analysis is not yet complete (see Section 4.10.1 for more detail). Current data indicate that CD4+ or CD8+ responses for any protein were 24/31 for vaccinees (77%), with CD4+ responses predominant. This is consistent with the pre-clinical nonhuman primate (NHP) peripheral blood mononuclear cell (PBMC) responses; and these responses are nearly double the frequency of that seen with the ALVAC priming regimen used in RV144.

In RV144, the frequency of T-cell responses induced by the vaccination regimen was low. We posit that enhanced priming of the initial immune response in addition to more directed boosting with a protein adjuvant combination will be needed to improve upon these results.

HVTN 086 / SAAVI 103 may be expected to elicit better immunogenicity than observed in RV144 because of differences in the pox vector used, as well. HVTN 086 / SAAVI 103 tests MVA in combination with a protein boost, while RV144 utilized the avipoxvirus vector based on canarypox virus (CPV), ALVAC. HVTN 055 tested different combinations of 2 vaccines based on MVA and avipoxvirus vector fowlpox virus (FPV). There were 2 pairs of vaccines with 1 set expressing Env and Gag and the other set expressing Tat, Rev, and Nef-RT. Interestingly, data from HVTN 055 demonstrate that, while a higher immune response rate was seen with MVA alone than with FPV alone, a heterologous prime-boost elicited a greater immune response rate than either MVA or FPV alone (see Table 4-1).

Table 4-1 HVTN 055: IFN-γ enzyme-linked immunospot (ELISpot) overall (any antigen) response rates

Group (number of vaccinations with specific vaccines)	Responses (%)	
	Post-3rd vaccination	Post-5th vaccination
1 (5x FPV 10e9 Alone)	1/28 (3.6%)	2/25 (8%)
2 (2x MVA 10e7 + 3 x FPV10e9)	2/8 (25%)	5/8 (62.5%)
3 (2 x MVA 10e8 + 3 x FPV10e9)	5/9 (55.6%)	5/8 (62.5%)
4 (2 x MVA 10e9 + 3 x FPV10e9)	16/27 (59.3%)*	12/19 (63.2%)^
5 (5 x MVA 10e 9 Alone)	8/29 (27.6%)	6/26 (23.1%)

*95% CI: 39-78%; p = 0.03 vs Gp 5 by Fisher's exact test
^95% CI: 38-84%; p = 0.01 vs Gp 5

It is important to note the differences in the products used and vaccination strategy between RV144 and HVTN 086 / SAAVI 103. Both trials include a pox virus vectored vaccine. The ALVAC product is a recombinant canarypox vector (vCP1521), produced

by Sanofi Pasteur, engineered to express HIV-1 Gag and Protease (subtype B, LAI strain) and CRF01_AE (subtype E) HIV-1 gp120 (92TH023) linked to the transmembrane 3 anchoring portion of gp41 (LAI). The SAAVI pox virus vectored product is a recombinant MVA vector with Gag- reverse transcriptase (RT)-Tat-Nef and subtype C truncated gp150, described in detail in section 4.3. The ALVAC vaccine was used as a prime whereas the SAAVI MVA-C is used as a prime in 1 arm (group 1) and as a boost, with (group 4) or without (group 3) TV1gp140ΔV2, and in a concurrent vaccination strategy with TV1gp140ΔV2 (group 2) (see study schema, Table 3-1).

The envelope proteins used in the 2 trials also differ. The AIDSVAX product is a bivalent B/E gp120 (CM235/SF2) with adjuvant (Alum) (provided by VaxGen, now Global Solutions for Infectious Diseases (South San Francisco, CA, USA)) which was used to boost the ALVAC prime. The gp120 is not truncated, nor are any of the variable loops deleted, and it has shown to be immunogenic in human trials [15]. The Novartis TV1gp140ΔV2 is a subtype C gp140 with the V2 loop deleted, which has been shown to be immunogenic in animals, but not yet in humans, although a gp140 ΔV2 subtype B product has been evaluated in humans (see Sections 4.9 and 4.10 for more detail). The AIDSVAX product was used as a boost and administered concomitantly with ALVAC at months 4 and 5. The Novartis TV1gp140ΔV2 is used as a boost, with (group 4) or without (group 1) SAAVI MVA-C, and as part of a concurrent vaccination strategy with SAAVI MVA-C (group 2) (see study schema, Table 3-1). This evaluation of concurrent versus sequential protein boosting in combination with MVA-C may contribute to interpretation of RV144 data and to future HIV vaccine regimen design.

Although HVTN 086 / SAAVI 103 was inspired by the ALVAC/AIDSVAX trial, it does not include a study arm that mirrors the ALVAC/AIDSVAX vaccination strategy, nor does HVTN 086 / SAAVI 103 mirror the SAAVI DNA-C2/SAAVI MVA-C (HVTN 073/SAAVI 102) trial. Rather, HVTN 086 / SAAVI 103 evaluates different products (to the ALVAC/AIDSVAX trial) and vaccination strategies with the aim of building upon knowledge currently being gleaned from recent and ongoing trials, and adding to our knowledge of the best products and vaccination strategies.

The primary analysis of HVTN 086 / SAAVI 103 focuses upon a ranking strategy of various regimens that is guided by measurements of the immune responses elicited by these vaccines and their respective modes of delivery in various combinations, both sequential and concurrent. Data from these analyses will inform future trials, prospectively and/or adaptively. Certainly, safety data from this trial will inform later phase trials of these products and regimens. Furthermore, the immunogenicity data from this trial could significantly contribute to an analysis of correlates of protection identified in a later test-of-concept trial, for example, through retrospective analysis of those correlates in the regimens proposed in this earlier phase trial.

4.1.1 Rationale for mucosal secretion sampling

Mucosal immunity represents the initial defense against sexual transmission of HIV. One recent NHP study of intramuscular and intranasal administration of a protein subunit vaccine demonstrated protective immunity from vaginal challenge. Vaginal IgG and IgA demonstrated antiviral properties, though plasma IgG did not [16].While the vaccines in HVTN 086/SAAVI 103 are not being administered at mucosal sites, recent data in rhesus macaques demonstrated the ability of intramuscularly administered DNA prime-rAd5 simian immunodeficiency virus (SIV) boost vaccination regimens to elicit SIV-specific

CTLs in mucosal compartments [17,18] and lead to a reduction in the replication of SIVmac251 in gut lymphoid tissue as compared to controls [18]. These data suggest that systemic administration of HIV vaccines designed to induce HIV-specific T-cells can elicit cells that are effective in reducing HIV replication in the gut.

To further address the ability of vaccines given parenterally to elicit mucosal responses, participants enrolled at the Seattle HVTU in HVTN 069 participated in an ongoing study of mucosal responses. Of the nineteen participants enrolled who received the VRC DNA prime and rAd5 boost, potential T-cell epitope (PTE) Env-specific T-cell responses were seen in the blood of 18 (95%) participants. Of the 15 rectal specimens with sufficient cells for analysis at visit 7, 2 (13%) had PTE Env-specific T-cell responses. Although mucosal immune responses were detected less frequently than systemic immune responses, this appears to be the first preventative HIV vaccine clinical trial to show mucosal immune responses to an HIV vaccine.

Furthermore, the humoral responses anticipated with the gp140-containing regimens in HVTN 086/SAAVI 103 invite measurement of mucosal antibodies that may be generated following intramuscular administration of this envelope subunit vaccine. We will therefore measure production of HIV-specific IgA and IgG in mucosal secretions from vaccinees in this trial. Though the primary endpoints of this trial do not include mucosal immune responses, rectal and cervical secretions will be collected from willing volunteers at baseline (Month 0), when possible, and at the primary immunogenicity time point 2 weeks after the final vaccination, in order to evaluate changes in vaccine-induced immune responses at these mucosal sites.

4.2 SAAVI DNA-C2

The 2 SAAVI vaccines were designed for use in South Africa, which has a predominantly HIV-1 subtype C epidemic. The genes incorporated in the vaccine were derived from 2 primary HIV-1 subtype C strains, Du151 and Du422, which were selected based on their amino acid similarity to a derived South African consensus sequence [19] (see Table 4-2). This approach minimizes the genetic distance between the vaccine immunogen and circulating viruses. Samples were collected within 2 months of infection and thus may share some features with other transmitted variants. The amino acid sequences of proteins expressed by SAAVI DNA-C2 vaccine constructs are depicted in Figure 4-1.

Table 4-2 Description of the HIV-1 C genes and modification in SAAVI DNA-C2

Gene	Length Base pairs	HIV-1 Isolate	Modification and Rationale/Effect
gag	1326 bp	Du422	Mutated MGA to MAA (amino acid 2) to remove myristylation site. Codon optimized.
RT	1354 bp	Du151	Inactivated Reverse transcriptase (RT) by mutating YMDDL to YMAAL (amino acid 337). Codon optimized.
tat	365 bp	Consensus Du422/Du151	Inactivation of functional sites by shuffling and preserving potential CTL epitopes by repeat amino acid sequences. Codon optimized.
nef	592 bp	Du151	Inactivation of function by removal of myristylation site by deletion of 30 bp (5' end). Codon optimized.
env	2208	Du151	Deletion of 372 bases 3' end. Insertion of 10 amino acid Balb/C epitope originating from subtype B Env V3 sequence (RGPGRAFVTI) 3' end of gp150. Codon optimized.

A

1	**KLAT**MAARAS ILRGEKLDKW EKIRLRPGGK KHYMLKHIVW ASRELERFAL NPGLLETSEG	
61	CKQIMKQLQP ALQTGTEELK SLYNTVATLY CVHEKIEVRD TKEALDKIEE EQNKCQQKTQ	
121	QAKAADGKVS QNYPIVQNLQ GQMVHQAISP RTLNAWVKVI EEKAFSPEVI PMFTALSEGA	
181	TPQDLNTMLN TVGGHQAAMQ MLKDTINEEA AEWDRLHPVH AGPIAPGQMR EPRGSDIAGT	Gag
241	TSTLQEQIAW MTSNPPIPVG DIYKRWIILG LNKIVRMYSP VSILDIRQGP KEPFRDYVDR	
301	FFKTLRAEQA TQEVKNWMTD TLLVQNANPD CKTILRALGP GATLEEMMTA CQGVGGPGHK	
361	ARVLAEAMSQ TNSGNIMMQR SNFKGPRRIV KCFNCGKEGH IARNCRAPRK KGCWKCGKEG	
421	HQMKDCTERQ ANFLGKIWPS HKGRPGEFCG KKAIGTVLVG PTPVNIIGRN MLTQLGCTLN	
481	FPISPIETVP VKLKPGMDGP KVKQWPLTEV KIKALTAICE EMEKEGKITK IGPENPYNTP	
541	IFAIKKEDST KWRKLVDFRE LNKRTQDFWE VQLGIPHPAG LKKKKSVTVL DVGDAYFSVP	
601	LDEGFRKYTA FTIPSINNET PGIRYQYNVL PQGWKGSPAI FQASMTKILE PFRAKNPEIV	RT
661	IYQYMAALYV GSDLEIGQHR AKIEELREHL LKWGFTTPDK KHQKEPPFLW MGYELHPDKW	
721	TVQPIQLPEK DSWTVNDIQK LVGKLNWTSQ IYPGIKVRQL CKLLRGTKAL TDIVPLTEEA	
781	ELELAENREI LKEPVHGVYY DPSKDLIAEI QKQGDDQWTY QIYQEPFKNL KTGKYAKRRT	
841	THTNDVKQLT EAVQKISLES IVTWGKTPKF RLPIQKETWE IWWTDYWQAT WIPEWEFVNS	
901	GRKLATMVGI SYGRKKRRQR RSTPPSSEDH QNPISKQPLP QTRGDPTGSE ESKKKVESKT	Tat
961	KTDPFDCKYC SYHCLVCFQT KGLGISYGRK KRMEPIDPNL EPWNHPGSQP NTPCNKCYCK	
1021	YCSYHCLVVG WPAVRERIRR TEPAAEGVGP ASQDLDKHGA LTSSNTAHNN PDCAWLQAQE	
1081	EEEDVGFPVR PQVPLRPMTY KAAFDLSFFL KEKGGLEGLI HSKRRQDILD LWVYHTQGYF	
1141	PDWQNYTPGP GVRYPLTFGW CFKLVPVDPR EVEEANKGEN NCLLHPMSQH GMEDADREVL	Nef
1201	RWVFDSSLAR RHLAREKHPE YYKD***EFSR**	

B

1	MRVMGIQRNW PQWWIWGTLG FWMIIICRVV GNLNLWVTVY YGVPVWKEAK	
51	TTLFCASDAK AYDKEVHNVW ATHACVPTDP NPREIVLENV TENFNMWKND	
101	MVDQMHEDII SLWDQSLKPC VKLTPLCVTL NCTNAPAYNN SMHGEMKNCS	
151	FNTTTEIRDR KQKAYALFYK PDVVPLNRRE ENNGTGEYIL INCNSSTITQ	
201	ACPKVTFDPI PIHYCAPAGY AILKCNNKTF NGTGPCNNVS TVQCTHGIMP	
251	VVSTQLLLNG SLAEEEIIIR SENLTNNIKT IIVHLNKSVE IVCTRPNNNT	Gp150
301	RKSIRIGPGQ TFYATGEIIG NIREAHCNIS KSNWTSTLEQ VKKKLKEHYN	
351	KTIEFNPPSG GDLEVTTHSF NCRGEFFYCN TTKLFSNNSD SNNETITLPC	
401	KIKQIINMWQ KVGRAMYAPP IEGNITCKSN ITGLLLTRDG GKNTTNEIFR	
451	PGGGNMKDNW RSELYKYKVV EIEPLGVAPT KSKRRVVERE KRAVGLGAVL	
501	LGFLGAAGST MGAASITLTV QARQLLSGIV QQQSNLLRAI EAQQHMLQLT	
551	VWGIKQLQTR VLAIERYLKD QQLLGLWGCS GKIICTTAVP WNSSWSNKSQ	
601	EDIWDNMTWM QWDREISNYT GTIYRLLEDS QNQQEKNEKD LLALDSWKNL	
651	WNWFNITNWL WYIKIFIMIV GGLIGLRIIF GVLAIVKRVR QGYSPLSFQT	
701	LTPSPRGPDR LGRIEEEGGE QDKD**PRGPGR AFVTI***	

V3

Figure 4-1 Amino acid sequences of proteins expressed by SAAVI DNA-C2 vaccine constructs (A) GRTTN (kozak and restriction enzyme sequences bold and underlined): (B) gp150 with 10 amino acid Balb/C epitope originating from subtype B Env V3 sequence (RGPGRAFVTI) 3' end

SAAVI DNA-C2 is a multigene DNA vaccine consisting of 2 DNA plasmids, namely pVRCgrttnC, expressing an HIV-1 subtype C polyprotein comprised of Gag-Reverse Transcriptase-Tat-Nef (Grttn) and pVRCgp150CT, expressing an HIV-1 subtype C truncated Env (124 amino acids deleted from the carboxyl terminus) (Figure 4-2). Multiple genes were included in the vaccine in order to elicit broad immune responses and to avoid viral escape from vaccine-induced protective immune responses.

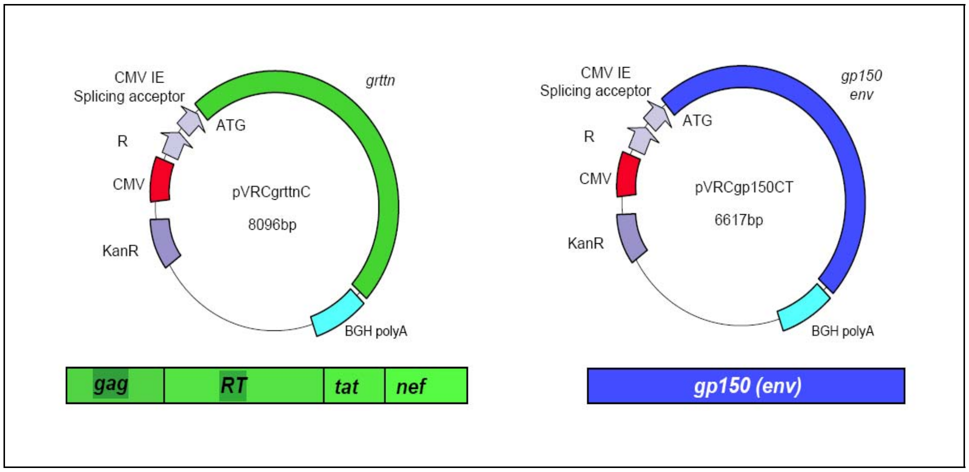

Figure 4-2 Plasmids making up SAAVI DNA-C2

All the genes in SAAVI DNA-C2 are optimized to reflect human codon usage. In addition, the genes have been modified for safety, increased expression, and stability. For improved levels of expression, HIV-1 gene codons were humanized and inhibitory sequence (INS) sites were removed. During synthesis, additional mutations and modifications were introduced for safety and increased immunogenicity.

4.3 SAAVI MVA-C

SAAVI MVA-C is a recombinant MVA vaccine containing the same 2 inserts as SAAVI DNA-C2 vaccine (*gag-RT-tat-nef* and *gp150CT*) in 2 insertion sites of the same MVA: grttnC, under the control of the vaccinia virus 40K promoter, is inserted into the Del III region, and gp150CT, under the control of the vaccinia virus I3 promoter, is inserted into the 49/50 region (Figure 4-3). The SAAVI MVA-C product was manufactured by Therion Biologics (Cambridge, MA, USA) under contract to NIAID. Prior to the closure of Therion, DAIDS transferred the responsibility for release and stability testing of the SAAVI MVA-C product to Advanced BioScience Laboratories, Inc. (Kensington, MD, USA).

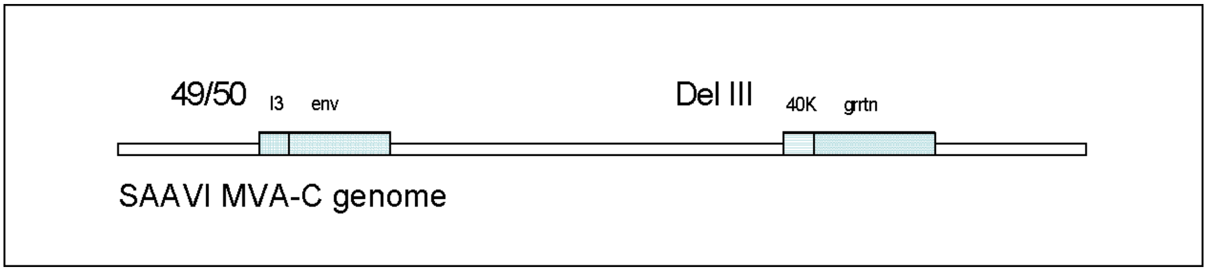

Figure 4-3 SAAVI MVA-C vaccine containing *env* (gp150CT) and *grttn*. The *env* gene is under the control of the I3 promoter in the 49/50 site and the *grttn* is under the control of the 40K promoter in the Del III site.

4.4 Novartis subtype C gp140

Novartis has pursued an Env-based HIV vaccine approach based on 2 important enhancements over those based on monomeric gp120 antigens explored so far, namely the stabilization of the Env immunogen in its more native trimeric state and deletion of the second hypervariable region (V2 loop) [20-25]. This approach relies on a series of observations suggesting that the V2-loop serves to mask conserved regions of the Env involved in viral entry and susceptibility to virus neutralization. On this basis, Novartis developed the subtype B and subtype C V2 loop-deleted Env protein boosts delivered in the MF59 adjuvant to stimulate nAb responses.

The subtype C ΔV2 Env antigen is a recombinant modified HIV envelope protein, 630 amino acids in length that is expressed in CHO cells (Figure 4-4).

```
  1 NTEDLWVTVYYGVPVWRDAKTTLFCASDAKAYETEVHNVWATHACVPTD
 50 PNPQEIVLGNVTENFNMWKNDMADQMHEDVISLWDQSLKPCVKLTPLCV
 99 TLNCTDTNVTGNRTVTGNSTNNTNGTGIYNIEEMKNCSFNAGAGRLINC
148 NTSTITQACPKVSFDPIPIHYCAPAGYAILKCNNKTFNGTGPCYNVSTV
197 QCTHGIKPVVSTQLLLNGSLAEEGIIIRSENLTENTKTIIVHLNESVEI
246 NCTRPNNNTRKSVRIGPGQAFYATNDVIGNIRQAHCNISTDRWNKTLQQ
295 VMKKLGEHFPNKTIQFKPHAGGDLEITMHSFNCRGEFFYCNTSNLFNST
344 YHSNNGTYKYNGNSSSPITLQCKIKQIVRMWQGVGQATYAPPIAGNITC
393 RSNITGILLTRDGGFNTTNNTETFRPGGGDMRDNWRSELYKYKVVEIKP
442 LGIAPTKAISSVVQSEKSAVGIGAVFLGFLGAAGSTMGAASITLTVQAR
491 QLLSGIVQQQSNLLKAIEAQQHMLQLTVWGIKQLQARVLAIERYLKDQQ
540 LLGIWGCSGRLICTTAVPWNSSWSNKSEKDIWDNMTWMQWDREISNYTG
589 LIYNLLEDSQNQQEKNEKDLLELDKWNNLWNWFDISNWPWYI
```

Figure 4-4 Amino acid sequence of the TV1gp140ΔV2 subtype C antigen predicted from DNA sequence

The protein is extensively purified following fermentation to remove cell culture medium, host cell related proteins and nucleic acids. In addition, the purification process includes steps specifically intended to reduce levels of adventitious virus through inactivation and /or removal. The theoretical monomer mass of TV1gp140ΔV2 subtype C antigen is 70422 Da based on the amino acid sequence. However, the TV1gp140ΔV2 subtype C antigen is extensively glycosylated during CHO intracellular processing

resulting in an apparent monomer mass of approximately 128 kDa. After purification, the antigen is composed primarily of dimeric and trimeric TV1gp140ΔV2 subtype C units.

4.5 Novartis MF59 adjuvant

MF59 adjuvant is an oil-in-water emulsion with a squalene internal oil phase and a citrate buffer external aqueous phase. Two non-ionic surfactants, sorbitan trioleate and polysorbate 80, serve to stabilise the emulsion. The bulk formula is shown in Table 4-3 below.

Table 4-3 Composition of MF59C.1 per liter

Name of Ingredients	Quantity per Litre [1]	Function	Reference to Standards
Squalene	39.0 g	oil phase	In-house specification
Polysorbate 80	4.7 g	surfactant	USP/NF
Sorbitan Trioleate	4.7 g	surfactant	USP/NF
Sodium Citrate, dihydrate	2.65 g	buffer	USP/NF
Citric Acid, monohydrate	0.17 g	buffer	USP/NF
Water for Injection	q.s 1 L	aqueous phase	Ph.Eur. and USP/NF
Nitrogen	overlay	inert gas	USP/NF

[1] An overage of up to 10% is included to compensate for manufacturing losses.

This adjuvant is used in the formulation of Fluad® trivalent inactivated influenza vaccine adjuvanted with MF59, produced in Siena/Rosia, Italy and licensed under the Mutual Recognition Procedure (IT/H/0104/001) in 12 EU countries with Italy as a Reference Member State (RMS) and Austria, Belgium, Denmark, France, Germany, Greece, Ireland, Luxembourg, Portugal, Spain, and Sweden as Concerned Member States (CMS). See the Investigator's Brochure (IB) for further detail.

4.6 Trial design rationale

HVTN 086 / SAAVI 103 evaluates T- and B-cell responses, with a focus on Ab responses, to SAAVI DNA-C2, SAAVI MVA-C and Novartis subtype C TV1gp140ΔV2 with MF59 adjuvant in various vaccination regimens. The primary analysis focuses upon characterizing the vaccine regimens, including identifying those that are superior to placebo in terms of the magnitude and breadth of the nAb responses they elicit to tier 1 and 2 pseudoviruses, followed by a ranking strategy of the regimens that is guided by measurements of the immune responses elicited by these vaccines and their respective modes of delivery in various combinations, both sequential and concurrent. Data from these analyses will inform future trials, prospectively and/or adaptively.

It is important to try to build upon the results of RV144, the ALVAC/AIDSVAX trial, with the aim of finding the optimal vaccination regimen that will induce cell-mediated immune responses and broadly nAb and binding Ab. The ALVAC/AIDSVAX trial included a B/E canarypox prime with a subtype E gp120 linked to subtype B tm gp41, and subtype B gag and protease (given at months 0, 1, 3 and 6) and boosted with a bivalent B/E gp120 (months 3 and 6) [15]. HVTN 086 / SAAVI 103 also evaluates the cellular and humoral immunogenicity of a pox prime (SAAVI MVA-C) followed by a protein boost (TV1gp140ΔV2), and concurrent administration of pox and protein. Furthermore, HVTN 086 / SAAVI 103 assesses the contribution of a DNA prime (SAAVI DNA-C2) to cellular and humoral responses when followed by a pox boost, with or without concurrent protein boost. This study may help inform efforts to interpret the ALVAC/AIDSVAX trial results, particularly the relative contribution of the protein versus the pox vaccines of the regimen and the roles of concurrent versus sequential protein and pox vaccinations.

4.6.1 Dose (amount and number)

SAAVI DNA-C2: 4mg per dose

SAAVI MVA-C: 1.45x10^9 pfu per dose

The justification for the dose is based upon preclinical data (see section 4.9) and clinical data from HVTN 073/SAAVI 102 (see section 4.10) and from HVTN 055. HVTN 055 tested different combinations of 2 vaccines based on MVA and FPV. There were 2 pairs of clinical grade vaccines with 1 set expressing Env and Gag and the other set expressing Tat, Rev, and Nef-RT based on a subtype B pediatric primary isolate. In HVTN 055, a 10^8 dose of MVA elicited more frequent responses to Gag and Pol than a 10^7 dose; and higher dosages of MVA vaccine primed a better response that could be boosted by the FPV vaccine. HVTN 055 also demonstrated that, based upon both IFN-γ and intracellular cytokine staining (ICS), FPV alone is poorly immunogenic, but it boosts well after MVA priming (refer to Table 4-4). Furthermore, a heterologous prime-boost elicited a greater response than either MVA or FPV alone in HVTN 055.

Table 4-4 HVTN 055: IFN-γ ELISpot Response Rates by Antigen, 2 weeks post fourth vaccination

	FPV	MVA (10^7) + FPV	MVA (10^8) + FPV	MVA (10^9) + FPV	MVA(10^9)
Any ENV	0/31 (0%)	5/9 (55.6%)	4/9 (44.4%)	9/26 (34.6%)	4/28 (14.3%)
Any GAG	2/31 (6.5%)	5/9 (55.6%)	7/9 (77.8%)	15/26 (57.7%)	11/28 (39.3%)
Any POL	0/31 (0%)	0/9 (0%)	2/9 (22.2%)	3/26 (11.5%)	2/28 (7.1%)

Novartis subtype C gp140: 100 mcg per vial

Preclinical studies using 100 mcg doses of the subtype B and C gp140 proteins in MF59 in NHP models have repeatedly demonstrated the immunogenicity and protective efficacy of this dosage when given as a single modality (protein) vaccine or when used in heterologous prime-boost regimens as described herein [22-24,26]. The 100 mcg dose was selected to facilitate comparison of results across studies [27]. Although extensive dose ranging has not yet been performed with these trimeric HIV Env proteins in humans, this dosage was initially selected based on previous clinical experience, including limited dose range studies performed with Novartis' subtype B SF2 gp120 and subtype E gp120 protein candidates, selected to be used at 50 mcg and 100 mcg doses, respectively, in the MF59 adjuvant [28-30].

4.6.2 Schedule

HVTN 073/SAAVI 102 evaluates the safety and immunogenicity of the SAAVI DNA-C2 prime (months 0, 1 and 2) followed by SAAVI MVA-C boost (months 4 and 5). A similar regimen is included in this protocol, HVTN 086 / SAAVI 103. However, in HVTN 086 / SAAVI 103 the SAAVI DNA-C2 prime is given only twice, at months 0 and 1, and the SAAVI MVA-C is given as both a prime at months 0 and 1 (group 1) or as a boost at months 3 and 6 (groups 3 and 4). Furthermore, HVTN 086 / SAAVI 103 includes an additional arm (group 2) in which SAAVI MVA-C is given concurrently with Novartis' TV1gp140ΔV2. Together with HVTN 073/SAAVI 102, this trial will provide insight into the optimal interval between the first and second SAAVI MVA-C vaccines, and the optimal number of DNA vaccinations to prime for a pox and/or protein boost.

Preclinical data support the plan for 2 doses of MVA (see Section 4.9), as do data from HVTN 055. In that study, marginal to no additional immune response was observed from greater than 2 doses of MVA (Table 4-5).

Table 4-5 HVTN 055: Optimal # of MVA doses by IFN-γ ELISpot (any antigen)

# of MVAs	+ responses	% + to > 1 Ag
x 2	19/44 (43.2%)	4/19 (21.1%)
x 3	8/29 (27.6%)	2/8 (25.0%)
x 4	13/28 (46.4%)	4/13 (30.7%)
x 5	6/26 (23.1%)	1/6 (16.7%)

HVTN 086 / SAAVI 103 also evaluates whether giving the SAAVI MVA-C vaccine and Novartis' TV1gp140ΔV2 vaccine, at the same time in different arms, induces similar nAb responses to those seen when the vaccines are given sequentially. This may contribute to ongoing assessments of the relative contribution of the concurrent pox-protein boost injections in RV144 and may inform efforts to design immunogenic and logistically feasible HIV vaccine regimens. This type of concurrent vaccine regimen was first described by Evans, et al [31], and was found to result in the earlier elicitation of virus nAb responses.

A 3 month window between the subtype C gp140 injections is proposed in light of evidence that this time frame may allow for more optimal B-cell response when compared with a narrower interval. High titers of Ab generally observed following adjuvanted protein boosts would be expected to form immune complexes with

subsequently administered protein vaccine, while a longer interval would allow Ab titers to decrease. Moreover, longer interval immunizations have been previously shown to be more effective at raising virus nAb responses with other subunit vaccines [27].

4.6.3 Prime-boost regimen

Three prime-boost strategies are evaluated:

- MVA prime with sequential gp140 boost (group 1)
- DNA prime with sequential MVA boost (group 3)
- DNA prime with concurrent MVA/gp140 boost (group 4)

One study group evaluates a concurrent SAAVI MVA/gp140 vaccination, given on two occasions three months apart (group 2). This vaccination strategy serves multiple purposes. It may contribute to an understanding of the relative role of this portion of the RV144 regimen on the results noted in that study and the role of this portion of the regimen proposed in group 4 of the present study. A comparison of groups 2 and 4 may also contribute to our understanding of the role of a DNA prime in advance of a co-administered pox-protein boost. A comparison of groups 2 and 1 may help distinguish between immune responses elicited by simultaneous vs. sequential administration of the same products. Groups 2, 3 and 4 together may help identify the relative contributions of the protein and the DNA in any immune responses seen in these arms. Furthermore, there are operational, adherence, and other potential advantages to a vaccine regimen that involves the fewest number of injections over the shortest span of time; in this context, exploration of simultaneous administration of multiple components of a regimen may add valuable knowledge to the field.

Both the SAAVI DNA-C2 and MVA-C vaccines include a truncated gp150, with a single nucleotide difference between the two. While SAAVI DNA-C2 priming has been demonstrated to enhance immunogenicity of SAAVI MVA-C boost over MVA alone (see Section 4.9.1), the value of adding a DNA prime to the MVA/gp140 vaccine boost in inducing broadly nAb or other immune response is unknown. The ALVAC/AIDSVAX trial did not utilize a DNA vaccine, whereas HVTN 086 / SAAVI 103 uses the SAAVI DNA-C2 vaccine as a prime followed by a boost with MVA alone (group 3) or with MVA in combination with gp140 (group 4).

Group 3 (DNA vaccine prime with sequential MVA vaccine boost) is designed in concert with group 4 to help distinguish the contribution of the DNA prime, versus the protein boost, in any immune response noted. Furthermore, in the effort to inform the development of the most logistically feasible, cost-effective, and immunogenic vaccine regimen, both groups 3 and 4 utilize 2 DNA primes instead of 3, as were utilized in some preclinical studies and in HVTN 073/SAAVI 102. Any immune responses seen in group 3 may be compared with those seen in studies utilizing 3 DNA primes, like HVTN 073/SAAVI 102, to further assess the optimum number of DNA primes.

As noted above, a comparison of DNA + MVA/gp140 vaccines (group 4) and MVA/gp140 vaccines alone (group 2) is designed to help further elucidate the potential contribution of a protein boost for an MVA vaccine that has been primed with a DNA vaccine. In light of the fact that a pox+protein vaccine strategy demonstrated modest

protective efficacy in RV144, and DNA+pox demonstrated immunogenicity in NHP and in early human results, it is of interest to investigate the potential of combining these regimens, as in group 4. The hope is that a DNA prime followed by a combined pox/protein boost will elicit greater depth and breadth of humoral and cellular immune responses than those seen with only 2 of the 3 components of the regimen; and a comparison of groups 2, 3, and 4 may help distinguish the individual contributions of each component to any immune responses elicited by the various regimens.

4.6.4 Choice of control

Sodium Chloride for Injection, 0.9% will serve as the placebo for the SAAVI DNA-C2 vaccine, SAAVI MVA-C vaccine and Novartis subtype C gp140/MF59 adjuvant. Sodium Chloride for Injection is nonreactogenic and well tolerated.

4.7 Plans for future product development and testing

HVTN 086 / SAAVI 103 evaluates vaccination strategies of DNA, MVA, and protein based vaccines with the aim of building upon knowledge currently being gleaned from recent and ongoing trials, and adding to our knowledge of the best products and vaccination strategies.

The primary analysis of this trial focuses upon a ranking strategy of the regimens, guided by measurements of the immune responses elicited by these vaccines and their respective modes of delivery in various combinations, including sequential and concurrent prime-boost. Data from these analyses will inform future trials, prospectively and/or adaptively. Safety data from HVTN 086 / SAAVI 103 will inform later phase trials of these products and regimens. Immunogenicity data from this trial could significantly contribute to an analysis of correlates of protection identified in a later test-of-concept trial, for example, through retrospective analysis of those correlates in the regimens proposed in this earlier phase trial.

4.8 Preclinical safety studies

Table 4-6 Summary of preclinical safety studies

Study number	Product	Type of study	Animal	N	Dose groups	Route	Schedule
Bridge GPS, Inc. Study No. 1195-06086	SAAVI DNA-C2<br>SAAVI MVA-C	Toxicity	New Zealand white rabbits	10m, 10f each group	SAAVI DNA-C2 vaccine (4mg in a split dose of 0.5 ml injections in each hind limb) and SAAVI MVA-C ($1.45x10^9$ pfu) as prime-boost combination and placebo/control group	IM	SAAVI DNA-C2 days 1, 15, 29, and 43; SAAVI MVA-C days 57, 71, and 85. Placebo received saline on same days.
Bridge GPS, Inc. Study No. 1195-06073	SAAVI DNA-C2	Bio-distribu-tion	New Zealand white rabbits	15f, 15m each group	SAAVI DNA-C2 group and control group, 4mg split into 4 injections of 0.5 mg in each hind limb	IM	4mg SAAVI DNA-C2 day 1. Controls inoculated with normal sterile saline on day 1. 5/gender/group sacrificed days 9, 60, and 90. Only positive tissues at day 60 were tested at day 90.
	TV1gp140ΔV2 +/- Tat		BALB/c mice	30	120 mcg (N=10)	ID	Single dose
					120 mcg (N=10, with Alum)	SC	Single dose
					120 mcg (N=10, SC with Alum, ID without Alum)	2xID, 2xSC	Repeated dose
	TV1gp140ΔV2, Subtype C DNA, Gag DNA, Pol DNA		Rhesus macaques	11	100 mcg (3 groups, TV1gp140ΔV2 with M59 following DNA prime)	IM	30 and 45 weeks

m = male
f = female

4.8.1 A repeat intramuscular (IM) dose toxicity study of SAAVI DNA-C2 and SAAVI MVA-C vaccines in the New Zealand white rabbit

Repeat IM injections with the SAAVI DNA-C2 and SAAVI MVA-C vaccines were generally well-tolerated and did not result in any toxicologically significant effects. The study evaluated the potential toxicity of SAAVI DNA-C2 and SAAVI MVA-C vaccines in the New Zealand white rabbit. Ten rabbits/sex/group were assigned to receive either saline or HIV vaccines. Vaccine-treated animals received IM injections of the SAAVI DNA-C2 vaccine (4 mg in a split dose of 0.5 ml injections in each hind limb) on study days (SDs) 1, 15, 29, and 43, and the SAAVI MVA-C vaccine ($1.45x10^9$ pfu) on SDs 57, 71, and 85. Control animals received IM injections of saline on all treatment days. Six control males, 4 control females, and 5 animals of each sex from the vaccine-treated animals were euthanized on SD 86, and all surviving animals were euthanized following a 14-day no-treatment recovery period on SD 100. All animals were subjected to a full gross necropsy. Parameters evaluated for all animals included mortality, clinical observations, dermal Draize observations, body weights, food consumption, ophthalmologic examination, clinical pathology, immunology, gross necropsy, organ weights, and histopathology.

Repeat IM injection with the SAAVI DNA-C2 and SAAVI MVA-C vaccines did not affect mortality, clinical observations, dermal Draize scores, body weights, food consumption, ophthalmology, gross pathology, or organ weights. All animals survived until scheduled termination with only incidental observations noted. Both body weights and food consumption remained comparable between control and vaccine-treated groups of both sexes throughout the course of study, and there were no test article-related findings at necropsy. IM administration of the vaccines resulted in minimally higher globulin concentration and minimally lower albumin to globulin ratio, consistent with an immunological response to the vaccine administration. On SD 100, all clinical chemistry parameters were considered to be normal. The longer activated partial thromboplastin time (aPTT) and higher fibrinogen concentration (FIB) values observed on SD 86 were considered to be a reflection of increased synthesis and release of inflammatory proteins from the liver due to the inflammatory response to the administered vaccine. Data for APTT and FIB on SD 100 were consistent with resolution of an acute inflammatory response. Immunology analysis showed moderate levels of seroconversion in vaccine-treated animals. Test-article related histologic findings were present in the injection sites, perineural connective tissue surrounding sciatic nerves, and skeletal muscle. In injection sites, inflammatory lesions existed both in control and vaccine-treated animals, but were more severe in vaccine-treated animals, suggesting that a component of the inflammatory response was due to the test article. Sciatic nerve and skeletal muscle lesions were present only in vaccine-treated animals. The severity of injection site lesions and sciatic nerve lesions was reduced in recovery-sacrifice animals, suggesting that the inflammatory response was resolving.

4.8.2 Biodistribution study of SAAVI DNA-C2 vaccine in New Zealand white rabbits

Upon analysis of the data generated in the biodistribution study, the US Food and Drug Administration (FDA) product review division determined that the amount of plasmid DNA observed in skin and muscle in New Zealand white rabbits is acceptable. Therefore, an integration study was not necessary. Biodistribution of SAAVI DNA-C2 in New

Zealand white rabbits was determined in a 90-day study conducted in compliance with Good Laboratory Practices (GLP) at Bridge Global Pharmaceutical Services (Gaithersburg, MD, USA). Results from the quantitative polymerase chain reaction (qPCR) analysis determined that the vaccine remained primarily localized in the muscle and skin at the site of injection, however plasmid was detected in the lung of 1 female animal at Day 9 and in the blood of 1 male animal at Day 60. Over the time course of the study, the copy numbers in the injection site decreased significantly demonstrating clearance over time.

Briefly, 15 animals/gender (11-15 week old New Zealand white rabbits) were inoculated IM into both thighs with 0.5 ml x 2 injections (total 1 ml/animal) containing 4 mg of plasmid DNA on SD 1. In addition, 15 animals/gender were inoculated with normal sterile saline also on SD 1. One third of the animals (5/gender/group) were sacrificed on SDs 9, 60, and 90. Tissues were collected and sent to Althea Technologies (San Diego, CA, USA) for PCR quantification. Only tissues positive at SD 60 were tested at SD 90. In addition, Althea performed dose verification by quantitating copy numbers of the 2 plasmids in the 4 mg dose. This analysis showed that the total number of copies was 3.06×10^{14} copies or 62% of the expected nominal dose (4.93×10^{14}copies). All animals survived to necropsy. No treatment-related observations were made with regard to clinical observations, body weights and changes, or food consumption.

A single dose of 4mg of SAAVI DNA-C2 vaccine did not affect mortality, clinical observations, body weights or food consumption. All animals survived to scheduled termination with only incidental observations noted. Random fluctuations in body weights and food consumption were noted during the course of study. Results of the qPCR analysis determined that the SAAVI DNA-C2 vaccine remained localized in the muscle and skin at the site of injection, and was not distributed to the blood or other organs of the body. Detection of the vaccine at the site of injection decreased in both the number of copies detected and percent of animals in which it was detected over the course of the study from SD 9 through 90.

4.8.3 Pre-Clinical evidence of responses to SAAVI MVA-C despite preimmunity to MVA

Preclinical data [32] demonstrate that there can still be responses to transgenes expressed by MVA despite preimmunity to the vectors. However, this may not be the same as immunity to replication competent vaccinia virus. One could expect the immune response to the replication competent vaccinia virus used to vaccinate against smallpox to be stronger and possibly have an even higher impact on immune responses to HIV protein than the replication incompetent vectors such as MVA or NYVAC.

The longevity of the immune response generated by a vaccine may be critical for its success, given that exposure to HIV may occur years after vaccination. In NHP studies with SAAVI DNA-C and SAAVI MVA-C [33] an immune response was boosted with a third SAAVI MVA vaccination (see Figure 4-5). Animals were inoculated bilaterally in the quadriceps muscle with 4 mg DNA 3 times, at 1 month intervals. Thirty-three weeks later, all animals were boosted with 10^9 pfu rMVA, given IM as two 1 mL inoculations. This was followed by a second rMVA boost 8 weeks later. To investigate the effect of further boosting of the immune response, a third MVA immunization was given to 4 animals at week 112. In all 4 animals, boosting induced responses that were higher than the previous peak responses. Mapping of individual peptide responses in DNA–MVA vaccinees revealed that this was not due to a broadening of the response, but in most

cases to a considerable increase in the magnitude of a single response that had dominated previously. For example, a single p24-peptide-specific response in animal 575 was responsible for approximately 90%, or 7993 net spot-forming units (SFU) per 10^6 PBMC, of the response. Interestingly, in 1 animal (549) an initially subdominant Nef response dominated after the third MVA dose, supporting a role for epitope competition for shared major histocompatibility complex (MHC) molecules. In contrast, in the single MVA-only animal tested (623), a third dose of MVA not only increased the magnitude of the response sixfold with respect to the previous peak response, but also considerably broadened the response, with an additional Gag and 2 Pol peptide pool responses being generated.

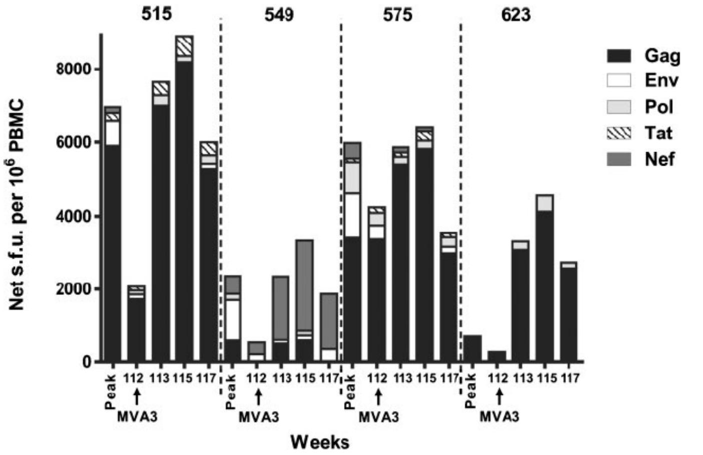

Figure 4-5 Vaccine memory persists and can be boosted. Four animals (515, 549, 575 and 623) received a third dose of MVA (10^9 pfu) at week 112, 1 year after the second MVA immunization. Cumulative IFN-γ ELISpot responses to peptide pools spanning Gag, Pol, Nef, Tat, and Env are shown, reported as net SFU. per 10^6 PBMC. Peak responses prior to the third immunization are shown, as well as responses on the day of immunization and the peak response thereafter, which occurred 3 weeks postvaccination in all animals.

4.8.4 Toxicity of TV1gp140ΔV2 in BALB/c mice

Toxicity of Single TV1gp140ΔV2 Inoculation in BALB/c Mice (conducted by Istituto Superiore di Sanità)

Acute toxicity was evaluated in a non-GLP study after a single injection of recombinant Tat protein (B cladeGMP grade) or TV1gp140ΔV2 protein (GMP grade) in BALB/c mice (n=45) after intradermal (ID) or subcutaneous (SC) inoculation (in the presence of Alum adjuvant for SC injection). After injection, mice were monitored daily for systemic and local effects. Systemic effects were monitored by evaluation of weight changes, alteration of vital signs and behavioral changes such as spontaneous activities, stereotypies or irritability. Local effects included evaluation of the appearance of induration, edema, papulae or the formation of angiogenic lesions at the site of injection. Two weeks after injection mice were sacrificed by cervical dislocation: the injection sites were evaluated for the presence of lesions and the internal organs examined for macroscopic modifications. All mice injected with Alum + buffer or + the various immunogens developed a visible granuloma at the site of injection approximately 4 days

after inoculation. No other local effects or altered systemic signs were observed throughout the experiment in all groups injected ID or SC with Tat + TV1gp140ΔV2, Tat alone, TV1gp140ΔV2 alone, and control animals.

Toxicity of Repeated TV1gp140ΔV2 Inoculations in BALB/c Mice (conducted by Istituto Superiore di Sanità)

The toxicity of repeated inoculations of the recombinant Tat (clade B) + TV1gp140ΔV2 proteins (subacute toxicity) was evaluated in mice. Mice were injected 2 times ID and 2 times SC (in the presence of Alum for SC injection). During vaccination mice were monitored for systemic signs and local effects. All mice injected SC with Alum alone or with the vaccine formulations + Alum developed a visible granuloma at the site a few days after the first inoculation. Histological examination of the site of injection of some mice showed a similar inflammatory reaction in vaccinated and control animals in agreement with the observation of a visible granuloma at the site of injection in all injected mice. No other local effects were reported during the experiments. No systemic effects were observed in vaccinated mice as compared to controls. Histological examination of some animals organs (liver, kidney, intestine, heart, lungs, brain, lymph nodes) did not show abnormalities that may be related to the vaccine formulations.

4.8.5 Toxicity of TV1gp140ΔV2 in rabbits

New Zealand white rabbits were injected 2-3 times IM with Env (GLP grade) in the presence of the MF59 adjuvant with or without priming immunizations with DNA. No specific local effects or altered systemic signs that might be related to the immunogens were observed throughout the experiment or at sacrifice in all groups. All vaccine regimens tested proved safe in rabbits with no specific systemic or local effects reported in any of the rabbits. Daily health monitoring was performed. There were no abnormalities in behaviour or clinical signs with regards to the rabbits in these studies.

4.8.6 Toxicity of TV1gp140ΔV2 in macaques

Subacute toxicity was evaluated in rhesus macaques (*Macaca mulatta*) (n=11) by injecting IM the TV1gp140ΔV2 immunogen (subtype C) in combination with Gag and Pol (each subtype C) in a DNA prime/protein boost regimen. Testing revealed no local effects, abnormalities or alteration of vital signs, or behavioral changes that may be related to the vaccine formulation.

4.8.7 Toxicity of MF59

Pivotal toxicology studies performed with MF59 include the evaluation of single- and repeat-dose toxicity (including local tolerability), genotoxicity, sensitization, and embryofetal and developmental toxicity. MF59 is not associated with any potential for systemic toxicity and it has a low order of local reactogenicity. In repeat-dose rabbit studies, clinical pathology findings of increased fibrinogen and minor inflammatory and degenerative changes at the injection site are consistent with the effects of intramuscular injections of an immunological adjuvant. These findings are readily reversible within days to 1 to 2 weeks. In repeat-dose toxicology studies in dogs, there were no effects on cardiovascular or central nervous system (safety pharmacology) parameters. MF59 is not genotoxic (Ames test) or clastogenic (mouse micronucleus), is not a dermal sensitizer (Guinea pig), and was not teratogenic (rat and rabbit) or a developmental toxicant (rat).

4.9 Preclinical immunogenicity studies

Table 4-7 Summary of preclinical immunogenicity studies (SAAVI)

Product	Animal	N	Dose groups	Route	Schedule
SAAVI DNA-C2 and SAAVI MVA-C	Rhesus macaques	6-8 per group	4mg DNA-C2 10^9 pfu MVA-C	IM	8 received DNA-C2 at months 0, 1, and 2 followed by MVA-C at months 4 and 5<br>6 received MVA-C only at months 4 and 5
SAAVI DNA-C and SAAVI MVA-C	Chacma baboons	3-6 per group	4mg DNA-C 10^9 pfu MVA-C	IM	6 received DNA-C at 0, 1, and 2 months<br>MVA-C at 10 and 12 months<br>3 received MVA-C only at months 10 and 12

Table 4-8 Summary of preclinical immunogenicity studies with TV1 Env proteins (Novartis)

Study Number	Product	Animal	N	Dose groups	Route	Schedule
04-0114	TV1gp140ΔV2<br>TV1gp120	New Zealand white rabbit	4 per group	50 mcg	IM	0, 7, and 17 weeks
01-0114	TV1gp140ΔV2<br>TV1gp140<br>TV1gp140ΔV1V2	New Zealand white rabbit	4 per group	50 mcg	IM	20, 47, and 59 weeks
775-MM-2	TV1gp140ΔV2	Rhesus macaques	3-4 per group	100 mcg	IM	30 and 45 weeks
04-0359	TV1gp140ΔV2	New Zealand white rabbit	10 per group	25 mcg	IM	0, 4, 12, and 24 weeks

4.9.1 Immunogenicity of SAAVI DNA-C2 and SAAVI MVA-C in rhesus macaques

An immunogenicity study was undertaken in Chinese rhesus macaques (*Macaca mulatta)* using the clinical good manufacturing practice (cGMP) lots of SAAVI DNA-C2 and SAAVI MVA-C. Eight female rhesus macaques were immunized IM with SAAVI DNA-C2 and SAAVI MVA-C according to the vaccination regimen described in Table 4-7. Animals received 3 doses of 4 mg SAAVI DNA-C2 1 month apart, and 8 weeks later received 10^9 pfu SAAVI MVA-C. A further 6 animals received 2 doses of 10^9 pfu

SAAVI MVA-C only, 1 month apart. Fresh PBMC were tested in an IFN-γ ELISpot assay for responses to peptide pools based on the vaccine-expressed HIV antigens. ICS was performed on cryopreserved PBMC using the same peptide pools.

T-cell responses by ELISpot: In rhesus macaques that received the SAAVI MVA-C vaccine only, 2 out of 6 responded to the vaccine (Figure 4-6A, right panel). Responses were directed at single proteins, with 1 animal responding to Nef (403 net SFU/10^6 PBMC) and the second responding to Env (334 net SFU/10^6 PBMC) after 2 doses of SAAVI MVA-C. However, in the animals that had been primed with SAAVI DNA-C2 prior to SAAVI MVA-C boosting, broad, high magnitude responses were detectable after SAAVI MVA-C vaccine boosting (Figure 4-6 A, left panel). Eight out of 8 rhesus macaques that had been primed with SAAVI DNA-C2 responded to the vaccine regimen after boosting with SAAVI MVA-C vaccine. The median response magnitude was 1118 net SFU/10^6 PBMC (range 329 to 9405 net SFU/10^6 PBMC). The animal with the highest cumulative response magnitude responded to all 5 HIV proteins in the vaccines.

IFN-γ ELISpot responses were monitored longitudinally (Figure 4-6 B). Immune responses peaked 1 week after the first immunization with SAAVI MVA-C vaccine in 7 out of 8 animals that responded to immunization with SAAVI DNA-C2 and SAAVI MVA-C, after which they contracted dramatically. Immune responses were detected against all the HIV proteins expressed by the vaccines (Figure 4-6 C). Responses to Gag and Nef were of the highest frequency, with 8 out of 8 rhesus macaques responding to Gag, 7 out of 8 to Nef, 6 out of 8 to Pol, 5 out of 8 to Env and 1 out of 8 to Tat. Nef had the highest median response, at 232 net SFU/10^6 PBMC.

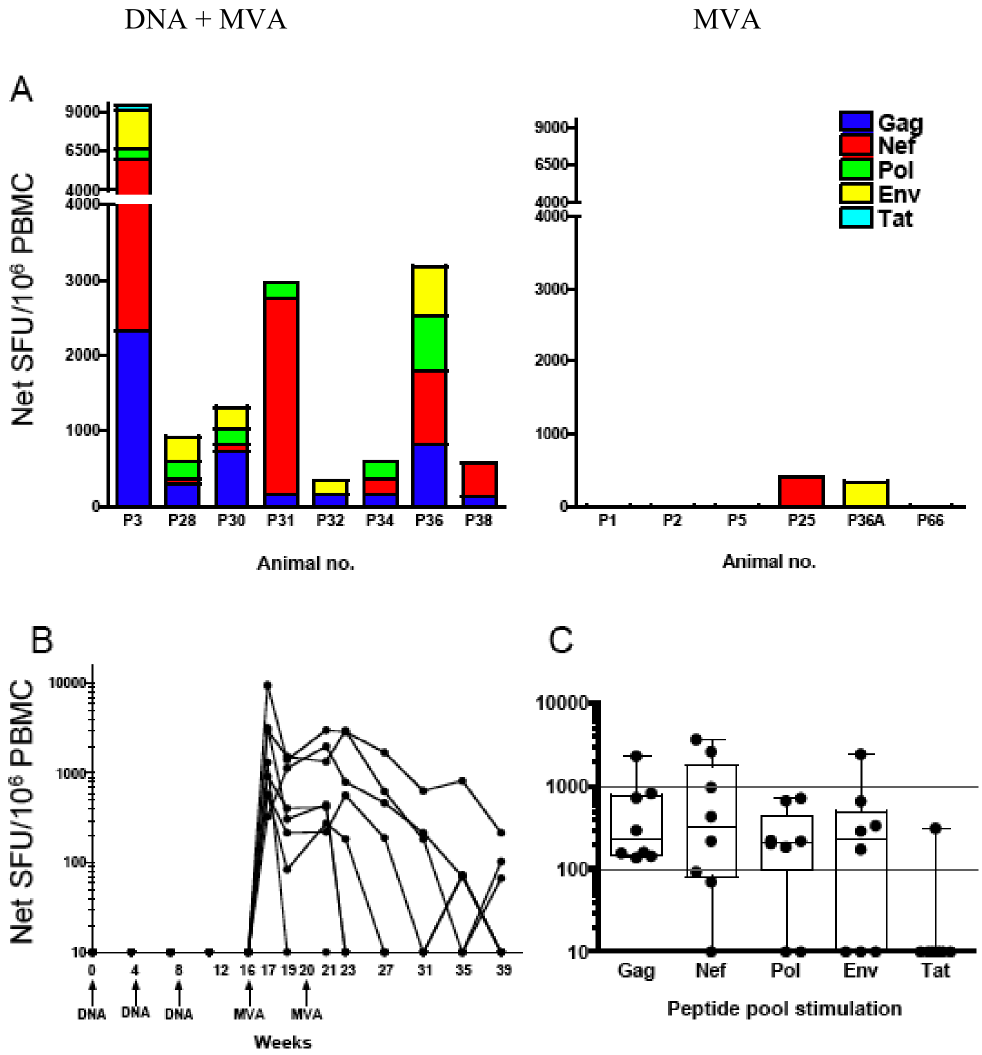

Figure 4-6 Immunogenicity of vaccine regimen after SAAVI MVA-C boosting in rhesus macaques. IFN-γ ELISpot responses in fresh PBMC to 5 peptide pools based on the HIV antigens expressed by the vaccines were measured. (A) ELISpot responses for the 8 rhesus macaques that had previously been primed with SAAVI DNA-C2 (left panel), and 6 rhesus macaques that received SAAVI MVA-C only (right panel). (B) Kinetics of ELISpot responses over time in rhesus macaques immunized with SAAVI DNA-C2 and SAAVI MVA-C. (C) Median responses to vaccine antigens in SAAVI DNA-C2/SAAVI MVA-C immunized rhesus macaques. Median and interquartile ranges are presented. Responses are expressed as net SFU/10^6 PBMC.

Polyfunctional T-cell responses by ICS in rhesus macaques: Six rhesus macaques were tested for T-cell responses using ICS to detect IFN-γ, IL-2 and TNF-α in response to vaccine antigens. Rhesus macaques vaccinated with SAAVI DNA-C2 and SAAVI MVA-C elicited both CD4+ and CD8+ T-cell responses (Figure 4-7 A), with CD4+ responses predominating. Median cytokine response magnitudes per animal for CD4+ T cells were > 1% for IFN-γ and IL-2 (Figure 4-7 B). Polyfunctional T cells expressing combinations of cytokines IFN-γ, IL-2 and TNF-α were also detected (Figure 4-7 C). Thus, the combination of SAAVI DNA-C2 and SAAVI MVA-C immunization induced

high magnitude, polyfunctional CD8+ and CD4+ T-cell responses in rhesus macaques targeting multiple HIV antigens.

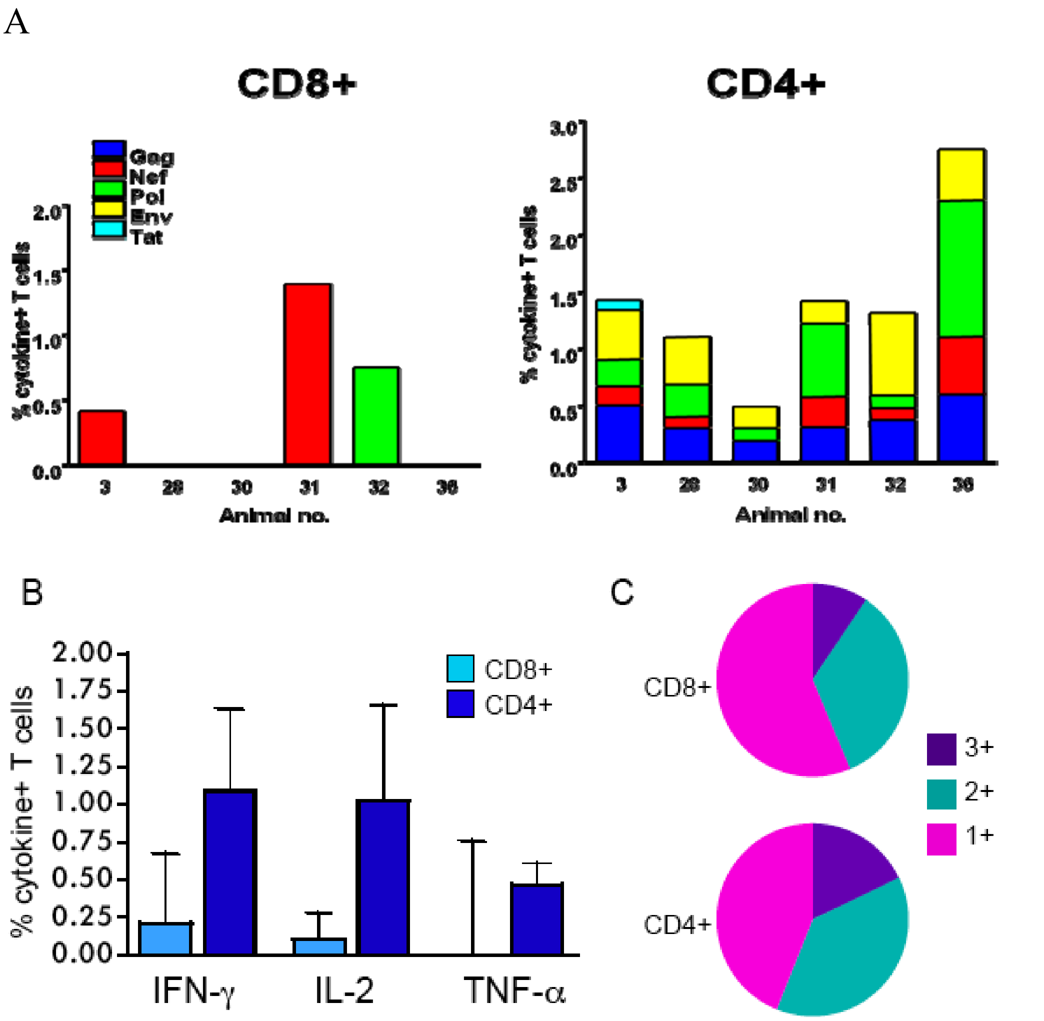

Figure 4-7 Cytokine responses from CD8+ and CD4+ T cells induced by SAAVI DNA-C2 and SAAVI MVA-C vaccination in rhesus macaques. HIV-specific T-cell responses were detected using multiparameter flow cytometry. Shown are the magnitude of CD8+ and CD4+ cytokine+ T cells per animal (A); median IFN-γ, IL-2 and TNF-α, responses (B), and the proportion of cells simultaneously secreting 3, 2 or 1 cytokines (C).

Humoral immunity elicited in response to SAAVI DNA-C2 and SAAVI MVA-C immunization in rhesus macaques: In order to investigate whether the SAAVI DNA-C2 and SAAVI MVA-C vaccines elicited an Ab response to HIV Env, sera from vaccinated rhesus macaques were tested for the presence of antibodies to HIV-1 subtype C gp120 by enzyme-linked immunosorbent assay (ELISA). Binding antibodies to gp120 were detectable in 8/8 rhesus macaques that received both SAAVI DNA-C2 and SAAVI MVA-C vaccines (Figure 4-8 A and B), but did not develop in rhesus macaques that received SAAVI MVA-C only. Titers ranged from 10^2 to 10^5.

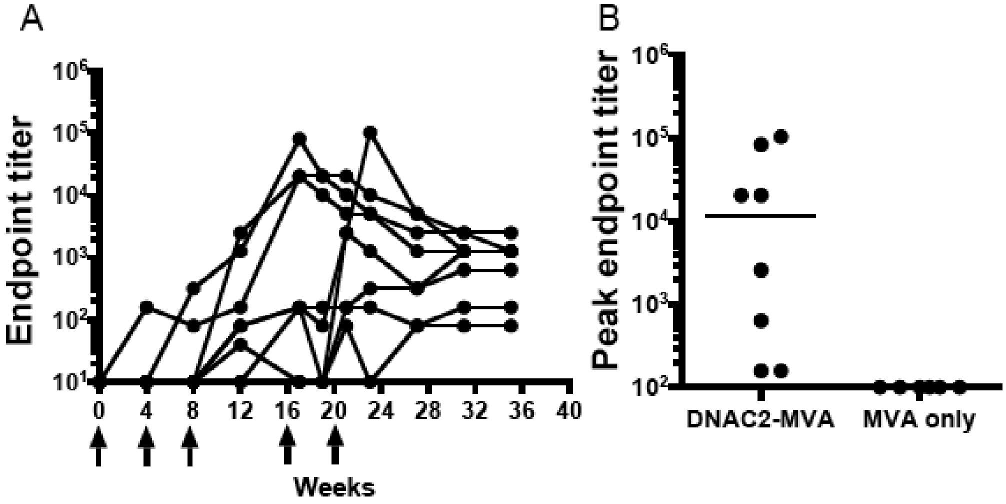

Figure 4-8 Ab responses to HIV gp120 in rhesus macaques immunized with SAAVI DNA-C2 and SAAVI MVA-C. (A) Sera from rhesus macaques immunized with SAAVI DNA-C2 and SAAVI MVA-C were tested for Ab responses to HIV-1 subtype C gp120. Immunizations are indicated by arrows. (B) Peak Ab titers for rhesus macaques immunized with SAAVI DNA-C2 and SAAVI MVA-C and SAAVI MVA-C-only. No responses were detected in animals that received SAAVI MVA-C vaccine-only. Ab responses were evaluated by ELISA and values represent endpoint serum titers.

Summary: All 8 rhesus macaques that had been primed with SAAVI DNA-C2 and boosted with SAAVI MVA-C responded to the vaccine regimen after the MVA boost. IFN-γ T-cell responses were broadly-directed and of high magnitude. Responses were present to all vaccine-expressed proteins, with the highest frequency responses to Gag and Nef. Whilst no detectable T-cell responses were present after DNA immunizations, DNA did appear to have a priming effect, since the macaques which had received MVA only in the absence of a DNA prime had low frequency and narrowly-directed T-cell responses. Responses were mainly CD4-mediated, and polyfunctional in nature, producing IFN-γ, IL-2 and TNF-α.

These results are comparable to the magnitude and breadth of responses elicited in *chacma* baboons with an earlier generation of the DNA vaccine (SAAVI DNA-C) and the research lot of SAAVI MVA-C (see section 4.9.2). Whilst individual ELISpot responses in rhesus macaques have not been mapped beyond the peptide pool level, there is some indication that responses generated are broad, as evidenced from immunogenicity studies carried out in baboons (see section4.9.2) [32].

4.9.2 Immunogenicity of SAAVI DNA-C2 and SAAVI MVA–C in *chacma* baboons

IFN-γ ELISpot responses to SAAVI DNA-C and SAAVI MVA-C vaccines in *chacma* baboons: As part of the initial preclinical studies, an earlier generation vaccine, SAAVI DNA-C (Cobra Biomanufacturing, Keele, Staffordshire, UK), which expresses gene inserts identical to those in SAAVI DNA-C2 on a different plasmid backbone [34], was tested in *chacma* baboons. SAAVI DNA-C vaccine consists of 2 plasmids, pTHr.grttnC and pTHr.gp150CT, in an equimolar ratio. The levels of expression of SAAVI DNA-C and SAAVI DNA-C2 are similar as assessed by Western blot, and there is no significant difference in immunogenicity between the constructs tested in BALB/c mice.

In this study, 6 baboons were vaccinated with 3 doses of 4 mg SAAVI DNA-C given at 1 month intervals, and were boosted with 10^9 pfu SAAVI MVA-C at 8 and 10 months after the last SAAVI DNA-C vaccine. Three animals were vaccinated with 10^9 pfu SAAVI MVA-C only, given at a 2 month interval, and all 3 demonstrated T-cell responses. Five out of the 6 baboons that received the SAAVI DNA-C immunizations followed by SAAVI MVA-C boosts generated T-cell responses to the vaccines. Furthermore, 4 out of the 5 responders recognized Gag, RT, Env, Nef, whilst 1 responded to all 5 vaccine-expressed proteins (Gag, RT, Env, Nef, Tat) (Figure 4-9). ICS by flow cytometry indicated that all 5 SAAVI DNA-C/SAAVI MVA-C responders developed antigen specific CD8+ and CD4+ T cells that produced IFN-γ and IL-2 following peptide stimulation. Each of the responders also developed a small fraction of dual cytokine (IFN-γ+IL-2+)-expressing cells specific for the vaccine antigens. The SAAVI MVA-C-only group exhibited narrow responses, with responses detected only to a single Gag or Env peptide pool. There were significant differences between the cumulative median response magnitudes of SAAVI DNA-C/SAAVI MVA-C responders (4103 SFU/10^6 PBMC) and SAAVI MVA-C-only animals (213 SFU/10^6 PBMC) as measured by the IFN-γ ELISpot assay.

Results in baboons receiving these vaccines compared favorably with the magnitude and breadth of the T-cell response detected by IFN-γ ELISpot assays in macaques using the cGMP manufactured SAAVI DNA-C2 and SAAVI MVA-C.

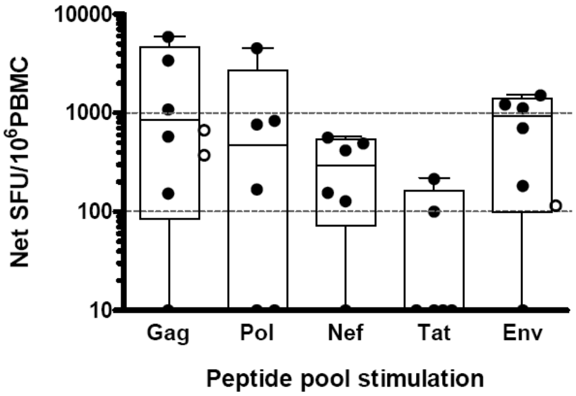

Figure 4-9 IFN-γ ELISpot responses in vaccinated *chacma* baboons. Cumulative IFN-γ ELISpot responses to peptide pools spanning Gag, Pol, Nef, Tat and Env are reported as net SFU/10^6 PBMC at peak timepoints. Medians, interquartile ranges and ranges are represented for SAAVI DNA-C/SAAVI MVA-C vaccinated animals. Filled symbols represent SAAVI DNA-C/SAAVI MVA-C vaccinated animals, and open symbols depict animals that received SAAVI MVA-C only.

Ab responses to gp120 in *chacma* baboons: SAAVI DNA-C and SAAVI MVA-C elicited an Ab response to HIV Env in vaccinated baboons. Binding antibodies to HIV-1 subtype C gp120 were detectable by ELISA in sera from all baboons that received both SAAVI DNA-C and SAAVI MVA-C (Figure 4-10). Endpoint Ab titers present in 5 out of 6 animals after DNA immunization were boosted 12 fold after SAAVI MVA-C immunization, and peak endpoint titers ranged from 80 to 5160. Weak and transient Ab responses were detectable in only 1 of the 3 animals that received SAAVI MVA-C only. No neutralizing activity was detected using a pseudovirion neutralization assay.

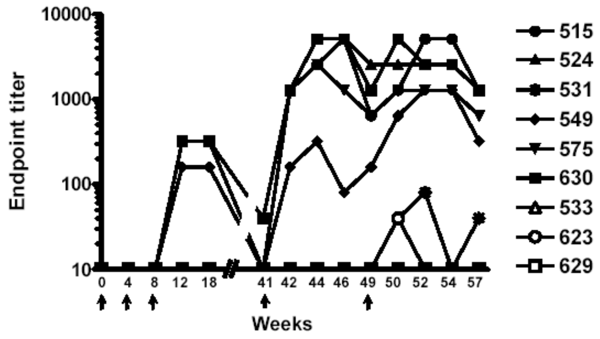

Figure 4-10 Ab responses to HIV gp120 Sera from baboons immunized with SAAVI DNA-C and SAAVI MVA-C (filled symbols) or SAAVI MVA-C alone (open symbols) were tested for Ab responses to gp120. Immunizations are indicated by arrows. Ab responses to HIV-1 subtype C gp120 were evaluated by ELISA and values represent endpoint serum titers.

4.9.3 Immunogenicity of TV1gp140ΔV2, study 04-0114

To evaluate the immunogenic potential of modified and trimeric envelope protein as compared to unmodified monomeric envelope, rabbits were immunized with either V2-deleted oligomeric gp140 or intact monomeric gp120 envelope proteins derived from either subtype C TV1 or subtype B SF162 (Table 4-9). Immunizations were carried out at weeks 0, 7, and 17. Animals were evaluated for the induction of serum nAb against HIV-1 SF162 and TV1 isolates.

Table 4-9 Immunization with oligomeric or monomeric envelope protein

Group	Imm'n #	Adjuvant	Immunogen	Total Dose	Vol/ Site	Sites/ Animal	Route
**1**	1, 2, 3	MF59	gp140ΔV2 SF162	50 mcg	0.5 ml	2	IM/Glut
**2**	1, 2, 3	MF59	gp120 SF162	50 mcg	0.5 ml	2	IM/Glut
**3**	1, 2, 3	MF59	TV1gp140ΔV2	50 mcg	0.5 ml	2	IM/Glut
**4**	1, 2, 3	MF59	gp120 TV1	50 mcg	0.5 ml	2	IM/Glut

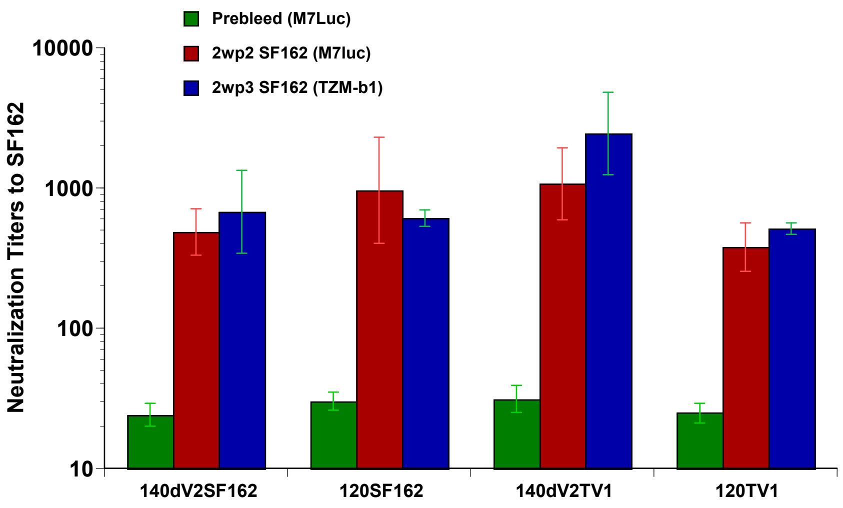

Figure 4-11 Neutralizing activity against subtype B HIV-1 SF162. Values represent the geometric mean titers of the reciprocal dilution of sera needed to neutralize 50% of the activity of SF162 in the M7luc or TZM-bl assay. Timepoints shown are prebleed and 2 weeks post third (2wp3) protein administration. Standard error is indicated.

All groups showed potent neutralizing activity against the HIV-1 isolate SF162 following the second protein immunization (Figure 4-11). With the exception of the group immunized with gp120 SF162, neutralization titers increased with an additional third protein administration. The TV1gp140ΔV2 immunized group had the highest neutralizing titer among all groups at 2 weeks after the third immunization.

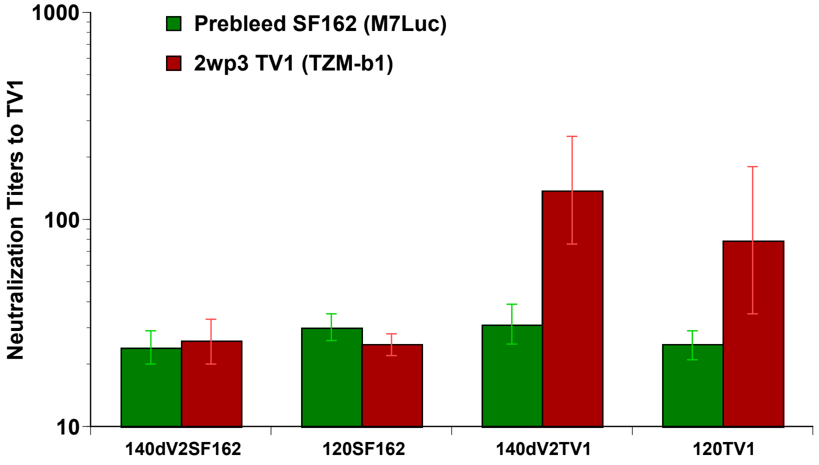

Figure 4-12 Neutralizing activity against subtype C HIV-1 TV1: Values represent the geometric mean titers of the reciprocal dilution of sera needed to neutralize 50% of the activity of TV1 in the TZM-bl assay. Timepoints shown are prebleed and 2 weeks post third (2wp3) protein administration. Standard error is indicated.

Neutralization against the HIV-1 subtype C TV1 strain was only seen in groups immunized with the homologous TV1 immunogens and not in those receiving the SF162 antigen (Figure 4-12). Following the third protein immunization, the group which

received TV1gp140ΔV2 yielded higher TV1 neutralization titers than the group receiving monomeric gp120 TV1.

4.9.4 Immunogenicity of TV1gp140ΔV2 in rabbits, study 01-0114

To determine the optimal immunogenic form of TV1 envelope, gene cassettes were generated that deleted the variable region V2 (ΔV2) separately or in combination with V1 (ΔV1V2). These modified constructs were then used in a DNA prime/protein boost vaccine regimen in rabbits to test for their capacity to elicit nAb as listed in Table 4-10.

Table 4-10 Rabbit immunization groups

Category	Group	Plasmid DNA Prime Weeks 0, 4, & 20	Protein Boost Weeks 20, 49, & 59
Subtype B gp140	1	pCMVgp140ΔV2.SF162	gp140ΔV2 SF162
Subtype C gp160	2	pCMVgp160.TV1	gp140 TV1
	3	pCMVgp160ΔV2.TV1	TV1gp140ΔV2
	4	pCMVgp160ΔV1V2.TV1	gp140ΔV1V2 TV1
Subtype C gp140	5	pCMVgp140.TV1	gp140 TV1
	6	pCMVgp140ΔV2.TV1	TV1gp140ΔV2
	7	pCMVgp140ΔV1V2.TV1	gp140ΔV1V2 TV1
Combined Subtype B + C	8	pCMVgp140ΔV2.TV1<br>pCMVgp140ΔV2.SF162	TV1gp140ΔV2<br>gp140ΔV2 SF162
	9	pCMVgp140ΔV2.TV1<br>pCMVgp140ΔV1V2.SF162	gp140ΔV1V2 TV1<br>gp140ΔV2 SF162

The potency of homologous nAb responses elicited by the various TV1 vaccine constructs was first compared. To check for the neutralization of a heterologous subtype B isolate, and because some groups also received SF162 envelopes, both TV1 and SF162 were included in this nAb potency comparison. All prime/boosted animals, including those immunized with only TV1 immunogens, developed significant nAb responses against the neutralization-sensitive SF162 strain (Figure 4-13). Two significant differences in neutralization titers against SF162 were observed 2 weeks after the second protein boost (2wp4). First, animals primed with the subtype C TV1gp140 forms had higher titers of SF162 nAb than those primed with the gp160 forms (groups 5, 6, and 7 versus groups 2, 3, and 4, P=0.0102, t test). Not surprisingly, animals primed and boosted with the gp140ΔV2 SF162 form had higher nAb titers to the homologous subtype B strain than those primed and boosted with TV1gp140ΔV2 (group 1 versus group 6, P=0.0134, t test). Interestingly, rabbits immunized with the combined V2-modified SF162 and TV1 immunogens (groups 8 and 9) showed indications of higher nAb titers against SF162 at 2wp4 than those immunized with the modified TV1 immunogens alone (groups 6 and 7) (P=0.0532, t test). However, following the third protein boost (2wp5), the neutralization titer toward SF162 remained at a level similar to that after the second protein (P=0.4121, t test), and no significant differences were observed among groups (P=0.0550, ANOVA).

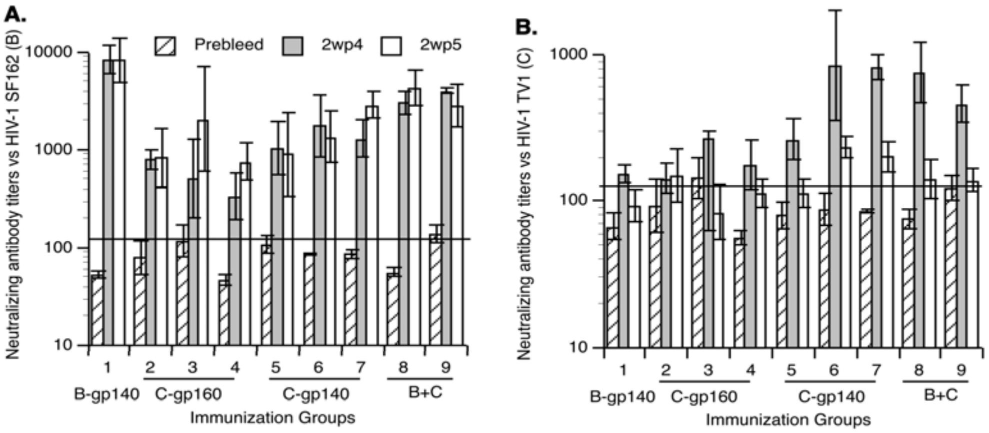

Figure 4-13 nAb titers after protein immunizations (study 01-0114): Virus neutralization assays were performed using the M7luc assay. Titers are the reciprocal dilution of sera needed at which RLU were reduced 50% compared to virus control wells for SF162 (A) or TV1 (B). Group numbers are as in **Table 4-10**. Pre-bleed and 2 weeks post the second (2wp4) and third protein boosts (2wp5) are shown. Standard error is indicated.

Several significant differences in the potency of TV1 nAb (Figure 4-13 B) were also noted. Two weeks after the second protein boost (2wp4), priming with gp140 TV1 elicited higher nAb titers than priming with gp160 TV1 immunogens (groups 5, 6, and 7 versus 2, 3 and 4, P=0.0025, t test). Compared to group 1 animals, the subtype C TV1gp140ΔV2 rabbits (group 6) had higher nAb titers to the homologous TV1 strain (P=0.0073, t test). Priming with a combination of ΔV2 gp140s from both subtypes (group 8) elicited higher homologous nAb titers against the TV1 strain than priming with the subtype B gp140 alone (group 1) (P=0.0067, t test). Immunizing with TV1gp140ΔV2, as well as gp140ΔV1V2 TV1, yielded higher titers of homologous TV1 neutralization than the intact gp140 TV1 (Figure 4-13 B; group 5 versus group 6, P=0.0548; group 5 versus group 7, P=0.0598 [t test]). However, following the third protein boost (2wp5), TV1 nAb were significantly reduced ($P < 0.0001$, t test), although the difference between the gp140 and gp160 forms remained (groups 5, 6, and 7 versus groups 2, 3, and 4, P=0.0384, t test). Importantly, TV1gp140ΔV2 showed significantly higher titers of homologous neutralization than the intact gp140 TV1 (2wp5, Figure 4-13 B; group 5 versus group 6, P=0.0491, t test).

In some cases, the contribution of DNA priming to the nAb activity against SF162ΔV2 and against the closely related heterologous subtype C TV2 strain was able to be assessed. In these experiments, HIV neutralization was analyzed in sera collected 2 weeks after the second DNA immunization, prior to the animals receiving any protein immunizations. All 4 animals in group 6, those primed with subtype C TV1gp140ΔV2 DNA, had significant serum nAb activity against the neutralization-sensitive SF162ΔV2 strain (Figure 4-14 A) and against the heterologous South African TV2 strain (Figure 4-14 B). Among the gp140 groups, priming with TV1gp140ΔV2 (group 6) was the most effective at eliciting nAb activity against TV2 (group 6 versus groups 5 and 7 combined values, P=0.0333 [Kruskal-Wallis test]).

In summary, the TV1gp140ΔV2 protein was found to elicit virus nAb responses when used as a boost in DNA-primed animals. In addition, results support the use of the TV1gp140ΔV2 protein alone with adjuvant (details provided in IB and [35]).

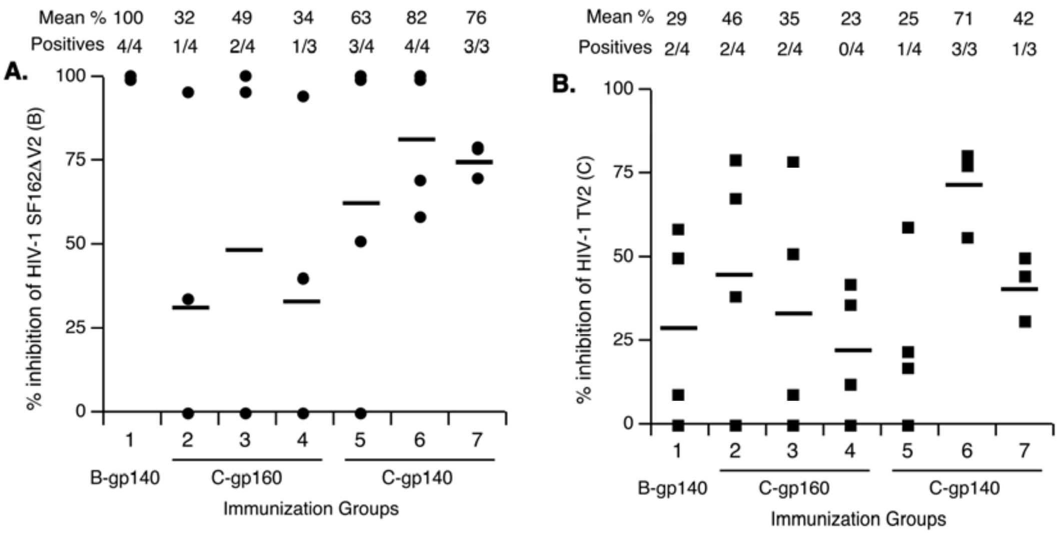

Figure 4-14 nAb activity in sera after priming with DNA: Experiments were performed using a GFP-reporter cell assay. The percent virus inhibition indicates the percent reduction in GFP-expressing cells at a 1:20 serum dilution. Neutralization against SF162 ΔV2 (A) and TV2 (B) at 2 weeks after the second DNA immunization.

4.9.5 Immunogenicity of TV1gp140ΔV2 in macaques, study 775-MM-2

To determine whether the proposed TV1gp140ΔV2 vaccines were immunogenic in NHP, rhesus macaques were immunized with a HIV subtype C DNA prime protein boost regimen. DNA vaccines encoding TV1gp140ΔV2 were tested in combination with DNA vaccines encoding subtype C-derived *gag* and *pol* in rhesus macaques (Macaca mulatta). Three groups of macaques were included in this NHP study. Group 1 animals (n = 4) received 3 immunizations with 2 mg of pCMVgp140ΔV2.TV1 and 3.5 mg of pCMV*gag*SF2 at 0, 4, and 8 weeks, followed by 2 boosts with 100 mcg TV1gp140ΔV2 protein in MF59 adjuvant at 30 and 45 weeks. Groups 2 (n = 3) and 3 (n =4) did not receive envelope DNA primes but instead received 3 DNA immunizations with 3.5 mg of pCMV*gag* plus 4.2 mg of pCMV*pol* or 5.0 mg of pCMV*gagpol*, respectively; these groups also received 2 IM immunizations with 100 mcg TV1gp140ΔV2 protein in MF59 adjuvant at 30 and 45 weeks.

Figure 4-15 A indicates that priming with TV1gp140ΔV2 envelope-encoding DNA vaccines (group 1) elicited TV1-specific envelope-binding antibodies that were boosted to higher titer after TV1gp140ΔV2 protein immunizations. The potency of nAb responses against SF162 and TV1 were also examined following DNA immunizations (week 28) and after both protein immunizations (week 47) (Figure 4-15 B and C). At the end of the vaccination regimen, animals that had been primed with the DNA encoding the envelope gene showed higher nAb titers against both the homologous TV1 strain and the

neutralization-sensitive SF162 isolate than animals that had been primed with the *gag*-C-*pol* DNA (P=0.0253, t test). This highlights the effectiveness of the TV1gp140ΔV2 protein as a boost in animals that have been primed.

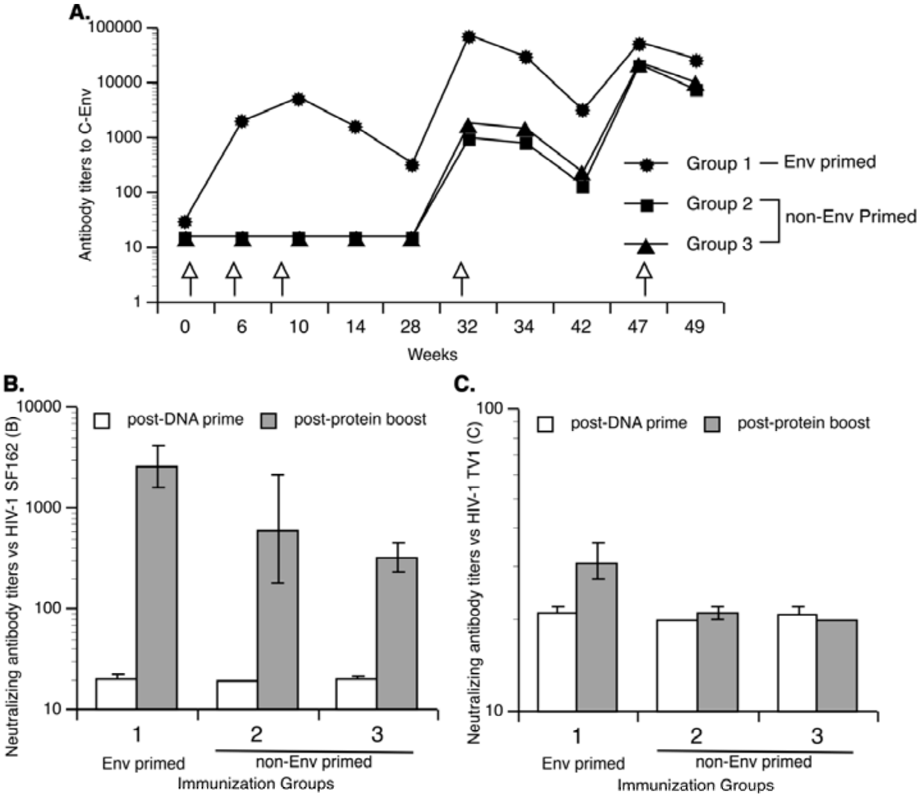

Figure 4-15 Humoral immune responses in rhesus macaques with subtype C immunogens: (A) Oligomeric env-binding Ab titers (geometric mean) during the course of immunization. ELISA was performed using TV1gp140ΔV2. (B) nAb titers against subtype B SF162 and (C) subtype C TV1. Titers are the reciprocal of the highest serum dilution at which RLU were reduced 50% compared to virus control wells. Two timepoints were used to evaluate neutralization titers: 2 weeks before the first protein immunization (post-DNA) and 2 weeks after the second protein immunization (post-protein).

4.10 Clinical studies

4.10.1 Clinical Data on SAAVI DNA-C2 and SAAVI MVA-C

HVTN 073/SAAVI 102 is a phase 1 randomized, placebo-controlled, double-blinded trial conducted in South Africa and the US to evaluate the safety and immunogenicity of SAAVI DNA-C2 followed by boosting with SAAVI MVA-C, in 48 HIV-uninfected adults, 18 to 45 years of age.

Participants were randomly assigned to receive either the SAAVI prime-boost preventive vaccine regimen (n=40) or 0.9% saline placebo (n=8). Vaccination with the SAAVI DNA-C2 vaccine 4 mg (or placebo) occurred at Months 0, 1, and 2; boost vaccinations with the SAAVI MVA-C vaccine 1.45 x 10^9 pfu (or placebo) occurred at Months 4 and 5.

Following vaccination, participants were assessed for local reactogenicity (pain, tenderness, induration, erythema) and systemic reactogenicity (fever, chills, malaise/fatigue, myalgia, arthralgia, headache, nausea, vomiting). In addition, adverse events (AEs) were reported.

The study began enrollment in the US on January 29, 2009 and in South Africa on July 2, 2009. The study completed enrollment of 48 participants as of October 1, 2009. All protocol injections were completed as of February 26, 2010. Study participants have received protocol injections at the following timepoints: month 0 (n = 48), month 1 (n = 46), month 2 (n = 45), month4 (n = 46), and month 5 (n = 45).

An interim review of safety data in May 2010 indicated that for local reactogenicity, only 1 participant reported severe pain at the injection site, which resolved. All other local reactogenicity symptoms were none, or mild or moderate in severity, and resolved. In terms of systemic reactogenicity, there were 2 reports of severe malaise/fatigue, 1 report of severe myalgia, and 1 report of severe headache following vaccination, all of which resolved. All other systemic reactogenicity symptoms were none, or mild or moderate in severity, and resolved.

In regard to AEs, upper respiratory infections were most commonly reported (n=20), followed by lymphadenopathy (n=7). Additional events reported 4 or more times were: decreased hemoglobin (n=6), neutropenia (n=5), AST elevation (n=5), proteinuria (n=4), hypertension (n=6), elevated blood pressure (n=4), headache (n=6, 1 was severe), and abnormal loss of weight (n=6). There were 3 reports each for gastroenteritis (1 was severe), back pain (1 severe), arthralgia, influenza-like symptoms, and oral herpes. Other mild or moderate AEs occurred in only 1 or 2 participants.

One serious adverse event (SAE) of hospitalization for schizophrenia was reported for a participant with a 10-year history of schizophrenia that the participant did not disclose during screening and enrollment. The site investigator assessed the schizophrenia as probably not related to vaccine. Two additional AEs of hospitalization were reported on an expedited basis: 1 stabbing in the back and 1 right forearm injury; both were assessed by the local investigator as not related to the vaccination.

In regard to early product discontinuation, 1 participant withdrew consent for vaccinations and 1 participant was hospitalized for schizophrenia (described above) after each receiving 1 vaccination. Vaccinations were also discontinued in 1 participant who reported tongue swelling (not observed) after the 4th vaccination. All participants remained on study follow-up.

In regard to missed vaccinations, 1 participant did not receive vaccination #3 only (received vaccinations #1, 2, 4, and 5).

Administration of the SAAVI DNA-C2 and SAAVI-MVA injections has concluded. The immunogenicity endpoints in HVTN 073 / SAAVI 102 include assays for humoral and cellular responses on all participants at the primary immunogenicity timepoints, which are the 2-week visits after the scheduled fourth and fifth vaccination visits. Preliminary T cell immunogenicity data are available as of April 15, 2010, and a summary of responses after the fifth (final) injection is provided in Table 4-11. Data is available for 37 of 48 participants (31 vaccinees, 6 controls). Results are shown for vaccine recipients only; samples from 6 placebo recipients tested did not show responses to the HIV antigens.

CD4+ or CD8+ responses for any protein were 24/31(77%), with CD4+ responses predominant which is consistent with the pre-clinical non-human primate peripheral PBMC responses.

Table 4-11 Summary of preliminary ICS data for a subset of participants after final vaccination (HVTN 073 / SAAVI 102)

T cell Subset	**Response to Any Antigen**	**Response by Protein**			
		**Env**	**Gag**	**Nef**	**Pol**
Any	24/31 (77%)	21/31 (68%)	7/31 (23%)	0/31 (0%)	9/31 (29%)
CD4+	21/31 (68%)	20/31 (65%)	5/31 (16%)	0/31 (0%)	2/31 (7%)
CD8+	12/31 (39%)	3/31 (10%)	2/31 (7%)	0/31 (0%)	9/31 (29%)

The magnitude of the responses is noted in Figure 4-16.

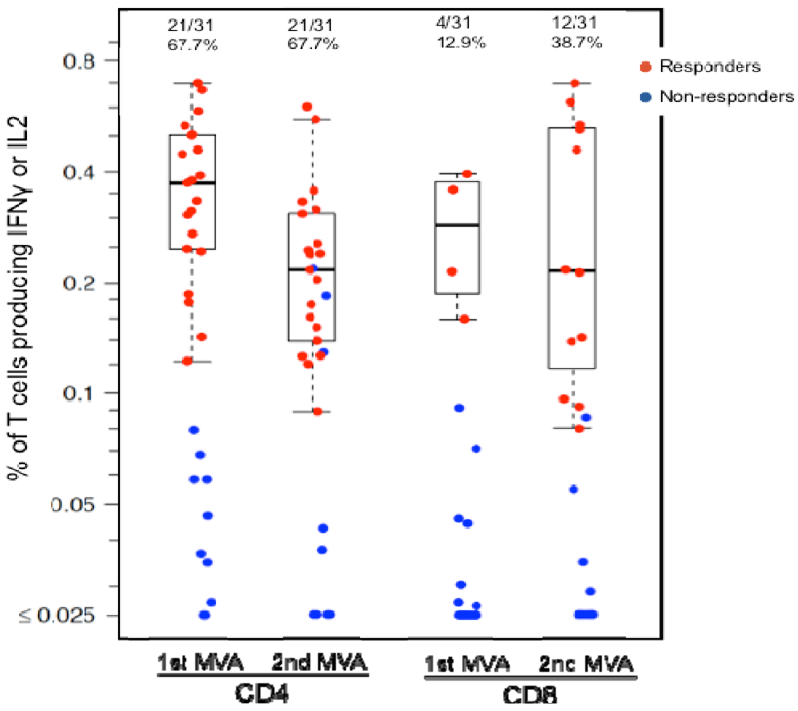

Figure 4-16 Magnitude of the maximum CD4+ and CD8+responses to any potential T-cell epitope (PTE) pools. Positives are shown in red.

4.10.2 Clinical studies of TV1gp140ΔV2

Although the oligomeric HIV subtype C ΔV2 gp140 (TV1gp140ΔV2)/MF59 vaccine has not been administered to humans, other closely related recombinant gp120 subunit vaccine formulations and Novartis' oligomeric HIV subtype B gp140/MF59 have been tested in numerous clinical trials. Overall, these studies have shown that HIV Env, whether monomeric (HIV gp120) or oligomeric (HIV o-gp140), are both safe and immunogenic. Recombinant monomeric vaccine candidates studied include Novartis' early gp120 based candidates from subtypes B and E, and VaxGen's HIV gp120 vaccine candidates from subtypes B and E AIDSVAX [36]. These have been tested in numerous phase 1 trials in uninfected and infected volunteers, including children, and have also been evaluated in phase 2 trials in HIV-1 seronegative volunteers who are at high risk for acquisition of HIV infection. Over 1200 subjects (adults and children) have participated in the evaluation of the Novartis HIV SF2 gp120/MF59 vaccine and the Novartis HIV CM235 Thai E gp120/MF59 vaccine. No unusual or serious vaccine-associated AEs were reported during the conduct of these clinical trials. In general, these vaccines were well tolerated. The only exceptions were transient pain and tenderness at the injection site, which were reported by most subjects. Additional details can be found in the Novartis subtype C gp140 IB.

VaxGen's HIV gp120-based vaccines AIDSVAX B and E have been the most widely tested HIV vaccine candidates in human trials [15,37]. After testing these vaccines for safety and immunogenicity in phase 1 and 2 trials, the bivalent vaccines AIDSVAX B/B and AIDSVAX B/E were evaluated in 2 phase 3 trials as adjuvanted protein vaccines in Alum, and then more recently, AIDSVAX B/E was tested as a booster in the first

example of a partially effective phase 3 vaccine trial just completed in Thailand [15]. The initial multicentre phase 3 trial of AIDSVAX B/B was conducted mainly in Canada and the US and was complete at the end of 2002. The second phase 3 trial was conducted in Thailand with the AIDSVAX B/E vaccine. The study volunteers for the AIDSVAX B/B trial included 5108 men who have sex with men and 309 at-risk women, all of whom were meant to be HIV negative when they joined the trial. At the end of the trial, VaxGen announced that AIDSVAX B/B did not prove effective in the trials conducted in North America and Europe. The study did not show a statistically significant reduction of HIV infection within the study population as a whole, which was the primary endpoint of the trial. That trial was followed by a phase 3 trial in Thailand, in which AIDSVAX B/E was evaluated in 2546 injection drug users. Despite the successful completion of this efficacy trial, the vaccine did not prevent HIV-1 infection or delay HIV-1 disease progression [38]. The most recent demonstration of the safety, immunogenicity, and potential efficacy of these gp120 vaccine candidates was reported following disclosure of the results of the heterologous prime-boost phase 3 trial in Thailand wherein more than 6000 individuals received 1-2 doses of the AIDSVAX B/E gp120 proteins as booster immunizations.

Furthermore, 2 phase 1 clinical trials have been conducted with Novartis' subtype B o-gp140 ΔV2 Env antigen. A phase 1, open label, randomized trial was conducted in Great Britain on healthy adults to evaluate the safety (primary endpoint) and immunogenicity (secondary endpoint) of 3 intranasal (IN) immunizations with HIV-1 subtype B SF162 o-gp140ΔV2 Env protein in association with the LTK63 mucosal adjuvant followed by IM boosting with HIV-1 o-gp140ΔV2 Env protein in Novartis MF59 adjuvant (MUVAPRED C56P1). The dose of LTK63 was 30 mcg and the Sub B gp140 dose was 100 mcg. The study was conducted in healthy subjects aged 18-45. For ease of study administration, the groups were enrolled sequentially.

A total of 30 subjects were enrolled with 10 subjects randomized to each of the 3 groups. The protocol was amended to halt further IN administration of LTK63 following a report of an AE (i.e., facial nerve paralysis) with a possible association with the LTK63 adjuvant in another study that did not involve subtype B gp140 [39]. When this event occurred, all 10 subjects in group 2 (LTK63+gp140 IN) and 5 of the subjects in group 1 (LTK63 alone) had received all 3 IN administrations. As a result, 5 subjects in the LTK63 alone group received only one dose of LTK63. There was one withdrawal from the study due to an AE in the LTK63 alone group as described below.

There were two SAEs in this study. One subject was reported to have Bell's Palsy (left side unilateral facial nerve palsy), considered possibly related to LTK63 44 days following the first administration of LTK63. This event of Bell's palsy was reported after the event had been reported in the other study and after IN dosing with LTK63 was suspended in this study. This subject never received any dose of subtype B gp140 [39]. Another subject in the LTK63 + gp140 group had a liver laceration due to a fall from a bicycle considered not related to the study vaccine. This subject completed all vaccinations. The results of this study indicated that the HIV-1 o-gp140ΔV2 Env protein in Novartis MF59 vaccine candidate is safe and immunogenic. Additional details can be found in the IB for o-gp140ΔV2.

The phase 1 HVTN 049 trial, named "A phase 1 Clinical Trial to Evaluate the Safety and Immunogenicity of a Clade B gag DNA/PLG and env DNA/PLG Microparticles Vaccine and a Clade B Recombinant, Oligomeric gp140/MF59 Adjuvant Vaccine in Healthy, HIV-1 Uninfected Adult Participants" was conducted in the USA, within the NIH-

sponsored HIV Vaccine Trials Network, to test the safety (primary endpoint) and immunogenicity (secondary endpoint) of a subtype B gag and env DNA/PLG microparticles priming followed by a boosting with a subtype B recombinant o-gp140ΔV2 Env protein in MF59 adjuvant. Additionally, the safety and immunogenicity of the subtype B recombinant o-gp140ΔV2Env/MF59 vaccine alone was evaluated. That phase 1 trial indicated that the regimen is generally safe and immunogenic.

Although, HIV-1 subtype C recombinant oligomeric V2 deleted gp140 (TV1gp140ΔV2) has not been administered to humans, GMP preparation of this material is highly comparable to the subtype B SF162 o-gp140ΔV2 GMP material. Prior to GMP manufacturing of both subtype B and subtype C Env antigens, a significant amount of process development was performed including development of the cell culture process, purification, analytical and formulation development.

4.10.3 Clinical studies of MF59

While MF59 in combination with TV1gp140ΔV2 has not been administered to humans, other subunit antigens (e.g., hepatitis C virus [HCV] E2, HIV gp120, hepatitis B surface antigen [HBsAg], cytomegalovirus [CMV] gB, and herpes simplex virus type 2 [HSV-2] gD and gB) have been combined with MF59 adjuvant and administered to over 20,000 subjects to date with an excellent safety and tolerability profile [40-46]. Additional information on MF59 can be found in the MF59 Investigator Brochure.

4.10.4 Clinical studies of the proposed product combination

The SAAVI DNA-C2 and SAAVI MVA-C products have been evaluated in combination in the preclinical and ongoing clinical trials described above. However, the Novartis TV1gp140ΔV2 has not been evaluated in clinical trials, to date. Furthermore, the Novartis product has never been evaluated in combination with the SAAVI products. However, pox-vectored and protein combinations have been evaluated in several trials including some AIDS Vaccine Evaluation Group (AVEG) trials in the 1990s [31,36,47] and, most recently, in RV144.

4.10.5 Evidence that pre-immunity to the poxvirus vectors can blunt immune responses

Poxvirus immunity is long-lived [33] and therefore pre-immunity to MVA could prevent the use of recombinant MVA as a second generation vaccine or as a vaccine against other infectious diseases [48,49]. There is some evidence that pre-immunity to the poxvirus vectors results in lower immune responses. For example, an attenuated vaccinia virus (VV) vector expressing Japanese encephalitis virus (JEV) proteins, NYVAC-JEV vaccine, induced nAb responses only in vaccinia-nonimmune recipients while vaccinia-immune volunteers failed to develop protective JEV antibodies. Another study with an HIV vaccine with 3 DNA vaccine primes and an MVA boost demonstrated that while pre-immunity to VV did not abolish the immune response the magnitude of response was lower than when there was no pre-immunity [50]. Therefore, there is a risk of blunted response in participants that have immunity to VV; and for the purpose of this trial people with a history of vaccinia vaccination are excluded (see Section 7.2).

4.11 Potential risks of study products and administration

Table 4-12 Summary of potential risks of study products and administration

Common	• Mild to moderate injection site pain, tenderness, erythema, or swelling/induration/edema<br>• Malaise/fatigue, myalgia, or headache in the first few days following injection<br>• A vaccine-induced positive HIV Ab test result
Less common	• Severe injection site pain or tenderness<br>• Fever, chills, flu-like syndrome, arthralgia, rash, nausea, or dizziness in the first few days following injection<br>• Vasovagal reaction/lightheadedness/dizziness related to the injection procedure<br>• Transient changes in clinical laboratory values<br>• Injection site hematoma, bruising/ecchymosis, laceration, other transient lesions, or bleeding related to the injection procedure
Uncommon or rare	• Severe localized injection site reaction, such as sterile abscess or secondary bacterial infection<br>• Allergic reaction, including rash, urticaria, angioedema, bronchospasm, or anaphylaxis
Unknown frequency or theoretical risks	• Muscle damage at the injection site<br>• Myo/pericarditis (MVA)<br>• Autoimmune disease or cancer<br>• Effects on a participant's response to an approved HIV vaccine administered in the future<br>• Effects on susceptibility to HIV, if the participant is exposed to HIV<br>• Effects on the course of HIV infection/disease, if the participant is infected with HIV<br>• Effects on the fetus and on pregnancy

5 Objectives and endpoints

5.1 Primary objectives and endpoints

Primary objective 1:

- To evaluate the safety and tolerability of IM administration of SAAVI DNA-C2, SAAVI MVA-C and Novartis subtype C gp140/MF59 vaccines, in HIV-uninfected healthy vaccinia-naïve adult participants in South Africa.

Primary endpoint 1:

- Safety data, which will include signs and symptoms of local and systemic reactogenicity, laboratory measures of safety, and AEs and SAEs.

Primary objective 2:

- To characterize and rank the four vaccine regimens and to select the best performing vaccine regimen based on HIV-specific nAb responses following vaccination with Novartis subtype C gp140/MF59 vaccine, as a concurrent or sequential boost to SAAVI MVA-C prime; SAAVI MVA-C boost to SAAVI DNA-C2 prime; and SAAVI MVA-C with concurrent Novartis subtype C gp140/MF59 boost to SAAVI DNA-C2 prime in HIV-uninfected healthy vaccinia-naïve adult participants in South Africa.

Primary endpoint 2:

- nAb titers to tier 1 and tier 2 virus isolates as assessed by magnitude-breadth curves. Please see Sections 6 and 10 for more detail.

5.2 Secondary objectives and endpoints

Secondary objective 1:

- To evaluate CD4+ T-cell responses to SAAVI MVA-C as a prime with sequential and concurrent Novartis subtype C gp140/MF59 vaccine boost, with and without SAAVI DNA-C2 prime, and T-cell responses to SAAVI DNA-C2 prime with SAAVI MVA-C boost in HIV-uninfected healthy vaccinia-naïve adult participants in South Africa.

Secondary endpoints 1:

- Response rate of CD4+ T-cell responses detected by HIV-1 specific ICS.
- Magnitude of $CD4^+$ T-cell responses detected by HIV-1 specific ICS.

Secondary objective 2:

- To evaluate HIV-specific binding Ab responses to the vaccine regimens (SAAVI MVA-C priming with sequential or concurrent Novartis subtype C gp140/MF59 vaccine boost; SAAVI DNA-C2 priming with SAAVI MVA-C boosting, with or without Novartis subtype C gp140/MF59 vaccine) in HIV-uninfected healthy vaccinia-naïve adult participants in South Africa.

Secondary endpoints 2:

- Total HIV-1 Env-specific IgG-binding antibodies as determined by HIV-1 multiplex Ab assay.
- ConS gp140, Du151 Env, and/or TV1gp140 specific IgG subclass (IgG1- IgG4) and IgA characterization as determined by HIV-1 multiplex Ab assay.

5.3 Exploratory objectives

Exploratory objective 1:

- To evaluate HIV-specific B-cell/Ab immune responses to the vaccine regimens (SAAVI MVA-C priming with sequential or concurrent Novartis subtype C gp140/MF59 vaccine boost; SAAVI DNA-C2 priming with SAAVI MVA-C boosting, with or without Novartis subtype C gp140/MF59 vaccine) in HIV-uninfected healthy vaccinia-naïve adult participants in South Africa.

Exploratory endpoints 1 (may be conducted):

- Ab dependent cellular cytotoxicity
- B cell phenotyping by flow cytometry
- B cell ELISpot
- Binding Ab epitope mapping by peptide array
- HIV-specific Ab avidity

Exploratory objective 2:

- To evaluate CD8+ T-cell responses to SAAVI MVA-C as a prime with sequential and concurrent Novartis subtype C gp140/MF59 vaccine boost, with and without SAAVI DNA-C2 prime, and T-cell responses to SAAVI DNA-C2 prime with SAAVI MVA-C boost in HIV-uninfected healthy vaccinia-naïve adult participants in South Africa.

Exploratory endpoints 2 (may be conducted):

- Response rate of CD8+ T-cell responses detected by HIV-1 specific ICS.
- Magnitude of CD8+ T-cell responses detected by HIV-1 specific ICS.

Exploratory objective 3:

- To evaluate HIV-specific mucosal IgA and IgG responses elicited by the vaccine regimens (SAAVI MVA-C priming with sequential or concurrent Novartis subtype C gp140/MF59 vaccine boost; SAAVI DNA-C2 priming with SAAVI MVA-C boosting, with or without Novartis subtype C gp140/MF59 vaccine) in HIV-uninfected healthy vaccinia-naïve adult participants in South Africa

Exploratory endpoint 3 (may be conducted):

- HIV-specific IgG and IgA in rectal and cervical secretions as measured by HIV-1 multiplex antibody assay

6 Statistical considerations

6.1 Accrual and sample size calculations

Recruitment will target 184 healthy, HIV-uninfected healthy vaccinia-naïve adult participants in South Africa. Subjects will be randomized to 1 of 4 groups in a 1:1:1:1 allocation, and within each group, will be randomized to vaccine:placebo in a 38:8 allocation.

Since enrollment is concurrent with receiving the first study vaccination, all participants will provide some safety data. Hence, sample size calculations for safety in section 6.1.1 are based on the target sample sizes. It is possible, however, for immunogenicity data to be missing; previous HVTN and AVEG studies suggest 10% is a reasonable estimate for the rate of missing data. For this reason, the sample size calculations in section 6.1.2 account for 10% of enrolled participants having missing data for the primary immunogenicity endpoints.

6.1.1 Sample size calculations for safety

The goal of the safety evaluation for this study is to identify safety concerns associated with product administration. Sample size calculations for safety are expressed in terms of the ability to detect AEs.

The ability of the study to identify AEs can be expressed by the true event rate above which at least 1 event would likely be observed and the true event rate below which no events would likely be observed. Specifically, for each vaccine arm of the study (n = 38), there is a 90% chance of observing at least 1 event if the true rate of such an event is 5.9% or more; and there is a 90% chance of observing no events if the true rate is 2.7% or less.

Probabilities of observing 0, 1 or more, and 2 or more events among arms of size 38 and 152 are presented in Table 6-1 for a range of possible true event rates. These calculations provide a more complete picture of the sensitivity of this study design to identify potential safety problems with the vaccine. In previous AVEG HIV vaccine trials, 3.5% of control participants experienced a SAE; in HVTN vaccine trials (as of January 2006) about 1% of control participants experienced such an event.

Table 6-1 Probability of observing 0 events, 1 or more events, and 2 or more events, among arms of size 38 and 152, for different true event rates

True event rate (%)	Pr(0/38)	Pr(1+/38)	Pr(2+/38)	Pr(0/152)	Pr(1+/152)	Pr(2+/152)
1	0.683	0.314	0.055	0.217	0.783	0.450
3.5	0.258	0.742	0.386	0.004	0.996	0.971
5	0.142	0.858	0.573	0.000	1.000	0.996
10	0.018	0.982	0.905	0.000	1.000	1.000
20	0.000	1.000	0.998	0.000	1.000	1.000
30	0.000	1.000	1.000	0.000	1.000	1.000
40	0.000	1.000	1.000	0.000	1.000	1.000

An alternative way of describing the statistical properties of the study design is in terms of the 95% confidence interval (CI) for the true rate based on the observed data. Table 6-2 shows the exact 2-sided 95% CIs for the probability of an event based on a particular observed rate. If none of the 152 participants receiving a vaccine regimen experience a safety event, the 95% exact 2-sided upper confidence bound for the true rate of such events in the total vaccinated population is 0.024. Restricted to any of the individual vaccine arms (n = 38), the exact 2-sided upper confidence bound for this rate is 0.092.

Table 6-2 Exact 2-sided 95% CIs based on observing a particular rate of safety endpoints for arms of size 38 and 152

Observed event rate	CI
0/38	(0.000, 0.092)
1/38	(0.001, 0.138)
2/38	(0.006, 0.177)
0/152	(0.000, 0.024)
1/152	(0.000, 0.036)
2/152	(0.002, 0.047)

6.1.2 Sample size calculations for immunogenicity

The sample size calculations are based on the area under the magnitude-breadth curve (AUC-MB) endpoint, calculated for both the 3 tier 1 isolates and for the 12 tier 2 isolates (see section 10.4.1). Sample size calculations are performed for the primary analysis described in 6.4.4, which entails 3 analyses. The first determines which of the 4 vaccine arms have higher mean AUC-MBs than the pooled placebo group according to Dunnett's procedure; the arms that do are said to pass the "tier 1 screen." For those vaccine arms that pass the tier 1 screen, the second analysis determines which of the 4 vaccine arms have higher mean AUC-MBs than the pooled placebo group according to Dunnett's procedure; the arms that do are said to pass the "tier 2 screen." The third analysis selects the best vaccine arm among those that pass the tier 2 screen, defined as the regimen with the highest average AUC-MB.

6.1.2.1 Power for Detecting Vaccine Arms Superior to Placebo

Based on Table 1 of [51] (entry beta=0.90, alpha=0.05, k=4, nu=infinity and using n=34 to account for 10% missing data), there is at least 90% probability that all of the vaccine groups with mean AUC-MB at least 1 standard deviation (sd) larger than that of the pooled placebo group will be detected with Dunnett's procedure, with at most 5% chance of any false positive results. This result applies for the tier 1 screen and for the tier 2 screen if all 4 vaccine regimens pass the tier 1 screen. This result suggests that the trial is very well-powered to identify the set of vaccine arms that have superior tier 1 neutralization magnitude and breadth than the pooled placebo group.

To interpret how much power is available for the tier 2 screen, we consider neutralization data from 90 recipients of the AIDSVAX bivalent subtype B vaccine in the North America/Netherlands VaxGen efficacy trial (Gilbert et al., unpublished data). For these 90 vaccine recipients, the sample average of the AUC-MB to a panel of 12 tier 2 subtype B isolates was 1.04 and the sample sd was 0.204. In addition, based on the 30 placebo recipients evaluated for neutralization responses to the tier 2 isolate panel, the sample average of the AUC-MB was 0.827 and the sample sd was 0.143. The average difference of 0.213 is about the same size as the sd for the vaccine group, suggesting that this type of difference would be just-detectable with 90% power in this trial with 34 subjects per group. Therefore, the trial is powered to identify the set of vaccine arms with superior tier 2 neutralization than placebo for effect sizes equal to (or greater than that) observed in the VaxGen trial.

6.1.2.2 Probability of Selecting the Best Vaccine Regimen

Probabilities of correct selection are estimated assuming that all 4 vaccine regimens pass the tier 2 screen. AUC-MBs were simulated for the 4 vaccine arms as follows. First, one vaccine arm was simulated using a normal distribution with mean mu1 = 1.04 and sd 0.204, matching the results observed in the VaxGen trial. The other vaccine arms were simulated using the same sd and different mean values mu2, mu3, and mu4. To interpret the results, note that a difference of mu2 – mu1 = $\log_{10}(X)$ means that the typical average titer to an isolate in the panel is X-fold higher for vaccine arm 2 than for vaccine arm 1.

For several scenarios of means, Table 6-3 shows the probability of correctly selecting the vaccine arm with the highest mean AUC-MB for nAb, and the probability of correctly selecting a vaccine group that has mean within $\log_{10}(1.2)$ (typical 1.2-fold difference in IC50 to an isolate) of the truly best vaccine arm. To interpret the table, note that a mean increase from 1.04 to 1.34 represents a 2-fold increase in typical neutralization titer. Similarly, increases from 1.04 to 1.08; 1.04 to 1.19; and 1.04 to 1.27 represent 1.1-fold, 1.4-fold, and 1.7-fold increases (ie, 10%, 40%, and 70% increases).

Table 6-3 Probability of correctly selecting the vaccine arm with the best/highest neutralization AUC-MB

Mean AUC-MB Group 1 (mu1)	Mean AUC-MB Group 2 (mu2)	Mean AUC-MB Group 3 (mu3)	Mean AUC-MB Group 4 (mu 4)	Probability Selecting the Best Vaccine	Probability Selecting the Best Vaccine within log10(1.2)
1.04	1.04	1.04	1.34	1.0	1.0
1.04	1.04	1.04	1.27	1.0	1.0
1.04	1.04	1.04	1.19	0.99	1.0
1.04	1.04	1.04	1.08	0.61	1.0
1.04	1.04	1.33	1.34	0.59	1.0
1.04	1.04	1.26	1.27	0.62	1.0
1.04	1.04	1.06	1.08	0.54	1.0
1.04	1.04	1.27	1.34	0.92	1.0
1.04	1.04	1.19	1.34	1.0	1.0
1.04	1.04	1.19	1.27	0.95	0.95
1.04	1.04	1.08	1.27	1.0	1.0
1.04	1.04	1.33	1.34	0.42	1.0
1.04	1.26	1.26	1.27	0.44	1.0
1.04	1.17	1.17	1.19	0.47	1.0
1.04	1.06	1.06	1.08	0.47	1.0
1.04	1.27	1.27	1.34	0.87	1.0
1.04	1.19	1.19	1.34	1.0	1.0
1.04	1.19	1.19	1.27	0.92	0.92
1.04	1.08	1.08	1.27	1.0	1.0
1.04	1.19	1.27	1.34	0.92	1.0
1.04	1.08	1.19	1.34	1.0	1.0
1.04	1.08	1.19	1.27	0.95	0.95
1.04	1.04	1.08	1.19	0.98	0.98

The calculations show that for all scenarios studied, there is at least 90% power to select a vaccine arm that produces typical titers to isolates within 1.2-fold of the truly best regimen (last column). Therefore the study is unlikely to advance a vaccine regimen for further testing that is substantially less potent in neutralization activity than the truly best regimen.

6.1.2.3 Comparison of Immune Response Rates among Vaccine Arms

Secondary and exploratory endpoints include the rate of CD4+ T-cell responses and the rate of other endpoints. The precision with which the true response rate can be estimated from the observed data depends on the true underlying response rate and the sample size. Exact 2-sided 95% CIs for the response rate based on observing a particular rate of responses in a vaccine arm is shown in Table 6-4. The n = 34 assumes a 10% loss of data.

Table 6-4 Exact 2-sided 95% CIs for the true response rate based on observing a particular rate of responses in the vaccinees (n = 34)

No. of responses	Observed response rate (%)	CI (%)
17	50	(32.4,67.6)
20	58.8	(40.7, 75.4)
24	70.6	(52.5, 84.9)
27	79.4	(62.1, 91.3)
31	91.2	(76.3, 98.1)

Table 6-5 shows power for a comparison of immunogenicity response rates between vaccine arms of size n = 34, allowing for 10% missing data. These calculations use an exact 2-sided test with a Type I error rate of 0.05/6 = 0.0083.

Table 6-5 Power for comparison of response rates between 2 vaccine arms (n1 = 34, n2 = 34)

True response rate vaccine arm 1 (%)	Minimum true response rate in Arm 2 in order to detect a difference (%)	
	80% power	90% power
40	82.3	86.2
50	89.7	92.8
60	95.5	97.5
70	99.5	NA
80	NA	NA

6.2 Randomization

The randomization sequence will be obtained by computer-generated random numbers and provided to each HVTN CRS through the SDMC's Web-based randomization system. The randomization will be done in blocks to ensure balance across arms. At each institution, the pharmacist with primary responsibility for dispensing study products is charged with maintaining security of the treatment assignments.

The randomization for this trial will be stratified by site.

6.3 Blinding

Participants and site staff (except for site pharmacists) will be blinded to treatment arm assignment (vaccine or placebo) and will be blinded to assignment to group 1 or 2, as well as to assignment to group 3 or 4. Site staff are unblinded to assignment to the combined groups 1 and 2 versus to the combined groups 3 and 4. The laboratory staff is completely blinded to all groups and to the treatment arm assignments. Therefore, comparisons of immunogenicity endpoints between arms are statistically valid with very low risk for bias due to unblinding. Study product assignments are accessible to those HVTN CRS pharmacists, DAIDS protocol pharmacists and contract monitors, and SDMC staff who are required to know this information in order to ensure proper trial conduct. Any discussion of study product assignment between pharmacy staff and any other HVTN CRS staff is prohibited. The HVTN SMB members are also unblinded to treatment assignment in order to conduct review of trial safety.

When a participant leaves the trial prior to study completion, the participant will be told he or she must wait until all the participants are unblinded to learn his or her treatment assignment.

Emergency unblinding decisions will be made by the site investigator. If time permits, the HVTN PSRT should be consulted before emergency unblinding occurs.

6.4 Statistical analysis

All data from enrolled participants will be analyzed according to the initial randomization assignment regardless of how many vaccinations they received. Since enrollment is concurrent with receiving the first vaccination, all participants will have received at least 1 vaccination and therefore will provide some safety data. The analysis is a modified intent-to-treat analysis in that individuals who are randomized but not enrolled do not contribute data and hence are excluded. Because of blinding and the brief length of time between randomization and enrollment—typically no more than 4 working days—very few such individuals are expected.

Analyses for primary endpoints will be performed using SAS. All other descriptive and inferential statistical analyses will be performed using SAS, StatXact, S-Plus, and/or R statistical software.

6.4.1 Analysis variables

The analysis variables consist of baseline participant characteristics, safety, and immunogenicity for primary- and secondary-objective analyses.

6.4.2 Baseline comparability

Randomized groups will be compared for baseline participant characteristics using descriptive statistics.

6.4.3 Safety analysis

Reactogenicity

The number and percentage of participants experiencing each type of reactogenicity sign or symptom will be tabulated by severity. For a given sign or symptom, each participant's reactogenicity will be counted once under the maximum severity for all injection visits.

AEs

AEs will be tabulated using MedDRA preferred terms. The number and percentage of participants experiencing each specific AE will be tabulated by severity and by relationship to treatment. Relationship to treatment is coded as either related or not related. For the calculations in the severity tables, if a participant has multiple specific AEs, then only the maximum severity AE will be included. For the calculations in the relationship to treatment tables, if a participant has multiple specific AEs with some not related to treatment and some related to treatment, then only the specific AEs related to treatment will be included. If a participant has multiple specific AEs related to treatment, then only the one of greatest severity will be included.

A listing of SAEs will provide details of the events including severity, relationship to study product (related or not related), time between onset and last vaccination, number of vaccinations received, and a summary of the event.

Local laboratory values

Boxplots of local laboratory values will be generated for baseline values and for values measured during the course of the study. Each boxplot will show the first quartile, the median, and the third quartile. Outliers (values outside the boxplot) will also be plotted. If appropriate, horizontal lines representing boundaries for abnormal values will be plotted.

6.4.4 Immunogenicity analysis

The analysis of the immunogenicity endpoints that evaluate a single timepoint will be based on data from the primary immunogenicity timepoint defined in section 10.3.

6.4.4.1 General Approach

For the statistical analysis of immunogenicity endpoints, data from enrolled participants will be used according to the initial randomization assignment regardless of how many injections they received. Assay results that are unreliable, from specimens collected outside of the visit window, or from HIV-infected participants postinfection are excluded. Since the exact date of HIV infection is unknown, any assay data from blood draws 4 weeks prior to an infected participant's last seronegative sample and thereafter may be excluded. If an HIV-infected participant does not have a seronegative sample postenrollment, then all data from that participant may be excluded from the analysis. Additional analyses may be performed for participants who received all scheduled injections per protocol. For this trial, this issue is especially relevant for comparing group 2 versus groups 1, 3 and 4.

For secondary and exploratory endpoints that are qualitative (ie, positive or negative) analyses will be performed by tabulating the frequency of positive responses for each assay by arm at each timepoint that an assessment is performed. For vaccine arms, crude and net response rates will be presented with their corresponding exact 95% CI estimates, as well as the Fisher's exact test p-value comparing vaccine to pooled placebo arms. For the pooled placebo arms, crude response rates and exact 95% CI estimates will be presented.

To compare the response rates among the 4 vaccine arms, first an overall test for any difference in crude response rate among any of the 4 vaccine arms will be conducted, using Fisher's exact test. If this test is significant at the 2-sided 0.05 level, then the Agresti-Coull method [52] will be used to construct 2-sided (1-0.05/6)×100% CIs about the differences in response rates for each of the 6 pair-wise comparisons of vaccine arms. Significance of the differences will be based on whether the CI excludes zero. If assays are run from samples taken at multiple timepoints, the probability of observing at least 1 positive response by a given timepoint and the probability of observing more than 1 positive response by a given timepoint will be estimated, with corresponding CIs, for each vaccine arm using maximum likelihood-based methods [53].

For continuous assay variables, the difference between vaccine arms at a specific timepoint will be tested with a nonparametric Wilcoxon rank sum test if the data are not

normally distributed and with a 2-sample t-test if the data appear to be normally distributed. An appropriate data transformation (eg, $\log_{10}$ transformation) may be applied to better satisfy assumptions of symmetry and homoscedasticity (constant variance). Similar to the comparison of response rates between vaccine arms, first an overall test will be done to evaluate any differences among the 4 vaccine arms, using the Kruskal-Wallis rank test or an F-test (depending on the normality assumption). Secondly, if the overall test is significant at the 2-sided 0.05 level, then individual tests comparing the 6 pairs of vaccine arms will be done. If rank-based tests are used then the tests will be inverted to construct Hodges-Lehmann point-estimates and 2-sided $(1\text{-}0.05/6) \times 100\%$ CIs about the differences in location centers of the 6 pair-wise comparisons of vaccine arms, and if actual-value tests are used then Fisher's least significant differences procedure will be used to construct simultaneous 95% CIs about the 6 pairs of mean differences.

More sophisticated analyses of continuous assay variables employing repeated measures methodology (for example, repeated measures analysis of variance [ANOVA] or generalized estimating equations) may be utilized to incorporate immune responses over several timepoints. However, inference from such analyses would be limited by the small sample size of this study. All statistical tests will be 2-sided and will be considered statistically significant if $p \leq 0.05/K$, where K is the number of pair-wise vaccine arms compared head-to-head. Graphical representations of the longitudinal immune responses will also be given.

Some immunologic assays have underlying continuous or count-type readout that is often dichotomized into responder/non-responder categories. For these assays, graphical and tabular summaries of the underlying distributions will be made. If arm comparisons in these underlying distributions reveal that differences are best summarized as a shift in the location of the distribution, then results will be presented in the form of arm means (or medians) with associated CIs and statistical tests for differences between arms as described above. If arm comparisons in these underlying distributions reveal that differences are best summarized by a mixture model (ie, responder and non-responder subgroups are clearly identifiable), then results will be presented in the form of response rates with associated CIs and statistical tests as described above. In addition, Lachenbruch's test statistic [54] will be used for evaluating the composite null hypothesis of equal response rates in the 2 arms and equal response distributions among responders in the 2 such arms. This test statistic equals the square of a binomial Z-statistic for comparing the response rates plus the square of a Wilcoxon statistic for comparing the response distributions in the subgroup of responders. This test evaluates any differences in the response distributions between the 2 arms. A permutation procedure is used to obtain a 2-sided p-value.

6.4.4.2 Primary analyses of neutralization magnitude-breadth curves

Tier 1 Screen of Vaccine Regimens versus Placebo

The area-under-the-magnitude-breadth curve (AUC-MB) to the tier 1 panel of 3 isolates will be computed for each participant with evaluable neutralization data, as described in [55]. Dunnett's procedure will be applied with 2-sided alpha = 0.05 to determine which of the 4 vaccine groups have a significantly higher mean AUC-MB than that of the pooled placebo groups, as described in [51] (see their formula (1.1)). This procedure will be applied to construct 95% CIs about the 4 differences in mean AUC-MB for each

vaccine regimen versus the pooled placebo groups (vaccine – placebo), which simultaneously have at least 95% coverage probability. The rule for a vaccine regimen passing the tier 1 screen is that the lower confidence limit about the mean difference is above zero. The vaccine regimens passing the tier 1 screen will be advanced to tier 2 evaluation, and regimens failing the tier 1 screen are not planned to undergo evaluation for neutralization of the tier 2 isolates.

Tier 2 Screen of Vaccine Regimens versus Placebo

For the set of vaccine regimens passing the tier 1 screen, the same Dunnett's procedure as described above, using the AUC-MB endpoint for the tier 2 isolates, will be used to determine the set of vaccine regimens that pass the tier 2 screen.

Select the Best Vaccine Regimen Among Those Passing the Tier 2 Screen

The vaccine regimens that passed the tier 2 screen will be ranked by the estimated mean of the AUC-MB curves. The vaccine regimen with the highest estimated mean will be selected as the best regimen.

6.4.4.3 Secondary Analyses of Neutralization Magnitude-Breadth Curves

Superiority Comparisons of Vaccine Regimens Passing the Tier 2 Screen.

For the set of vaccine regimens that passed the tier 2 screen, an F-test will be performed for whether any of the mean AUC-MBs differ. If this test is not significant (p-value > 0.05), then the conclusion will be that there are no significant differences in mean AUC-MBs among the advanced vaccine regimens. If the F-test is significant, then simultaneous 95% CIs about the mean-differences in AUC-MBs will be reported. These CIs are computed as the estimated mean-difference plus or minus t_.025 multiplied by the square-root of S2 (1/ni + 1/nj), where t_.025 is the 97.5th percentile of a t-distribution with N – m degrees of freedom, where N is the total number of vaccine recipients evaluated (summing over the advanced vaccine regimens) and m is the number of advanced vaccine regimens. In addition, S2 is an estimate of the common sample variance of the AUC-MB, whereas ni and nj are the sample sizes of evaluable subjects for vaccine regimens i and j being compared. Following Fisher's least significant difference procedure, the pairs of vaccine regimens with this CI excluding zero are deemed to have a significant difference.

Omnibus Comparison of Magnitude-Breadth Distributions

The analyses of magnitude-breadth described above are based on the endpoint area-under-the-curve, which is interpreted as the average $\log_{10}$ IC50 to the set of isolates in the test panel. Use of this endpoint is maximally statistically powerful if 1 vaccine arm has greater magnitude and breadth than the comparator vaccine arm, but may miss an effect wherein 1 vaccine arm has greater magnitude and the comparator vaccine arm has greater breadth. Therefore, a secondary analysis will compare the distribution of magnitude-breadth curves among vaccine arms using the test statistic max|BdG| from Huang, et al [55] (see page 85), which is designed to detect general differences in magnitude-breadth curve distributions.

Selecting the Best Vaccine Regimen Among Those Passing the Tier 2 Screen

For each vaccine regimen that passes the tier 2 screen, the best vaccine regimen will be deemed as that with the greatest value of the max|BdG| test statistic comparing its distribution of magnitude-breadth curves versus the pooled placebo group.

Superiority Comparisons of Vaccine Regimens Passing the Tier 2 Screen

Similarly, the max|BdG| test statistic will be used to compare the distribution of magnitude-breadth curves between each pair of advanced vaccine regimens. The Holm-Bonferroni procedure will be applied to determine the pairs of regimens with significant differences in distribution controlling the family-wise false positive error rate at no more than 0.05.

6.4.4.4 Analysis of CD4+ T-cell responses (secondary endpoints 1)

The CD4+ T-cell response rate will be evaluated and compared among the randomized vaccine arms using the approach for qualitative assay variables described in Section 6.4.4.1 above. The total magnitude of CD4+ T-cell response, as measured by the sum of peptide pool-specific CD4+ T-cell readouts from the ICS assay over all evaluated pools, will be evaluated and compared among the randomized vaccine arms using the approach for continuous assay variables described in Section 6.4.4.1 above.

6.4.4.5 Analysis of HIV-specific binding responses to the vaccine regimens (secondary endpoints 2)

Total HIV-1 Env-specific IgG antibodies will be compared among the randomized vaccine arms using the approach for continuous assay variables described in Section 6.4.4.1 above. The same analysis approach will be used to evaluate and compare the levels of IgG1-, IgG2-, IgG3-, and IgG4-specific gp140 antibodies and the levels of ConS gp140 IgA antibodies, where the CIs will have at least 95% simultaneous coverage. This will require an additional correction for evaluation of the 5 Ab types (for rank-based tests this will entail CIs for vaccine-arm differences in location centers at level $(1 - 0.05/[6\times5])\times100\%$).

6.4.4.6 Missing data

Based upon previous AVEG and HVTN trials, missing 10% of immunogenicity results for a specific assay is common due to study participants terminating from the study early, problems in shipping specimens, or low cell viability of processed PBMCs. To achieve unbiased statistical estimation and inferences with nonparametric tests and generalized linear models fit by generalized estimating equation (GEE) methods, missing data are assumed to be missing completely at random (MCAR). MCAR assumes that the probability of an observation being missing does not depend upon the observed responses or upon any unobserved covariates but may depend upon observed covariates (eg, missing more among whites than nonwhites). When missing data are minimal (specifically if no more than 20% of participants are missing any values), then nonparametric tests and GEE methods will be used, because violations of the MCAR assumption will have little impact on the estimates and hypothesis tests.

If a substantial amount of immunogenicity data are missing (at least 1 value missing from more than 20% of participants), then using the methods that require the MCAR assumption may give misleading results. In this situation, analyses of the immunogenicity

endpoints at a specific timepoint will be performed using parametric generalized linear models fit by maximum likelihood. These methods provide unbiased estimation and inferences under the parametric modeling assumptions and the assumption that the missing data are missing at random (MAR). MAR assumes that the probability of an observation being missing depends upon the observed responses and upon observed covariates, but not upon any unobserved factors. Generalized linear models for response rates will use a binomial error distribution and for quantitative endpoints, a normal error distribution. For assessing repeated immunogenicity measurement, linear mixed effects models will be used. If the immunological outcomes are left- and/or right- censored, then the linear mixed effects models of Hughes [56] will be used, because they accommodate the censoring. In addition, secondary analyses of repeated immunogenicity measurements will be done using weighted GEE [57] methods, which are valid under MAR. All of the models described above will include as covariates all available baseline predictors of the missing outcomes.

6.4.5 Analyses prior to end of study

Safety

During the course of the trial, unblinded analyses of safety data will be prepared approximately every 4 months for review by the SMB. Unblinded ad hoc safety reports may also be prepared for SMB review at the request of the PSRT. The HVTN leadership must approve any other requests for unblinded safety data prior to the end of the study.

Immunogenicity

An unblinded statistical analysis by treatment assignment of an immunogenicity endpoint may be performed when the Laboratory Program has completed testing of at least 80% of samples from the primary immunogenicity visit and all participants have completed the visit. The Laboratory Program will review the analysis report prior to distribution to the protocol chairs, DAIDS, vaccine developer, and other key HVTN members and investigators. Distribution will be limited to those with a need to know for the purpose of informing future trial-related decisions. The HVTN leadership must approve any other requests for HVTN immunogenicity analyses prior to the end of the study. Any analyses conducted prior to the end of the study should not compromise the integrity of the trial in terms of participant retention or safety or immunogenicity endpoint assessments.

7 Selection and withdrawal of participants

Participants will be HIV-uninfected (seronegative) healthy vaccinia-näive adults in South Africa who comprehend the purpose of the study and have provided written informed consent. Volunteers will be recruited and screened; those determined to be eligible, based on the inclusion and exclusion criteria, will be enrolled in the study and followed with scheduled clinic visits for a period of 12 months. Final eligibility determination will depend on results of laboratory tests, medical history, physical examinations, and answers to self-administered and/or interview questions. After completion of scheduled clinic visits participants will be contacted annually for purposes of extended safety surveillance for an additional 2 years.

Investigators should always use good clinical judgment in considering a volunteer's overall fitness for trial participation. Some volunteers may not be appropriate for enrollment even if they meet all inclusion/exclusion criteria. Medical, psychiatric, occupational, or other conditions may make evaluation of safety and/or immunogenicity difficult, and some volunteers may be poor candidates for retention. There is some evidence that pre-immunity to poxvirus vectors results in lower immune responses. Therefore, there is a risk of blunted response in participants that have immunity to vaccinia virus and for the purpose of this trial, people with history of vaccinia vaccination are excluded.

Determination of eligibility, taking into account all inclusion and exclusion criteria, must be made within 56 days prior to enrollment unless otherwise noted in sections 7.1 and 7.2.

7.1 Inclusion criteria

1. **Age** of 18 to 45 years

2. **Access to a participating HVTN CRS** and willingness to be followed for the planned duration of the study

3. Ability and willingness to provide **informed consent**

4. **Assessment of understanding:** volunteer demonstrates understanding of this study and other relevant study results and completes a questionnaire prior to first vaccination with verbal demonstration of understanding of all questionnaire items answered incorrectly

5. Willingness to receive **HIV test results**

6. Willingness to discuss **HIV infection risks**, amenable to **HIV risk reduction counseling,** and committed to maintaining behavior consistent with low risk of HIV exposure through the last required protocol clinic visit

7. Assessed by clinic staff as being at **"low risk" for HIV infection** on the basis of sexual behavior within the 12 months prior to enrollment defined as follows:

- Sexually abstinent, or
- In a mutually monogamous relationship with a partner with a known HIV-uninfected status, or
- Had one partner believed to be HIV-uninfected with whom he/she regularly used condoms for vaginal and anal intercourse

8. **Willing to be contacted annually** after completion of scheduled clinic visits for a total of 3 years following initial study injection
9. **Agrees not to enroll in another study of an investigational research agent** prior to completion of last required protocol clinic visit (excludes annual contacts for safety surveillance)
10. **Good general health** as shown by medical history, physical exam, and screening laboratory tests
11. **Hemoglobin** ≥ 11.0 g/dL for volunteers who were born female, ≥ 13.0 g/dL for volunteers who were born male
12. **White blood cell (WBC) count** = 3,300 to 12,000 cells/mm^3
13. **Total lymphocyte count** ≥ 800 cells/mm^3
14. **Platelets** = 125,000 to 550,000/mm^3
15. **Chemistry panel**: Alanine aminotransferase (ALT), aspartate aminotransferase (AST), and alkaline phosphatase < 1.25 times the institutional upper limit of normal
16. **Cardiac Troponin T (cTnT)** does not exceed the institutional upper limit of normal
17. **Negative HIV-1 and -2 blood test:** Sites may use locally available assays that have been approved by HVTN Laboratory Operations.
18. **Negative Hepatitis B surface antigen** (HBsAg)
19. **Negative anti-Hepatitis C virus antibodies** (anti-HCV), or negative HCV PCR if the anti-HCV is positive
20. **Normal urine:**
    - Negative urine glucose, and
    - Negative or trace urine protein, and
    - Negative or trace urine hemoglobin (if trace hemoglobin is present on dipstick, a microscopic urinalysis within institutional normal range).

21. **Volunteers who were born female**: negative serum or urine beta human chorionic gonadotropin (β-HCG) pregnancy test performed prior to vaccination on the day of initial vaccination

22. **Reproductive status**: A volunteer who was born female must:

    - Agree to consistently use effective contraception, from at least 21 days prior to enrollment through 90 days after the participant's last vaccination, for sexual activity that could lead to pregnancy. Effective contraception for participants in South Africa is defined as using 2 methods, including 1 of the following:
        - Condoms (male or female) with or without a spermicide,
        - Diaphragm or cervical cap with spermicide,

    PLUS 1 of the following methods:
        - Intrauterine device (IUD),
        - Hormonal contraception, or
        - Successful vasectomy in the male partner (considered successful if a volunteer reports that a male partner has [1] documentation of azoospermia by microscopy, or [2] a vasectomy more than 2 years ago with no resultant pregnancy despite sexual activity postvasectomy);

    - Or not be of reproductive potential, such as having reached menopause (no menses for 1 year) or having undergone hysterectomy, bilateral oophorectomy, or tubal ligation;

    - Or be sexually abstinent.

23. **Volunteers who were born female must also agree not to seek pregnancy through alternative methods,** such as artificial insemination or in vitro fertilization until after the last required protocol clinic visit

7.2 Exclusion criteria

1. **Vaccinia (smallpox) vaccination** determined by: (1) clinical evidence of vaccinia scarification; (2) self-reported history of vaccinia vaccination; or (3) birth year 1977 or earlier. (Not excluded: a participant born in or before 1977 who self reports he/she did not receive vaccinia [smallpox] vaccination and has no evidence of scarification.)

2. **Within the 12 months prior to enrollment:** excessive daily alcohol use or frequent binge drinking or chronic marijuana abuse or any other use of illicit drugs

3. **Within the 12 months prior to enrollment:** a history of newly acquired syphilis, gonorrhea, non-gonococcal urethritis, herpes simplex virus type 2 (HSV2), Chlamydia, pelvic inflammatory disease (PID), trichomonas, mucopurulent cervicitis, epididymitis, proctitis, lymphogranuloma venereum, chancroid, or hepatitis B

4. **Untreated or incompletely treated syphilis infection**

5. **HIV vaccine(s)** received in a prior HIV vaccine trial. For potential participants who have received control/placebo in an HIV vaccine trial, the HVTN 086 / SAAVI 103 PSRT will determine eligibility on a case-by-case basis.

6. **Non-HIV experimental vaccine(s)** received within the last 5 years in a prior vaccine trial. Exceptions may be made for vaccines that have subsequently undergone licensure by the FDA or MCC. For potential participants who have received control/placebo in an experimental vaccine trial, the HVTN 086 / SAAVI 103 PSRT will determine eligibility on a case-by-case basis. For potential participants who have received an experimental vaccine(s) greater than 5 years ago, eligibility for enrollment will be determined by the PSRT on a case-by-case basis.

7. **Immunosuppressive medications** received within 168 days before first vaccination. (Not excluded: [1] corticosteroid nasal spray for allergic rhinitis; [2] topical corticosteroids for mild, uncomplicated dermatitis; or [3] oral/parenteral corticosteroids given for non-chronic conditions not expected to recur [length of therapy 10 days or less with completion at least 30 days prior to enrollment].)

8. **Blood products** received within 120 days before first vaccination

9. **Immunoglobulin** received within 60 days before first vaccination

10. **Live attenuated vaccines** (not including influenza vaccine) received within 30 days before first vaccination or scheduled within 14 days after injection (eg, measles, mumps, and rubella [MMR]; oral polio vaccine [OPV]; varicella; yellow fever)

11. **Influenza vaccine or any vaccines that are not live attenuated vaccines** and were received within 14 days prior to first vaccination (eg, tetanus, pneumococcal, Hepatitis A or B)

12. **Investigational research agents** received within 30 days before first vaccination or scheduled within 14 days after vaccination

13. **Allergy treatment with antigen injections** within 30 days before first vaccination or that are scheduled within 14 days after first vaccination

14. **Current anti-tuberculosis (TB) prophylaxis or therapy**

15. **Clinically significant medical condition,** physical examination findings, clinically significant abnormal laboratory results, or past medical history with clinically significant implications for current health. A clinically significant condition or process includes but is not limited to:

    - A process that would affect the immune response,
    - A process that would require medication that affects the immune response,
    - Any contraindication to repeated injections or blood draws,
    - A condition that requires active medical intervention or monitoring to avert grave danger to the participant’s health or well-being during the study period,

- A condition or process for which signs or symptoms could be confused with reactions to vaccine, or

- Any condition specifically listed among the exclusion criteria below.

16. **Any medical, psychiatric, occupational, or other condition** that, in the judgment of the investigator, would interfere with, or serve as a contraindication to, protocol adherence, assessment of safety or reactogenicity, or a participant's ability to give informed consent.

17. **Serious adverse reactions to vaccines** including anaphylaxis and related symptoms such as hives, respiratory difficulty, angioedema, and/or abdominal pain. (Not excluded: a participant who had a nonanaphylactic adverse reaction to pertussis vaccine as a child.)

18. **Hypersensitivity to eggs or egg products**

19. **ECG with clinically significant findings,** or features that would interfere with the assessment of myo/pericarditis, as determined by a contract ECG laboratory or cardiologist, including any of the following: (1) conduction disturbance (complete left or complete right bundle branch block or nonspecific intraventricular conduction disturbance with QRS ≥120ms, AV block of any degree, or QTc prolongation (> 440ms); (2) repolarization (ST segment or T wave) abnormality; (3) significant atrial or ventricular arrhythmia; (4) frequent atrial or ventricular ectopy (eg, frequent premature atrial contractions, 2 premature ventricular contractions in a row); (5) ST elevation consistent with ischemia; (6) evidence of past or evolving myocardial infarction.

20. **Cardiac risk factors,** including 2 or more of the following: (1) participant report of history of elevated blood cholesterol defined as fasting low density lipoprotein (LDL) > 160 mg/dL; (2) first degree relative (eg, mother, father, brother, or sister) who had coronary artery disease before the age of 50 years; (3) current smoker; or (4) BMI ≥ 35.

21. **History of, or known active cardiac disease** including: (1) previous myocardial infarction (heart attack); (2) angina pectoris; (3) congestive heart failure; (4) valvular heart disease including mitral valve prolapse; (5) cardiomyopathy; (6) pericarditis; (7) stroke or transient ischemic attack; (8) chest pain or shortness of breath with activity (such as walking up stairs); (9) dysrhythmia/episodic palpitations (Not excluded: sinus arrhythmia); or (10) other heart conditions under the care of a doctor.

22. **Autoimmune disease**

23. **Immunodeficiency**

24. **Asthma** other than mild, well-controlled asthma. Exclude a participant who:

    - Generally uses a bronchodilator ($beta_2$ agonist) daily, or

    - In the past year, has (any of the following):

- Had > 1 exacerbation of symptoms treated with oral steroids (Note: oral/parenteral steroid use for asthma is exclusionary within 168 days before first vaccination.);
- Routinely used moderate to high dose inhaled corticosteroids (eg, more than the equivalent of 250 mcg fluticasone; 400 mcg budesonide; 500 mcg beclomethasone; or 1000 mcg triamcinolone/flunisolide, as a daily dose) or theophylline for asthma; or
- Needed emergency care, urgent care, hospitalization, or intubation for asthma.

25. **Diabetes mellitus** type 1 or type 2, including cases controlled with diet alone. (Not excluded: history of isolated gestational diabetes.)

26. **Thyroidectomy, or thyroid disease** requiring medication during the last 12 months

27. **Angioedema** within the last 3 years if episodes are considered serious or have required medication within the last 2 years

28. **Hypertension:**

    - If a person has been diagnosed with hypertension during screening or previously, exclude for hypertension that is not well controlled. Well-controlled hypertension is defined as blood pressure consistently ≤ 140 mm Hg systolic and ≤ 90 mm Hg diastolic, with or without medication, with only isolated, brief instances of higher readings, which must be ≤ 150 mm Hg systolic and ≤ 100 mm Hg diastolic. For these participants, blood pressure must be ≤ 140 mm Hg systolic and ≤ 90 mm Hg diastolic at enrollment.

    - If a person has NOT been diagnosed with hypertension during screening or previously, exclude for systolic blood pressure ≥ 150 mm Hg at enrollment or diastolic blood pressure ≥ 100 mm Hg at enrollment.

29. **Body mass index (BMI)** ≥ 40

30. **Bleeding disorder** diagnosed by a doctor (eg, factor deficiency, coagulopathy, or platelet disorder requiring special precautions)

31. **Malignancy** (Not excluded: a participant with a surgical excision and subsequent observation period that in the investigator's estimation has a reasonable assurance of sustained cure or is unlikely to recur during the period of the study.)

32. **Seizure disorder** (Not excluded: a participant with a history of seizures who has not required medications or had a seizure within the past 3 years.)

33. **Asplenia**: any condition resulting in the absence of a functional spleen

34. **Psychiatric condition** that precludes compliance with the protocol. Specifically excluded are persons with psychoses within the past 3 years, ongoing risk for suicide, or history of suicide attempt or gesture within the past 3 years.

35. **Pregnant or breastfeeding**

7.3 Participant departure from vaccination schedule or withdrawal

This section concerns an individual participant's departure from the vaccination schedule. Pause rules for the trial as a whole are described in section 11.4.

7.3.1 Delaying vaccinations for a participant

Under certain circumstances, a participant's scheduled vaccination will be delayed. The factors to be considered in such a decision include but are not limited to the following:

- Live attenuated vaccines other than influenza vaccine received within 30 days prior to any vaccination,
- Influenza vaccine or any vaccines that are not live attenuated vaccines (eg, tetanus, pneumococcal) and were received within 14 days prior to any vaccination,
- Allergy treatment with antigen injections within 30 days prior to any study injection,
- Blood products or immunoglobulin received within 45 days prior to any vaccination, or
- Prevaccination abnormal vital signs or clinical symptoms that may mask assessment of vaccine reaction.

Vaccinations should not be administered outside the visit window period specified in the *HVTN 086 / SAAVI 103 Study Specific Procedures*.

In order to avoid vaccination delays and missed vaccinations, participants who plan to receive medically indicated or elective vaccinations, allergy treatments, or other investigational research agents should be counseled to schedule receipt of these substances outside the intervals indicated above, when possible. Because their effects on safety and immunogenicity assessments and their interactions with study vaccines are unknown, unless urgently needed, these substances should also be avoided in the 2-week interval between a study vaccination and completion of the next scheduled postvaccination follow-up visit.

7.3.2 Participant departure from vaccination schedule

Every effort should be made to follow the vaccination schedule per the protocol. If a participant misses a vaccination and the visit window period for the vaccination has passed, that vaccination cannot be given. The participant should be asked to continue study visits. A participant who has missed only 1 vaccination should resume the vaccination schedule with the next vaccination unless there are circumstances that require further delay or permanent discontinuation of vaccination (see sections 7.3.1 and 7.3.3). If a participant misses more than 1 vaccination, the participant's vaccinations will be permanently discontinued.

7.3.3 Discontinuing vaccination for a participant

Under certain circumstances, an individual participant's vaccinations will be permanently discontinued. Specific events that will result in stopping a participant's vaccination schedule include:

- Co-enrollment in a study with an investigational research agent (rare exceptions allowing for the continuation of vaccinations may be granted with the unanimous consent of the PSRT).

- Clinically significant condition (ie, a condition that affects the immune system or for which continued vaccinations and/or blood draws may pose additional risk), including but not limited to the following:
  - Pregnancy (regardless of outcome);
  - Any grade 4 local or systemic reactogenicity symptom, lab abnormality, or AE that is judged to have a suspected causal relationship to study vaccination;
  - Any grade 3 lab abnormality or other clinical AE (exception: fever or vomiting and subjective local and systemic symptoms) that is judged to have a suspected causal relationship to study vaccination; or
  - Clinically significant type 1 hypersensitivity reaction associated with study vaccination. Consultation with the HVTN 086 / SAAVI 103 PSRT is required prior to subsequent vaccinations following any type 1 hypersensitivity reaction associated with study vaccination.

- Investigator determination in consultation with Protocol Team leadership (eg, for repeated nonadherence to study staff instructions).

- Participant has missed 2 scheduled vaccinations and the visit window period for the second missed vaccination has passed.

Such participants should be counseled on the importance of continuing with the study and strongly encouraged to participate in follow-up visits and protocol-related procedures (unless medically contraindicated) per the protocol for the remainder of the trial.

In addition, vaccinations will be stopped for participants diagnosed with HIV infection. HIV-infected participants will not continue in the trial (see sections 7.3.4 and 9.7).

7.3.4 Participant termination from the study

Under certain circumstances, an individual participant may be terminated from participation in this study. Specific events that will result in early termination include:

- Participant refuses further participation,

- Participant relocates and remote follow-up or transfer to another HVTN CRS is not possible,

- HVTN CRS determines that the participant is lost to follow-up,

- Participant becomes HIV infected, or
- Investigator decides, in consultation with Protocol Team leadership, to terminate participation (eg, if participant exhibits inappropriate behavior toward clinic staff).

8 Study product preparation and administration

CRS pharmacists should consult the *Pharmacy Guidelines and Instructions for DAIDS Clinical Trials Networks* for standard pharmacy operations. The protocol schema is shown in Table 3-1. See the IBs for further information about study products.

8.1 Vaccine regimen

<u>Group 1</u>

Treatment 1 (T1):

Month 0

SAAVI MVA-C 1.45×10^9 pfu administered as 0.5 mL IM in RIGHT deltoid

AND

Placebo for Sub C gp140/MF59C.1 administered as 0.5 mL IM in LEFT deltoid

Month 1

SAAVI MVA-C 1.45×10^9 pfu administered as 0.5 mL IM in RIGHT deltoid

Month 3 and 6

Placebo for SAAVI MVA-C administered as 0.5 mL IM in RIGHT deltoid

AND

Sub C gp140 100 mcg admixed with MF59C.1 administered as 0.5 mL IM in LEFT deltoid

Placebo 1 (P1):

Month 0

Placebo for SAAVI MVA-C administered as 0.5 mL IM in RIGHT deltoid

AND

Placebo for Sub C gp140/MF59C.1 administered as 0.5 mL IM in LEFT deltoid

Month 1

Placebo for SAAVI MVA-C administered as 0.5 mL IM in RIGHT deltoid

Month 3 and 6

Placebo for SAAVI MVA-C administered as 0.5 mL IM in RIGHT deltoid

AND

Placebo for Sub C gp140/MF59C.1 administered as 0.5 mL IM in LEFT deltoid

__**Group 2**__

Treatment 2 (T2):

Month 0:

SAAVI MVA-C 1.45×10^9 pfu administered as 0.5 mL IM in RIGHT deltoid

AND

Sub C gp140 100 mcg admixed with MF59C.1 administered as 0.5mL IM in LEFT deltoid

Month 1:

Placebo for SAAVI MVA-C administered as 0.5 mL IM in RIGHT deltoid

Month 3:

SAAVI MVA-C 1.45×10^9 pfu administered as 0.5 mL IM in RIGHT deltoid

AND

Sub C gp140 100 mcg admixed with MF59C.1 administered as 0.5mL IM in LEFT deltoid

Month 6:

Placebo for SAAVI MVA-C administered as 0.5 mL IM in RIGHT deltoid

AND

Placebo for Sub C gp140/MF59C.1 administered as 0.5 mL IM in LEFT deltoid

Placebo 2 (P2):

Month 0:

Placebo for SAAVI MVA-C administered as 0.5 mL IM in RIGHT deltoid

AND

Placebo for Sub C gp140/MF59C.1 administered as 0.5 mL in LEFT deltoid

Month 1:

Placebo for SAAVI MVA-C administered as 0.5 mL IM in RIGHT deltoid

Months 3 and 6:

Placebo for SAAVI MVA-C administered as 0.5 mL IM in RIGHT deltoid

AND

Placebo for Sub C gp140/MF59C.1 administered as 0.5 mL in LEFT deltoid

<u>Group 3:</u>

Treatment 3 (T3):

Month 0:

SAAVI DNA-C2 4mg administered as 1 mL in RIGHT deltoid

AND

Placebo for Sub C gp140/MF59C.1 administered as 0.5 mL in LEFT deltoid

Month 1:

SAAVI DNA-C2 4mg administered as 1 mL in RIGHT deltoid

Months 3 and 6:

SAVVI MVA-C 1.45 x 10^9 pfu administered as 0.5 mL IM in RIGHT deltoid

AND

Placebo for Sub C gp140/MF59C.1 administered as 0.5 mL in LEFT deltoid

Placebo 3 (P3):

Month 0:

Placebo for SAAVI DNA-C2 administered as 1 mL IM in RIGHT deltoid

AND

Placebo for Sub C gp140/MF59C.1 administered as 0.5 mL in LEFT deltoid

Month 1:

Placebo for SAAVI DNA-C2 administered as 1 mL IM in RIGHT deltoid

Months 3 and 6:

Placebo for SAAVI MVA-C administered as 0.5 ml IM in RIGHT deltoid

AND

Placebo for Sub C gp140/MF59C.1 administered as 0.5 mL in LEFT deltoid

<u>Group 4:</u>

Treatment 4 (T4):

Month 0:

SAAVI DNA-C2 4mg administered as 1 mL in RIGHT deltoid

AND

Placebo for Sub C gp140/MF59C.1 administered as 0.5 mL in LEFT deltoid

Month 1:

SAAVI DNA-C2 4mg administered as 1mL in RIGHT deltoid

Months 3 and 6:

SAAVI MVA-C 1.45 x 10^9 pfu administered as 0.5 mL IM in RIGHT deltoid

AND

Sub C gp140 100 mcg admixed with MF59 administered as 0.5mL IM in LEFT deltoid

Placebo 4 (P4):

Month 0:

Placebo for SAAVI DNA-C2 administered as 1 mL IM in RIGHT deltoid

AND

Placebo for Sub C gp140/MF59C.1 administered as 0.5 mL in LEFT deltoid

Month 1:

Placebo for SAAVI DNA-C2 administered as 1 mL IM in RIGHT deltoid

Months 3 and 6:

Placebo for SAAVI MVA-C administered as 0.5 ml IM in RIGHT deltoid

AND

Placebo for Sub C gp140/MF59C.1 administered as 0.5 mL in LEFT deltoid

8.2 Study product formulation

The study products are described in further detail in the IB.

8.2.1 SAAVI DNA-C2 (labeled as SAAVI DNA-C2 Vaccine 4.0 mg/mL):

SAAVI DNA-C2 vaccine was manufactured by Althea Technologies, Inc. (San Diego, California, USA). The product is provided in 2 mL clear glass vials containing 1.3 mL of a clear, colorless, aqueous DNA solution at a concentration of 4 mg/mL in Tris-buffered saline (10 mM Tris HCl, 1 mM EDTA, 0.9% sodium chloride). The product must be stored at –25°C to –15°C.

8.2.2 Placebo for SAAVI DNA-C2 (Sodium Chloride for Injection, 0.9%):

Sodium Chloride for Injection, 0.9% will be used as the placebo. Product must be stored as directed by the manufacturer.

8.2.3 SAAVI MVA-C (labeled as SAAVI rMVA-C TBC-M456 Vaccine For 10^9 dose):

SAAVI MVA-C vaccine was manufactured by Therion Biologics Corporation (Cambridge, Massachusetts, USA). The product is supplied in 2 mL glass vials containing 0.75 mL of vaccine at a titer of 2.9×10^9 pfu/mL as a frozen, milky to clear solution that may contain clumps or aggregates. The product must be stored at –70°C or colder.

8.2.4 Placebo for SAAVI MVA-C (Sodium Chloride for Injection 0.9%)

Sodium Chloride for Injection, 0.9% will be used as the placebo. Product must be stored as directed by the manufacturer.

8.2.5 Novartis subtype C gp140 (labeled as Sub C gp140)

Novartis Sub C gp140 vaccine was manufactured by Novartis Vaccine and Diagnostics, Inc. The vaccine is a clear colorless to slightly yellow liquid and is supplied in a 3 mL glass vial containing 0.4mg/ml of gp140 in 0.35 mL. The product must be stored at -60^0C (or colder).

8.2.6 Placebo for Sub C gp140/MF59C.1 (Sodium Chloride for Injection, 0.9%)

Sodium Chloride for Injection, 0.9% will be used as the placebo. Product must be stored as directed by the manufacturer.

8.2.7 Novartis MF59C.1 Adjuvant (labeled as MF59C.1)

Novartis MF59C.1 was manufactured by Novartis Vaccines. MF59C.1 adjuvant is an oil-in-water emulsion with a squalene internal oil phase and a citrate buffer external aqueous phase. Two non-ionic surfactants, sorbitan trioleate and polysorbate 80, serve to stabilize the emulsion. The product is supplied in a 5 mL glass vial containing 1.9 mL MF59C.1 per vial. The product must be stored refrigerated between 2°C to 8°C. Do not store at room temperature for longer than 8 hours. Protect from light. Do not freeze.

8.3 Preparation of study products

8.3.1 SAAVI DNA C-2 (labeled as SAAVI DNA-C2 Vaccine 4.0 mg/mL)

On the day of administration, the pharmacist will remove 1 vial from the –20°C freezer and allow the product to thaw at room temperature. The vaccine can be thawed by handholding. Once thawed, the pharmacist, using aseptic technique will withdraw 1 mL into a syringe. Prior to dispensing, the pharmacist will place an overlay on the syringe. The product must be administered as soon as possible after preparation. Each syringe should be labeled as directed in the HVTN 086 / SAAVI 103 Site Specific Procedures (SSP).

Any unused portion of entered vials or expired prefilled syringes should be disposed of in accordance with institutional or pharmacy policy.

8.3.2 Placebo for SAAVI DNA-C2 (Sodium Chloride for Injection 0.9%)

Using aseptic technique, the pharmacist will withdraw 1 mL of Sodium Chloride for Injection 0.9% into a syringe. Prior to dispensing the pharmacist will place an overlay on the syringe. The product must be administered as soon as possible after preparation. Each syringe should be labeled as directed in the HVTN 086 / SAAVI 103 SSP.

Any unused portion of entered vials or expired prefilled syringes should be disposed of in accordance with institutional or pharmacy policy.

8.3.3 SAAVI MVA-C (labeled as SAAVI rMVA-C TBC-M456 Vaccine For 10^9 dose)

On the day of administration, the pharmacist will remove 1 vial from the –70°C freezer and allow the product to thaw at room temperature in an upright position. Once thawed, the contents in the vial must be vortexed vigorously for 30 seconds using gentle contact pressure and observing afterwards to assure that any possible aggregates are dispersed and the contents of the vial appear opaque to clear without visible floating objects. The pharmacist should tap the top of the vial to ensure the contents are at the bottom of the upright vial. Using aseptic technique, the pharmacist will withdraw 0.5 mL into a syringe.

Prior to dispensing the pharmacist will place an overlay on the syringe. The product must be administered as soon as possible after preparation.

Each syringe should be labeled as directed in the HVTN 086 / SAAVI 103 SSP.

Any unused portion of entered vials or expired prefilled syringes should be disposed of in accordance with institutional or pharmacy policy.

8.3.4 Placebo for SAAVI MVA-C (Sodium Chloride for Injection, 0.9%)

Using aseptic technique, the pharmacist will withdraw 0.5 mL of Sodium Chloride for Injection, 0.9% into a syringe. Prior to dispensing the pharmacist will place an overlay on the syringe. The product must be administered as soon as possible after preparation. Each syringe should be labeled as directed in the HVTN 086 / SAAVI 103 SSP.

Any unused portion of entered vials or expired prefilled syringes should be disposed of in accordance with institutional or pharmacy policy.

8.3.5 Novartis Sub C gp140/MF59C.1

On the day of administration, the pharmacist will remove the Novartis gp140 vaccine from the freezer and allow the product to thaw at room temperature for 30 minutes. The pharmacist will also remove the MF59C.1 vial from the refrigerator and mix by repeated gentle swirling and inversion (do not shake vigorously). Using aseptic technique, the pharmacist will withdraw 0.35 mL of MF59 into a 1 mL sterile syringe, inject the MF59C.1 into the 3 mL vial containing the thawed gp140 vaccine, and mix by gentle swirling. The pharmacist will then withdraw 0.5 mL of the mixture into a syringe. Prior to dispensing, the pharmacist will place an overlay on the syringe. The admixed vaccine may be stored at room temperature and administered within 8 hours after addition of MF59C.1.

Any unused portion of entered vials or expired prefilled syringes should be disposed of in accordance with institutional or pharmacy policy.

8.3.6 Placebo for Sub C gp140/MF59C.1 (Sodium Chloride for Injection, 0.9%)

Using aseptic technique, the pharmacist will withdraw 0.5 mL of Sodium Chloride for Injection, 0.9% into a syringe. Prior to dispensing, the pharmacist will place an overlay on the syringe. The product must be administered as soon as possible after preparation. Each syringe should be labeled as directed in the HVTN 086 / SAAVI 103 SSP.

Any unused portion of entered vials or expired prefilled syringes should be disposed of in accordance with institutional or pharmacy policy

8.3.7 Procedures to preserve blinding

The pharmacist will prepare all doses for administration and dispense to the clinic. In order to preserve blinding, the pharmacist will place an overlay on ALL the syringes.

8.4 Administration

All injections should be administered IM in the deltoid indicated.

At sites where registered pharmacists are legally authorized to administer drug, the HVTN CRS may choose to have the pharmacist administer the vaccinations.

When preparing a dose in a syringe and administering the dose, consideration should be given to the volume of solution in the needle before and after the dose is administered. Consideration should be given to conserving the full dose of product. The pharmacy and clinic staff members are encouraged to work together to administer the dose specified in the protocol.

8.5 Acquisition of study products

SAAVI DNA-C2 and SAAVI MVA-C will be provided by SAAVI, MRC–SA. Sodium Chloride for Injection, 0.9% will not be provided through the protocol but must be obtained by the site.

Once an HVTN CRS is protocol registered, the pharmacist can obtain the SAAVI DNA-C2 vaccine, SAAVI MVA-C2 vaccine, Novartis Sub C gp140 vaccine, Novartis MF59C.1 adjuvant, and overlays for the syringes from the NIAID Clinical Research Products Management Center (CRPMC) by following the ordering procedures given in *Pharmacy Guidelines and Instructions for DAIDS Clinical Trials Networks*.

8.6 Pharmacy records

The HVTN CRS pharmacist is required to maintain complete records of all study products. The pharmacist of record is responsible for maintaining randomization codes and randomization confirmation notices for each participant in a secure manner.

8.7 Final disposition of study products

All unused study products must be returned to the CRPMC after the study is completed or terminated unless otherwise instructed. The procedures and relevant form are included in the *Pharmacy Guidelines and Instructions for DAIDS Clinical Trials Networks*.

9 Clinical procedures

The schedule of clinical procedures is shown in Appendix F.

9.1 Informed consent

Informed consent is the process of ensuring that participants fully understand what will and may happen to them while participating in a research study. The HVTN informed consent form documents that a participant (1) has been informed about the potential risks, benefits, and alternatives to participation, and (2) is willing to participate in an HVTN study. Informed consent encompasses all written or verbal study information HVTN CRS staff provide to the participant, before and during the trial. HVTN CRS staff will obtain informed consent of participants according to HVTN policies and procedures.

The informed consent process continues throughout the study. Key study concepts should be reviewed periodically with the participant and the review should be documented. At each study visit, HVTN CRS staff should consider reviewing the procedures and requirements for that visit and for the remaining visits. Additionally, if any new information is learned that might affect the participants' decisions to stay in the trial, this information will be shared with trial participants. If necessary, participants will be asked to sign revised informed consent forms.

An HVTN CRS may employ recruitment efforts prior to the participant consenting. For example, some HVTN CRSs use a telephone script to prescreen people before they come to the clinic for a full screening visit. Participants must sign a screening or protocol-specific consent before any procedures are performed to determine eligibility. HVTN CRSs must submit recruitment and prescreening materials to IRBs/IECs for human subjects protection review and approval.

9.1.1 Screening consent form

Some HVTN CRSs have approval from their local IRB or IEC to use a general screening consent form that allows screening for an unspecified HIV vaccine trial. In this way, HVTN CRS staff can continually screen potential participants and, when needed, proceed quickly to obtain protocol-specific enrollment consent. Sites conducting IRB/IEC-approved general screening or prescreening may use the results from this screening to determine eligibility for this protocol, provided the tests are conducted within the time periods specified in the eligibility criteria. Without a general screening consent, screening for a specific study cannot take place until the site is activated by HVTN Regulatory Affairs.

9.1.2 Protocol-specific consent forms

The protocol-specific consent forms describe the study products to be used and all aspects of protocol participation, including screening and enrollment procedures.

- A sample protocol-specific consent form for the main study is located in Appendix A.

- A separate consent form for extended safety surveillance following the main study is located in Appendix B.

Each HVTN CRS is responsible for developing protocol-specific consent forms for local use, based on the sample protocol-specific consent forms in Appendix A and Appendix B. The consent forms must be developed in accordance with local IRB/IEC requirements and the principles of informed consent as described in Title 45, CFR Part 46, in the International Conference on Harmonisation (ICH) E6, Good Clinical Practice (GCP): Consolidated Guidance 4.8, and South African GCP.

Study sites are strongly encouraged to have their local CABs review the sites' protocol-specific consent forms. This review should include, but should not be limited to, issues of cultural competence, local language considerations, and the level of understandability.

The sample informed consent form includes instructions throughout the document for developing specific content.

Prior to implementing version 1 of this protocol, each site must have the protocol and site-specific protocol consent forms approved by its IRB/EC and any other applicable Regulatory Entity (RE). Prior to site activation, site-specific informed consent forms will be reviewed and approved by HVTN Regulatory Affairs.

Regarding protocol registration, sites should follow procedures outlined in the current version of the DAIDS Protocol Registration Manual.

9.1.3 VISP registry consent form

Experimental HIV vaccines may induce antibody production to HIV antigens, producing reactive results on commercially available HIV test kits. This is called "vaccine-induced seropositivity" (VISP) (see Section 9.7.1). In order to provide post-study HIV testing to distinguish between VISP and HIV infection, and to mitigate potential social harms resulting from VISP in HIV vaccine recipients who are not infected with HIV, the HVTN has created a VISP registry. Following study unblinding, the registry will allow HVTN counselors to verify that an individual has received an HIV vaccine, and therefore has the potential for VISP. Information in the VISP registry will not be used for research. Rather, the registry exists to support provision of post-study testing and counseling services to HIV vaccine recipients.

The VISP registry consent form describes the purpose of the VISP registry, the participant information to be included in the registry, confidentiality protections, and risks and benefits associated with inclusion in the registry. The VISP registry consent form is contained in Appendix J.

The VISP Registry consent form will be presented to all participants. It is recommended to be presented no later than the last scheduled vaccination visit.

9.1.4 Assessment of Understanding

Study staff should ensure that participants fully understand the study before enrolling them. This process involves reviewing the informed consent form with the participant,

allowing time for the participant to reflect on the procedures and issues presented, and answering all questions completely.

An Assessment of Understanding is used to document the participant's understanding of key concepts in this HIV vaccine trial, including information about the results of other relevant studies. The participant must complete the Assessment of Understanding before enrollment. Staff may provide assistance in reading and understanding the questions and responses, if necessary. Participants must verbalize understanding of all questions answered incorrectly. This process and the participant's understanding of the key concepts should be recorded in source documentation at the site.

IRBs/IECs may require that a participant has signed either a screening or protocol-specific consent document prior to administering the Assessment of Understanding. The consent process (including the use of the Assessment of Understanding) should be explained thoroughly to the IRB/IEC, whose recommendations should be followed.

9.2 Pre-enrollment procedures

Screening procedures are done to determine eligibility and to provide a baseline for comparison of safety data. Baseline data are obtained during screening. Screening may occur over the course of several contacts/visits, up to and including before vaccination on day 0. All inclusion and exclusion criteria must be assessed within 56 days before enrollment, unless otherwise specified in the eligibility criteria (or below in this section).

After the appropriate informed consent has been obtained and before enrollment, the following procedures are performed:

- Medical history, documented in the case history record;
- Assessment of whether the volunteer is at low risk for HIV infection (as defined in Section 7.1);
- Complete physical examination, including height, weight, vital signs, and clinical assessments of head, ears, eyes, nose, and throat; neck; lymph nodes; heart; chest; abdomen; extremities; neurological function; and skin;
- Assessment of concomitant medications the volunteer is taking, including prescription and nonprescription drugs, vitamins, topical products, alternative/complementary/traditional medicines (e.g., herbal and health food supplements), recreational drugs, vaccinations, and allergy shots (record the complete generic name for all medications);
- Laboratory tests as defined in the inclusion and exclusion criteria, including:
    - Screening HIV test,
    - HBsAg,
    - Anti-HCV,
    - Syphilis test,
    - Complete blood count (CBC) with differential and platelets,

- Chemistry panel (ALT, AST, alkaline phosphatase, and creatinine [creatinine done at baseline to provide reference for potential future tests if indicated, but not part of eligibility criteria])
- Urine dipstick (as described in Section 9.9),
- Urine or serum pregnancy test (participants who were born female),
- cTnT, and
- 12-lead ECG with interpretation;

- Administration of behavioral risk assessment questionnaire;
- Obtaining of volunteer demographics in compliance with the *NIH Policy on Reporting Race and Ethnicity Data: Subjects in Clinical Research,* Aug. 8, 2001 (available at http://grants.nih.gov/grants/guide/notice-files/NOT-OD-01-053.html);
- Counseling on HIV testing and risk reduction, performed in compliance with the US Centers for Disease Control and Prevention (CDC)'s current guidelines or other local guidelines for HIV counseling, testing, and referral; and
- Discussion of pregnancy prevention. A pregnant or breastfeeding person may not be enrolled in this trial. Specific criteria and assessment of contraception and pregnancy status are described in study inclusion criteria. Discussion of pregnancy prevention includes advising a participant who was born female and who reports no current sexual activity that could lead to that participant becoming pregnant to have a plan to begin adequate birth control. This plan would be put to use if, during the study, the participant becomes sexually active in a way that could lead to that participant becoming pregnant.

9.2.1 Use of screening results from another HVTN study

If a participant screens for an HVTN study at the same HVTN CRS but then does not join that study, screening results from that effort may be applied to the screening for this protocol, as long as the screening was done under participant consent, the participant has signed a consent form to begin screening for this study, and the tests were conducted within the time periods specified in the eligibility criteria (see sections 7.1 and 7.2).

9.3 Enrollment and vaccination visits

Enrollment is simultaneous with first vaccination. The time interval between randomization and enrollment should not exceed 4 working days. The HVTN CRS registers the participant by scheduling the day 0 visit (enrollment) via the Web-based randomization system, and requests the randomization assignment. Circumstances may require a participant's enrollment visit to be changed. This may exceed the 4-day randomization time limit.

At all vaccination visits, the following procedures are performed before vaccination:

- Abbreviated physical examination, including weight, vital signs, and a symptom-directed evaluation by history and/or appropriate physical exam based on participant self-reported symptoms or complaints;
- Assessment of baseline reactogenicity parameters;
- Assessment of concomitant medications (as described in section 9.2);
- Assessment of any new or unresolved AEs/intercurrent illnesses;
- Urine or serum pregnancy test (for participants who were born female); and
- Cardiac symptoms assessment.

Following completion of all procedures in the preceding list, and confirmation of results that indicate that vaccination may proceed, vaccination is prepared and administered (see sections 8.3 and 8.4).

Immediately following vaccination, the participant remains in the clinic for observation. An initial reactogenicity assessment is made at a target of 30 minutes after injection, with an acceptable range of 25-60 minutes. Before leaving the clinic, the participant is given the postvaccination symptom log and is instructed on how to complete it. The site will make arrangements to obtain daily reports of reactogenicity events from the participant during the reactogenicity period (as described in section 9.10).

The following procedures will be performed at all vaccination visits. These procedures may be performed prior to or following vaccination:

- Risk reduction counseling (as described in section 9.7);
- Pregnancy prevention assessment (as described in section 9.2 and 9.8);
- Assessment of new or unresolved social impacts (site staff will ask participant about the status of any unresolved social impacts and if s/he has experienced any new social impacts as a result of the trial participation); and
- Assessment of local and systemic reactogenicity symptoms (as described in Section 9.10).

Additional procedures will be performed at scheduled visits as specified in Appendix F:

- Administration of the social impact assessment questionnaire (types of impacts assessed involve personal relationships, medical insurance, life insurance, educational or employment opportunities, housing, immigration, or travel);
- Administration of a questionnaire that asks the participant about any HIV testing he or she may have received outside of the study. Participants will also be asked whether they believe they received the active vaccine or the control;
- Confirm that participants received HIV test results from previous visit. If not, provide test results and post-test counseling when appropriate; and

- Specimen collection.
- Note: for optional mucosal secretion sampling, see section 9.5

9.4 Follow-up visits

The following procedures are performed at all scheduled follow-up visits:

- Risk reduction counseling (as described in section 9.7);
- Pregnancy prevention assessment (as described in section 9.2 and 9.8);
- Assessment of new or unresolved social impacts (site staff will ask participant about the status of any unresolved social impacts and if s/he has experienced any new social impacts as a result of the trial participation);
- Assessment of new or continuing concomitant medications (as described in section 9.2);
- Assessment of new or unresolved AEs/intercurrent illnesses;
- Cardiac symptoms assessment (as described in section 9.4.1); and
- Specimen collection.

Additional procedures will be performed at scheduled follow-up visits as specified in Appendix F:

- Administration of the social impact assessment questionnaire (types of impacts assessed involve personal relationships, medical insurance, life insurance, educational or employment opportunities, housing, immigration, or travel);
- Administration of a questionnaire that asks the participant about any HIV testing he or she may have received outside of the study. Participants will also be asked whether they believe they received the active vaccine or the control;
- HIV infection assessment including pre-test counseling. A subsequent follow-up contact is conducted to provide post-test counseling and to report results to participant;
- Confirm that participants received HIV test results from previous visit. If not, provide test results and post-test counseling as appropriate;
- Abbreviated physical examination including weight, vital signs, and a symptom-directed evaluation by history and/or appropriate physical exam based on participant self-reported symptoms or complaints;
- Complete physical examination, including weight, vital signs, and clinical assessments of head, ears, eyes, nose, and throat; neck; lymph nodes; heart; chest; abdomen; extremities; neurological function; and skin;

- Clinical laboratory tests including:
    - CBC with differential and platelet count,
    - Chemistry panel (ALT, AST, and alkaline phosphatase),
    - cTnT, and
    - Urine dipstick (urinalysis if appropriate; see section 9.9); and
- Urine or serum pregnancy test (for participants who were born female).
- Mucosal secretion sampling (rectal and cervical [for participants born female] specimens for volunteers who consent to the procedure(s)) (see section 9.5)
- Gonorrhea, Chlamydia, Trichomonas, Bacterial Vaginosis, and HSV-2 testing for female volunteers who consent to mucosal secretion sampling (see section 9.5)

9.4.1 Cardiac safety monitoring

Myo/pericarditis has been observed in recipients of vaccinia vaccinations used to protect against smallpox. It has been an *uncommon* occurrence in vaccinia recipients. Myo/pericarditis has not been documented to occur with MVA. However, the safety of trial participants is the major priority for the HVTN and the Protocol Team, and thus enhanced cardiac safety monitoring has been added to this protocol to detect potential cardiac effects of study vaccine. Specifically, screening ECGs and testing for cTnT, biomarkers for cardiac tissue injury, have been added to the protocol. The eligibility criteria exclude potential participants with pre-existing cardiac risk factors and/or cardiac conditions, baseline elevations in troponin or certain ECG findings which could compromise the detection of myo/pericarditis.

Several reports in the literature suggest that cardiac enzymes are more sensitive and specific for myo/pericarditis than ECGs, with cardiac enzyme elevation seen in 60/61 (98.4%) of the military cases of myo/pericarditis compared to identifiable ECG abnormalities in 46/61 (75.4%) [58] and that cardiac troponin is more sensitive than creatine kinase myocardial band isoenzyme (CK-MB) for myo/pericarditis [59]. cTnT is assessed at screening and at 2-week postvaccination visits.

ECGs are performed at screening, and as clinically indicated. ECG interpretation will be centralized, and provided by a contract ECG laboratory staffed by a cardiologist who has ongoing experience with interpreting ECGs from several other vaccinia and MVA trials. This should provide consistency in the application of ECG screening criteria for enrollment, in the quality of the ECG data, and in assessing ECG changes as in other MVA studies [60].

9.4.1.1 Cardiac symptoms assessment

At all vaccination visits and all subsequent visits (see Appendix F), participants will be questioned specifically about symptoms and signs suggestive of myo/pericarditis or other cardiovascular complications as listed below:

- Shortness of breath,

- Chest pain/discomfort,
- Palpitations,
- Unexplained fatigue, and
- Fever, chills, myalgias/arthralgias.

If cardiopulmonary symptoms are reported, the study staff will perform a cardiopulmonary evaluation. Any report of these or any other signs and symptoms suggestive of any new cardiovascular condition will prompt an appropriate diagnostic evaluation as medically indicated.

9.4.1.2 cTnT

Testing for cTnT, biomarkers for cardiac tissue injury, is required for all participants at screening and at additional visits specified in Appendix F. cTnT tests will be performed at local laboratories. Sites should consult their local laboratories for information on specific handling requirements.

The inclusion criteria specify that cTnT must not exceed the institutional upper limit of normal.

Once a participant has been enrolled, any cTnT result above the institutional upper limit of normal should be reported by phone or email to the SDMC Clinical Affairs staff within 24 hours (contact information listed in *HVTN 086 / SAAVI 103 Study Specific Procedures, Key Resource Guide*), and repeated as soon as possible along with a CK-MB, and an additional ECG.

9.4.1.3 ECG testing

A 12-lead ECG is required at screening, and as clinically indicated. ECG equipment will be provided by the HVTN or accessed locally by the sites. ECG interpretation will be provided by a contract ECG laboratory.

9.4.1.4 Evaluation of suspected myo/pericarditis

The classic presentation of myo/pericarditis may not always be apparent with very early involvement. Since apparently benign symptoms may be suggestions of or mimic myo/pericarditis, there should be a low threshold for additional investigation of chest sensation or symptoms referable to the chest. As with evaluation for all potentially serious health problems in study participants, the protocol team recommends that the clinic physician be involved in the evaluation and clinical decision making associated with cardiac symptoms.

Any participant who develops symptoms or findings suggestive of possible myo/pericarditis (such as chest pain, dyspnea, palpitations, congestive heart failure, ECG abnormalities, or an elevated cTnT) 45 days following vaccination will be evaluated with an ECG, cTnT, and CK-MB by study staff as long as performing these tests in the research setting does not interfere with prompt medical care of the participant. Symptoms or findings that lead to a cardiac evaluation or referral for suspected myo/pericarditis

should be reported by phone or email to the SDMC Clinical Affairs staff within 24 hours (contact information listed in *HVTN 086 / SAAVI 103 Study Specific Procedures*, *Key Resource Guide*).

The participant with symptoms and cardiac enzyme findings and/or ECG findings consistent with suspected or probable myo/pericarditis related to vaccine according to the CDC case definition [61], attached as to this protocol, will be referred to a cardiologist for consultation and care. Asymptomatic participants with objective findings of myo/pericarditis such as ECG and cardiac enzyme abnormalities should also be evaluated and referred appropriately. The site will communicate a request to the cardiologist that the initial evaluation include any of the following tests that have not been done previously for evaluation of that specific cardiac event: an ECG, cTnT, CK-MB, and echocardiography. The site will request permission from the participant for access to medical records related to the evaluation. An AE of myo/pericarditis with a suspected causal relationship to vaccine would be followed by study staff until resolution and the participant will be contacted 1 year after the event to complete follow-up of the AE.

Any episode of myo/pericarditis at any grade must be reported to SDMC Clinical Affairs immediately and reported as an SAE, as described in Section 11.2.3. Study staff will follow any AE of myo/pericarditis until resolution.

9.5 Mucosal secretion sampling

Mucosal secretions samples will be collected from all study participants who have consented to, and are eligible for, these optional procedures at the timepoints indicated in Appendix F. Participants who have consented to, and are eligible for, mucosal secretion sampling will provide samples of rectal and cervical secretions (the latter for participants born female only).

Female participants who consent to provide rectal or cervical secretions will be tested for the following infections at the mucosal sampling visit: gonorrhea, Chlamydia, Bacterial Vaginosis, Trichomoniasis, and HSV-2. These testing results will be provided to participants and all participants who test positive for one or more of these infections will receive counseling as well as treatment or referral for treatment as appropriate.

Rectal secretion sampling: Participants should abstain from unprotected receptive anal sex without a condom for 24 hours prior to sample collection.

Cervical secretion sampling: Participants who were born female must report having had a Pap smear within the 3 years prior to enrollment, with the latest result reported as normal or ASCUS (atypical squamous cells of undetermined significance). A pregnancy test must be performed and must be negative prior to any cervical mucosal sampling. Cervical mucosal sampling should be deferred if a participant is menstruating, but should be performed as soon as possible, within the visit window. Participants providing cervical secretion samples should be advised as follows:

- Do not use anything with spermicide for 48 hours before the samples are collected;

- Do not insert anything into the vagina for 24 hours before the samples are collected (this includes but is not limited to abstaining from protected or unprotected vaginal sex);

- Do not douche for 48 hours before sample collection

9.6 Extended Safety Surveillance

Participants will be contacted for purposes of safety surveillance once a year for 2 years following the first 12 months of scheduled clinic visits (see Appendix B and Appendix G). At these contacts, CRS staff will collect the information listed below. Clinic visits will only be required if HIV confirmatory testing is necessary (see section 9.6.1); however, a clinic visit may be arranged for other reasons.

- Confirmation of vital status; if deceased, attempt to learn cause and date of death;
- If participant is alive, record the participant's responses to questions regarding any occurrence of the following events since the last HVTN study contact:
    - Life-threatening adverse experiences;
    - Persistent or significant disability/incapacity;
    - Hospitalizations and reasons;
    - Other important medical events that may jeopardize the participant or may require intervention to prevent 1 of the other outcomes listed above;
    - New chronic conditions requiring more than 30 days of medical intervention or medication;
    - New diagnosis of HIV infection; and
    - Pregnancies and outcomes, including congenital anomalies/birth defects.

All such events will be recorded, and AEs will be assessed for relationship to study product.

9.6.1 Interim contacts

CRSs may report safety information obtained at a contact other than the annual contact. These contacts are reported as interim visits.

9.7 HIV counseling and testing

HIV counseling will be performed in compliance with the CDC's guidelines or other local guidelines for HIV counseling and referral. HIV testing will be performed in accordance with the current HVTN HIV testing algorithm following enrollment.

Participants will be counseled at all scheduled visits during the trial on the avoidance of HIV infection and on the potential negative social impacts of testing Ab positive due to the vaccine. They will also be counseled on the risks of HIV Ab testing outside of the HVTN CRSs and will be discouraged from doing so during study participation and/or during any period of vaccine-induced positive serology.

CRS staff should also inform participants of the need to maintain study blinding by getting HIV testing only at the study CRS. CRS staff should provide participants with CRS contact information and should encourage participants to ask medical providers to contact the CRS. The CRS can verify that the participant is in an HIV vaccine clinical trial and should only be tested at the study CRS.

Potential participants identified as being HIV infected during screening are not enrolled. All participants who become HIV infected during the study will be terminated from this study. Potential and enrolled participants identified as HIV infected will be referred for medical treatment, counseling, and management of the HIV infection. These individuals may also be referred to appropriate ongoing clinical trials or observational studies.

9.7.1 Distinguishing intercurrent HIV infection from vaccine-induced positive serology

The study product may elicit an Ab response to HIV proteins. Therefore, vaccine-induced positive serology may occur in this study. Several precautionary measures will be taken to distinguish intercurrent HIV infection from vaccine-induced positive serology. These precautionary measures include:

- Participants will have physical examinations at visits specified in Appendix F. Signs or symptoms of an acute HIV infection syndrome, an intercurrent illness consistent with HIV-1 infection, or probable HIV exposure would prompt a diagnostic workup per the HVTN algorithm for Recent Exposure/Acute Infection Testing to determine HIV infection.

- HIV testing will be performed at multiple timepoints throughout the study (see Appendix E). The Laboratory Program (or approved diagnostic laboratory) will follow the HVTN HIV testing algorithm (as described in the *HVTN Laboratory Manual of Operations*), which is able to distinguish vaccine-induced Ab responses from actual HIV infections.

- During the period between last study visit and unblinding, all participants can receive HIV-1 diagnostic testing from the site.

- All participants who received vaccine product and who have vaccine-induced positive or indeterminate HIV-1 serology (as measured by the standard anti-HIV Ab screening tests) at or after the study is unblinded will be offered poststudy HIV-1 diagnostic testing (per the HVTN poststudy HIV-1 testing algorithm) periodically and free of charge as medically/socially indicated (approximately every 6 months).

9.8 Contraception status

Contraception status is assessed and documented at every scheduled clinic visit for a participant who was born female and who is sexually active in a way that could cause that participant to become pregnant. Prior to enrollment and throughout the study, staff will ask participants to verbally confirm their use of adequate contraceptive methods. A participant who was born female and is sexually active in a way that could cause that participant to become pregnant should be reminded at all scheduled clinic visits of the importance of using contraception and should be referred to specific counseling,

information, and advice as needed. (Specific contraception requirements are listed in section 7.1.) This reminder should be documented in the participant's study record.

Self-reported infertility—including having reached menopause (no menses for 1 year) or having undergone hysterectomy, bilateral oophorectomy, or tubal ligation—must be documented in the participant's study record.

9.9 Urinalysis

Dipstick testing may be performed in the clinic or the lab, as long as the required elements (glucose, protein, and hemoglobin) are tested. The examination is performed on urine obtained by clean catch.

If the screening dipstick is transiently abnormal due to menses or infection, document this issue in the participant's source documentation. For infection, provide appropriate treatment and/or referral. Following resolution, repeat the dipstick and, if within the eligibility limits specified in the protocol, the participant may be enrolled.

Follow-up urinalysis should be deferred if a participant is menstruating, but should be performed as soon as possible. If a follow-up dipstick is abnormal due to a participant's menstrual period, document in the comment section of the case report form (CRF) and repeat the dipstick once the participant is no longer menstruating. A micro-urinalysis is not required.

9.10 Assessments of reactogenicity

For all participants, baseline assessments are performed before and reactogenicity assessments are performed after each vaccination. All reactogenicity symptoms are followed until resolution and graded according to the *Division of AIDS Table for Grading the Severity of Adult and Pediatric Adverse Events* (DAIDS AE Grading Table), Version 1.0, December 2004 (Clarification, August 2009).

The reactogenicity assessment period is 3 full days following each vaccination per the assessment schedule shown in Table 9-1 below. Participants are instructed to record symptoms using a postvaccination symptom log and to contact the site daily during the assessment period. Clinic staff will follow new or unresolved reactogenicity symptoms present at day 3 to resolution. Participants are instructed to contact the clinic for events that arise during the period between vaccination and the next scheduled visit. In general, a participant who self-reports any postvaccination reaction greater than mild is seen by a clinician within 48 hours after onset, unless the reaction is improving and/or has completely resolved.

Reactogenicity events are reported using CRFs that correspond to the time of assessment in Table 9-1. Reactogenicity assessments include assessments of systemic and local symptoms, vaccine-related lesions, and lymph nodes. Events not listed on a CRF, or with an onset after the reactogenicity assessment period (day of vaccination and 3 full days after), or those meeting SAE criteria, are recorded on an adverse experience log form.

Table 9-1 Schedule of reactogenicity assessments

Day	Time	Performed by
0[a]	Baseline: before vaccination	HVTN CRS staff
	Early: 25-60 minutes after vaccination	HVTN CRS staff
	Between early assessment and 11:59 pm day 0	HVTN CRS staff or participant
1	Between 12:00 AM and 11:59 PM day 1	HVTN CRS staff or participant
2	Between 12:00 AM and 11:59 PM day 2	HVTN CRS staff or participant
3[b]	Between 12:00 AM and 11:59 PM day 3	HVTN CRS staff or participant

[a] Day of vaccination

[b] New or unresolved reactogenicity symptoms present on day 3 are followed until resolution

9.10.1 Assessment of systemic and local symptoms

Systemic symptoms include increased body temperature, malaise and/or fatigue, myalgia, headache, chills, arthralgia, nausea, and vomiting. Local symptoms include pain and/or tenderness proximal to the injection site. The daily maximum severity reached for each symptom during the assessment period is reported.

Body temperature is measured by oral or infrared thermometry and reported in degrees Celsius. If temperature is measured in Fahrenheit, the conversion to Celsius should be documented in the participant's chart note. A measurement is taken once daily during the assessment period and should be repeated if participant is feeling feverish.

9.10.2 Assessment of injection site

Typical injection site reactions are erythema/induration/swelling/edema. The maximum horizontal and maximum vertical measurements for all injection site reactions are recorded.

All injection site reactions are monitored until resolution. Areas greater than 25 cm^2 are followed daily; otherwise, the frequency of follow-up is based on clinician judgment.

9.10.3 Assessment of lymph nodes

This assessment is required only when reactogenicity assessments are performed by HVTN CRS staff, not by the participant.

Only the proximally draining lymph nodes are assessed (eg, axillary nodes on the same side of the body for injections given in the deltoid). Lymph nodes are first evaluated for enlargement and tenderness. If they are found to be enlarged, measurements are taken to determine the size (widest diameter) of the enlarged node(s).

9.11 Visit windows and missed visits

Visit windows are defined in *HVTN 086 / SAAVI 103 Study Specific Procedures*. For a visit not performed within the window period, a Missed Visit form is completed. If the missed visit is one that required safety assessments or local safety labs, HVTN CRS staff should attempt to bring the participant in for an interim visit as soon as possible.

Procedures performed at an interim visit are limited to toxicity/safety assessments (including local safety labs) and HIV testing. With the exception of HIV testing, these procedures are performed only if they were required at the missed visit or if clinically indicated. HIV testing may be performed as deemed appropriate by the study staff. Blood samples for immunogenicity assays are not typically collected at interim visits.

If a missed visit required vaccination, please refer to section 7.3.2 and section 7.3.3 for resolution.

9.12 Early termination visit

In the event of early participant termination, site staff should consider if the following assessments are appropriate: a final physical examination, clinical laboratory tests (including urine dipstick, CBC with differential, platelet count, and chemistry panel), pregnancy testing, social impact assessment, and HIV test.

9.13 Pregnancy

If a participant becomes pregnant during the course of the study, no more injections of study product will be given but remaining visits and study procedures should be completed unless medically contraindicated. If the participant terminates from the study prior to the pregnancy outcome, the site should make every effort to keep in touch with the participant in order to ascertain the pregnancy outcome.

10 Laboratory

10.1 HVTN CRS laboratory procedures

The *HVTN Site Lab Reference Manual* provides further guidelines for operational issues concerning the clinical and processing laboratories. The manual includes guidelines for general specimen collection, special considerations for phlebotomy, specimen labeling, whole blood processing, HIV screening/diagnostic testing, and general screening and safety testing.

Tube types for blood collection are specified in Appendix E. For tests performed locally, the local lab may assign appropriate tube types.

In specific situations, the blood collection tubes will be redirected to another laboratory or will require study-specific processing techniques. In these cases, laboratory special instructions will be posted on the protocol-specific section of the HVTN website.

10.2 Total blood volume

Required blood volumes per visit are shown in Appendix E. Not shown is any additional blood volume that would be required if a safety lab needs to be repeated, or if a serum pregnancy test needs to be performed. The additional blood volume would likely be minimal. The total blood volume drawn for each participant will not exceed 500 mL in any 56-day (8-week) period.

10.3 Primary immunogenicity timepoints

The primary immunogenicity timepoints in this study are at visit 9 and visit 12. For groups 1, 3 and 4 this is 2 weeks after the 3rd and 4th vaccinations; for group 2 this is 2 and 14 weeks after the 2nd vaccination. Endpoint assays for humoral and cellular responses are performed on all participants at the primary immunogenicity timepoints and may be performed on baseline specimens. Depending on the number of responders observed, assays for humoral and cellular responses may be performed on all participants at other timepoints. The schedule is shown in Appendix E.

10.4 Endpoint assays: humoral

10.4.1 HIV-nAb assay

HIV-1–specific nAb assays will be performed on serum samples from all study participants taken at the primary immunogenicity timepoints. Specimens from baseline and other timepoints may also be analyzed contingent on the results of the primary immunogenicity timepoints. The tier 1 assays will test neutralization of HIV-1 strains represented in the vaccine constructs (TV1, Du151) and the highly neutralization-sensitive tier 1 subtype C isolate (MW965.26). The tier 2 assays may be conducted to test

neutralization of a panel of at least 12 primary subtype C isolates. Neutralization of additional isolates may be assessed.

10.4.2 HIV-1 multiplex Ab assay

As a secondary endpoint, total binding IgG (IgG1, IgG2, IgG3, IgG4) and IgA antibodies to HIV-1 ConS gp140, Du151 Env, and/or TV1 gp140 will be assessed on plasma samples from all study participants taken at the primary immunogenicity timepoints and the baseline. Specimens from other timepoints as well as other HIV antigens may also be assayed based on the results of the initial assay.

10.4.3 Ab avidity

As an exploratory analysis, antigen specific IgG avidity may be determined by using a BIAcore instrument. Binding responses (steady-state), dissociation constant (Kd) and dissociation rate constant (Kd, off-rate) as measures of Ab affinity will be determined for Ab responses in each participant's plasma sample. Higher avidity Ab responses will be defined as those with relatively lower Kd (~nM range) and slower off-rates ($\sim 10^{-3}\ s^{-1}$) for a specific antigen. To monitor changes in Ab avidity, SPR-based assays provide the capability to detect lower avidity Ab with faster off-rates ($Kd < 0.1\ s^{-1}$).

10.4.4 Ab epitope mapping by peptide array

As an exploratory analysis, linear epitopes may be mapped using peptide array technology. The array will consist of 15mer peptides overlapping by 12 amino acids covering multiple full length gp140 consensus sequences consisting of ConA, ConB, ConC, ConD, ConAG, ConAE, and ConM.

10.4.5 Antibody dependent cellular cytotoxicity (ADCC) assay

As an exploratory analysis, ADCC may be investigated. The ADCC assay will use HIV-1 infected cells as targets (eg, CEM.NK.CCR5 cells) and either cryopreserved PBMC or a suitable cell line as effectors cells. Percent specific ADCC activity will be based on either degranulation or lysis and will be quantified by either flow cytometry or luminescence, respectively.

10.5 Endpoint assays: cellular

10.5.1 Flow cytometry

Flow cytometry will be used to examine vaccine-specific CD4+ and CD8+ T-cell responses following stimulation of PBMCs with synthetic HIV peptides that span the proteins encoded by the vaccine construct. ICS parameters will include cytokines such as IFN-γ, IL-2, and TNF-α, and may include other cytokines to identify T cells of specific functionality (such as Th2 and Th17). Markers of cytotoxic potential (Granzyme B, perforin and CD57) may also be included. Data will be reported as percentages of CD4+ or CD8+ T cells responding to a specific peptide pool. Additional cell surface markers, cytokines, or functional markers may also be analyzed.

10.5.2 B cell assays

As an exploratory analysis, B cell phenotyping will be conducted on PBMCs using flow cytometry to examine memory B cell and plasmablast phenotypes. Markers will include CD19, CD20, CD21, CD27, CD38, IgM, and IgD. Percentages of B cells expressing various combinations of these markers will be reported.

B cell ELISpot assays may also be conducted to analyze B cells secreting HIV-specific antibodies. For this assay, ELISpot plates are coated with capture anti-Ig antibodies (either total Ig or specific subtypes), incubated with PBMC and subsequently incubated with the biotinylated HIV antigen of interest. Visualization of spots occurs analog to the detection of IFN-γ-secreting T cells. Data will be reported as the number of spot-forming cells (SFC) per 10^6 PBMCs recognizing a specific antigen.

10.5.3 Natural Killer (NK) cell function

As an exploratory analysis, NK cell phenotyping by flow cytometry may be conducted. Markers will include NK cell receptors of the KIR, NCR and FcγR families. In addition, a flow-based assay assessing NK cell function will be utilized. This assay determines the NK cells' ability to respond to stimuli delivered through the different classes of NK receptors (eg, KIR, NCR and FcγR) by assessing NK cell degranulation (CD107 upregulation) as well as the secretion of cytokines and chemokines (especially MIP-1β).

10.6 Genotyping

Molecular human leukocyte antigen (HLA) typing may be performed on enrolled participants using cryopreserved PBMC, initially on specimens from participants who demonstrate vaccine-induced T-cell responses at postvaccination timepoints. Other participants (including control recipients) may be HLA-typed to support future studies of immunological interest at the discretion of the HVTN Laboratory Program. Other markers, such as genes associated with immune responses or susceptibility to HIV-1 infection may also be assessed.

10.7 Exploratory studies

These samples will be used for other testing and research related to furthering the understanding of HIV or vaccines. In addition, cryopreserved samples may be used to perform additional assays to support standardization and validation of existing or newly developed methods.

10.7.1 Antibody responses in the mucosa

As an exploratory endpoint, total binding IgG (IgG1, IgG2, IgG3, IgG4) and IgA antibodies to HIV-1 may be assessed on cervical and rectal secretions using the HIV-1 multiplex antibody assay. If binding antibody activity is detected, IgG and/or IgA antibodies may be purified and assessed for HIV-1 specific neutralizing activity. Additional exploratory assays for mucosal antibodies may be performed on these samples.

10.8 Other use of stored specimens

The HVTN aims not only to test vaccine candidates but also to continue to explore the correlates of immunity to HIV. In order to do so, the HVTN intends to store blood samples from participants. These samples will be used for other testing and research related to furthering the understanding of HIV pathogenesis or vaccines to the extent authorized in each study site's informed consent form, or as otherwise authorized under applicable law. Other testing on specimens will only occur, at a minimum, after review and approval by the HVTN and the IRB of the researcher requesting the specimens.

Samples will be stored at the central specimen repository in South Africa. The approval of the national PI will be obtained prior to samples being moved out of the country. The protocol sample informed consent form is written so that the participant either explicitly allows or does not allow sample storage for other research when he or she signs the form. Participants who initially agree to other use of their samples may rescind their approval once they enter the study; such participants will still remain in this study. If a participant decides against allowing other research using his or her samples, or at any time rescinds prior approval for such other use, the study site investigator or designee must notify HVTN Regulatory Affairs in writing. In either case, after study analyses are complete, the HVTN Laboratory Program will request that the repository destroy all specimens with the participant identification numbers (PTIDs) of all participants who do not agree to other use of their samples. HVTN Core will report the destruction of relevant specimens to the participants' site PIs.

Study sites must notify HVTN Regulatory Affairs if institutional or local governmental requirements pose a conflict with or impose restrictions on the use of stored specimens.

10.9 Biohazard containment

As the transmission of HIV and other blood-borne pathogens can occur through contact with contaminated needles, blood, and blood products, appropriate precautions will be employed by all personnel in the drawing of blood and shipping and handling of all specimens for this study, as currently recommended by the CDC and the NIH or other locally appropriate agencies.

All dangerous goods materials, including Biological Substances, Category A or Category B, must be transported according to instructions detailed in the International Air Transport Association Dangerous Goods Regulations.

11 Safety monitoring and safety review

11.1 Safety monitoring and oversight

11.1.1 HVTN 086 / SAAVI 103 PSRT

The PSRT is composed of the following members:

- DAIDS medical officer representative,
- Protocol chair and co-chair,
- Protocol Team leader,
- Core medical monitor, and
- SDMC Clinical Affairs safety associate.

The clinician members of PSRT are responsible for decisions related to participant safety.

The Protocol Team clinic coordinator, project manager, vaccine developer representatives, clinical trial manager, and others may also be included in PSRT meetings.

11.1.2 HVTN SMB

The SMB is a multidisciplinary group consisting of biostatisticians, clinicians, and experts in HIV vaccine research, including individuals from South Africa, that, collectively, has experience in the conduct and monitoring of vaccine trials. Members of the SMB are not directly affiliated with the protocols under review.

The SMB reviews safety data, unblinded as to treatment arm, approximately every 4 months. The reviews consist of evaluation of cumulative reactogenicity events, AEs, laboratory safety data, and reports of individual safety events that require expedited reporting. To increase the sensitivity for detecting potential safety problems, the SMB will review safety data aggregated across multiple protocols that use the same or similar vaccine candidates. The SMB conducts additional special reviews at the request of the PSRT.

Study sites will receive SMB summary minutes and are responsible for forwarding them to their local IRB/IECs.

11.1.3 SDMC roles and responsibilities in safety monitoring

The roles and responsibilities of the SDMC in relation to safety monitoring include:

- Maintaining a central database management system for HVTN clinical data;

- Providing reports of clinical data to appropriate groups such as the HVTN 086 / SAAVI 103 PSRT and HVTN SMB (see section 11.1.2);
- Daily monitoring of clinical data for events that meet the safety pause and PSRT AE review criteria (see section 11.4);
- Notifying HVTN CRSs and other groups when safety pauses or planned holds are instituted and lifted (see section 11.4);
- Querying HVTN CRSs for additional information regarding reported clinical data; and
- Providing support to the HVTN 086 / SAAVI 103 PSRT.

11.2 Safety reporting

11.2.1 Submission of safety forms to SDMC

Sites must submit all safety forms (eg, reactogenicity, adverse experience, urinalysis, local lab results, concomitant medications) before the end of the next business day after receiving the information. The forms should not be held in anticipation of additional information at a later date. If additional information is received at a later date, the forms should be updated and refaxed before the end of the next business day after receiving the new information.

11.2.2 AE reporting

An AE is any untoward medical occurrence in a clinical investigation participant administered a study product/procedure(s) and which does not necessarily have a causal relationship with this treatment. An AE can therefore be any unfavorable and unintended sign (including an abnormal laboratory finding), symptom, or disease temporally associated with the use of an investigational study product/procedure(s), whether or not related to the investigational study product/procedure(s). All AEs are graded according to the *Division of AIDS Table for Grading the Severity of Adult and Pediatric Adverse Events* (DAIDS AE Grading Table), Version 1.0, December 2004 (Clarification, August 2009), available on the RSC website at http://rsc.tech-res.com/safetyandpharmacovigilance/, except:

- unintentional weight loss of less than 10% loss in body weight from baseline is not required to be reported as an AE;
- PR interval ≤ 0.219 sec will not be reported as an AE;
- asymptomatic increase in QTc interval < 0.06 sec above baseline, if the QTc ≤ 0.45 sec, will not be reported as an AE; and
- The definition of Grade 1 mild prolonged PR interval (Adult > 16 years) that will be used is 0.22 – 0.25 sec.

The criteria for prolonged QTc interval that will be used are:

- Grade 1, mild: asymptomatic, QTc interval 0.45–0.47 sec;
- Grade 2, moderate: asymptomatic, QTc interval 0.48–0.49 sec;
- Grade 3 severe: asymptomatic, QTc interval ≥ 0.50 sec OR increase in interval ≥ 0.06 sec above baseline; and
- Grade 4, potentially life-threatening: life-threatening consequences (eg, Torsade de pointes or other associated serious ventricular dysrhythmia).

All AEs are reported to the SDMC on the appropriate CRF. Clinic staff should evaluate every AE to determine (1) if the AE meets the requirements for expedited reporting to MCC (section 11.2.4) and (2) if the AE meets the criteria for a safety pause/prompt AE review (section 11.4).

Sites are expected to notify SDMC Clinical Affairs staff of any serious safety concern requiring their attention (see Table 11-1). Telephone numbers and email addresses are listed in the Key Resource Guide of the *HVTN 086 / SAAVI 103 Study Specific Procedures*. Concerns requiring immediate attention should be communicated by calling the SDMC Clinical Affairs safety phone.

In the case of email notification, SDMC Clinical Affairs staff will reply during working hours (US Pacific Time) to confirm that the email has been received and reviewed. If email service is not available, the HVTN CRS should notify SDMC Clinical Affairs of the event by telephone, then submit CRFs.

In addition, site investigators are required to submit AE information in accordance with local regulatory agencies' or other local authorities' requirements.

11.2.3 SAEs

An AE is considered to be a "serious adverse event" by Medicines Control Council guidelines as described in *Reporting Adverse Drug Reactions in South Africa*, Version 1, May 2003 if it:

- results in death,
- is life-threatening,
- requires patient hospitalisation or prolongation of existing hospitalisation,
- results in persistent or significant disability/incapacity, or
- is a congenital anomaly/birth defect.

The term "life-threatening" in the definition of "serious" refers to an event in which the patient was at risk of death at the time of the event; it does not refer to an event, which hypothetically might have caused death, if it were more severe.

Medical and scientific judgement should be exercised when deciding if other situations are serious. Such instances could include medical events that may not be immediately life-threatening or result in death or hospitalisation, but which may jeopardise the patient or may require intervention to prevent one of the outcomes listed in the definition above. Examples include blood dyscrasias or convulsions not resulting in hospitalisation, or development of drug dependency or drug abuse.

11.2.4 SAEs requiring expedited reporting

The sponsor or designee(s) prepares and files expedited reports to appropriate regulatory authorities and ethics committees within the timelines required by the South African MCC guidelines, which are detailed in *Reporting Adverse Drug Reactions in South Africa*, Version 1, May 2003.

Any SAE that is considered unexpected and for which the contribution of the study products cannot be ruled out will qualify for expedited reporting to the South African Medicines Control Council (MCC). The expedited reporting period for this study comprises the entire study period for each individual participant (from study enrollment until study completion or discontinuation from the study).

The study products that must be considered in determining relationships of AEs requiring expedited reporting to MCC are:

- SAAVI DNA-C2/placebo
- SAAVI MVA-C/placebo
- TV1gp140ΔV2 + MF59 / placebo

Any SAE which there is a reasonable possibility that the study agent caused or contributed to will qualify for expedited reporting to Clinical Affairs at SCHARP within 24 hours in addition to completion of the standard AE form. Clinical Affairs staff notifies the HVTN 086 / SAAVI 103 PSRT as soon as possible during working hours (US Pacific Time) – or, if the information was received during off hours, by the morning of the next working day – that a prompt PSRT AE review is needed. The PSRT review will take place within 72 hours.

11.3 Safety reviews

11.3.1 Initial safety evaluation

Enrollment across all participating HVTN CRSs will be restricted to a maximum of 1 participant per group per day until 5 participants have been enrolled in each group. The HVTN 086 / SAAVI 103 PSRT will review the safety data (AEs and laboratory results) and local and systemic reactogenicity data reported for the first 72 hours postvaccination on each of these 5 participants per group and will determine whether it is safe to proceed with full enrollment in that group.

11.4 Safety pause and prompt PSRT AE review

The AEs that will lead to a safety pause or prompt PSRT AE review are summarized in Table 11-1. Vaccinations may be suspended for safety concerns other than those described in the table, or before pause rules are met, if, in the judgment of the PSRT, participant safety may be threatened. Criteria for an individual participant's departure from the schedule of vaccinations are listed in section 7.3.

Table 11-1 AE notification and safety pause/AE review rules

Event and relationship to study products / procedure	Severity	HVTN CRS action	SDMC action
SAE, not related*	Grade 5	Phone immediately, email and fax forms immediately	Immediate PSRT notification
SAE, related*	Grade 5 or Grade 4	Phone immediately, email and fax forms immediately	Immediate pause
SAE, related	Grade 3	Email and fax forms immediately	Prompt PSRT AE review to consider pause
AE**, related	Grade 3 or 4	Email and fax forms immediately	Prompt PSRT AE review to consider pause
SAE, related	Grade 1 or 2	Email and fax forms immediately	Prompt PSRT AE review to consider pause

For AE descriptions and grading, see *The Division of AIDS Table for Grading the Severity of Adult and Pediatric Adverse Events* (DAIDS AE Grading Table).

Phone numbers and email addresses are listed in *HVTN 086 / SAAVI 103 Study Specific Procedures*, Key Resource Guide.

* Definition of relatedness to be used for AE notifications, pauses, and reviews, from *The Manual for Expedited Reporting of Adverse Events to DAIDS*, Version 2.0, January 2010: There is a reasonable possibility that the study agent caused or contributed to the event.

** Does not include subjective reactogenicity symptoms (injection site pain, tenderness, fatigue/malaise, myalgia, arthralgia, chills, headache, nausea).

For all safety pauses, the SDMC Clinical Affairs staff notifies the PSRT, DAIDS Pharmaceutical Affairs Branch (PAB), and participating HVTN CRSs that all enrollment and vaccination with the product related to the event that triggered the pause will be held until further notice. When an immediate safety pause is triggered, the SDMC Clinical Affairs staff also notifies the HVTN SMB; the trial sponsor or designee(s) notifies the South African MCC for all safety pauses.

If a prompt PSRT AE review is triggered, the SDMC Clinical Affairs staff notifies the HVTN 086 / SAAVI 103 PSRT as soon as possible during working hours (US Pacific

Time)—or, if the information was received during off hours, by the morning of the next work day—that a prompt PSRT AE review is needed. If a prompt PSRT AE review cannot be completed within 72 hours of SDMC notification (excluding weekends), an automatic safety pause occurs.

The PSRT reviews safety data and decides whether the pause can be lifted or permanent discontinuation of vaccination is appropriate, consulting the SMB if necessary. Sponsor or designee(s) will notify the South African MCC for all safety pauses. SDMC Clinical Affairs staff notifies the participating HVTN CRSs and DAIDS PAB of the decision regarding resumption or discontinuation of study vaccinations. Sponsor or designee(s) will notify the South African MCC.

Each HVTN CRS is responsible for submitting to its IRB/IEC and any local regulatory authority protocol-related safety information (such as IND safety reports, notification of vaccine holds due to the pause rules, etc), as required.

Each South African HVTN CRS must comply with the MCC guide for *Reporting Adverse Drug Reactions in South Africa* located on the MCC website (http://www.mccza.com).

AEs that do not trigger a safety pause or a prompt PSRT AE review are routinely reviewed by the PSRT (section 11.5.2).

11.5 Review of cumulative safety data

Routine safety review occurs at the start of enrollment and then throughout the study.

Reviews proceed from a standardized set of protocol-specific safety data reports. These reports are produced by the SDMC and include queries to the HVTN CRSs. Events are tracked by internal reports until resolution.

11.5.1 Daily review

Blinded daily safety reviews are routinely conducted by the SDMC Clinical Affairs staff for events requiring expedited reporting to MCC (SAEs), and events that meet safety pause criteria or prompt PSRT AE review criteria.

11.5.2 Weekly review

During the injection phase of the trial, the SDMC Clinical Affairs staff and the PSRT review clinical safety reports on a weekly basis and conduct calls to review the data as appropriate. After the injections and the final 2-week safety visits are completed, less frequent reporting and safety reviews may be conducted at the discretion of the PSRT. The SDMC Clinical Affairs staff reviews reports of clinical and laboratory AEs. Events identified during the review that are considered questionable, inconsistent, or unexplained are referred to the HVTN CRS clinic coordinator for verification.

11.6 Study termination

This study may be terminated early by the determination of the HVTN 086 / SAAVI 103 PSRT, HVTN SMB, South African MCC, NIH, Office for Human Research Protection (OHRP), or vaccine co-developers. In addition, the conduct of this study at an individual HVTN CRS may be terminated by the determination of the local IRB or IEC, or of the appropriate local or national regulatory authority.

12 Protocol conduct

The protocol will be conducted in compliance with the principles of GCP and according to standard DAIDS and HVTN and MCC policies and procedures, including procedures for the following:

- Protocol registration, activation, and implementation;
- Informed consent, screening, and enrollment;
- Clinical and safety assessments;
- Safety monitoring and reporting;
- Data collection and documentation;
- Study follow-up and close-out;
- Unblinding of staff and participants;
- Quality control;
- Protocol monitoring and compliance;
- Advocacy and assistance through local and governmental activities to participants regarding social impacts associated with the vaccine trial;
- Risk reduction counseling; and
- Specimen collection, processing, and analysis.

Any policies or procedures that vary from DAIDS and HVTN and MCC standards or require additional instructions will be described in the HVTN 086 / SAAVI 103 *Study Specific Procedures* (eg, instructions for randomization specific to this study).

12.1 Overview of data collection methods

Clinical research data will be collected in a secure electronic data management system by the assigned SDMC. Data will be extracted and provided to the protocol statistician for statistical analysis.

12.1.1 Source documents and data entry at sites

Standard GCP will be followed to ensure accurate, reliable, and consistent data collection. Each participating site will maintain appropriate medical and research records for this trial, in compliance with ICH, GCP, regulatory, network, and institutional requirements for the protection of confidentiality of participants.

HVTN sites will follow the *DAIDS Standard Operating Procedure on Source Documentation*, Version 2 or any later version in managing source documentation for the trial. Source document information may include but is not limited to:

- Signed informed consent documents;
- Dates of visits, including dates of study injections;
- Documentation of the study eligibility evaluation;
- Reported laboratory results;
- AE evaluations;
- Participant-reported concomitant medications; and
- Participant-reported local and systemic reactogenicity.

CRFs and laboratory reports will be reviewed by the site clinical team responsible for ensuring that they are accurate and complete. Many HVTN CRFs are designed to be used as source documents. HVTN CRSs complete a source documentation table to indicate which CRFs the site will use as source documents for the trial.

12.1.2 Participant confidentiality

Documentation, data, and all other information generated for a participant will be held in strict confidence. No identifying participant information concerning the study or the data will be released to any unauthorized third party without prior written approval of the participant except as necessary for monitoring by the IRB/IEC, the MCC, the FDA (if applicable), the study sponsor, the OHRP, and the pharmaceutical supporter(s) (if applicable). Information about a study participant also may be released when required by law. Participants must be made aware in the informed consent document of the occasions when information may be released without their consent. In addition, if information is released, either by accident or deliberately without a participant's consent, the site must attempt to notify the participant of the release, complete a Protocol Event Form (see section 12.2.2), and notify their IRB/IEC.

The study database assembled by the SDMC will identify study participants only by a study identification number and will not contain identifying information such as name, address, national identification number, medical record number, or personal contact information.

12.1.3 Lab data transfer

Data generated at central and regional laboratories will be transferred directly from the laboratory to the SDMC by secure means and with procedures that ensure the integrity of the data.

12.1.4 Storage of source documents and completed CRFs

All study data must be verifiable to the source documentation. A file containing all the source documents will be maintained for each study participant at the study site. Source documentation will be available for review to ensure that the collected data are consistent with the CRFs.

CRFs, source documents, and other supporting documents will be kept in a secure location.

12.2 HVTN CRS monitoring

To ensure protection of study participants, compliance with the protocol, and accuracy and completeness of records, site monitors under contract to NIAID may visit participating CRSs to review the individual subject records, including consent forms, CRFs, supporting data, laboratory specimen records, and medical records (physicians' progress notes, nurses' notes, and individuals' hospital charts). The monitors will inspect sites' regulatory files to ensure that regulatory requirements are being followed and may also inspect sites' pharmacies to review product management and storage.

12.2.1 Access to source documents

Because this study is funded by NIAID, each site must permit authorized representatives of NIAID and regulatory agencies to examine (and, when required by applicable law, to copy) clinical records for the purposes of quality assurance reviews, audits, and evaluation of the study's safety and progress.

Additionally, each site must permit representatives of the HVTN, SDMC, and related contractors to examine clinical records for the purposes of quality assurance reviews, audits, and evaluation of the study's safety and progress.

12.2.2 Protocol events

A protocol event is defined as an individual incident or omission in study conduct that results in significant added risk to the participant, or nonadherence to significant protocol requirements, or nonadherence to the *International Conference on Harmonisation E6: Guideline for Good Clinical Practice*.

The nonadherence may be either on the part of a participant, the investigator, or the study site staff.

It is the responsibility of the site to identify and report protocol events according to the guidelines of the regulatory authority under which the study is conducted and the local IRB/IEC per their guidelines. The site must also report protocol events to the HVTN using the Protocol Event Form. The site PI and study staff are responsible for knowing and adhering to their IRB requirements.

In response to noted protocol events, site personnel are to implement corrective actions promptly, as necessary.

12.3 Social impacts

Participants in this study risk experiencing discrimination or other personal problems as a result of being in the study or developing a vaccine-induced positive HIV Ab response. The HVTN CRS is obliged to provide advocacy for and assistance to participants regarding negative social impacts associated with the vaccine trial. If HVTN CRS staff have questions regarding how to assist a participant dealing with a social impact, a designated NIAID representative can be contacted.

Social harms are tabulated by the SDMC and subjected to descriptive analysis with a view toward reducing their incidence and enhancing the ability of study staff to mitigate them when possible.

Summary tables of social impact events will be generated weekly and made available for review by the protocol chairs, Protocol Team leader, and the designated NIAID representative.

12.4 Study participant reimbursement

Reimbursement of study participants for attendance at study visits is at the discretion of each study site. Reimbursement should be comparable to the reimbursement offered for similar research in the local community, if possible. The study site is encouraged to confer with its local CAB in deciding appropriate reimbursement.

The study consent submitted to the site IRB/IEC will state the plan for reimbursement (if any). The HVTN relies upon local IRBs/IECs to determine whether the proposed plan for reimbursement meets ethical requirements in the local context. The exact amounts may be modified during the course of the study in consideration of changes in costs such as bus fares, exchange rates, child care, or other factors that affect the ability of a participant to comply with study visit requirements. Reviewing IRBs/IECs must be made aware of the changes in reimbursement before they occur. Study participants will not be charged for study injections, research clinic visits, research-related examinations, or research-related laboratory tests.

The HVTN does not allow reimbursement that induces a study participant to remain in the study against his or her will. A lump sum reimbursement at trial completion is unacceptable.

12.5 Compliance with NIH guidelines for research involving products containing recombinant DNA

Because this study is evaluating products containing recombinant DNA, per NIH *Guidelines for Research Involving Recombinant DNA Molecules* the study must be submitted to site Institutional Biosafety Committees (IBC) and must be approved before participants are enrolled at each respective institution. Investigators at each site are responsible for obtaining IBC approval and periodic review of the research per NIH guidelines *section IV-B07-b-(6)* and *section IV-B-2-b.* IBC review and approval must be documented by the investigator and submitted as part of protocol registration for this trial.

12.6 Specific regulatory considerations for South Africa

South Africa has laws regarding the use, manufacture, importation, and experimentation of products which are genetically modified. These are contained in the Genetically Modified Organism (GMO) Act 15 of 1997, administered by the South African National Department of Agriculture, Pretoria. The Registrar of GMO shall be consulted on all formal developments relating to this protocol and clinical trial, and as required, a formal application will be made to the Registrar of GMO to review the HVTN 086 / SAAVI 103 clinical trial, to obtain approval for the proposed clinical trial and for the importation of the study products.

13 Version history

The Protocol Team may modify the original version of the protocol. Modifications are made to HVTN protocols via clarification memos, letters of amendment, or full protocol amendments.

The table below describes the version history of, and modifications to, Protocol HVTN 086 / SAAVI 103.

Protocol history and modifications

Date	Protocol version	Protocol modification	Comment
28-SEP-11	Version 2	Full Protocol Amendment	
16-MAY-11	Version 1	Clarification memo #1 to Version 1	
11-FEB-11	Version 1	LOA #1 to Version 1	
28-MAY-10	Version 1	Original protocol	

14 Document references (other than literature citations)

Other documents referred to in this protocol, and containing information relevant to the conduct of this study, include:

- Assessment of Understanding. Accessible through the HVTN protocol-specific website.

- Current ABPI Guidelines. *Guidelines for Phase 1 Clinical Trials 2007 Edition* Available at http://www.abpi.org.uk/publication/pdfs/phase1_guidelines.pdf

- Current CDC Guidelines. *Revised Recommendations for HIV Testing of Adults, Adolescents, and Pregnant Women in Health-Care Settings.* Available at http://www.cdc.gov/mmwr/PDF/rr/rr5514.pdf.

- *Division of AIDS (DAIDS) Clinical Research Policies and Standard Procedures Documents*. Available at http://www3.niaid.nih.gov/research/resources/DAIDSClinRsrch/

- *Division of AIDS Table for Grading the Severity of Adult and Pediatric Adverse Events* (DAIDS AE Grading Table), Version 1.0, December 2004 (Clarification, August 2009). Available at http://rsc.tech-res.com/safetyandpharmacovigilance/

- *Guidelines for Good Practice in the Conduct of Clinical Trials in Human Participants in South Africa*. Available at http://www.doh.gov.za/docs/policy/trials/trials_contents.html

- *Guidelines on Ethics for Medical Research*: *HIV Preventive Vaccine Research.* Available at http://www.mrc.ac.za//ethics/ethicsbook5.pdf

- *HVTN 086 / SAAVI 103 Special Instructions*. Accessible through the HVTN protocol-specific website.

- *HVTN 086 / SAAVI 103 Study Specific Procedures.* Accessible through the HVTN protocol-specific website.

- *HVTN Site Lab Reference Manual*. Accessible through the HVTN website.

- *HVTN Manual of Operations*. Accessible through the HVTN website.

- HVTN algorithm for diagnosis of HIV infections. Part of the *HVTN Laboratory Manual of Operations* (see above).

- International Conference on Harmonisation (ICH) E6 (R1), *Guideline for Good Clinical Practice*: section 4.8, *Informed consent of trial subjects*. Available at http://www.emea.europa.eu/pdfs/human/ich/013595en.pdf.

- *Participants' Bill of Rights and Responsibilities.* Accessible through the HVTN website.

- *Reporting Adverse Drug Reactions in South Africa.* Available at http://www.mccza.com/showdocument.asp?Cat=17&Desc=Guidelines%20-%20Human%20Medicines
- *NIH Guidelines for Research Involving Recombinant DNA Molecules*. Available at http://oba.od.nih.gov/rdna/nih_guidelines_oba.html.
- *NIH Policy on Reporting Race and Ethnicity Data: Subjects in Clinical Research*. Available at http://grants1.nih.gov/grants/guide/notice-files/NOT-OD-01-053.html.
- *Pharmacy Guidelines and Instructions for DAIDS Clinical Trials Networks*, July 2008.
- *Requirements for Source Documentation in DAIDS Funded and/or Sponsored Clinical Trials.* Available at http://www3.niaid.nih.gov/research/resources/DAIDSClinRsrch/ClinicalSite.htm
- *The Manual for Expedited Reporting of Adverse Events to DAIDS,* Version 2.0, January 2010 Available at: http://rsc.tech-res.com/safetyandpharmacovigilance/
- Title 45, Code of Federal Regulations, Part 46. Available at http://www.access.gpo.gov/nara/cfr/waisidx_07/45cfrv1_07.html.

See Section 16 for literature cited in the background and statistics sections of this protocol.

15 Acronyms and abbreviations

Ab	antibody
Ad	adenovirus
ADCC	antibody dependent cellular cytotoxicity
AE	adverse event
ALT	alanine aminotransferase
ANOVA	analysis of variance
ART	antiretroviral therapy
aPTT	activated partial thromboplastin time
AST	aspartate aminotransferase
AUC-MB	area under magnitude-breadth curve
AVEG	AIDS Vaccine Evaluation Group
β-HCG	beta human chorionic gonadotropin
BMI	body mass index
BV	Bacterial vaginosis
CAB	Community Advisory Board
CAPRISA	Center for the AIDS Programme for Research in South Africa
CBC	complete blood count
CDC	US Centers for Disease Control and Prevention
CFR	Code of Federal Regulations
cGMP	clinical good manufacturing practice
CHO	Chinese Hamster Ovary
CI	confidence interval
CK-MB	creatine kinase myocardial band isoenzyme
CMS	Concerned Member State
CPV	canarypox virus
CRF	case report form
CRPMC	NIAID Clinical Research Products Management Center
CRS*	clinical research site
CT	Chlamydia trachomatis
CTL	cytotoxic T lymphocyte
cTnT	cardiac troponin T
DAIDS	Division of AIDS (US NIH)
DHHS	US Department of Health and Human Services
EIA	enzyme immunoassay
ELISA	enzyme-linked immunosorbent assay
ELISpot	enzyme-linked immunospot
FDA	US Food and Drug Administration
FHCRC	Fred Hutchinson Cancer Research Center
FIB	fibrinogen concentration

FPV	fowlpox virus
GCP	Good Clinical Practice
GEE	generalized estimating equation
GLP	Good Laboratory Practice
GMO	Genetically Modified Organism
HBsAG	hepatitis B surface antigen
HCV	hepatitis C virus
HLA	human leukocyte antigen
HSV2	herpes simplex virus type 2
HVTN	HIV Vaccine Trials Network (FHCRC)
IB	Investigator’s Brochure
IBC	Institutional Biosafety Committee
ICH	International Conference on Harmonisation
ICS	intracellular cytokine staining
ID	intradermal
IEC	Independent Ethics Committee
IFN-γ	interferon gamma
IM	intramuscular
IN	intranasal
INS	inhibitory sequence
IRB	Institutional Review Board
IUD	intrauterine device
JEV	Japanese encephalitis virus
Kd	dissociation constant
LDL	low density lipoprotein
LTFU	loss to follow-up
MAR	missing at random
MCAR	missing completely at random
MHC	major histocompatibility complex
MRC-SA	Medical Research Council of South Africa
MMR	measles, mumps, and rubella
MVA	modified vaccinia Ankara
nAb	neutralizing antibody
NG	Neisseria gonorrhoeae
NHP	nonhuman primate
NIAID	National Institute of Allergy and Infectious Diseases (US NIH)
NICD	National Institute for Communicable Diseases (Johannesburg, South Africa)
NIH	US National Institutes of Health
NK	Natural Killer
OBA	NIH Office of Biotechnology Activities
OHRP	US Office for Human Research Protections

OPV	oral polio vaccine
PAB	DAIDS Pharmaceutical Affairs Branch
PBMC	peripheral blood mononuclear cell
PCR	polymerase chain reaction
PI	Principal Investigator
PID	pelvic inflammatory disease
PRO	Protocol Registration Office (DAIDS)
PSRT	Protocol Safety Review Team
PTE	potential T-cell epitope
PTID	participant identification number
RAC	NIH Recombinant DNA Advisory Committee
RSC	DAIDS Regulatory Support Center
RMS	Reference Member State
SAE	serious adverse event
SAAVI	South African AIDS Vaccine Initiative
SC	subcutaneous
SCHARP	Statistical Center for HIV/AIDS Research and Prevention
sd	standard deviation
SD	study day
SDMC	statistical and data management center
SFU	spot-forming unit
SMB	Safety Monitoring Board
SSP	Site Specific Procedures
TB	tuberculosis
UCT	University of Cape Town
VV	vaccinia virus
WBC	white blood cell

* CRSs were formerly referred to as HIV Vaccine Trial Units (HVTUs). Conversion to use of the term CRS is in process, and some HVTN documents may still refer to HVTUs.

16 Literature cited

1. Council for International Organizations of Medical Sciences(CIOMS). International ethical guidelines for biomedical research involving human subjects. Bull Med Ethics **2002**;17-23.

2. UNAIDS. Ethical considerations in HIV preventive vaccine research. **2002**.

3. The National Commission for the Protection of Human Subjects of Biomedical and Behavioral Research. The Belmont Report: Ethical Principles and Guidelines for the Protection of Human Subjects of Research. **1979**.

4. UNAIDS. Report on the global AIDS epidemic. **2008**.

5. Bredell H, Williamson C, Sonnenberg P, Martin DJ, Morris L. Genetic characterization of HIV type 1 from migrant workers in three South African gold mines. AIDS Res Hum Retroviruses **1998**;14:677-84.

6. Van Harmelen JH, Van der RE, Loubser AS, York D, Madurai S, Lyons S, Wood R, Williamson C. A predominantly HIV type 1 subtype C-restricted epidemic in South African urban populations. AIDS Res Hum Retroviruses **1999**;15:395-8.

7. Novitsky V, Smith UR, Gilbert P, McLane MF, Chigwedere P, Williamson C, Ndung'u T, Klein I, Chang SY, Peter T, Thior I, Foley BT, Gaolekwe S, Rybak N, Gaseitsiwe S, Vannberg F, Marlink R, Lee TH, Essex M. Human immunodeficiency virus type 1 subtype C molecular phylogeny: consensus sequence for an AIDS vaccine design? J Virol **2002**;76:5435-51.

8. Guevara H, Johnston E, Zijenah L, Tobaiwa O, Mason P, Contag C, Mahomed K, Hendry M, Katzenstein D. Prenatal transmission of subtype C HIV-1 in Zimbabwe: HIV-1 RNA and DNA in maternal and cord blood. J Acquir Immune Defic Syndr **2000**;25:390-7.

9. Abebe A, Lukashov VV, Pollakis G, Kliphuis A, Fontanet AL, Goudsmit J, de Wit TF. Timing of the HIV-1 subtype C epidemic in Ethiopia based on early virus strains and subsequent virus diversification. AIDS **2001**;15:1555-61.

10. Lole KS, Bollinger RC, Paranjape RS, Gadkari D, Kulkarni SS, Novak NG, Ingersoll R, Sheppard HW, Ray SC. Full-length human immunodeficiency virus type 1 genomes from subtype C-infected seroconverters in India, with evidence of intersubtype recombination. J Virol **1999**;73:152-60.

11. Hemelaar J, Gouws E, Ghys PD, Osmanov S. Global and regional distribution of HIV-1 genetic subtypes and recombinants in 2004. AIDS **2006**;20:W13-W23.

12. Corey L, McElrath MJ, Kublin JG. Post-step modifications for research on HIV vaccines. AIDS **2009**;23:3-8.

13. Buchbinder SP, Mehrotra DV, Duerr A, Fitzgerald DW, Mogg R, Li D, Gilbert PB, Lama JR, Marmor M, Del RC, McElrath MJ, Casimiro DR, Gottesdiener KM, Chodakewitz JA, Corey L, Robertson MN, Robertson MN. Efficacy assessment of a cell-mediated immunity HIV-1 vaccine (the Step Study): a double-blind, randomised, placebo-controlled, test-of-concept trial. Lancet **2008**;372:1881-93.

14. Buchbinder S. Multivariate analyses in the Step Study Or What's the story with HSV2, Ad5 and male circumcision?? HVTN Conference, Seattle, WA, November 18, **2009**.

15. Rerks-Ngarm S, Pitisuttithum P, Nitayaphan S, Kaewkungwal J, Chiu J, Paris R, Premsri N, Namwat C, de Souza M, Adams E, Benenson M, Gurunathan S, Tartaglia J, McNeil JG, Francis DP, Stablein D, Birx DL, Chunsuttiwat S, Khamboonruang C, Thongcharoen P, Robb ML, Michael NL, Kunasol P, Kim JH. Vaccination with ALVAC and AIDSVAX to Prevent HIV-1 Infection in Thailand. N Engl J Med **2009**.

16. Bomsel M, Tudor D, Drillet AS, Alfsen A, Ganor Y, Roger MG, Mouz N, Amacker M, Chalifour A, Diomede L, Devillier G, Cong Z, Wei Q, Gao H, Qin C, Yang GB, Zurbriggen R, Lopalco L, Fleury S. Immunization with HIV-1 gp41 subunit virosomes induces mucosal antibodies protecting nonhuman primates against vaginal SHIV challenges. Immunity **2011**;34:269-80.

17. Baig J, Levy DB, McKay PF, Schmitz JE, Santra S, Subbramanian RA, Kuroda MJ, Lifton MA, Gorgone DA, Wyatt LS, Moss B, Huang Y, Chakrabarti BK, Xu L, Kong WP, Yang ZY, Mascola JR, Nabel GJ, Carville A, Lackner AA, Veazey RS, Letvin NL. Elicitation of simian immunodeficiency virus-specific cytotoxic T lymphocytes in mucosal compartments of rhesus monkeys by systemic vaccination. J Virol **2002**;76:11484-90.

18. Mattapallil JJ, Hill B, Douek DC, Roederer M. Systemic vaccination prevents the total destruction of mucosal CD4 T cells during acute SIV challenge. J Med Primatol **2006**;35:217-24.

19. Williamson C, Morris L, Maughan MF, Ping LH, Dryga SA, Thomas R, Reap EA, Cilliers T, van Harmelen J, Pascual A, Ramjee G, Gray G, Johnston R, Karim SA, Swanstrom R. Characterization and selection of HIV-1 subtype C isolates for use in vaccine development. AIDS Res Hum Retroviruses **2003**;19:133-44.

20. Barnett SW, Srivastava IK, Ulmer JB, Donnelly JJ, Rappuoli R. Development of V2-deleted trimeric envelope vaccine candidates from human immunodeficiency virus type 1 (HIV-1) subtypes B and C. Microbes Infect **2005**;7:1386-91.

21. Srivastava IK, Kan E, Sun Y, Sharma VA, Cisto J, Burke B, Lian Y, Hilt S, Biron Z, Hartog K, Stamatatos L, Diaz-Avalos R, Cheng RH, Ulmer JB, Barnett SW.

Comparative evaluation of trimeric envelope glycoproteins derived from subtype C and B HIV-1 R5 isolates. Virology **2008**;372:273-90.

22. Lian Y, Srivastava I, Gomez-Roman VR, zur MJ, Sun Y, Kan E, Hilt S, Engelbrecht S, Himathongkham S, Luciw PA, Otten G, Ulmer JB, Donnelly JJ, Rabussay D, Montefiori D, van Rensburg EJ, Barnett SW. Evaluation of envelope vaccines derived from the South African subtype C human immunodeficiency virus type 1 TV1 strain. J Virol **2005**;79:13338-49.

23. Barnett SW, Lu S, Srivastava I, Cherpelis S, Gettie A, Blanchard J, Wang S, Mboudjeka I, Leung L, Lian Y, Fong A, Buckner C, Ly A, Hilt S, Ulmer J, Wild CT, Mascola JR, Stamatatos L. The ability of an oligomeric human immunodeficiency virus type 1 (HIV-1) envelope antigen to elicit neutralizing antibodies against primary HIV-1 isolates is improved following partial deletion of the second hypervariable region. J Virol **2001**;75:5526-40.

24. Barnett SW, Srivastava IK, Kan E, Zhou F, Goodsell A, Cristillo AD, Ferrai MG, Weiss DE, Letvin NL, Montefiori D, Pal R, Vajdy M. Protection of macaques against vaginal SHIV challenge by systemic or mucosal and systemic vaccinations with HIV-envelope. AIDS **2008**;22:339-48.

25. Srivastava IK, VanDorsten K, Vojtech L, Barnett SW, Stamatatos L. Changes in the immunogenic properties of soluble gp140 human immunodeficiency virus envelope constructs upon partial deletion of the second hypervariable region. J Virol **2003**;77:2310-20.

26. Xu R, Srivastava IK, Greer CE, Zarkikh I, Kraft Z, Kuller L, Polo JM, Barnett SW, Stamatatos L. Characterization of immune responses elicited in macaques immunized sequentially with chimeric VEE/SIN alphavirus replicon particles expressing SIVGag and/or HIVEnv and with recombinant HIVgp140Env protein. AIDS Res Hum Retroviruses **2006**;22:1022-30.

27. Frey SE, Harrison C, Pass RF, Yang E, Boken D, Sekulovich RE, Percell S, Izu AE, Hirabayashi S, Burke RL, Duliege AM. Effects of antigen dose and immunization regimens on antibody responses to a cytomegalovirus glycoprotein B subunit vaccine. J Infect Dis **1999**;180:1700-3.

28. Graham BS, Keefer MC, McElrath MJ, Gorse GJ, Schwartz DH, Weinhold K, Matthews TJ, Esterlitz JR, Sinangil F, Fast PE. Safety and immunogenicity of a candidate HIV-1 vaccine in healthy adults: recombinant glycoprotein (rgp) 120. A randomized, double-blind trial. NIAID AIDS Vaccine Evaluation Group. Ann Intern Med **1996**;125:270-9.

29. Pitisuttithum P, Nitayaphan S, Thongcharoen P, Khamboonruang C, Kim J, de Souza M, Chuenchitra T, Garner RP, Thapinta D, Polonis V, Ratto-Kim S, Chanbancherd P, Chiu J, Birx DL, Duliege AM, McNeil JG, Brown AE. Safety and immunogenicity of combinations of recombinant subtype E and B human immunodeficiency virus type 1

envelope glycoprotein 120 vaccines in healthy Thai adults. J Infect Dis **2003**;188:219-27.

30. Kim JH, Pitisuttithum P, Kamboonruang C, Chuenchitra T, Mascola J, Frankel SS, DeSouza MS, Polonis V, McLinden R, Sambor A, Brown AE, Phonrat B, Rungruengthanakit K, Duliege AM, Robb ML, McNeil J, Birx DL. Specific antibody responses to vaccination with bivalent CM235/SF2 gp120: detection of homologous and heterologous neutralizing antibody to subtype E (CRF01.AE) HIV type 1. AIDS Res Hum Retroviruses **2003**;19:807-16.

31. Evans TG, Keefer MC, Weinhold KJ, Wolff M, Montefiori D, Gorse GJ, Graham BS, McElrath MJ, Clements-Mann ML, Mulligan MJ, Fast P, Walker MC, Excler JL, Duliege AM, Tartaglia J. A canarypox vaccine expressing multiple human immunodeficiency virus type 1 genes given alone or with rgp120 elicits broad and durable CD8+ cytotoxic T lymphocyte responses in seronegative volunteers. J Infect Dis **1999**;180:290-8.

32. Burgers WA, Chege GK, Muller TL, Van Harmelen JH, Khoury G, Shephard EG, Gray CM, Williamson C, Williamson AL. Broad, high-magnitude and multifunctional CD4+ and CD8+ T-cell responses elicited by a DNA and modified vaccinia Ankara vaccine containing human immunodeficiency virus type 1 subtype C genes in baboons. J Gen Virol **2009**;90:468-80.

33. Amara RR, Nigam P, Sharma S, Liu J, Bostik V. Long-lived poxvirus immunity, robust CD4 help, and better persistence of CD4 than CD8 T cells. J Virol **2004**;78:3811-6.

34. Burgers WA, Van Harmelen JH, Shephard E, Adams C, Mgwebi T, Bourn W, Hanke T, Williamson AL, Williamson C. Design and preclinical evaluation of a multigene human immunodeficiency virus type 1 subtype C DNA vaccine for clinical trial. J Gen Virol **2006**;87:399-410.

35. Burke B, Gomez-Roman VR, Lian Y, Sun Y, Kan E, Ulmer J, Srivastava IK, Barnett SW. Neutralizing antibody responses to subtype B and C adjuvanted HIV envelope protein vaccination in rabbits. Virology **2009**;387:147-56.

36. Thongcharoen P, Suriyanon V, Paris RM, Khamboonruang C, de Souza MS, Ratto-Kim S, Karnasuta C, Polonis VR, Baglyos L, Habib RE, Gurunathan S, Barnett S, Brown AE, Birx DL, McNeil JG, Kim JH. A phase 1/2 comparative vaccine trial of the safety and immunogenicity of a CRF01_AE (subtype E) candidate vaccine: ALVAC-HIV (vCP1521) prime with oligomeric gp160 (92TH023/LAI-DID) or bivalent gp120 (CM235/SF2) boost. J Acquir Immune Defic Syndr **2007**;46:48-55.

37. Francis DP, Heyward WL, Popovic V, Orozco-Cronin P, Orelind K, Gee C, Hirsch A, Ippolito T, Luck A, Longhi M, Gulati V, Winslow N, Gurwith M, Sinangil F, Berman PW. Candidate HIV/AIDS vaccines: lessons learned from the World's first phase III efficacy trials. AIDS **2003**;17:147-56.

38. Pitisuttithum P, Gilbert P, Gurwith M, Heyward W, Martin M, van Griensven F, Hu D, Tappero JW, Choopanya K. Randomized, Double-Blind, Placebo-Controlled Efficacy Trial of a Bivalent Recombinant Glycoprotein 120 HIV-1 Vaccine among Injection Drug Users in Bangkok, Thailand. J Infect Dis **2006**;194:1661-71.

39. Lewis DJ, Huo Z, Barnett S, Kromann I, Giemza R, Galiza E, Woodrow M, Thierry-Carstensen B, Andersen P, Novicki D, Del Giudice G, Rappuoli R. Transient facial nerve paralysis (Bell's palsy) following intranasal delivery of a genetically detoxified mutant of Escherichia coli heat labile toxin. PLoS One **2009**;4:e6999.

40. Keefer MC, Graham BS, McElrath MJ, Matthews TJ, Stablein DM, Corey L, Wright PF, Lawrence D, Fast PE, Weinhold K, Hsieh RH, Chernoff D, Dekker C, Dolin R. Safety and immunogenicity of Env 2-3, a human immunodeficiency virus type 1 candidate vaccine, in combination with a novel adjuvant, MTP- PE/MF59. NIAID AIDS Vaccine Evaluation Group. AIDS Res Hum Retroviruses **1996**;12:683-93.

41. Graham BS, Belshe RB, Clements ML, Dolin R, Corey L, Wright PF, Gorse GJ, Midthun K, Keefer MC, Roberts NJ, Jr., et al. Vaccination of vaccinia-naive adults with human immunodeficiency virus type 1 gp160 recombinant vaccinia virus in a blinded, controlled, randomized clinical trial. The AIDS Vaccine Clinical Trials Network. J Infect Dis **1992**;166:244-52.

42. Heineman TC, Clements-Mann ML, Poland GA, Jacobson RM, Izu AE, Sakamoto D, Eiden J, Van Nest GA, Hsu HH. A randomized, controlled study in adults of the immunogenicity of a novel hepatitis B vaccine containing MF59 adjuvant. Vaccine **1999**;17:2769-78.

43. Bernstein DI, Schleiss MR, Berencsi K, Gonczol E, Dickey M, Khoury P, Cadoz M, Meric C, Zahradnik J, Duliege AM, Plotkin S. Effect of previous or simultaneous immunization with canarypox expressing cytomegalovirus (CMV) glycoprotein B (gB) on response to subunit gB vaccine plus MF59 in healthy CMV-seronegative adults. J Infect Dis **2002**;185:686-90.

44. Podda A. The adjuvanted influenza vaccines with novel adjuvants: experience with the MF59-adjuvanted vaccine. Vaccine **2001**;19:2673-80.

45. De Donato S, Granoff D, Minutello M, Lecchi G, Faccini M, Agnello M, Senatore F, Verweij P, Fritzell B, Podda A. Safety and immunogenicity of MF59-adjuvanted influenza vaccine in the elderly. Vaccine **1999**;17:3094-101.

46. Ott G, Barchfeld GL, Chernoff D, Radhakrishnan R, van Hoogevest P, Van Nest G. MF59. Design and evaluation of a safe and potent adjuvant for human vaccines. Pharm Biotechnol **1995**;6:277-96.

47. Gilbert PB, Chiu YL, Allen M, Lawrence DN, Chapdu C, Israel H, Holman D, Keefer MC, Wolff M, Frey SE. Long-term safety analysis of preventive HIV-1 vaccines

evaluated in AIDS vaccine evaluation group NIAID-sponsored Phase I and II clinical trials. Vaccine **2003**;21:2933-47.

48. Hawkridge T, Scriba TJ, Gelderbloem S, Smit E, Tameris M, Moyo S, Lang T, Veldsman A, Hatherill M, Merwe L, Fletcher HA, Mahomed H, Hill AV, Hanekom WA, Hussey GD, McShane H. Safety and immunogenicity of a new tuberculosis vaccine, MVA85A, in healthy adults in South Africa. J Infect Dis **2008**;198:544-52.

49. Gomez CE, Najera JL, Krupa M, Esteban M. The poxvirus vectors MVA and NYVAC as gene delivery systems for vaccination against infectious diseases and cancer. Curr Gene Ther **2008**;8:97-120.

50. Gudmundsdotter L, Nilsson C, Brave A, Hejdeman B, Earl P, Moss B, Robb M, Cox J, Michael N, Marovich M, Biberfeld G, Sandstrom E, Wahren B. Recombinant Modified Vaccinia Ankara (MVA) effectively boosts DNA-primed HIV-specific immune responses in humans despite pre-existing vaccinia immunity. Vaccine **2009**;27:4468-74.

51. Liu W. On sample size determination of Dunnett's procedure for comparing several treatments with a control. Journal of Statistical Planning and Inference **1997**;62:255-61.

52. Agresti A, Coull BA. Approximate is better than "exact" for interval estimation of binomial proportions. Am Stat **1998**;52:199-256.

53. Hudgens MG. Estimating cumulative probabilities from incomplete longitudinal binary responses with application to HIV vaccine trials. Statistics in Medicine **2003**;22:463-79.

54. Lachenbruch PA. Comparisons of two-part models with competitors. Stat Med **2001**;20:1215-34.

55. Huang Y GPMDSS. Simultaneous evaluation of the magnitude and breadth of a left- and right-censored multivariate response, with application to HIV vaccine development. Statistics in Biopharmaceutical Research **2009**;1:81-91.

56. Hughes JP. Mixed effects models with censored data with application to HIV RNA levels. Biometrics **1999**;55:625-9.

57. Rotnitzky A, Robins J. Analysis of semi-parametric regression models with non-ignorable non-response. Stat Med **1997**;16:81-102.

58. Eckart RE, Love SS, Atwood JE, Arness MK, Cassimatis DC, Campbell CL, Boyd SY, Murphy JG, Swerdlow DL, Collins LC, Riddle JR, Tornberg DN, Grabenstein JD, Engler RJ. Incidence and follow-up of inflammatory cardiac complications after smallpox vaccination. J Am Coll Cardiol **2004**;44:201-5.

59. Smith SC, Ladenson JH, Mason JW, Jaffe AS. Elevations of cardiac troponin I associated with myocarditis. Experimental and clinical correlates. Circulation **1997**;95:163-8.

60. Sano J, Chaitman BR, Swindle J, Frey SE. Electrocardiography screening for cardiotoxicity after modified Vaccinia Ankara vaccination. Am J Med **2009**;122:79-84.

61. Update: cardiac-related events during the civilian smallpox vaccination program--United States, 2003. MMWR Morb Mortal Wkly Rep **2003**;52:492-6.

Appendix A: Sample informed consent form

Title: A phase 1 placebo-controlled clinical trial to evaluate the safety and immunogenicity of SAAVI DNA-C2, SAAVI MVA-C and Novartis subtype C gp140 with MF59 adjuvant in various vaccination schedules in HIV-uninfected healthy vaccinia-naïve adult participants in South Africa

Short title: SAAVI/Novartis Study

Site: [Insert site name]

Thank you for considering being a participant in this research study to test the safety and immune response of these HIV vaccines.

We will require you to read this form or have it read to you. Please take your time in deciding if you wish to join this study. Talk to people you trust such as your friends, family, or doctor if it helps you decide. We will test your understanding of the information in this form to make sure we have explained everything clearly. If you decide to join the study, we will ask you to sign this form. You will get a copy to keep.

About the study

The HIV Vaccine Trials Network (HVTN), the South African AIDS Vaccine Initiative (SAAVI) and Novartis Vaccines and Diagnostics, Inc., together with [Site: insert name of researcher and institution] are conducting a research study to find a preventive HIV vaccine (HIV stands for the human immunodeficiency virus). HIV is the virus that causes AIDS (acquired immune deficiency syndrome). Vaccines are given to teach the body to prevent infection or fight disease.

About 184 people will take part in this study at 3 sites in South Africa. Participants will be men and women between 18 and 45 years old. The researcher in charge of this study at this clinic is [Insert name of site PI]. The US National Institutes of Health (NIH) is paying for the study.

1. **We are doing this study to answer several questions.**

   - Are the study vaccines safe to give to people?
   - Are people able to take the study vaccines without becoming too uncomfortable?
   - How do people's immune systems respond to the study vaccines? (Your immune system protects you from disease.)

   Remember research is not treatment or medical care.

2. **The study vaccines cannot give you HIV.**

   The study vaccines are not made from actual HIV. It is impossible for the study vaccines to give you HIV.

3. **We do not know if the study vaccines will decrease, increase, or not change your chance of becoming infected with HIV if you are exposed to the virus.**

   You can still get HIV infection even if you join this study. You should not do things that could expose you to HIV. We will show you how to lower your risk.

   A large HIV vaccine study in Thailand tested the combination of a canary pox-based HIV vaccine and a protein-based HIV vaccine. The study showed the vaccines are safe. Also, the vaccines were modestly effective in lowering the chance of people getting HIV infection.

   Another study (the Step Study) tested a different HIV vaccine that contained adenovirus type 5 (Ad5). In the Step Study, men who had antibodies to Ad5 and then got the study vaccine had more HIV infections than those who got the placebo. Men who were not circumcised and then got the study vaccine were also more likely to become HIV infected.

   We do not know why the Step Study vaccine seemed to increase the risk of these men becoming infected with HIV. Very few women became infected with HIV in the study. As a result, we cannot tell what effect the vaccine had on a woman's chance of getting HIV. We do know that the study vaccine did not give anyone HIV. People in the Step Study got HIV from another person.

   In South Africa, another study (called Phambili) used the same experimental vaccines as the Step Study. We stopped injections early in the Phambili Study when the Step Study results were announced. We told participants about the potential increased risk of HIV infection. We told them if they received the study vaccine or placebo. Once they learned this, some people could have changed their HIV risk behaviors. In the Phambili study, with continued follow-up, the number of people who became infected in the vaccine arm has become similar to the number in the placebo arm. We are not sure if this is due to decreased risk behaviour in people who received the vaccine or not. We are still studying these outcomes.

   This study (SAAVI/Novartis) uses different study vaccines than those in the Step Study. The clinic staff here can tell you more about the HIV vaccines used in this study and how they compare to the HIV vaccines in the Thai, Phambili, and Step studies. However, even with these differences, we do not know if the study vaccines, or any other experimental HIV vaccine, will change your risk of getting HIV if you are exposed.

4. **These study vaccines are experimental.**

   The South African Medicines Control Council (MCC) has not approved them for any other use. The study vaccines are called SAAVI DNA-C2 and SAAVI MVA-C and Novartis subtype C gp140/MF59. From here on, we will call them the DNA vaccine, the MVA vaccine, and the gp140 vaccine, or the "study vaccines." They are experimental HIV vaccines. That means we do not know whether the vaccines will be totally safe to use in people, or whether they will work to prevent HIV infection.

   The DNA and MVA vaccines were developed by scientists at the University of Cape Town, South Africa, for the SAAVI, a lead program of the Medical Research Council, South Africa.

The DNA vaccine, SAAVI DNA-C2, is made out of DNA. DNA is a natural substance found in all living things, including people and viruses. DNA tells cells to make proteins. In this study, the DNA vaccine will tell your body to make a small amount of some proteins that are found in HIV. Your body's immune system may recognize these proteins and prepare itself to fight HIV. This is called an immune response. The DNA vaccine is similar to natural DNA, but it was made in a laboratory.

The MVA vaccine, SAAVI MVA-C, was made from a virus called modified vaccinia Ankara (MVA) virus. It is similar to the smallpox vaccine that has been used worldwide. The MVA virus in the vaccine has been changed so that it cannot grow in humans or spread to other people. Like the DNA vaccine, the MVA vaccine will tell your body to make small amounts of some proteins that are found in HIV. These proteins may cause your body to have an immune response.

The DNA and MVA study vaccines have been tested in people in South Africa and people in the US in a study called HVTN 073 / SAAVI 102. HVTN 073 / SAAVI 102 has 48 people in the study. The vaccines have not caused any serious health concerns.

The gp140 vaccine is made by Novartis Vaccines and Diagnostics, Inc. (Novartis). Like the DNA and MVA vaccines, the gp140 vaccine will tell your body to make small amounts of protein that look like part of HIV. These proteins may cause your body to have an immune response (called an antibody response) that is a little different than the immune response that the DNA and MVA vaccines might cause.

The exact gp140 vaccine that will be used in this study has not been given to people before. It has been tested in mice, rabbits, and monkeys and it did not cause any health concerns. Animal testing may not always predict what will happen with humans. Novartis has done human testing on a very similar gp140 vaccine to the one used in this study. That similar gp140 vaccine has not shown any serious health concerns.

This will be the first time the gp140 vaccine used in this study will be tested with the DNA or MVA vaccines used in this study.

Sometimes a substance is given with a vaccine to help make a person's immune response stronger. This is called an adjuvant. The gp140 vaccine will use an adjuvant called MF 59. MF 59 has been used with a gp140 vaccine similar to one of the vaccines in this study. It has been approved for use with a vaccine licensed in Europe. Also, it has been used with other vaccines and given to over 20,000 people without causing any health concerns.

Joining the study

5. It is completely up to you whether or not to join the study.

Take your time in deciding. If it helps, talk to people you trust, such as your doctor, friends or family. If you decide not to join this study, or if you leave it after you have joined, your other care at this clinic and the benefits or rights you would normally have will not be affected.

If you join this study, you may not be allowed to join other HIV vaccine or HIV prevention studies now or in the future. Also during the study, you should not donate blood.

If you choose not to join this study, you could join another study if one is available and you are eligible. If you are interested, we can tell you about other HIV vaccine and prevention studies that we know of.

6. If you decide to join the study, we will screen you to see if you are eligible.

Screening involves a physical exam, HIV test and health history. A physical exam may include, but is not limited to:

- Checking your weight, temperature and blood pressure
- Looking in your mouth and throat
- Listening to your heart and lungs
- Feeling your abdomen (stomach and liver)
- Men: asking if you are circumcised

We will also do blood and urine tests. These tests tell us about some key aspects of your health, such as how healthy your kidneys, liver, and immune system are. We will also test you for these infectious diseases: syphilis, Hepatitis B, and Hepatitis C. We will ask you about medications you are taking. We will ask you about behaviors that might put you at risk for getting HIV. If you were born female, we will test you for pregnancy.

To make sure your heart is healthy we will conduct a blood test to check for heart injury. This test will also let us see if there are any changes in your heart health later in the study. We will also do another heart test called an electrocardiogram (ECG, also known as EKG). For the ECG, we will place leads (suction cups, or stickers) on your chest, arms and legs and you will need to lie still for several seconds.

We will review the screening results with you and offer you counseling and referral if you need medical care. The screening results may show you are not eligible to join the study, even if you want to. You cannot be in another research study where you receive a study product and be enrolled in this study.

7. If you were born female and could become pregnant, you must agree to use birth control to join this study.

You must agree to use effective birth control from 21 days (3 weeks) before your first injection until 90 days (3 months) after your last injection. We will talk to you about effective birth control methods. They are listed on a handout that we will give to you. If you join the study, we will test you for pregnancy periodically, including before each study injection.

Being in the study

If you are eligible to join the study after screening, and still want to participate, here is what will happen:

8. You will come to the clinic about [#] times over [Insert period of time].

Site: Insert number of visits and range of visit lengths. (There is site-specific variation in screening protocols and in the number of possible follow-up visits between protocol-mandated visits.)

Non injection visits should take no longer than one hour. Injection visits can take between 2-4 hours. We will provide you with refreshments during these extended visits.

You may have to come for more visits if you have a laboratory or health issue.

9. When you finish your scheduled clinic visits, we will continue to stay in contact with you for safety monitoring.

We also want to learn about the long-term safety of the vaccines. Extended safety monitoring begins after the scheduled clinic visits are completed. In this part of the study, we will contact you, using your preferred mode of contact, once each year to check on your health. This will continue for 2 years.

There is a separate consent form for this extended safety monitoring part of the study.

10. We will give you [Site: Insert compensation] for each study visit you complete.

This payment for scheduled visits is to cover the cost of transport to and from the clinic, refreshments, and possibly some of the time spent in the clinic. There are no plans to share any profits with you if the study vaccines later become approved and are sold or if studies on your blood lead to a new discovery.

Site: Insert any costs to participants (eg, birth control costs for female participants who could become pregnant).

11. We will give you either the study vaccines or a placebo.

Not everyone in this study will get the study vaccines. Some people will get only a placebo, which is sterile salt water that does not contain vaccine. We will compare the results from people who got the placebo with results from people who got the study vaccines.

Whether you receive the study vaccines or only the placebo will be decided randomly. This is like deciding by tossing a coin or rolling dice. You will have an 80% chance of receiving the study vaccines.

The reason we are testing the study vaccines is because we do not know whether they work or are safe. That means we do not know whether it is better to get the vaccine or to get the placebo. In either case, you need to take steps to protect yourself from HIV infection.

The clinic staff and your healthcare provider have no say in whether you get the study vaccines or the placebo. They will not know which one you are getting, and neither will you. Only the pharmacist at your site will have this information while the study is going on, and he or she will keep it a secret.

You will have to wait until all participants complete their final study visits to find out whether you got the study vaccines or the placebo. This could be several years. But, if you have a serious medical problem and need to know what you got before the end of the study, we can tell you.

12. We will give you the study products on a schedule.

You will be in one of 4 groups. You will get 7 injections during the study by needle into the upper arm(s). At some visits you will receive 2 injections, 1 in each arm (see Appendix I more details). You have a 25% chance of being assigned to any particular group.

HVTN 086 / SAAVI 103 Participant Injection Schedule

	First Injection Visit	Month 1	Month 3	Month 6
Group 1	Placebo + MVA vaccine	MVA vaccine	gp140 vaccine + Placebo	gp140 vaccine + Placebo
	or	or	or	or
	Placebo + Placebo	Placebo	Placebo + Placebo	Placebo + Placebo
Group 2	gp140 vaccine + MVA vaccine	Placebo	gp140 vaccine + MVA vaccine	Placebo + Placebo
	or	or	or	or
	Placebo + Placebo	Placebo	Placebo + Placebo	Placebo + Placebo
Group 3	Placebo + DNA vaccine	DNA vaccine	Placebo + MVA vaccine	Placebo + MVA vaccine
	or	or	or	or
	Placebo + Placebo	Placebo	Placebo + Placebo	Placebo + Placebo
Group 4	Placebo + DNA vaccine	DNA vaccine	gp140 vaccine + MVA vaccine	gp140 vaccine + MVA vaccine
	or	or	or	or
	Placebo + Placebo	Placebo	Placebo + Placebo	Placebo + Placebo

You will have to wait in the clinic for about a half hour to an hour after each injection to see if there are any problems. Then for that night and for three more days, you will need to write down your symptoms. On each of these days, we will need to hear how you are feeling. It is very important to stay in touch with the clinic staff. We will ask you what is the best way to be in contact with you.

If you have a problem, we will continue to check on you until it goes away.

13. In addition to giving you the study products, we will perform these procedures:

- Regular HIV testing, as well as counseling on your results and on how to avoid getting HIV;
- Physical exams;
- Collection of blood and urine samples;
- Collection of rectal fluids (this is optional);
- Collection of cervical fluids if you were born female (this is optional);
- Testing for certain infections that may be sexually transmitted (if you were born female and agree to the optional collection of rectal and/or cervical fluids);
- Pregnancy tests if you were born female;
- Questions about your health, including medications you may be taking;
- Personal questions about your HIV risk, including sexual behavior and drug use;
- Questions about any personal problems or benefits you may have from participating in the study.

Site: Paste table of procedures in this section or distribute it as a separate sheet if it is helpful to your study participants.

- We will review the results of these procedures and tests with you at your next visit, or sooner if necessary. We will also offer you counseling and referral for needed care.

14. If you agree, we will collect rectal and cervical fluids

Because most people are exposed to HIV on their penis, vagina, or rectum, it is important to learn more about vaccine effects in these locations and in other similar locations. For this reason, we want to collect cervical and rectal fluids before you receive your first injection and at your clinic visit at month 6.5. We would only do this if you agree and are able to provide the samples.

For women, if you agree, we will collect cervical fluid. This will only be done if you are not pregnant and not having your period. We will do a pregnancy test, must be negative, before each cervical fluid collection. We will also do tests for the following infections:

gonorrhea, Chlamydia, Bacterial Vaginosis, Trichomoniasis, and Herpes Simplex Virus-2. We will explain each of these infections to you. We will do these tests at the same time as the cervical fluid collection. If any of these tests show that you have an infection, we will give you counseling and treatment, or we will refer you for treatment when appropriate. In addition, you must have had a Pap smear within the last 3 years with the most recent result being normal. (If you haven't had a Pap smear within the last 3 years and would like to get one, we will tell you where you can get one.) We will ask you not to douche or insert anything with spermicide into the vagina for 48 hours before the samples are collected. Also, we will ask you not to have vaginal sex and/or insert anything into the vagina for 24 hours before the samples are collected. This will help make sure the samples you provide give accurate lab readings. To collect cervical fluid, we will insert a speculum (a device that opens the vagina) into your vagina. Then we will place one or two small sponges in the opening of the cervix for about 1 minute to absorb the fluid.

If you agree, we will collect rectal fluids by placing one or two small absorbent sponges in the rectum for about 5 minutes each. An anoscope, a plastic viewing tube 2-3 inches long and ½ inch wide, may be inserted into the rectum so that the clinician can see better when doing this procedure. We will ask you not to have unprotected anal sex for 24 hours before providing these samples. This will help make sure the samples you provide give accurate lab readings.

At the end of this consent form, we will ask you if you allow us to collect rectal and cervical fluids. You can decide not to give these samples and still be in the study. You can decide to provide some of these samples and not others. If you agree to provide these samples and change your mind, you can withdraw your consent for these procedures at any time during the study.

15. We will test your samples for reactions to the study products.

In this study, we will need to take blood from you with a needle on several occasions. The amount will depend on the lab tests we need to do. It will be some amount between 20 mL and 205 mL (4 teaspoons to 1 cup). Your body will make new blood to replace the blood we take out.

Site: You may want to add a sentence to the end of the previous paragraph contextualizing the blood volumes described (eg, "To compare, people who donate blood in the US can give a total of about 500 mL in an 8-week period."). Modify the example for cultural relevance and alter blood volumes as necessary.

We will use some of your samples to see if you have side effects from the study products. We will share these results with you.

We also will send your samples (without your name) to a lab to see how the immune system responds to the study products. While most of your samples will be stored in South Africa, some may be shipped to the US for some tests, including genetic testing. These tests are for research purposes only. The lab will not give the results to you or this clinic, and the results will not become part of your study record.

After this testing, we will continue to store your samples in case we need to repeat any tests for this study.

16. If you agree, we will store and use any of your extra samples and share limited information about you for other research.

This related information may include things such as your sex, age, ethnicity, and health history. The samples and information could be used to help researchers understand the immune system, HIV, and other diseases. This research may include genetic testing.

At the end of this form, we will ask you if you agree to donate your extra samples and limited information for other research. What you decide will not affect your participation in this study or any care you receive here.

If you do not want to donate your extra samples, they will be destroyed when this study is finished. If you choose to donate your extra samples for use in other research that means you are giving them to us. There is no limit on how long these samples will be stored.

You can change your mind about donating your extra samples at any time. If that happens, tell us and we will request that any remaining stored samples be destroyed.

Your donated samples and information may be shared with researchers in the US and in other countries. We will not sell your samples or data, and we will not pay you for them. We will not share any information that would make it easy for the researchers to identify you.

The researchers will not report their results to you, this clinic, or your doctor. The results will not appear in your medical or study record. Any other research with your samples and data will not benefit you personally. It is not needed for your medical care. Instead, the research will be done to help the public through new scientific discoveries.

We cannot predict exactly how your extra samples and limited information will be used if you choose to donate them. Any research using these samples or information would be reviewed by an IRB/ethics committee at the institution of the researcher requesting the samples or information and a local South African ethics committee. These committees will consider the proposed research and determine whether your rights and well-being will be adequately protected.

Site: Add text informing participants of any national storage requirements for their blood samples. Also, list any relevant in-country repositories.

17. We will do our best to protect your private information.

Your study records and samples will be kept in a secure location. We will label all of your samples and most of your records with a code number, not your name or other personal information. However, it is possible to identify you, if necessary. We will not share your name with the lab that does the tests on your samples, or with anyone else who does not need to know your name.

Research staff will have access to your study records and your samples. Copies of your data will be shared with people at the Fred Hutchinson Cancer Research Center in the US where the study results are analyzed. Your records may also be reviewed by groups who watch over this study, such as the ethics committee/IRB and the South African Medicines

Control Council. We can give you a list of the people who will be able to access your study data if you want it. All reviewers will take steps to keep your records private.

We cannot guarantee absolute privacy. Information about you may be released if you have an infectious disease (like Tuberculosis or Syphilis) that we must report to the health department or if required by law.

Site: Include any public health or legal reporting requirements. Bulleted examples should include all appropriate cases (reportable communicable disease, risk of harm to self or others, etc.).

- [Item 1]
- [Item 2]
- [Item 3]

The results of this study, and other studies that use the samples or information you agree to donate, may be published. No publication will use your name or identify you personally.

18. We may stop your injections or take you out of the study at any time.

This may happen if:

- you do not follow instructions,
- the researcher thinks that staying in the study might harm you,
- you get HIV,
- you enroll in a different research study where you receive another study product,
- you have missed too many injections, or
- the study is stopped for any reason.

We may stop your injections or take you out of the study even if you want to continue and even if you were scheduled for additional injections.

19. If you become pregnant during the study, we will continue with some procedures but not injections.

We will do this for as long as it is safe for you and your developing baby. If you leave the study while you are still pregnant, we will contact you after your due date to ask some questions about your pregnancy and delivery.

20. If you get infected with HIV during the study, we will help you get care and support.

You will not be able to stay in this study. We will counsel you about your HIV infection and about telling your partner(s). We will tell you where you can get support and medical care, and about other studies you may want to join. We will not provide or pay for any of your HIV care directly.

Risks

21. There are risks to being in this study.

This section describes the risks and restrictions we know about. There may also be unknown risks, even serious ones. We will tell you if we learn anything new that may affect your willingness to stay in the study.

Risks of routine medical procedures:

In this study, we will do some routine medical procedures. These are taking blood and giving injections. These procedures can cause bruising, pain, fainting, soreness, redness, swelling, itching, muscle damage, and (rarely) infection where the needle was inserted. Taking blood can cause a low blood cell count (anemia), making you feel tired.

General risks of vaccines:

Rarely, a vaccine can cause an allergic reaction, including a rash, hives, or difficulty breathing. **Allergic reactions can be life-threatening**. You should tell us if you have ever had a bad reaction to any injection or vaccine.

All vaccines can cause fever, chills, rash, aches and pains, nausea, headache, dizziness, and feeling tired. Most people can still do their planned activities after getting a vaccine. Rarely, people experience side effects that limit their normal activities or make them go to the doctor.

Risks of the study vaccines:

The DNA and MVA vaccines used in this study are being tested now in a study called HVTN 073 / SAAVI 102. This study is being done in South Africa and the US The study has 48 participants getting vaccines or placebos. The study has had no serious health concerns. Most of the reactions from the injections have been mild. Only one person has complained of severe arm pain and one person has complained of severe headache. Because these people may be receiving placebo, we do not know if these severe effects were related to the vaccines.

This is the first study where the DNA vaccine and the MVA vaccine will be used in the same study with the gp140 vaccine. We do not know what all of the possible risks are. We expect these products to cause no serious health concerns.

Risks of the DNA vaccine:

Possible risks related to DNA vaccines include

- muscle damage and
- insertion of the vaccine DNA into the body's DNA, leading to cancer, or
- autoimmune disease, where the immune system fights against the body.

We think the risk of these things happening are low. More than 1000 people have been given DNA vaccines being tested against HIV and none of these things has happened so far.

Since 1995, thousands of people have received experimental DNA vaccines for diseases such as hepatitis, human papilloma virus (HPV, also known as genital warts), and HIV. In these people, the DNA vaccines also have not caused serious side effects.

We expect the risks of the DNA vaccine in this study to be similar to those of other DNA vaccines. However, there may be new side effects that we don't know about.

Risks of the MVA vaccine:

The MVA vaccine is being tested with the DNA vaccine in HVTN 073 / SAAVI 102 in South Africa and the US. No serious health problems have occurred.

The MVA vaccine is like the smallpox vaccine that has been used all over the world. The smallpox vaccine may cause heart problems. Studies using different experimental MVA vaccines against HIV have been given to over 300 healthy people. These people have not had any heart problems or serious side effects.

Although heart problems are very unlikely, they could still happen with this MVA vaccine. If you join the study, we will ask you about symptoms related to heart problems. Let us know right away if you are having any problems like extreme tiredness, chest pain, or difficulty breathing.

If we suspect a heart problem during the study, we may do additional testing to check your heart. We may refer you to a heart doctor for diagnosis and treatment. We will keep in close touch with you until the problem is over. We will ask you to sign a form to allow us to review your medical records for the heart problem.

Risks of the gp140 vaccine and the adjuvant MF 59:

The exact gp140 vaccine that will be used in this study has not been given to people before. It has been tested in mice, rabbits, and monkeys and it did not cause any health concerns. Animal testing may not always predict what will happen with humans. Novartis has done human testing on a very similar gp140 vaccine to the one used in this study. That similar gp140 vaccine has not shown any serious health concerns.

This will be the first time the gp140 vaccine used in this study will be tested with the DNA or MVA vaccines used in this study.

MF 59 has been used with a gp140 vaccine very similar to the one in this study. MF 59 has also been used with other vaccines and given to over 20,000 people without causing any health concerns.

Risks of collecting rectal and cervical fluids

Collection of cervical fluids may cause some discomfort. This discomfort is similar to what happens during a routine Pap smear. It does not usually last very long.

Collection of rectal fluids may involve use of an anoscope. The anoscope is a plastic viewing tube, 2-3 inches long and ½ inch wide, which may be inserted into the rectum so that the clinician can see better when doing this procedure. This may cause temporary discomfort.

We will ask you to stop some behaviors related to your rectum and genitals for a short time before we collect samples from these areas. You may find this inconvenient.

During any of these procedures, you may feel anxious or embarrassed. We will try to make you as comfortable as possible.

Personal problems/discrimination/testing HIV antibody positive:

About 10 to 20% of HVTN participants report personal problems or discrimination because of joining an HIV vaccine study. Family or friends may worry, get upset or angry, or assume that you are infected with HIV or at high risk and treat you unfairly as a result. Rarely, a participant has lost a job because the study took too much time away from work, or because their employer thought they had HIV.

The study vaccines are likely to cause you to test positive on some types of HIV tests. This means that after you get the study vaccines, a routine HIV test may say you have HIV, even if you don't. For this reason, you should plan to get HIV tests only at this clinic during the study.

If you receive a positive test result caused by the vaccines at any time, we can provide you with free HIV testing for as long as you need it. If this happens, we do not know how long you will stay positive due to the study vaccines. If you receive a positive HIV test result and we determine it is because you have HIV, we will refer you for care.

It is unlikely, but you could test negative at the end of the study and positive some time later, even though you don't have HIV. This could happen if different HIV tests come into use. If you are concerned about this possibility, we will give you a phone number to call. This way you can get more information before you get an HIV test after you leave the study.

If someone believes you are infected with HIV even if you are not, you could face discrimination and other problems. For example, you could be denied medical or dental care, employment, insurance, a visa, or entry into the military. If you do have a positive HIV antibody test caused by the study vaccines, you will not be able to donate blood or organs. Your family and friends may treat you differently. We can help you with any problems you face because of a positive antibody test that is caused by the study

vaccines. We can give you a card with a number you can call for help with these issues. We can also give you a letter and/or a brochure explaining your situation.

Embarrassment/anxiety:

You may feel embarrassed when we ask about your HIV risks, such as having sex and using drugs. Also, waiting for your HIV test results or other health test results could make you feel anxious. You could feel worried if your test results show that you are infected with HIV or another infection. If you feel embarrassed or anxious, please tell us and we will try to help you.

Risks of disclosure of your personal information:

We will take several steps to protect your personal information. Although the risk is very low, it is possible that your personal information could be given to someone who should not have it. If that happened, you could face discrimination, stress, and embarrassment. We can tell you more about how we will protect your personal information if you would like it.

Unknown risks:

We do not know if the study vaccines will have a good or bad effect, or no effect at all, on your risk of becoming infected with HIV if exposed. If you get infected with HIV, we do not know how the study vaccines might affect your HIV infection or how long it takes to develop AIDS.

We do not know if getting these study vaccines will affect how you respond to any future approved HIV vaccine. It could be that a future HIV vaccine may not work as well for you because you got the study vaccines. Currently, no HIV vaccine has been approved for use.

We do not know how the study vaccines will affect a pregnant participant or a developing baby.

Benefits

22. The study may not benefit you.

We do not know whether getting the study vaccines might benefit you in any way. However, being in the study might help you in some ways. The counseling that you get as part of the study may help you avoid getting HIV. The lab tests and physical exams that you get while in this study might detect health problems you don’t yet know about.

This study may help in the search for a vaccine to prevent HIV. However, if the study vaccines later become approved and sold, there are no plans to share any money with you. You will also not receive any money if you decide to donate your extra samples or data for other research, even if this research leads to a new product or discovery.

Your rights and responsibilities

23. If you join the study, you have rights and responsibilities.

As a participant, you have many rights that we respect. You also have responsibilities. We will give you the Participant's Bill of Rights and Responsibilities (PBORR). It describes your rights and responsibilities as a study participant.

Leaving the study

24. Tell us if you decide to leave the study.

You are free to leave the study at any time and for any reason. Your care at this clinic and your legal rights will not be affected, but it is important for you to let us know.

We will ask you to come back to the clinic one last time for a physical exam, and we may ask to take some blood and urine samples. We will also ask about any personal problems or benefits you have experienced from being in the study. We believe these steps are important to protecting your health, but it is up to you whether to complete them.

Injuries

25. If you get sick or injured during the study, contact us immediately.

Study-related injury or illness is one that occurs as a direct result of the administration of the study vaccines or study-related procedures. The clinic staff will treat you for study-related problems or tell you where you can get the treatment you need. If a study-related injury occurs you have not waived any of the legal rights which you otherwise would have as a participant in this study by signing this form.

If you get sick or injured because of the study vaccines, an insurance has been purchased to cover for your medical treatment. This policy will follow the guidelines for payment of study-related illness or injury approved by the Association of the British Pharmaceutical Industry ("ABPI Guidelines"). You can get a copy of these ABPI Guidelines from us if you wish.

Some injuries are not physical. For example, someone might be harmed psychologically or emotionally by being in an HIV vaccine study. Or they might lose wages from injuries because they could not go to work. No funds have been set aside to pay for nonphysical injuries, even if they are related to participation in the study.

You and/or your health insurance carrier will continue to be responsible for the cost of your usual medical care outside this study, and for medical expenses that are determined not directly related to study procedures or products. You will not be giving up any of your rights by signing this consent form.

MEDICINES CONTROL COUNCIL SOUTH AFRICA – MCC

If you have questions about this study you should first discuss them with your study doctor or the ethics committee (contact details as provided on this form). After you have consulted your doctor or the ethics committee and if they have not provided you with answers to your satisfaction, you should write to the Medicines Control Council (MCC) South Africa at:

The Registrar
Medicines Control Council SA
Department of Health
Private Bag X828
Pretoria
0001

ETHICAL APPROVAL:

This clinical study protocol has been submitted to the *[site insert ethics committee name]* and written approval has been granted by that committee.

The study has been structured in accordance with the Declaration of Helsinki (last updated: October 2000), which deals with the recommendations guiding doctors in biomedical research involving human participants, and the Ethics in Health Research: Principles, Structures and Processes (2004) and Guidelines for Good Practice in the Conduct of Clinical Trials in Human Participants in South Africa.

We can provide you copies if you wish to review them.

The study is sponsored by:

South African AIDS Vaccine Initiative (SAAVI), Medical Research Council of South Africa (MRC-SA), Cape Town, Republic of South Africa

Questions

26. If you have questions or problems at any time during your participation in this study, use the following important contacts.

If you have questions about this study, contact
[name and telephone number of the investigator or other study staff].

If you have any symptoms that you think may be related to this study, contact
[name and telephone number of the investigator or other study staff].

If you have questions about your rights as a research participant, or problems or concerns about how you are being treated in this study, contact
[name/title/phone of person on IRB or other appropriate organization].

If you want to leave this study, contact
[name and telephone number of the investigator or other study staff].

Your signature

Study title: A phase 1 placebo-controlled clinical trial to evaluate the safety and immunogenicity of SAAVI DNA-C2, SAAVI MVA-C and Novartis subtype C gp140 with MF59 adjuvant in various vaccination schedules in HIV-uninfected healthy vaccinia-naïve adult participants in South Africa

In section 14 of this form, we told you about collection of cervical and rectal fluid samples, which is optional. You can decide not to give rectal and cervical fluid and still be in this study. You may change your mind about giving rectal and cervical fluid samples and still stay in the study. Please write your initials or make your mark in the boxes next to the options you choose.

	I agree to provide cervical fluid samples
	I do not agree to provide cervical fluid samples

	I agree to provide rectal fluid samples
	I do not agree to provide rectal fluid samples

In section 16 of this form, we told you about possible other uses of your extra samples outside this study. Please write your initials or make your mark in the box next to the option you choose.

	I agree to donate my extra samples and limited information for other research related to diseases and human immune system. This may include genetic testing.
	I do not agree to donate my extra samples and limited information for other research.

27. Before you sign this consent form, make sure of the following:

- You have read this consent form, or someone has read it to you.
- You feel that you understand what the study is about and what will happen to you if you join. You understand what the possible risks and benefits are.
- You have had your questions answered and know that you can ask more.
- You will not be giving up any of your rights by signing this consent form.

Participant's name (print)	Participant's signature or mark	Date	Time
Clinic staff conducting consent discussion (print)	Clinic staff signature	Date	Time

For participants who are unable to read or write, also complete the signature block below:

Witness's name (print)[#]	Witness's signature	Date	Time

[#] Witness is impartial and was present for the consent process.

Appendix B: Sample informed consent form for extended safety monitoring

Title: A phase 1 placebo-controlled clinical trial to evaluate the safety and immunogenicity of SAAVI DNA-C2, SAAVI MVA-C and Novartis subtype C gp140 with MF59 adjuvant in various vaccination schedules in HIV-uninfected healthy vaccinia-naïve adult participants in South Africa

Short title: SAAVI/Novartis Study

Site: [Insert site name]

Thank you for taking part in the SAAVI/Novartis study. Previously, we said we would ask permission to contact you once a year for 2 years after you complete your 12 months of scheduled clinic visits. In this consent form, we are asking you for that permission. These additional contacts are part of the regular study. Like always, it is totally up to you whether you want to remain in the study. You can leave at any time without negative consequences.

Please read this consent form or ask someone to read it to you. If you agree to have us contact you, we will ask you to sign this form. We will offer you a copy to keep. We will ask you questions to see if you understand the information in this form. You can also ask us questions as well.

About safety monitoring contacts

About 184 people will be enrolled in HVTN 086 / SAAVI 103. We are asking all participants to give us permission for annual follow-up contact for safety monitoring. The researcher in charge of this study at this clinic is [Insert name of site PI] .

We want to learn about possible long term safety effects of the HVTN 086 / SAAVI 103 study vaccines. If you agree, we will email or call *[site: modify mode of contact as appropriate]* once a year for 2 years after you have completed your 12 months of scheduled clinic visits.

When we contact you, we will ask you questions about your health since you were last contacted. This will include whether you have had any new illness that required extended medical care and whether you have become disabled or have been hospitalized. If you can become pregnant, we will ask you about any pregnancies and deliveries you may have had.

If you prefer to answer these questions in person, an appointment with the study clinic can be arranged.

If someone outside this study clinic told you that you are infected with HIV since our last contact with you, we will ask you to come back to the clinic for another HIV test. If you get an HIV test outside of this clinic, the results may appear positive if you received a study vaccine. Our tests can tell the difference between a true HIV infection and a positive test result that is caused by a study vaccine. If we test you we will draw about 15 mL (1 tablespoon) of blood.

If you are confirmed to have become HIV-infected, we will help you get care and support. We will tell you where you can get support and medical care, and about other studies you may want to join. We will not provide or pay for any of your HIV care directly.

Because we will want to contact you once a year, please stay in touch with us. Tell us if your address or phone number changes, if you are moving away, or if you do not want us to contact you anymore.

Keeping in touch will also help us contact you if there is new safety information about the study product(s) you received. We might also ask if you are interested in participating in any new studies.

Site: modify the next sentence to reflect what you are planning re: compensation for follow-up contacts.

We will give you *[Insert compensation]* for each study contact.

This amount is to cover the costs of *[Site: Insert text].*

We will do our best to protect your private information.

We will protect information from safety monitoring contacts in the same way we protect your other study information. Your study information will be kept in a secure location. We will label most of your information with a code number, not your name or other personal information.

Research staff will have access to your study information. Copies of your study information will be shared with people at the Fred Hutchinson Cancer Research Center in the US where the study results are analyzed. Your study information may also be reviewed by groups who watch over this study, such as the ethics committee/IRB and the Medicines Control Council (MCC). We can give you a list of the people who will be able to access your study information if you want it. All reviewers will take steps to keep your information private.

We cannot guarantee absolute privacy. At this clinic, we have to report the following information:

Site: Include any public health or legal reporting requirements. Bulleted examples should include all appropriate cases (reportable communicable disease, risk of harm to self or others, etc.).

- [Item 1]
- [Item 2]
- [Item 3]

US sites: Include the following boxed text. You can remove the box.

Researchers who use your information will also do their best to protect your private information. The information they receive will be labeled with a code number. They will

not have your name or any personal information. Any reviewers of those studies will take steps to keep your information private.

The results of this study, and other studies that use the samples or information you agree to donate, may be published. No publication will use your name or identify you personally.

Risks and benefits

This section describes the risks and restrictions we know about. There may be others we don't know about. We will tell you if we learn anything new that may affect your willingness to continue with safety monitoring contacts.

Embarrassment/anxiety:

You may feel embarrassed or anxious when we ask about your health. You may feel embarrassed or anxious when we ask if you have been diagnosed with HIV infection. If you feel embarrassed or anxious, please tell us and we will try to help you.

Risks of disclosure of your personal information:

We will take several steps to protect your personal information. Although the risk is very low, it is possible that your personal information could be given to someone who should not have it. If that happened, you could face discrimination, stress, and embarrassment. We can tell you more about how we will protect your personal information if you would like it.

Side effects of having blood taken for an HIV test, if one is performed:

You could faint or feel lightheaded from a blood draw. The needle used for drawing blood can cause pain and bruising. Rarely, some people get an infection where the needle was put in their arm to draw the blood.

Benefits

Safety monitoring contacts may not benefit you. If we learn something from these contacts that might affect your health, these contacts might help us get that information to you.

Stopping safety monitoring contacts

You can decide at any time that you don't want any more safety monitoring contacts. If you do so, you will not lose any benefits or rights you would normally have.

If you decide you do not want any more safety monitoring contacts, please tell the clinic staff.

Questions

If you have questions or concerns at any time during extended safety monitoring, use the following contacts:

If you have questions about safety monitoring, contact
[name and telephone number of the investigator or other study staff].

If you have any symptoms that you think may be related to this study, contact
[name and telephone number of the investigator or other study staff].

If you have questions about your rights as a research participant, or problems or concerns about how you are being treated, contact
[name/title/phone of person on IRB or other appropriate organization].

If you want to stop participation in safety monitoring, contact
[name and telephone number of the investigator or other study staff].

Your signature

Study title: A phase 1 placebo-controlled clinical trial to evaluate the safety and immunogenicity of SAAVI DNA-C2, SAAVI MVA-C and Novartis subtype C gp140 with MF59 adjuvant in various vaccination schedules in HIV-uninfected healthy vaccinia-naïve adult participants in South Africa

Before you sign this consent form, make sure of the following:

- You have read this consent form, or someone has read it to you.
- You have had your questions answered.
- You understand you can ask more questions at any time.
- You understand your study information will be available to the doctors, staff, and other groups working on the study.
- You agree to extended safety monitoring.

You will not be giving up any of your rights by signing this consent form.

Participant's name (print)	Participant's signature or mark	Date	Time
Clinic staff conducting consent discussion (print)	Clinic staff signature	Date	Time

For participants who are unable to read or write, also complete the signature block below:

Witness's name (print)[#]	Witness's signature	Date	Time

[#] Witness is impartial and was present for the consent process.

Appendix C: Approved birth control methods (for sample informed consent form)

You should not become pregnant during the study because we do not know how the study vaccines could affect the developing baby.

If you were born female and are sexually active in a way that could lead you to get pregnant, you must agree to use effective birth control, starting at least 3 weeks before you get your first injection of study vaccines or placebo and continuing until 90 days (3 months) after your last injection, as specified in the informed consent.

- Effective contraception for participants of this study in South Africa is defined as using **2 methods**, including 1 of the following:
    - Condoms (male or female) with or without a spermicide,
    - Diaphragm or cervical cap with spermicide,

PLUS 1 of the following methods;
    - Intrauterine device (IUD)
    - Hormonal contraception

You do not have to use birth control if:

- You are only having sex with a partner or partners who have had a vasectomy. (We will ask you some questions to confirm that the vasectomy was successful.);
- You have reached menopause, with no menstrual periods for one year;
- You have had a hysterectomy (your uterus removed);
- You have had your ovaries removed;
- You have a tubal ligation (your “tubes tied”) or confirmed successful placement of a product that blocks the fallopian tubes;
- You are having sex only with a female partner or partners;
- You only have oral sex; or,
- You are sexually abstinent (no sex at all).

Remember: If you are having sex, you need to use male or female condoms to protect yourself from HIV infection.

Appendix D: Table of procedures (for sample informed consent form)

Procedure	Screening visit	First injection visit	Time after first injection visit in months (and days or weeks)											
			0.03 (Day 1)	0.5 (Week 2)	1 (Week 4)	1.5 (Week 6)	3 (Week 12)	3.25 (Week 13)	3.5 (Week 14)	6 (Week 24)	6.25 (Week26)	6.5 (Week 28)	9 (Week 36)	12 (Week 48)
Injection		√			√		√			√				
Medical history	√													
Complete physical	√													√
Abbreviated physical		√		√	√	√	√	√	√	√	√	√	√	
ECG+	√													
Blood draw	√	√		√		√	√	√	√		√	√	√	√
Urine test	√			√								√		
Pregnancy test (participants born female)	√	√			√		√			√			√	
HIV testing/counseling	√	√		√	√				√	√		√	√	√
Mucosal secretion samples (rectal, cervical)*		√										√		
Cervical/Vaginal swab‡		√										√		
Interview/questionnaire	√	√		√	√	√	√	√	√	√	√	√	√	√
Risk reduction counseling	√	√		√	√	√	√	√	√	√	√	√	√	√

+ The ECG may be repeated at later visits in some cases.

* Participation in the collection of mucosal secretion samples is optional

‡ Cervical/Vaginal swab collection only required for female participants who consent to optional mucosal sample collection.

Grayed out column indicates a visit that is no longer required

Not shown in this table is a time after all study participants have completed their last scheduled visit when you can find out what products you received. After we tell you what products you received, we will contact you once each year to ask you some questions about your health. This will continue for 2 years after you complete your 12 months of scheduled clinic visits.

Appendix E: Laboratory procedures

				Tube volume (mL)														
			Visit:	**1**	**2**	**3**	**4**	**5**	**6**	**7**	**8**	**9**	**10**	**11**	**12**	**13**	**14**	
			Day:	Screening	D0	D1	D14	D28	D42	D84	D91	D98	D168	D175	D182	D273	D364	
			Month:	visit[4]	M0		M0.5	M1	M1.5	M3	M3.25	M3.5	M6	M6.25	M6.5	M9	M12	
**Description**	**Ship to[2]**	**Assay location[3]**	**Tube[5]**		**VAC1**			**VAC2**		**VAC3**			**VAC4**					**Total**
BLOOD COLLECTION																		
Screening or diagnostic assays																		
Screening HIV test	Local lab	Local lab	SST	5	—	—	—	—	—	—	—	—	—	—	—	—	—	**5**
HBsAg/anti-HCV/Syphilis	Local lab	Local lab	SST	5	—	—	—	—	—	—	—	—	—	—	—	—	—	**5**
HIV diagnostic algorithm[10]	SV/SMDU-NICD	SV/SMDU-NICD	EDTA	—	—	—	10	—	—	—	—	10	—	—	10	10	20	**60**
Safety labs																		
CBC/ Diff/ platelets	Local lab	Local lab	EDTA	5	—	—	5	—	5	—	—	5	—	—	5	5	—	**30**
Chemistry panel[6]	Local lab	Local lab	SST	5	—	—	5	—	5	—	—	5	—	—	5	5	—	**30**
Cardiac troponin	Local lab	Local lab		5	—	—	5	—	5	—	—	5	—	—	5	—	—	**25**
Immunogenicity assays[7]																		
HLA typing[8]	CSR	FHCRC	ACD	—	—	—	—	—	—	—	20	—	—	—	—	—	—	**20**
Humoral Assays																		
HIV-1 multiplex ab assay	CSR	Duke	EDTA	—	10	—	10	—	10	—	—	*10*	—	—	*10*	—	10	**60**
HIV neut ab assay	CSR	Duke / SAIL-NICD	SST	—	10	—	10	—	10	—	—	*10*	—	—	*10*	—	10	**60**
Cellular Assays																		
ICS	CSR	FHCRC	ACD	—	40	—	40	—	40	—	—	*40*	—	—	*40*	—	40	**240**
B cell phenotyping	CSR	FHCRC	ACD	—	10	—	10	—	10	—	10	*10*	—	10	*10*	—	10	**80**
NK cell function	CSR	FHCRC	ACD	—	10	—	10	—	10	—	10	*10*	—	10	*10*	—	10	**80**
Storage																		
Serum storage	CSR	—	SST	—	20	—	15	—	15	—	—	20	—	—	20	—	20	**110**
Plasma storage	CSR	—	EDTA	—	10	—	10	—	10	—	—	20	—	—	20	—	20	**90**
Plasma storage[1]	CSR	—	ACD	—	z	—	z	—	z	—	—	z	—	—	z	—	z	**0**
PBMC storage	CSR	—	ACD	—	40	—	30	—	20	—	—	60	—	—	60	—	60	**270**
**Maximum Total**				**25**	**150**	**0**	**160**	**0**	**140**	**0**	**40**	**205**	**0**	**20**	**205**	**20**	**200**	**1165**
**Maximum 56-Day total**				**25**	**175**	**175**	**335**	**335**	**475**	**140**	**180**	**385**	**0**	**20**	**225**	**20**	**200**	
URINE COLLECTION																		
Urinalysis	Local lab	Local lab	—	X	—	—	X	—	—	—	—	—	—	—	X	—	—	
Pregnancy test[9]	Local lab	Local lab	—	X	X	—	—	X	—	X	—	—	X	—	X[11]	X	—	
CERVICAL/VAGINAL SWAB[12]																		
NG/CT/T. vaginalis//BV/HSV-2[13]	Local lab	Local lab	—	—	X	—	—	—	—	—	—	—	—	—	X	—	—	
MUCOSAL COLLECTION																		
Rectal secretion (optional)	CSR	Duke	—	—	X	—	—	—	—	—	—	—	—	—	X	—	—	
Cervical secretion (optional)	CSR	Duke	—	—	X	—	—	—	—	—	—	—	—	—	X	—	—	

Grayed out column indicates a visit that is no longer required

[1] z = 5mL of plasma extracted from ACD blood being processed for PBMCs at the site processing lab

[2] CSR = central specimen repository

[3] HVTN Laboratory Program includes laboratories at Duke, FHCRC, SV/SMDU-NID, and SAIL-NCID; Duke = Duke University Medical Center (Durham, North Carolina, USA); FHCRC = Fred Hutchinson Cancer Research Center (Seattle, Washington, USA); SV/SMDU-NICD = Serology-Virology & Special Molecular Diagnostic Unit, National Institute for Communicable Diseases (Johannesburg, South Africa); SAIL-NICD = South African Immunology Laboratory - National Institute for Communicable Diseases (Johannesburg, South Africa)

[4] Screening may occur over the course of several contacts/visits up to and including day 0 prior to vaccination.

[5] Local labs may assign appropriate alternative tube types for locally performed tests.

[6] Chemistry panels are defined in section 9.2 *Pre-enrollment procedures* (pre-enrollment) and section 9.4 *Follow-up visits* (postenrollment).

[7] Based on the number of responders observed at the primary immunogenicity timepoints (visits 9 (M 3.5) and 12(M 6.5)), also visit 2 (M0) for the HIV-1 multiplex Ab assay, lab assays may be performed on all participants for humoral and cellular responses at visits 2, 3, 5, 7, 10 and/or 13.

[8] Genotyping may be performed on enrolled participants using cryopreserved PBMC, initially in participants who demonstrate vaccine-induced T-cell responses at postvaccination timepoints.

[9] Pregnancy test may be performed on blood specimens.

[10] At an early termination visit for a withdrawn or terminated participant (see section 9.12), blood should be drawn for HIV diagnostic testing, as shown for visit 14 above.

[11] Pregnancy test performed only if the participant is participating in cervical secretion collection.

[12] HSV-2 and Cervical/Vaginal swab collection only required for female participants who consent to optional mucosal sample collection.

[13] HSV-2 testing may alternatively be performed with serum samples from storage.

Appendix F: Procedures at HVTN CRS

Visit:	01[a]	02	03	04	05	06	07	08	09	10	11	12	13	14	Post
Day:		D0	D1	D14	D28	D42	D84	D91	D98	D168	D175	D182	D273	D364	
Month:		M0		M0.5	M1	M1.5	M3	M3.25	M3.5	M6	M6.25	M6.5	M9	M12	
Procedure	Scr.	VAC1			VAC2		VAC3			VAC4					
**Study procedures[b]**															
Signed screening consent (if used)	X	—	—	—	—	—	—	—	—	—	—	—	—	—	—
Assessment of understanding	X	—	—	—	—	—	—	—	—	—	—	—	—	—	—
Signed protocol consents	X	—	—	—	—	—	—	—	—	—	—	—	—	—	—
Medical history	X	—	—	—	—	—	—	—	—	—	—	—	—	—	—
Complete physical exam	X	—	—	—	—	—	—	—	—	—	—	—	—	X	—
Abbreviated physical exam	—	X	—	X	X	X	X	X	X	X	X	X	X	—	—
Risk reduction counseling	X	X	—	X	X	X	X	X	X	X	X	X	X	X	—
ECG[c]	X	—	—	—	—	—	—	—	—	—	—	—	—	—	—
Pregnancy prevention assessment[d]	X	X	—	X	X	X	X	X	X	X	X	X	X	X	—
Behavioral risk assessment	X	—	—	—	—	—	—	—	—	—	—	—	—	—	—
Confirm eligibility, obtain demographics, randomize	X	—	—	—	—	—	—	—	—	—	—	—	—	—	—
Social impact assessment	—	X	—	X	X	X	X	X	X	X	X	X	X	X	—
Social impact assessment questionnaire	—	—	—	—	—	—	X	—	—	X	—	—	—	X	—
Outside testing and belief questionnaire	—	—	—	—	—	—	—	—	—	X	—	—	—	X	—
Concomitant medications	X	X	—	X	X	X	X	X	X	X	X	X	X	X	—
Intercurrent illness/adverse experience	—	X	—	X	X	X	X	X	X	X	X	X	X	X	—
Cardiac symptoms assessment	—	X	—	X	X	X	X	X	X	X	X	X	X	X	—
HIV infection assessment[e]	X	—	—	X	—	—	—	—	X	—	—	X	X	X	—
Confirm HIV test results provided to participant	—	X	—	—	X	—	—	—	—	X	—	—	X	X	X
Mucosal secretion collection (rectal, cervical) (optional)[f]	—	X	—	—	—	—	—	—	—	—	—	X	—	—	—
**Local lab assessment**															
Urine dipstick	X	—	—	X	—	—	—	—	—	—	—	X	—	—	—
Pregnancy (urine or serum HCG)[g]	X	X	—	—	X	—	X	—	—	X	—	X	X	—	—
CBC, differential, platelet	X		—	X	—	X	—	—	X	—	—	X	X	—	—
Chemistry panel (see Sections 9.2 and 9.4)	X	—	—	X	—	X	—	—	X	—	—	X	X	—	—
cTnT	X	—	—	X		X	—	—	X	—	—	X	—	—	—
Syphilis, Hepatitis B, Hepatitis C	X	—	—	—	—	—	—	—	—	—	—	—	—	—	—
HSV-2 (cervical/vaginal swab or serum) (female)[h]	—	X	—	—	—	—	—	—	—	—	—	X	—	—	—
Cervical/Vaginal swab (NG, CT, T. vaginalis, BV) (female)[i]	—	X	—	—	—	—	—	—	—	—	—	X	—	—	—
**Vaccination procedures**															
Vaccination[j]	—	X	—	—	X	—	X	—	—	X	—	—	—	—	—
Reactogenicity assessments[k]	—	X	—	—	X	—	X	—	—	X	—	—	—	—	—
**Poststudy**															
Unblind participant	—	—	—	—	—	—	—	—	—	—	—	—	—	—	X

Grayed out column indicates a visit that is no longer required

[a] Screening may occur over the course of several contacts/visits up to and including day 0 prior to vaccination.
[b] For specimen collection requirements, see Appendix E.
[c] ECG and Cardiac Troponin tests are required at screening. ECG, Cardiac Troponin, and CK-MB may also be performed at additional timepoints as clinically indicated.
[d] Pregnancy prevention compliance occurs only with participants who were born female and are capable of becoming pregnant.
[e] Includes pre-test counseling. A subsequent follow-up contact is conducted to provide post-test counseling and to report results to participant.
[f] Mucosal secretion samples not to be collected if participant meets any of the conditions described in section 9.5
[g] For a participant who was born female, pregnancy test must be performed on the day of vaccination prior to vaccination. Pregnancy test to determine eligibility may be performed at screening or on day 0 prior to first vaccination. Serum pregnancy tests may be used to confirm the results of, or substitute for, a urine pregnancy test.
[h] HSV-2 only required for female participants who consent to optional mucosal sample collection.
[i] Cervical/Vaginal swab collection only required for female participants who consent to optional mucosal secretion sample collection. NG = *Neisseria gonorrhoeae*, CT = *Chlamydia trachomatis*, T. vaginalis = *Trichomonas vaginalis*, BV = Bacterial vaginosis.
[j] Blood draws required at vaccination visits must be performed prior to administration of study product; however, it is not necessary to have results prior to administration. Lab tests may be drawn within the 3 days prior to vaccination.
[k] Reactogenicity assessments performed daily for at least 3 days postvaccination (see section 9.9).

Appendix G: Extended Safety Surveillance

Contact[a]	ESS 03	ESS 04
Day	728	1092
Month	24	36
**Procedures**		
Vital status and extended safety surveillance[b]	X	X

[a] Clinic visits are not required, except that any participant reporting a diagnosis of HIV infection will be asked to come to the clinic so that HIV status can be confirmed.

[b] See section 9.5

ESS = Extended Safety Surveillance

Appendix H: Case definition of myo/pericarditis for use in adverse events monitoring

Myo/pericarditis

Myo/pericarditis is defined as a spectrum of disease caused by inflammation of the myocardium and/or pericardium. Patients might have symptoms and signs consistent with myocarditis, pericarditis, or both. For the purpose of surveillance reporting, patients with myocarditis or pericarditis will be reported as having myo/pericarditis. These categories are intended for surveillance purposes and not for use in individual diagnosis or treatment decisions.

Case Definition for Acute Myocarditis

A suspected case of acute myocarditis is defined by the following criteria and the absence of evidence of any other likely cause of symptoms or findings below:

- Presence of dyspnea, palpitations, or chest pain of probable cardiac origin in a patient with either one of the following:
  - Electrocardiogram (ECG) abnormalities beyond normal variants, not documented previously, including
    - ST-segment or T-wave abnormalities,
    - Paroxysmal or sustained atrial or ventricular arrhythmias,
    - AV nodal conduction delays or intraventricular conduction defects, or
    - Continuous ambulatory electrocardiographic monitoring that detects frequent atrial or ventricular ectopy

    or
  - Evidence of focal or diffuse depressed left-ventricular (LV) function of indeterminate age identified by an imaging study (e.g., echocardiography or radionuclide ventriculography).

A probable case of acute myocarditis, in addition to the above symptoms and in the absence of evidence of any other likely cause of symptoms, has one of the following:

- Elevated cardiac enzymes, specifically, abnormal levels of cardiac troponin I, troponin T, or creatine kinase myocardial band (a troponin test is preferred);
- Evidence of focal or diffuse depressed LV function identified by an imaging study (e.g., echocardiography or radionuclide ventriculography) that is documented to be of new onset or of increased degree of severity (in the absence of a previous study, findings of depressed LV function are considered of new onset if, on follow-up studies, these findings resolve, improve, or worsen); or
- Abnormal result of cardiac radionuclide imaging (e.g., cardiac MRI with gadolinium or gallium-67 imaging) indicating myocardial inflammation.

A case of acute myocarditis is confirmed if histopathologic evidence of myocardial inflammation is found at endomyocardial biopsy or autopsy.

Case Definition for Acute Pericarditis

A suspected case of acute pericarditis is defined by the presence of

- Typical chest pain (i.e., pain made worse by lying down and relieved by sitting up and/or leaning forward) and
- No evidence of any other likely cause of such chest pain.

A probable case of acute pericarditis is a suspected case of pericarditis, or a case in a person with pleuritic or other chest pain not characteristic of any other disease, that, in addition, has one or more of the following:

- Pericardial rub, an auscultatory sign with one to three components per beat,
- ECG with diffuse ST-segment elevations or PR depressions without reciprocal ST depressions that are not previously documented, or
- Echocardiogram indicating the presence of an abnormal collection of pericardial fluid (e.g., anterior and posterior pericardial effusion or a large posterior pericardial effusion alone).

A case of acute pericarditis is confirmed if histopathologic evidence of pericardial inflammation is evident from pericardial tissue obtained at surgery or autopsy.

Source: MMWR 2003; 52 (21); 492-496

Appendix I: HVTN 086 / SAAVI 103 Participant Injection Schedule

	FIRST INJECTION VISIT	MONTH 1	MONTH 3	MONTH 6
GROUP 1				
	Left arm: Placebo<br>Right arm: MVA-C	Right arm: MVA-C	Left arm: gp140<br>Right arm: Placebo	Left arm: gp140<br>Right arm: Placebo
	Left arm: Placebo<br>Right arm: Placebo	Right arm: Placebo	Left arm: Placebo<br>Right arm: Placebo	Left arm: Placebo<br>Right arm: Placebo
GROUP 2				
	Left arm: gp140<br>Right arm: MVA-C	Right arm: Placebo	Left arm: gp140<br>Right arm: MVA-C	Left arm: Placebo<br>Right arm: Placebo
	Left arm: Placebo<br>Right arm: Placebo	Right arm: Placebo	Left arm: Placebo<br>Right arm: Placebo	Left arm: Placebo<br>Right arm: Placebo
GROUP 3				
	Left arm: Placebo<br>Right arm: DNA-C2	Right arm: DNA-C2	Left arm: Placebo<br>Right arm: MVA-C	Left arm: Placebo<br>Right arm: MVA-C
	Left arm: Placebo<br>Right arm: Placebo	Right arm: Placebo	Left arm: Placebo<br>Right arm: Placebo	Left arm: Placebo<br>Right arm: Placebo
GROUP 4				
	Left arm: Placebo<br>Right arm: DNA-C2	Right arm: DNA-C2	Left arm: gp140<br>Right arm: MVA-C	Left arm: gp140<br>Right arm: MVA-C
	Left arm: Placebo<br>Right arm: Placebo	Right arm: Placebo	Left arm: Placebo<br>Right arm: Placebo	Left arm: Placebo<br>Right arm: Placebo

Appendix J: HVTN VISP registry consent

The HIV Vaccine Trials Network (HVTN) would like your permission to enter your name and link it to information about you in a computer registry (the “VISP registry”). By having your name and vaccine study information in the VISP registry, trained HVTN staff can quickly help you if you have problems with VISP.

About VISP

The body makes antibodies to prevent infection. Most vaccines cause the body to make antibodies as a way of preventing infection. Your body may make antibodies to HIV because you received an HIV vaccine. Some HIV test results could come back positive even if you are not infected with HIV. This is called a VISP (vaccine-induced seropositive) test result. We do not know who will have VISP test results or how long these test results may last.

People with VISP test results need specific HIV tests. These tests can tell whether a test result is VISP or a real HIV infection. These people may need help explaining their VISP situation if someone outside the study wants to test them for HIV. VISP test results may cause problems in several areas like insurance, job applications, the military, prison, visa applications, emigration/immigration, and blood and tissue donation.

We are asking you for your permission to enter your name in the registry now in case you have VISP test results later. The registry will not be used for any other purpose.

What are the benefits of the registry?

Your study site will help you with problems related to VISP test results. If you are unable to go to your original study site, an HVTN counselor will help you with these problems. The HVTN counselor will need to verify your study participation and if you received an HIV vaccine. The registry gives the HVTN counselor quick access to this information.

If you choose not to have your name entered in the registry, HVTN counselors still will do their best to help you. However, it will take longer to get that information. If your study site is no longer doing HIV vaccine studies, your records may be stored securely off site. It is possible your records may not be found.

What information does the registry contain and how is it protected?

The registry contains the following information:

- Your participant ID (the code used for you instead of your name at your study site)
- The study network and study you were in
- The site where you began the study
- The date you began the study
- Your date of birth or age
- If you received an HIV vaccine that may cause you to test VISP

We are asking for your permission to enter your name into the registry and link it to the information above.

The registry will NOT contain:

- Your HIV test results
- Your phone number or any other way to contact you

Any other personal information that you discuss with the HVTN counselor will be kept separate from the registry. We will keep your name in the registry until you tell us you want it removed.

All people who work with your registry information sign agreements to keep the information confidential.

The registry is a secured computer database. It can only be accessed with a password.

What are the risks?

The only risk to having your name entered and linked to the other pieces of information in the registry is that someone who is not authorized might see your information. The risk of this happening is low because of the security protections in place. However, we cannot guarantee this will never happen.

What if I have more questions about the registry?

Please talk to your study site or call the phone number they provided if you have any questions about the registry now or in the future.

If I agree now, can I change my mind later?

Yes. You can contact your study site or call the number you have been given anytime to tell us that you would like your name to be deleted from the registry. We will send you confirmation that we deleted it. Your decision will not affect your participation in the main HIV vaccine study.

By signing this form, you do not give up any legal rights.

Please write your initials or make your mark in the box next to the option you choose.

☐ I **AGREE** to allow my name to be entered and linked to the information in the HVTN VISP registry.

☐ I **DO NOT AGREE** to allow my name to be entered and linked to the information in the HVTN VISP registry.

Please sign or make your mark below.

Participant's name (print)	Participant's signature or mark	Date	Time
Study staff conducting consent discussion (print)	Study staff signature	Date	Time

For participants who are unable to read or write, a witness should complete the signature block below:

Witness's name (print)[#]	Witness's signature	Date	Time

Witness is impartial and was present for the consent process.
